# Trends of long noncoding RNA research from 2007 to 2016: a bibliometric analysis

**DOI:** 10.18632/oncotarget.20851

**Published:** 2017-09-12

**Authors:** Yan Miao, Si-Yi Xu, Lu-Si Chen, Ge-Yu Liang, Yue-Pu Pu, Li-Hong Yin

**Affiliations:** ^1^ Key Laboratory of Environmental Medicine Engineering, Ministry of Education, School of Public Health, Southeast University, Nanjing, Jiangsu 210009, P.R. China; ^2^ Department of Epidemiology and Health Statistics, School of Public Health, Southeast University, Nanjing, Jiangsu 210009, P.R. China

**Keywords:** lncRNA, citation, bibliometric, CiteSpace IV, WoSCC

## Abstract

**Purpose:**

This study aims to analyze the scientific output of long noncoding RNA (lncRNA) research and construct a model to evaluate publications from the past decade qualitatively and quantitatively.

**Methods:**

Publications from 2007 to 2016 were retrieved from the Web of Science Core Collection database. Microsoft Excel 2016 and CiteSpace IV software were used to analyze publication outputs, journals, countries, institutions, authors, citation counts, ESI top papers, H-index, and research frontiers.

**Results:**

A total of 3,008 papers on lncRNA research were identified published by June 17, 2017. The journal, *Oncotarget* (IF2016, 5.168) ranked first in the number of publications. China had the largest number of publications (1,843), but the United States showed its dominant position in both citation frequency (45,120) and H-index (97). Zhang Y (72 publications) published the most papers, and Guttman M (1,556 citations) had the greatest co-citation counts. The keyword “database” ranked first in research frontiers.

**Conclusion:**

The annual number of publications rapidly increased in the past decade. China showed its significant progress in lncRNA research, but the United States was the actual leading country in this field. Many Chinese institutions engaged in lncRNA research but significant collaborations among them were not noted. Guttman M, Mercer TR, Rinn JL, and Gupta RA were identified as good candidates for research collaboration. “Database,” “Xist RNA,” and “Genome-wide association study” should be closely observed in this field.

## INTRODUCTION

Long noncoding RNAs (lncRNAs) belong to a large class of non-protein coding transcribed RNA molecules with a length more than 200 nucleotides [1]. The length threshold is a simple but convenient biophysical cutoff that separates lncRNAs from smaller noncoding RNA species (e.g. miRNAs, siRNAs) [2, 3]. In the past decade, lncRNAs have attracted investigators’ much attention because the cumulative evidence indicated that lncRNAs play a pivotal role in multiple biological processes based on a variety of underlying mechanisms, including epigenetic regulation [4], organ or tissue development [5], cell development and differentiation [6], protein transport [7], gene transcription [8], chromatin remodeling [9], and metabolic processes [10]. Meanwhile, increasing evidence suggested that dysregulations and mutations of these lncRNAs were associated with development and progression of various human diseases, such as lung cancer [11], liver cancer [12], colon cancer [13], and breast cancer [14].

The research interest on lncRNA has increased dramatically in recent years, and many academic journals have published articles on lncRNA research. Nevertheless, few attempts have been made to analyze the evolution of scientific output in this field systematically. Bibliometrics is a good choice of method to analyze the literature of a scientific domain, and assess trends in research activity over time [15].

The objects of this study are to systematically evaluate lncRNA research from 2007 to 2016, to determine the publication pattern of lncRNA research outputs, to capture the collaboration pattern between countries/institutions/authors, and to identify research trends and frontiers in this field.

## RESULTS

### Publication outputs and growth prediction

**Figure 1:**
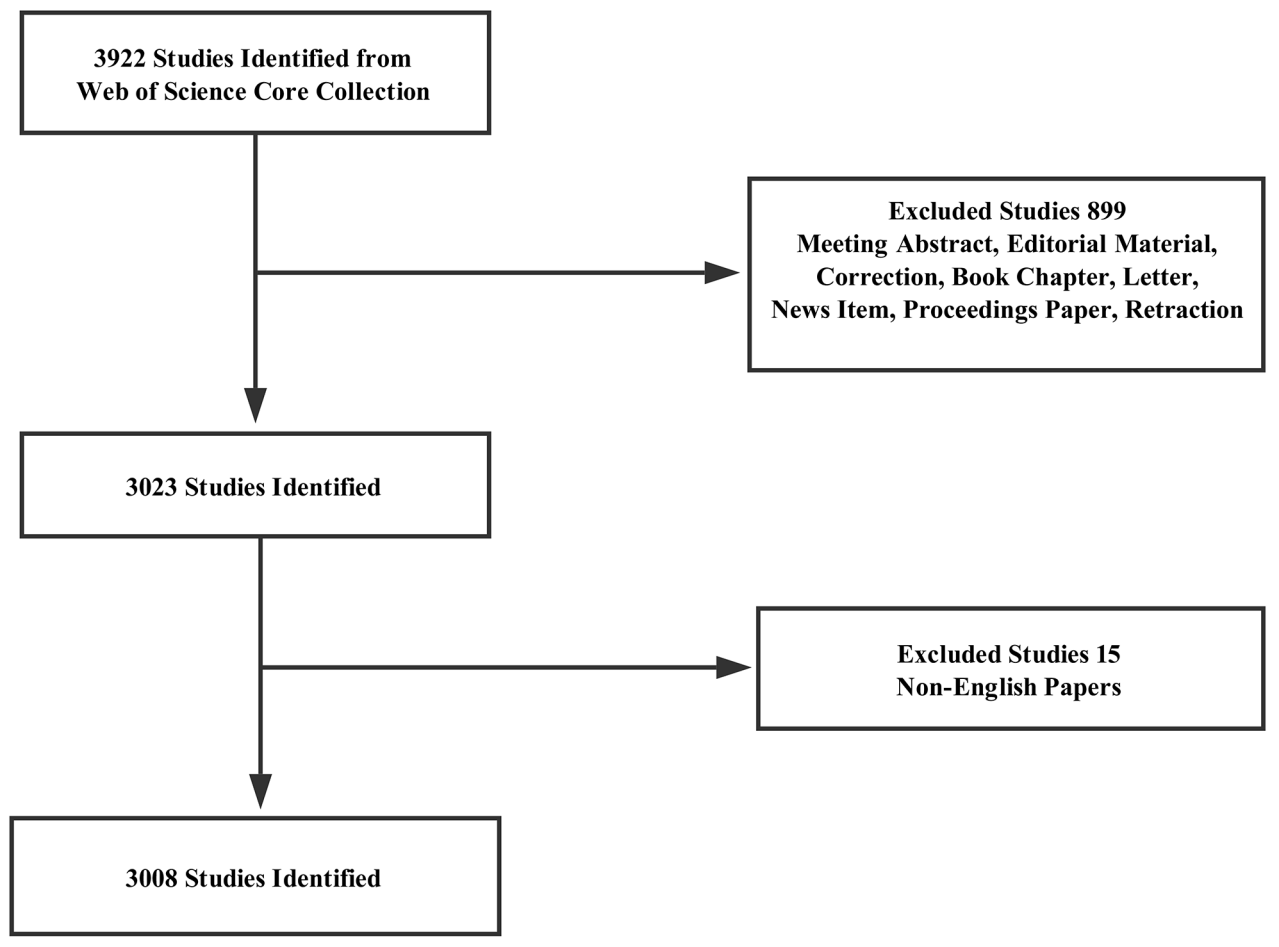
Flow chart of lncRNA studies inclusion

**Figure 2:**
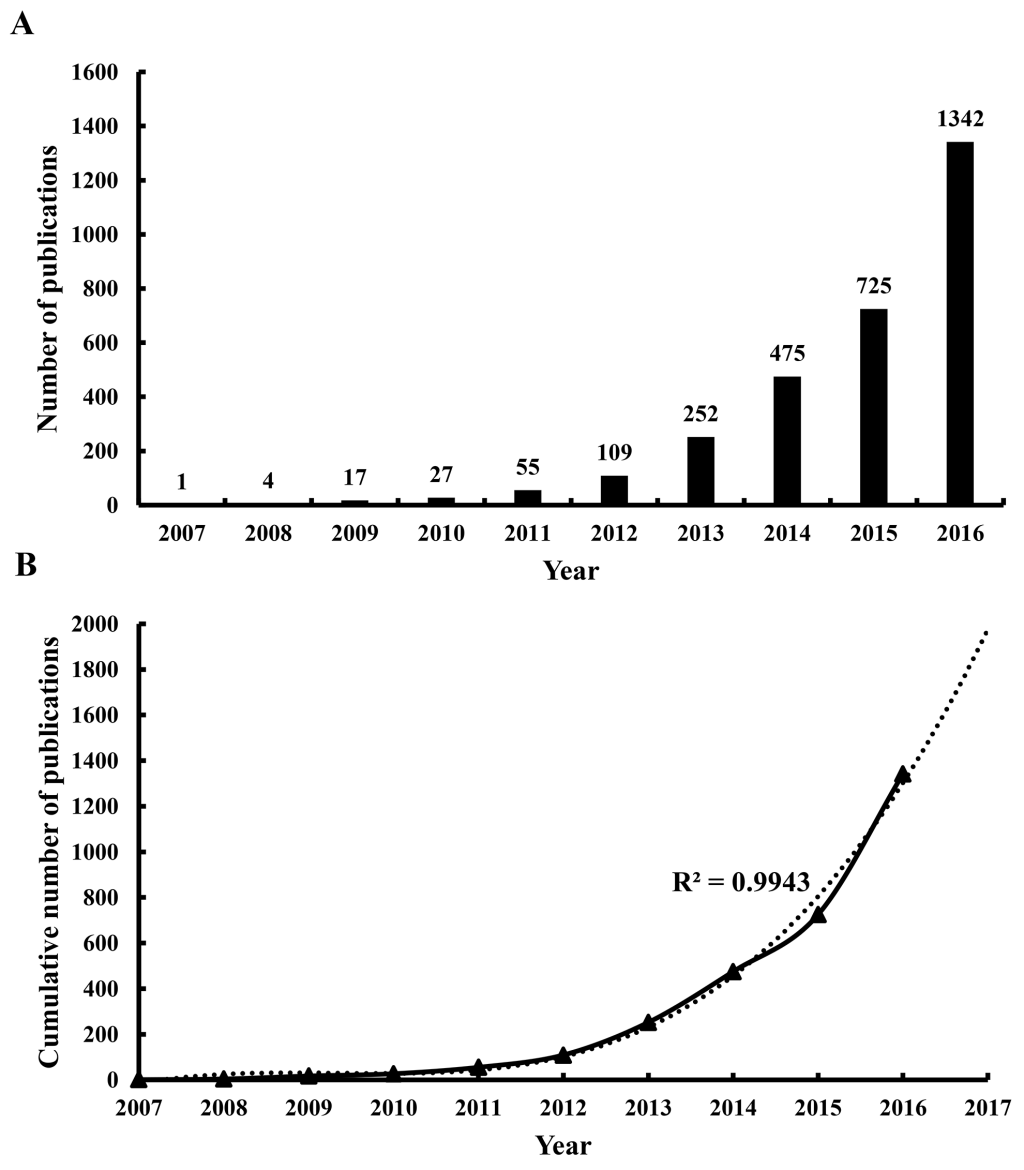
Publication outputs and growth prediction **(A)** The number of annual publications on lncRNA research from 2007 to 2016; **(B)** The model fitting curve of growth trend of lncRNA publications.

A total of 3,008 articles met the search criteria (Figure 1) (Supplementary Figure 1). The distribution of annual publications was shown in different time stage (Figure 2A). The overall trend of publication increased from one publication in 2007 to 1,342 publications in 2016.

The model fitting curve of lncRNA publication growth indicated a significant correlation (R^2^ = 0.9943) between the cumulative number of publications and publication year as shown in Figure 2B. By using cumulative publication numbers from 2007 to 2016, the number of publication was estimated to reach 1,976 in 2017.

### Distribution by journals

The 3,008 articles on lncRNA research were published in 663 academic journals (Supplementary Table 1). Among the top 15 journals (Table 1), *Oncotarget*, which impact factor (IF) 2016 is 5.168, contributed to the most publications on lncRNA research (203 publications, 6.75%), followed by *Tumor Biology* (IF2016, 3.650; 125 publications; 4.16%), *PLoS ONE* (IF2016, 2.806; 118 publications; 3.92%), and *Scientific Reports* (IF2016, 4.259; 97 publications; 3.23%).

**Table 1:** The top 15 journals that published articles on lncRNA research

Rank	Journal title	Country	Count	Percent	IF 2016
1	Oncotarget	United States	203	6.75%	5.168
2	Tumor Biology	Switzerland	125	4.16%	3.650
3	PLoS ONE	United States	118	3.92%	2.806
4	Scientific Reports	England	97	3.23%	4.259
5	International Journal of Clinical and Experimental Pathology	United States	94	3.13%	1.706
6	Nucleic Acids Research	England	46	1.53%	10.162
7	Biochemical and Biophysical Research Communications	United States	41	1.36%	2.466
8	Oncology Reports	Greece	38	1.26%	2.662
9	Molecular Medicine Reports	Greece	34	1.13%	1.692
10	BMC Genomics	England	33	1.10%	3.729
11	Biomed Research International	United States	33	1.10%	2.476
12	Molecular Cell	United States	32	1.06%	14.714
13	Biomedicine & Pharmacotherapy	France	32	1.06%	2.759
14	International Journal of Molecular Sciences	Switzerland	31	1.03%	3.226
15	Oncology Letters	Greece	30	1.00%	1.390

**Figure 3:**
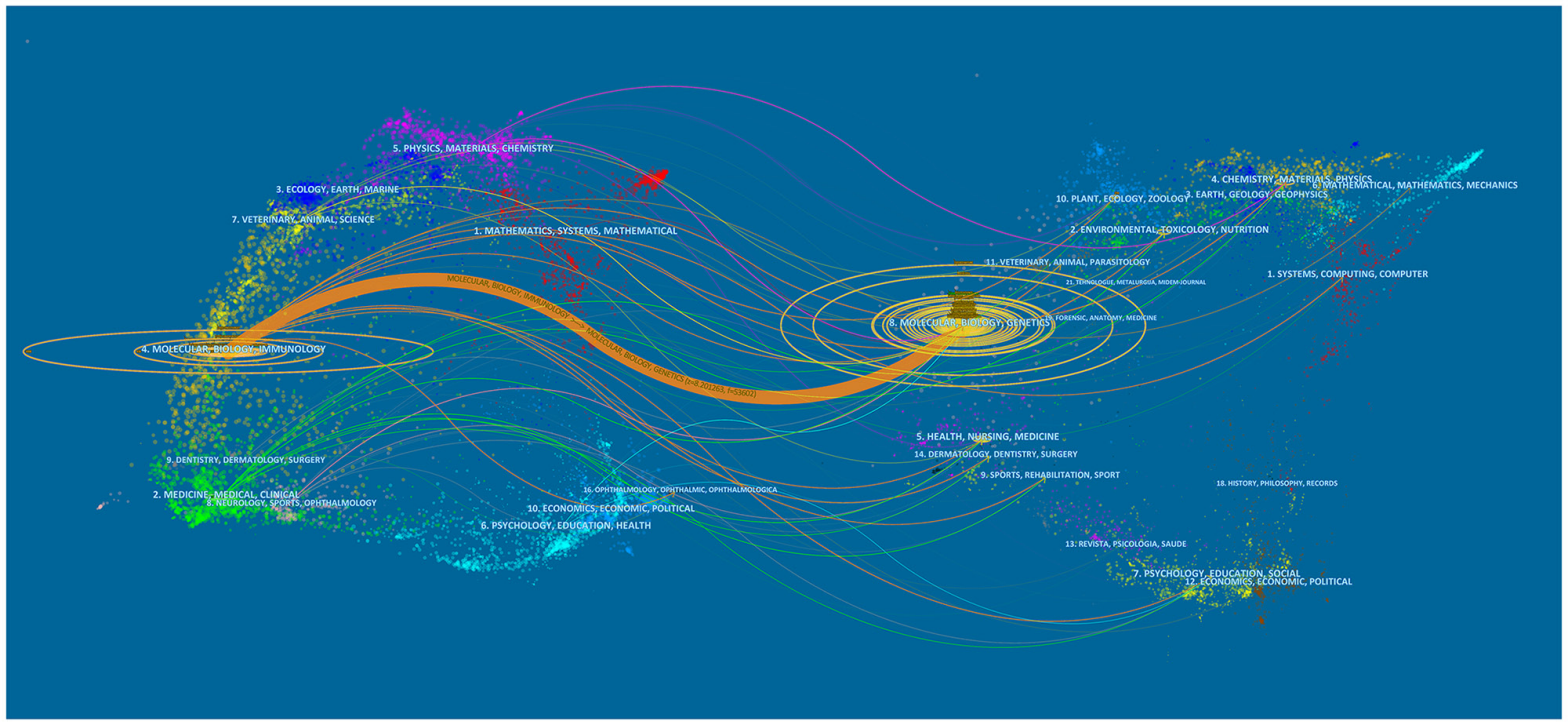
The dual-map overlay of journals related to lncRNA research

Figure 3 presented a dual-map overlay of journals. The left side was the citing journals map, and the right side was the cited journals map. The labels on the map showed the disciplines involved in journals. The lines were citation links starting from the left and point to the journals on the right. This dual-map overlay indicated that most articles were published in molecular journals, biology journals, and immunology journals, and they mainly cited journals from molecular, biology, and genetics areas.

### Distribution by countries and institutions

**Figure 4:**
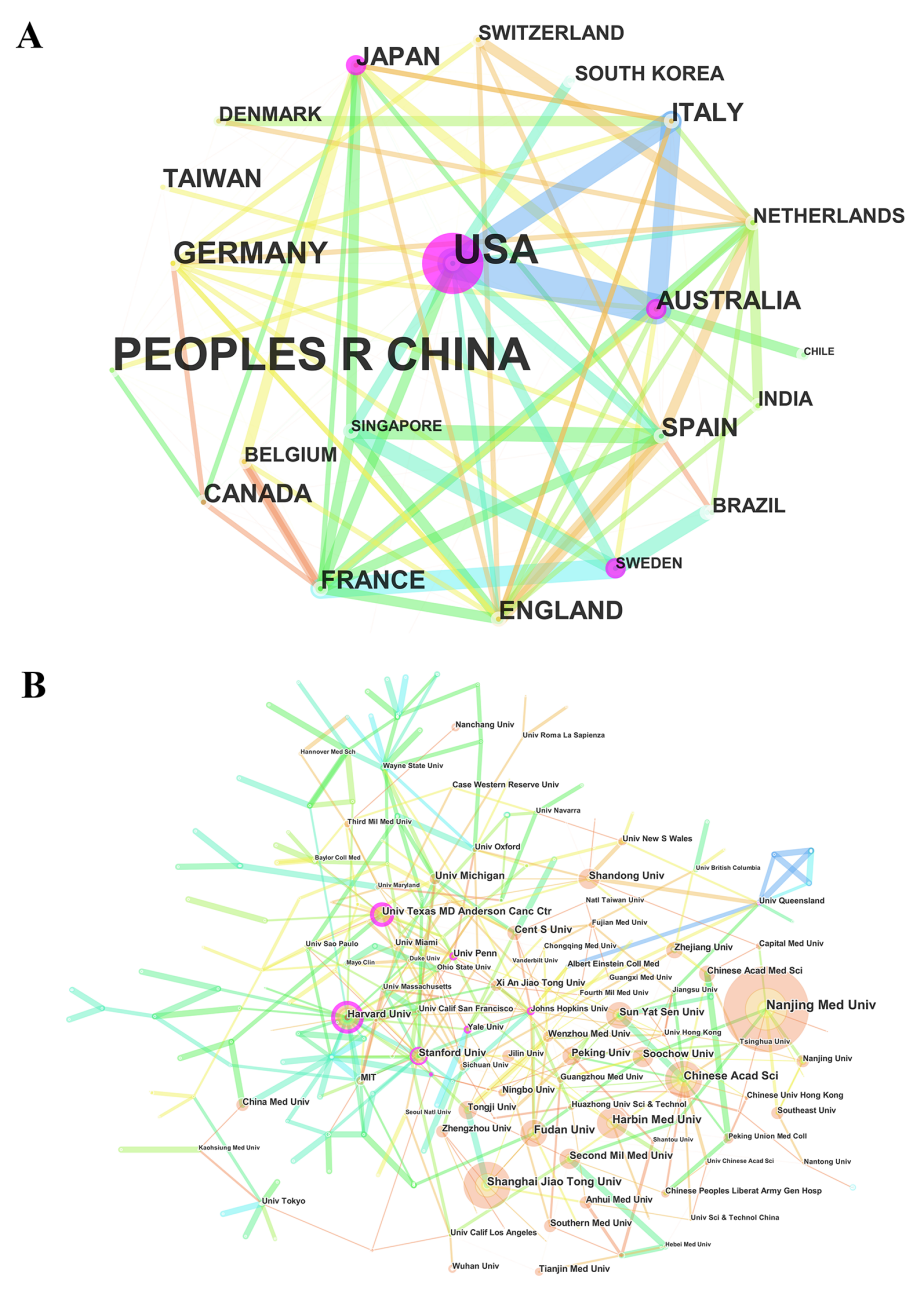
The analysis of countries and institutions **(A)** Network map of countries/territories engaged in lncRNA research; **(B)** Network map of institutions engaged in lncRNA research.

The 3,008 articles on lncRNA research were contributed by 57 countries/territories (Supplementary Table 2). There were extensive collaborations between countries/territories (Figure 4A). In relation to the top 10 countries that contributed to lncRNA research (Table 2), China had the largest number of publications (1843), followed by the United States (779), Germany (108), and England (100).

**Table 2:** The top 10 countries and institutions contributed to publications on lncRNA research

Rank	Country	Count	Institution	Count
1	China	1843	Nanjing Medical University	225
2	United States	779	Shanghai Jiao Tong University	125
3	Germany	108	Chinese Academy of Sciences	102
4	England	100	Harbin Medical University	89
5	Japan	97	Fudan University	80
6	Australia	88	Harvard University	78
7	Italy	78	Sun Yat-Sen University	75
8	Spain	57	Second Military Medical University	63
9	France	51	Shandong University	61
10	Canada	51	Soochow University	60

More than 2,100 institutions contributed to the publications on lncRNA research (Supplementary Table 3). Compared with countries, the cooperation between institutions was not significant (Figure 4B). The top 10 institutions contributed to 31.85% of the total number of publications. Nanjing Medical University led the first research echelon, followed by Shanghai Jiao Tong University, Chinese Academy of Sciences, and Harbin Medical University (Table 2).

### Analysis of citations, H-index, and ESI top papers

**Figure 5:**
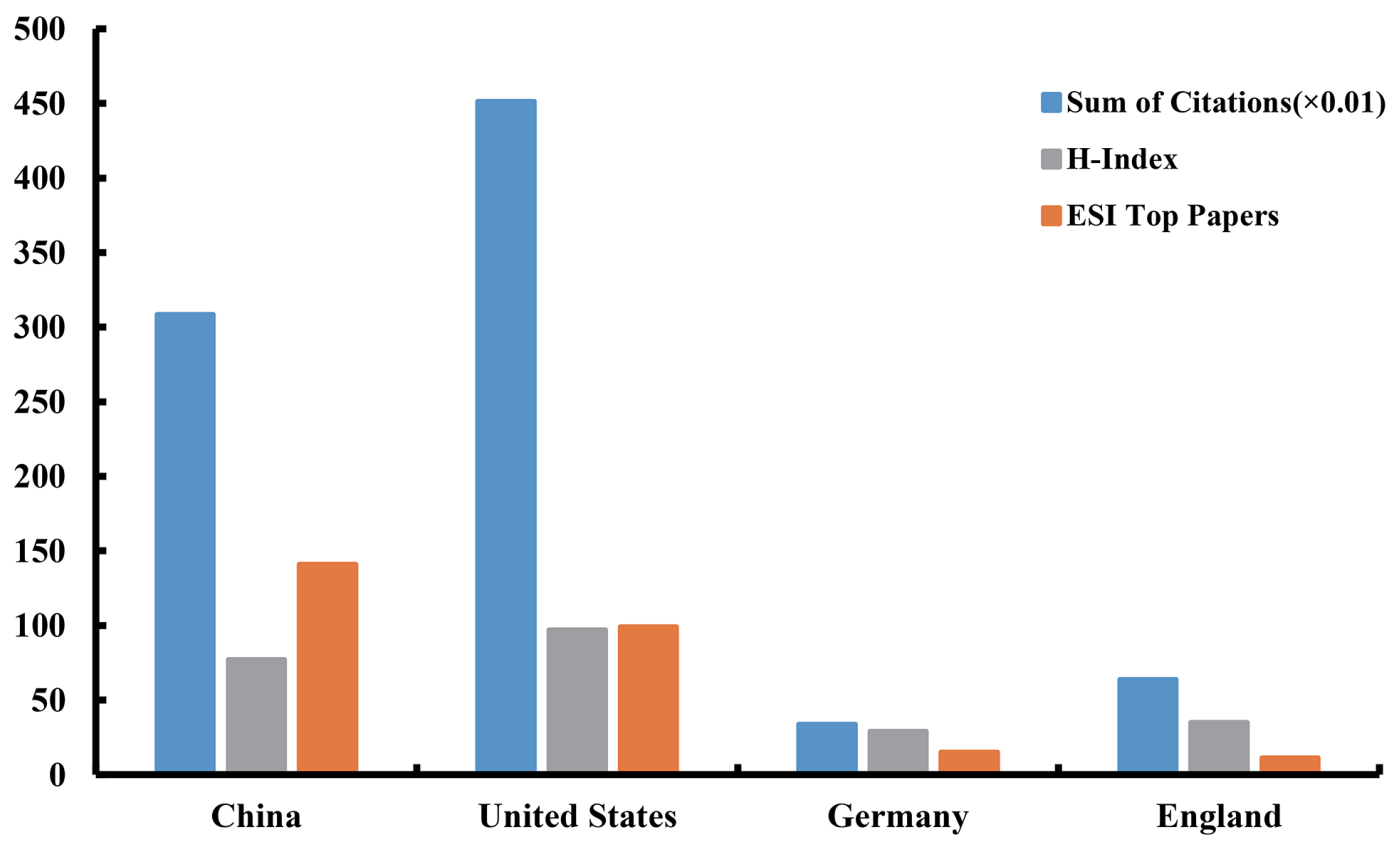
The citation counts (×0. 01), H-index, and ESI top papers in the top four countries

All articles related to lncRNA research had been cited 91,530 times since 2007. In top four countries (according to the number of publications), the United States had both the largest number of citations (45,120) and the highest value of H-index (97). Especially the citation counts, the United States accounted for 49.30% of the total citations. China had the largest number of ESI top papers (141). Due to the gap in the number of publications, Germany and England had no advantage in the ranking of these three items (Figure 5).

### Distribution by authors

**Figure 6:**
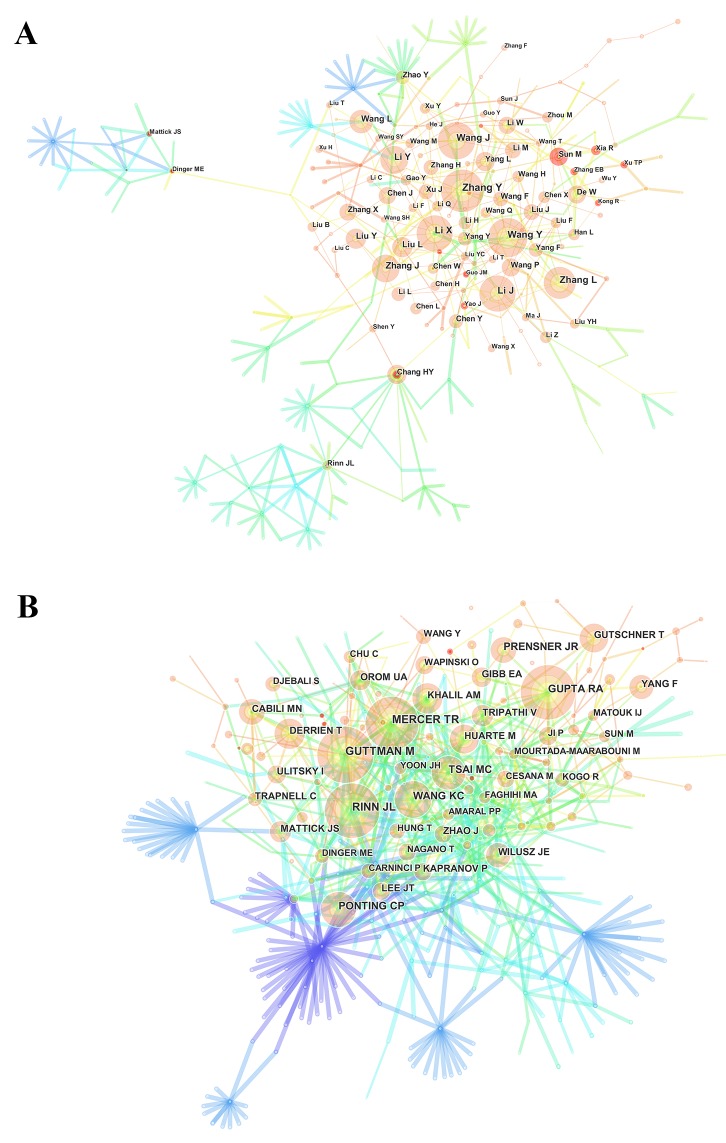
The analysis of authors **(A)** Network map of active authors contributed to lncRNA research; **(B)** Network map of co-cited authors contributed to lncRNA research.

Nearly 12,000 authors contributed to the total number of publications (Supplementary Table 4). The network map (Figure 6A) outlines the cooperation between authors. Regarding the authors who had the most publications (Table 3), Zhang Y ranked the first (72 publications), followed by Wang Y (67 publications), Wang J (63 publications), and Li J (60 publications).

**Table 3:** The top 10 authors, co-cited authors, and co-cited references in lncRNA research

Rank	Author	Count	Co-cited Author	Count	Co-cited Reference	Count
1	Zhang Y	72	Guttman M	1556	Gupta RA, 2010, Nature, V464, P1071	930
2	Wang Y	67	Mercer TR	1213	Derrien T, 2012, Genome Research, V22, P1775	543
3	Wang J	63	Rinn JL	1147	Tsai MC, 2010, Science, V329, P689	535
4	Li J	60	Gupta RA	932	Guttman M, 2009, Nature, V458, P223	477
5	Li X	58	Wang KC	753	Cabili MN, 2011, Genes & Development, V25, P1915	461
6	Zhang L	56	Ponting CP	708	Wang KC, 2011, Molecular Cell, V43, P904	450
7	Zhang J	49	Tsai MC	701	Rinn JL, 2012, Annual Review of Biochemistry, V81, P145	427
8	Li Y	47	Prensner JR	683	Mercer TR, 2009, Nature Review Genetics, V10, P155	423
9	Wang L	41	Gutschner T	652	Huarte M, 2010, Cell, V142, P409	422
10	Liu Y	37	Khalil AM	622	Khalil AM, 2009, Proceedings of the National Academy of Sciences, V106, P11667	409

CiteSpace IV mined the information on authors citation and presented it as a network map (Figure 6B). In relation to the top 10 co-cited authors (Table 3) (Supplementary Figure 2), Guttman M (1556 citations), led, followed by Mercer TR (1213 citations), Rinn JL (1147 citations), and Gupta RA (932 citations).

### Analysis of references

**Figure 7:**
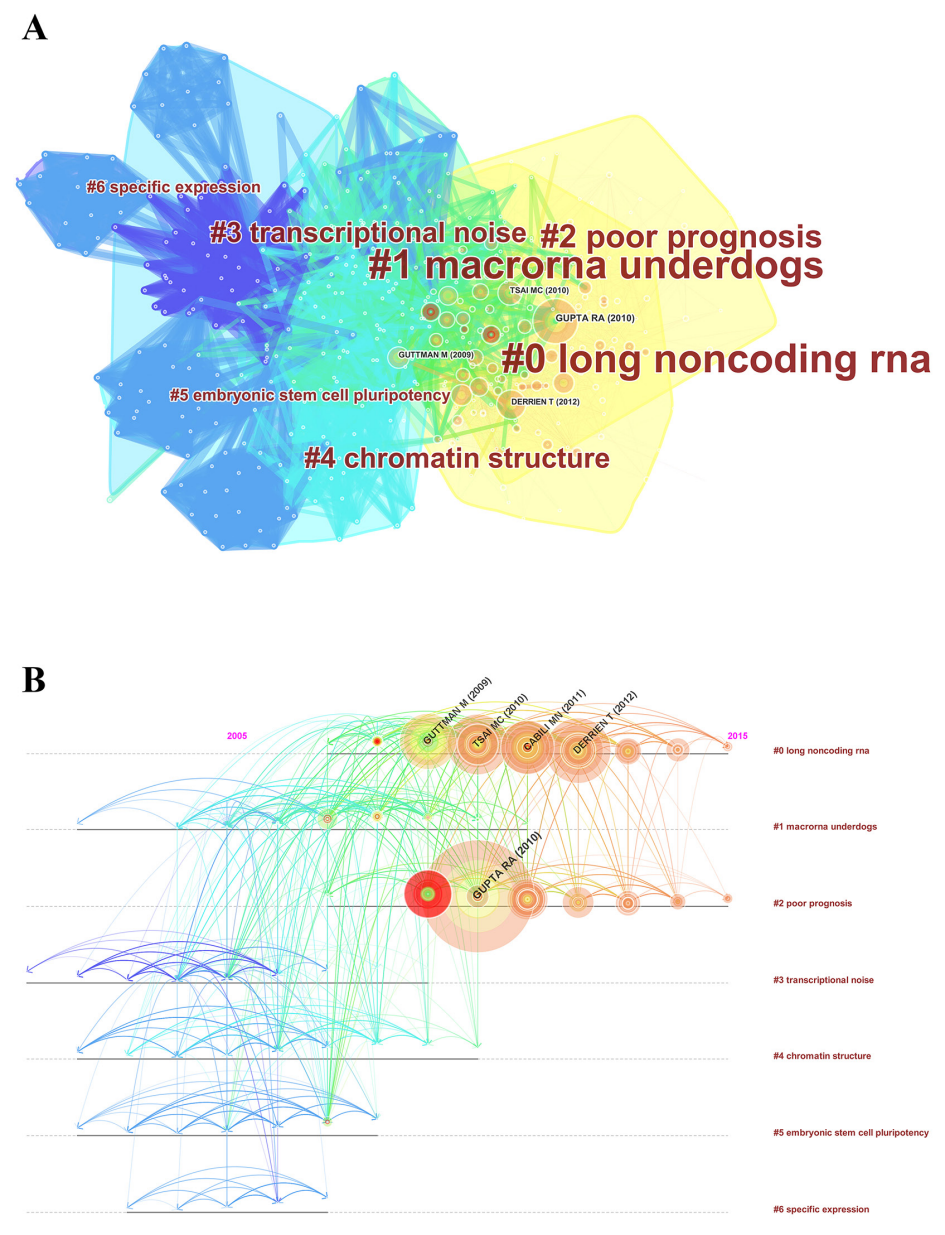
The analysis of references **(A)** Co-citation map of references from publications on lncRNA research; **(B)** Co-citation map (timeline view) of references from publications on lncRNA research.

Reference analysis is one of the most significant indicators in bibliometrics. The co-citation map of references suggests the scientific relevance of the publications (Figure 7A). Here, the modularity Q score was higher than 0.5 (0.5096) (Supplementary Figure 3), which indicates the network was reasonably divided into loosely coupled clusters. The average silhouette score was greater than 0.5 (0.6383) (Supplementary Figure 3), which means the homogeneity of these clusters was acceptable on average. All clusters were labeled with index terms extracted from the references (Supplementary Figure 4). The largest cluster #0 was labeled as “long noncoding RNA,” followed by the second largest cluster #1, labeled as “macroRNA underdogs,” and the third largest cluster #2, labeled as “poor prognosis.” These clusters mentioned above were also presented in a timeline view (Figure 7B).

### Analysis of keywords

**Figure 8:**
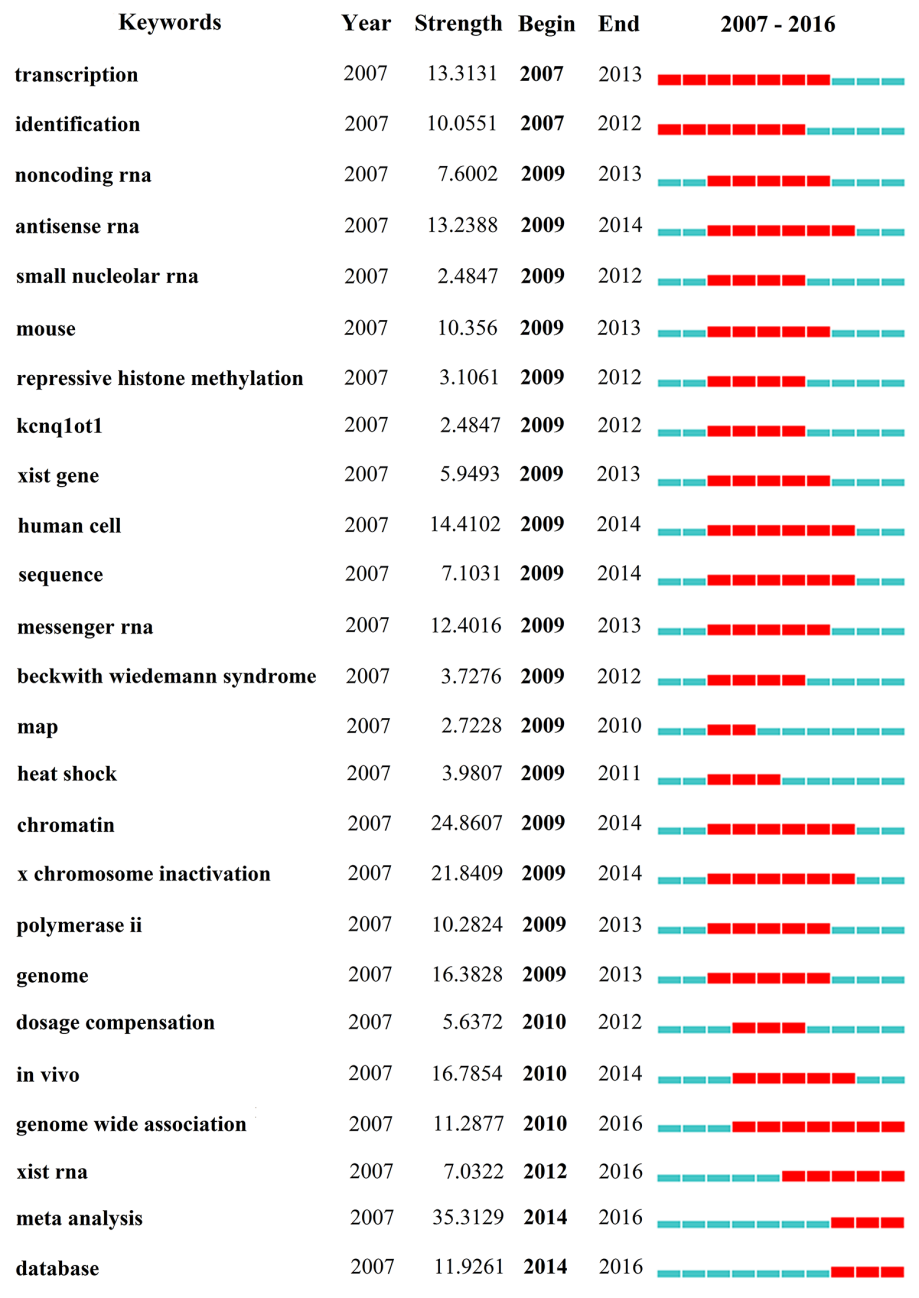
The keywords with the strongest citation bursts of publications on lncRNA research

CiteSpace IV extracted keywords that occurred in 3,008 publications. We used CiteSpace IV to detect and analyze keywords with the strongest citation bursts (Figure 8) (Supplementary Figure 5). The keywords that had the strongest citation bursts after 2010 are as follows: “dosage compensation” (2010-2012), “*in vivo*” (2010-2014), “genome-wide association” (2010-2016), “xist RNA” (2012-2016), “meta-analysis” (2014-2016), and “database” (2014-2016).

## DISCUSSION

### General data

According to the number of publications, the publication year can be divided into two phases. The first phase (2007-2011) could be considered as the initial stage of lncRNA research. Thus, the number of publications increased slowly during this period. With increase in the intensity of research, more findings will emerge. In the second phase (2012-2016), there was a sharp growth of publications related to lncRNA research. Thus, this stage could be considered as the golden period of development for lncRNA research. Moreover, the prediction curve indicated that there might be more publications in this field in the following years. The development prospects of lncRNA research could be expected.

Regarding the top 15 journals, 2 of the journals, including *Nucleic Acids Research* (IF2016, 10.162) and *Molecular Cell* (IF2016, 14.714) had an impact factor (IF) greater than 10.000; 1 of the journals, including *Oncotarget* (IF2016, 5.168) had an IF between 5.000 and 10.000; 4 of the journals, including *Tumor Biology* (IF2016, 3.650), *Scientific Reports* (IF2016, 4.259), *BMC Genomics* (IF2016, 3,729), and *International Journal of Molecular Sciences* (IF2016, 3.226) had an IF between 3.000 and 5.000. Moreover, the journals with high IF (greater than 3.000) contributed to 18.86% (IF >10.000, 2.59%; 10.000 >IF >5.000, 6.75%; 5.000 >IF >3.000, 9.52%) of the total number of publications. In summary, it was challenging of publishing papers related to lncRNA research in high-IF journals.

In the list of top 10 countries (5 European countries, 2 American countries, and 3 Asia-Pacific countries), China was the only developing country, contributed to more than 60% of the total number of publications, indicating that it has made significant progress in this field. Although China had a huge advantage in the number of publications, the United States showed its dominant position in both citation frequency and H-index. Therefore, from the perspective of research quality, the United States was the leading country in this field. Regarding the collaboration network, there was a broad range of cooperation between Western nations. The strongest collaborations were identified among the United States, Australia, and Italy, between France and Sweden, and between Spain and Singapore.

In the list of top 10 institutions, except Harvard University, the remaining 9 institutions were all from China. Moreover, Chinese institutions accounted for the largest proportion in the collaboration network. That is the reason why China contributed to the most number of publications related to lncRNA research.

### Citation data

According to the top 10 authors identified in this analysis, each contributed to no fewer than 35 papers. Therefore, they were identified as “prolific authors.” However, none of these prolific authors were included in the list of top 10 co-cited authors, with regard to annual co-citation counts, suggesting that prolific authors should consider more about their quality of papers while working to increase their number of papers. For co-cited authors, the authors who had at least 1,000 co-citation counts, include Guttman M, who provided an emerging model that identified modular regulatory principles of lncRNAs [16]; Mercer TR, who reported the structure and function of lncRNAs in epigenetic regulation [17]; and Rinn JL, who explored the genome regulation by lncRNAs [18]. Although none of these authors belonged to prolific authors, they made crucial contributions to lncRNA research.

For co-cited references, the map of co-citation clusters in the timeline view indicated that the most influential references were concentrated in the period from 2009 to 2012. The top 10 co-cited references were shown in Table 3, and they were regarded as the intellectual bases in lncRNA research. Gupta RA (2010), who published in *Nature*, had the highest co-citation counts (930), followed by Derrien T (2012, 543 co-citation counts), Tsai MC (2010, 535 co-citation counts), and Guttman M (2009, 477 co-citation counts), who published in *Genome Research*, *Science*, and *Nature* respectively. Furthermore, *Genes & Development*, *Molecular Cell*, *Annual Review of Biochemistry*, *Nature Review Genetics*, and *Proceedings of the National Academy of Sciences* also published some highly influential papers. These journals were fundamental in this field.

### Research frontiers

Keywords with bursts (abrupt changes or emerging trends) provide a reasonable prediction of research frontiers [19]. In this instance, CiteSpace IV was used to capture the keywords with the strongest citation bursts that identified as research frontiers over time. The time intervals were plotted on the blue line, while the periods of burst keywords were plotted on the red line, indicating the beginning and end of the time interval of each burst [20]. The top four research frontiers of lncRNA research were listed as follows:

i. Database: Many bioinformatics studies on lncRNAs have been conducted in recent years. The lncRNA profiles in these studies were mainly obtained from two databases: The Cancer Genome Atlas (TCGA) database and Gene Expression Omnibus (GEO) database. The lncRNA sequence extracted from TCGA or GEO were analyzed via bioinformatics methods, and lncRNA-expression signatures were identified as potential prognostic biomarkers for related cancer [21-24]. Moreover, the association between lncRNA expression and epigenetic regulation can also be revealed through bioinformatics analysis [25-27]. Apart from the two databases mentioned above, there are some specialized databases for lncRNA, including PLncDB [28], lncRNASNP [29], LncReg [30], and LNCCipedia [31], and so forth. These databases provide comprehensive data for lncRNA bioinformatics research.

ii. Meta-analysis: Many of the meta-analysis papers related to lncRNA research have been published in recent years, including some that were high-quality [32-34].

iii. Xist RNA: Xist RNA is a long noncoding RNA, which orchestrates X chromosome inactivation, a process that entails chromosome silencing and remodeling the three-dimensional structure of the X chromosome [35]. However, this argument remains controversial. A recent study found that Xist-mediated silencing required a direct interaction between Xist RNA and Lamin B receptor (an integral part of the nuclear lamina) [36]. The results indicated that Xist-mediated silencing needs lamina recruitment [36]. Apart from this, some studies have focused on the role and molecular mechanisms of Xist RNA in disease progression [37-39].

iv. Genome-wide association study: The genome-wide association study (GWAS) is an examination, where whole-gene variants in different individuals were examined, to evaluate the association of any variant with a trait [40]. GWAS involves the association between single-nucleotide polymorphisms (SNPs) and traits such as major human diseases [41]. In lncRNA research, GWAS has been used to reveal the different patterns of epigenetic features in lncRNA loci [42] and to identify susceptible lncRNAs as a disease-related risk factor [43-46].

### Strengths and limitations

Data on lncRNA publications were retrieved and collected from Web of Science Core Collection (WoSCC) database (Science Citation Index-Expanded journals). The analysis of data was relatively comprehensive and objective. However, the most of the publications in the WoSCC database were in English. Publication in non-English were very few. Other databases (e.g. Ovid, PubMed, Scopus, and Google Scholar) were not analyzed since WoSCC is more advanced at providing detailed data (e.g. annual publications, journal sources, author information, country and institution information). Secondly, the results of bibliometric analysis and the results of actual research conditions were still different, as some recent publications do not have a high citation count. Lastly, although all searches were retrieved on June 17, 2017, to avoid bias due to the update of WoSCC database for the year 2016, the database is still receiving new data. Despite this, we think this paper includes the vast majority of publications from 2016, and the small amount of new data may not change the conclusion.

## CONCLUSION

This study helps investigators master the trends of lncRNA research. The top three journals that contributed to the largest number of publications were *Oncotarget*, *Tumor Biology*, and *PLoS ONE*. China, United States, and Germany were the top three countries engaged in lncRNA research. The strongest cooperation was observed between developed countries, particularly, the United States was in the dominant position. There were many Chinese institutions engaged in lncRNA research, but significant collaborations among them were not noticed. Guttman M (Broad Institute, United States), Mercer TR (University of Queensland, Australia), Rinn JL (Harvard University, United States), and Gupta RA (Stanford University, United States) may be good candidates for research collaboration in this field. “Database,” “Xist RNA,” and “Genome-wide association study” may be the latest research frontiers, and related studies may pioneer this field in the next few years.

## MATERIALS AND METHODS

### Source of the data and search strategy

Literature was searched from the Science Citation Index-Expanded (SCI-E) of the Web of Science Core Collection (WoSCC) of Clarivate Analytics on June 17, 2017. The data were extracted from the public database. Ethical approval was not applicable in this case.

The following terms were used: TI= (“lncRNA*”) TI= (“lnc RNA*”) OR TI= (“long ncRNA*”) OR TI= (“long non translated RNA*”) OR TI= (“long non coding RNA*”) OR TI= (“long non protein coding RNA*”) OR TI= (“long noncoding RNA*”) OR TI= (“long untranslated RNA*”) OR TI= (“long intergenic non protein coding RNA*”) OR TI= (“large intergenic non coding RNA*”) OR TI= (“large intergenic noncoding RNA*”) OR TI= (“lincRNA*”) OR TI= (“linc RNA*”) AND Language= English. In this case, only articles and reviews were included.

### Data collection

All data were independently collected by two authors (Yan Miao and Si-Yi Xu) and downloaded in TXT format. The data were imported into CiteSpace IV (Drexel University, Philadelphia, United States) and Microsoft Excel 2016 (Redmond, Washington, United States), and qualitatively and quantitatively analyzed.

### Statistical methods

WoSCC analyzed the characteristics of publications, including annual publications, journal sources, countries or territories, institutions, authors, citation counts, ESI top papers, and H-index.

Microsoft Excel 2016 was used to analyze the time trend of publications. The model: *f(x) = ax*^*3*^ *+ bx*^*2*^ *+ cx + d* was used to predict future trend of papers in this field, based on the cumulative number of publications. Symbol *x* represented the publication year, and *f(x)* was the cumulative number of publications by the year.

CiteSpace IV was used to (i) capture the relationship between citing journals and cited journals, (ii) identify the collaborations between countries/institutions/authors, (iii) perform co-citation analysis on authors and references, (iv) perform a citation-burst analysis of keywords, and (v) generate visualizations of all the items mentioned above.

## SUPPLEMENTARY MATERIALS FIGURES AND TABLES











## Abbreviations

lncRNA: long noncoding RNA
WoSCC: Web of Science Core Collection
SCI-E: Science Citation Index-Expanded
IF: impact factor
TCGA: The Cancer Genome Atlas
GEO: Gene Expression Omnibus
GWAS: Genome-wide association study
SNPs: single-nucleotide polymorphisms
