# Supplementary material for: Trends of long noncoding RNA research from 2007 to 2016: a bibliometric analysis
Source: Oncotarget. 2017 Sep 12;8(47):83114–27. doi: 10.18632/oncotarget.20851 (PMC5669954; doi:10.18632/oncotarget.20851)
Supplement: Supplementary file 1 [file oncotarget-08-83114-s001.pdf]

Trends of long noncoding RNA research from 2007 to 2016: a bibliometric analysis

SUPPLEMENTARY MATERIALS

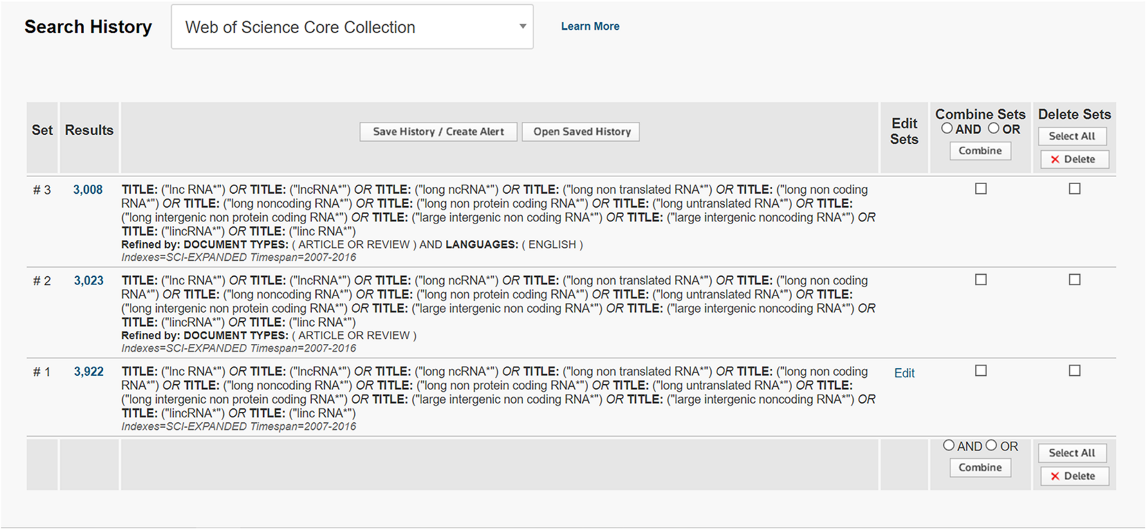

Supplementary Figure 1: Raw data on search history of Web of Science Core Collection.

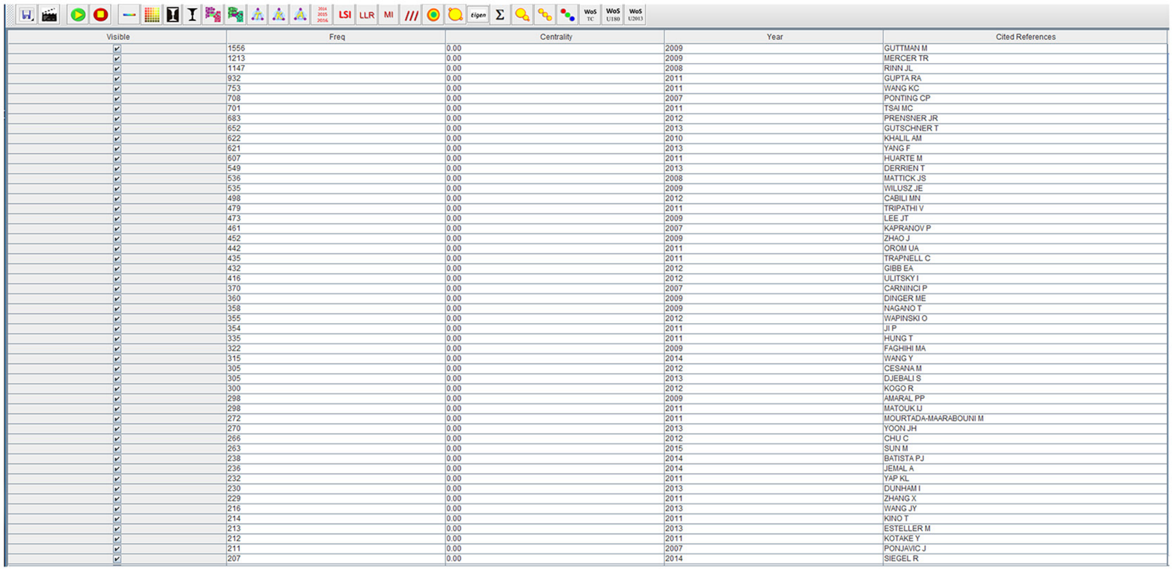

Visible	Freq	Centrality	Year	Cited References
✔	1556	0.00	2009	GUTTMAN M
✔	1213	0.00	2009	MERCER TR
✔	1147	0.00	2008	RINN JL
✔	932	0.00	2011	GUPTA RA
✔	753	0.00	2011	WANG KC
✔	708	0.00	2007	PONTING CP
✔	701	0.00	2011	TSAI MC
✔	683	0.00	2012	PRENSNER JR
✔	652	0.00	2013	GUTSCHNER T
✔	622	0.00	2010	KHALIL AM
✔	621	0.00	2013	YANG F
✔	607	0.00	2011	HUARTE M
✔	549	0.00	2013	DERRIEN T
✔	536	0.00	2008	MATTICK JS
✔	535	0.00	2009	WILUSZ JE
✔	498	0.00	2012	CABILI MN
✔	479	0.00	2011	TRIPATHI V
✔	473	0.00	2009	LEE JT
✔	461	0.00	2007	KAPRANOV P
✔	452	0.00	2009	ZHAO J
✔	442	0.00	2011	OROM UA
✔	435	0.00	2011	TRAPNELL C
✔	432	0.00	2012	GIBB EA
✔	416	0.00	2012	ULITSKY I
✔	370	0.00	2007	CARNINCI P
✔	360	0.00	2009	DINGER ME
✔	358	0.00	2009	NAGANO T
✔	355	0.00	2012	WAPINSKI O
✔	354	0.00	2011	JI P
✔	335	0.00	2011	HUNG T
✔	322	0.00	2009	FAGHIHI MA
✔	315	0.00	2014	WANG Y
✔	305	0.00	2012	CESANA M
✔	305	0.00	2013	DJEBALI S
✔	300	0.00	2012	KOGO R
✔	298	0.00	2009	AMARAL PP
✔	298	0.00	2011	MATOUK IJ
✔	272	0.00	2011	MOURTADA-MAARABOUNI M
✔	270	0.00	2013	YOON JH
✔	266	0.00	2012	CHU C
✔	263	0.00	2015	SUN M
✔	238	0.00	2014	BATISTA PJ
✔	236	0.00	2014	JEMAL A
✔	232	0.00	2011	YAP KL
✔	230	0.00	2013	DUNHAM I
✔	229	0.00	2011	ZHANG X
✔	216	0.00	2013	WANG JY
✔	214	0.00	2011	KINO T
✔	213	0.00	2013	ESTELLER M
✔	212	0.00	2011	KOTAKE Y
✔	211	0.00	2007	PONJAVIC J
✔	207	0.00	2014	SIEGEL R

Supplementary Figure 2: Raw data on co-cited authors that analyzed by CiteSpace IV.

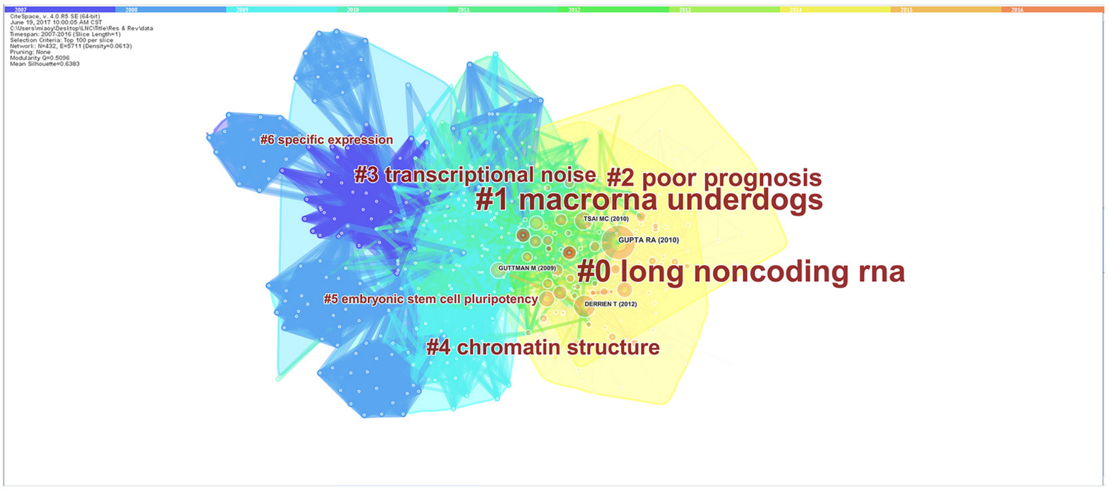

Supplementary Figure 3: Raw data on co-citation map of references that analyzed by CiteSpace IV.

Visible	Freq	Centrality	Year	Cited References
✓	930	0.00	2010	GUPTA RA, 2010, NATURE, V464, P1071, DOI 10.1038/NATURE08975
✓	543	0.00	2012	DERRIEN T, 2012, GENOME RES, V22, P1775, DOI 10.1101/GR.132159.111
✓	535	0.00	2010	TSAI MC, 2010, SCIENCE, V329, P689, DOI 10.1126/SCIENCE.1192002
✓	477	0.00	2009	GUTTMAN M, 2009, NATURE, V458, P223, DOI 10.1038/NATURE07672
✓	461	0.00	2011	CABILI MN, 2011, GENE DEV, V25, P1915, DOI 10.1101/GAD.17446611
✓	450	0.00	2011	WANG KC, 2011, MOL CELL, V43, P904, DOI 10.1016/J.MOLCEL.2011.08.018
✓	427	0.00	2012	RINN JL, 2012, ANNU REV BIOCHEM, V81, P145, DOI 10.1146/ANNUREV-BIOC...
✓	423	0.00	2009	MERCER TR, 2009, NAT REV GENET, V10, P155, DOI 10.1038/NRG2521
✓	422	0.00	2010	HUARTE M, 2010, CELL, V142, P409, DOI 10.1016/J.CELL.2010.06.040
✓	409	0.00	2009	KHALIL AM, 2009, P NATL ACAD SCI USA, V106, P11667, DOI 10.1073/PNAS.09...
✓	391	0.00	2009	PONTING CP, 2009, CELL, V136, P629, DOI 10.1016/J.CELL.2009.02.006
✓	374	0.00	2011	GUTTMAN M, 2011, NATURE, V477, P295, DOI 10.1038/NATURE10398
✓	364	0.00	2010	OROM UA, 2010, CELL, V143, P46, DOI 10.1016/J.CELL.2010.09.001
✓	354	0.00	2011	WAPINSKI O, 2011, TRENDS CELL BIOL, V21, P354, DOI 10.1016/J.TCB.2011.0...
✓	347	0.00	2010	TRIPATHI V, 2010, MOL CELL, V39, P925, DOI 10.1016/J.MOLCEL.2010.08.011
✓	313	0.00	2011	GIBB EA, 2011, MOL CANCER, V10, P, DOI 10.1186/1476-4598-10-38
✓	300	0.00	2011	CESANA M, 2011, CELL, V147, P358, DOI 10.1016/J.CELL.2011.09.028
✓	298	0.00	2011	WANG KC, 2011, NATURE, V472, P120, DOI 10.1038/NATURE09819
✓	297	0.00	2011	KOGO R, 2011, CANCER RES, V71, P6320, DOI 10.1158/0008-5472.CAN-11-1021
✓	296	0.00	2012	DJEBALI S, 2012, NATURE, V489, P101, DOI 10.1038/NATURE11233
✓	273	0.00	2012	GUTSCHNER T, 2012, RNA BIOL, V9, P703, DOI 10.4161/RNA.20481
✓	262	0.00	2011	PRENSNER JR, 2011, CANCER DISCOV, V1, P391, DOI 10.1158/2159-8290.CD...
✓	259	0.00	2012	GUTTMAN M, 2012, NATURE, V482, P339, DOI 10.1038/NATURE10887
✓	235	0.00	2009	WILUSZ JE, 2009, GENE DEV, V23, P1494, DOI 10.1101/GAD.1800909
✓	232	0.00	2010	YAP KL, 2010, MOL CELL, V38, P662, DOI 10.1016/J.MOLCEL.2010.03.021
✓	229	0.00	2013	BATISTA PJ, 2013, CELL, V152, P1298, DOI 10.1016/J.CELL.2013.02.012
✓	228	0.00	2013	GUTSCHNER T, 2013, CANCER RES, V73, P1180, DOI 10.1158/0008-5472.CAN...
✓	228	0.00	2012	DUNHAM I, 2012, NATURE, V489, P57, DOI 10.1038/NATURE11247
✓	222	0.00	2011	HUNG T, 2011, NAT GENET, V43, P621, DOI 10.1038/NG.848
✓	213	0.00	2013	ULITSKY I, 2013, CELL, V154, P26, DOI 10.1016/J.CELL.2013.06.020
✓	208	0.00	2007	RINN JL, 2007, CELL, V129, P1311, DOI 10.1016/J.CELL.2007.05.022
✓	204	0.00	2014	FATICA A, 2014, NAT REV GENET, V15, P7, DOI 10.1038/NRG3606
✓	202	0.00	2011	PRENSNER JR, 2011, NAT BIOTECHNOL, V29, P742, DOI 10.1038/NBT.1914
✓	199	0.00	2010	KINO T, 2010, SCI SIGNAL, V3, P, DOI 10.1126/SCISIGNAL.2000568
✓	198	0.00	2011	ESTELLER M, 2011, NAT REV GENET, V12, P861, DOI 10.1038/NRG3074
✓	197	0.00	2011	KOTAKE Y, 2011, ONCOGENE, V30, P1956, DOI 10.1038/ONC.2010.568
✓	195	0.00	2011	CHU C, 2011, MOL CELL, V44, P667, DOI 10.1016/J.MOLCEL.2011.08.027
✓	194	0.00	2010	WANG JY, 2010, NUCLEIC ACIDS RES, V38, P5366, DOI 10.1093/NAR/GKQ285
✓	189	0.00	2010	POLISENO L, 2010, NATURE, V465, P1033, DOI 10.1038/NATURE09144
✓	187	0.00	2011	ULITSKY I, 2011, CELL, V147, P1537, DOI 10.1016/J.CELL.2011.11.055
✓	186	0.00	2011	SALMENA L, 2011, CELL, V146, P353, DOI 10.1016/J.CELL.2011.07.014
✓	184	0.00	2011	YANG Z, 2011, ANN SURG ONCOL, V18, P1243, DOI 10.1245/S10434-011-1581-Y
✓	178	0.00	2010	GUTTMAN M, 2010, NAT BIOTECHNOL, V28, P503, DOI 10.1038/NBT.1633
✓	178	0.00	2010	LOEWER S, 2010, NAT GENET, V42, P1113, DOI 10.1038/NG.710
✓	177	0.00	2011	GONG CG, 2011, NATURE, V470, P284, DOI 10.1038/NATURE09701
✓	174	0.00	2010	TRAPNELL C, 2010, NAT BIOTECHNOL, V28, P511, DOI 10.1038/NBT.1621
✓	170	0.00	2012	LEE JT, 2012, SCIENCE, V338, P1435, DOI 10.1126/SCIENCE.1231776
✓	165	0.00	2013	KIM K, 2013, ONCOGENE, V32, P1616, DOI 10.1038/ONC.2012.193
✓	164	0.00	2011	YANG F, 2011, HEPATOLOGY, V54, P1679, DOI 10.1002/HEP.24563
✓	163	0.00	2011	JEMAL A, 2011, CA-CANCER J CLIN, V61, P69, DOI 10.3322/CAAC.20107
✓	162	0.00	2011	TSAI MC, 2011, CANCER RES, V71, P3, DOI 10.1158/0008-5472.CAN-10-2483
✓	159	0.00	2012	SPIZZO R, 2012, ONCOGENE, V31, P4577, DOI 10.1038/ONC.2011.621

Supplementary Figure 4: Raw data on co-cited references that analyzed by CiteSpace IV.

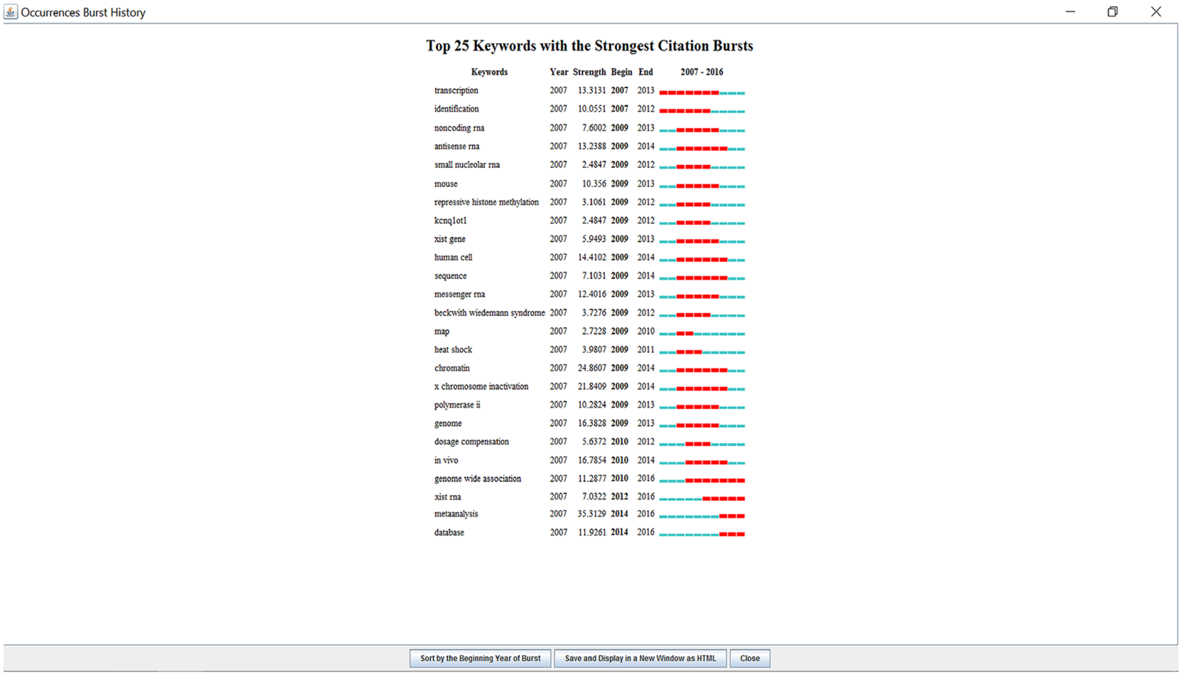

Supplementary Figure 5: Raw data on citation-burst keywords that analyzed by CiteSpace IV.

Supplementary Table 1: Raw data on journal sources of lncRNA publications extracted from the Web of Science Core Collection.

See Supplementary File 1

Supplementary Table 2: Raw data on countries/territories involved in lncRNA publications extracted from the Web of Science Core Collection.

See Supplementary File 2

Supplementary Table 3: Raw data on institutions involved in lncRNA publications extracted from the Web of Science Core Collection.

See Supplementary File 3

Supplementary Table 4: Raw data on authors involved in lncRNA publications extracted from Web of Science Core Collection.

See Supplementary File 4
