# Supplementary material for: Trends of long noncoding RNA research from 2007 to 2016: a bibliometric analysis
Source: Oncotarget. 2017 Sep 12;8(47):83114–27. doi: 10.18632/oncotarget.20851 (PMC5669954; doi:10.18632/oncotarget.20851)
Supplement: Supplementary file 2 [file oncotarget-08-83114-s002.docx]

**Supplementary Table 1: Raw data on journal sources of lncRNA publications extracted from the Web of Science Core Collection**

| **Source Titles** | **records** | **% of 3008** |
| --- | --- | --- |
| ONCOTARGET | 203 | 6.749 |
| TUMOR BIOLOGY | 125 | 4.156 |
| PLOS ONE | 118 | 3.923 |
| SCIENTIFIC REPORTS | 97 | 3.225 |
| INTERNATIONAL JOURNAL OF CLINICAL AND EXPERIMENTAL PATHOLOGY | 94 | 3.125 |
| NUCLEIC ACIDS RESEARCH | 46 | 1.529 |
| BIOCHEMICAL AND BIOPHYSICAL RESEARCH COMMUNICATIONS | 41 | 1.363 |
| ONCOLOGY REPORTS | 38 | 1.263 |
| MOLECULAR MEDICINE REPORTS | 34 | 1.13 |
| BMC GENOMICS | 33 | 1.097 |
| BIOMED RESEARCH INTERNATIONAL | 33 | 1.097 |
| MOLECULAR CELL | 32 | 1.064 |
| BIOMEDICINE PHARMACOTHERAPY | 32 | 1.064 |
| INTERNATIONAL JOURNAL OF MOLECULAR SCIENCES | 31 | 1.031 |
| ONCOLOGY LETTERS | 30 | 0.997 |
| NATURE COMMUNICATIONS | 30 | 0.997 |
| INTERNATIONAL JOURNAL OF CLINICAL AND EXPERIMENTAL MEDICINE | 30 | 0.997 |
| CANCER LETTERS | 29 | 0.964 |
| PROCEEDINGS OF THE NATIONAL ACADEMY OF SCIENCES OF THE UNITED STATES OF AMERICA | 27 | 0.898 |
| INTERNATIONAL JOURNAL OF ONCOLOGY | 26 | 0.864 |
| EUROPEAN REVIEW FOR MEDICAL AND PHARMACOLOGICAL SCIENCES | 26 | 0.864 |
| MOLECULAR CANCER | 25 | 0.831 |
| GENE | 25 | 0.831 |
| CELLULAR PHYSIOLOGY AND BIOCHEMISTRY | 25 | 0.831 |
| CELL DEATH DISEASE | 25 | 0.831 |
| BIOCHIMICA ET BIOPHYSICA ACTA GENE REGULATORY MECHANISMS | 25 | 0.831 |
| ONCOTARGETS AND THERAPY | 23 | 0.765 |
| AMERICAN JOURNAL OF TRANSLATIONAL RESEARCH | 22 | 0.731 |
| RNA BIOLOGY | 20 | 0.665 |
| PLOS GENETICS | 20 | 0.665 |
| GENOME BIOLOGY | 20 | 0.665 |
| CELL | 20 | 0.665 |
| CANCER RESEARCH | 19 | 0.632 |
| JOURNAL OF EXPERIMENTAL CLINICAL CANCER RESEARCH | 18 | 0.598 |
| ONCOGENE | 17 | 0.565 |
| MOLECULAR BIOSYSTEMS | 16 | 0.532 |
| CELL REPORTS | 16 | 0.532 |
| NATURE | 15 | 0.499 |
| MEDICAL ONCOLOGY | 15 | 0.499 |
| GENES DEVELOPMENT | 15 | 0.499 |
| JOURNAL OF HEMATOLOGY ONCOLOGY | 13 | 0.432 |
| GENOME RESEARCH | 13 | 0.432 |
| FEBS LETTERS | 13 | 0.432 |
| MEDICAL SCIENCE MONITOR | 12 | 0.399 |
| HEPATOLOGY | 12 | 0.399 |
| FRONTIERS IN GENETICS | 12 | 0.399 |
| EMBO JOURNAL | 12 | 0.399 |
| RNA | 11 | 0.366 |
| NEOPLASMA | 11 | 0.366 |
| NATURE STRUCTURAL MOLECULAR BIOLOGY | 11 | 0.366 |
| JOURNAL OF BIOLOGICAL CHEMISTRY | 11 | 0.366 |
| FEBS JOURNAL | 11 | 0.366 |
| BMC CANCER | 11 | 0.366 |
| AMERICAN JOURNAL OF CANCER RESEARCH | 11 | 0.366 |
| VIRUS RESEARCH | 10 | 0.332 |
| SCIENCE CHINA LIFE SCIENCES | 10 | 0.332 |
| MOLECULAR CARCINOGENESIS | 10 | 0.332 |
| MOLECULAR AND CELLULAR BIOLOGY | 10 | 0.332 |
| JOURNAL OF TRANSLATIONAL MEDICINE | 10 | 0.332 |
| EPIGENETICS | 10 | 0.332 |
| HUMAN MOLECULAR GENETICS | 9 | 0.299 |
| ELIFE | 9 | 0.299 |
| CIRCULATION RESEARCH | 9 | 0.299 |
| CELL STEM CELL | 9 | 0.299 |
| BIOINFORMATICS | 9 | 0.299 |
| WORLD JOURNAL OF GASTROENTEROLOGY | 8 | 0.266 |
| TRENDS IN GENETICS | 8 | 0.266 |
| SCIENCE | 8 | 0.266 |
| RNA A PUBLICATION OF THE RNA SOCIETY | 8 | 0.266 |
| MOLECULAR GENETICS AND GENOMICS | 8 | 0.266 |
| LONG NON CODING RNAS IN HUMAN DISEASE | 8 | 0.266 |
| JOURNAL OF CELLULAR AND MOLECULAR MEDICINE | 8 | 0.266 |
| JOURNAL OF CANCER RESEARCH AND CLINICAL ONCOLOGY | 8 | 0.266 |
| GENES | 8 | 0.266 |
| DEVELOPMENT | 8 | 0.266 |
| CURRENT TOPICS IN MICROBIOLOGY AND IMMUNOLOGY | 8 | 0.266 |
| CARCINOGENESIS | 8 | 0.266 |
| CANCER SCIENCE | 8 | 0.266 |
| STEM CELLS | 7 | 0.233 |
| MINERVA MEDICA | 7 | 0.233 |
| JOURNAL OF CELLULAR BIOCHEMISTRY | 7 | 0.233 |
| BRIEFINGS IN FUNCTIONAL GENOMICS | 7 | 0.233 |
| WILEY INTERDISCIPLINARY REVIEWS RNA | 6 | 0.199 |
| MOLECULAR THERAPY | 6 | 0.199 |
| MOLECULAR CANCER THERAPEUTICS | 6 | 0.199 |
| MEDICINE | 6 | 0.199 |
| JOURNAL OF MOLECULAR BIOLOGY | 6 | 0.199 |
| JOURNAL OF CANCER | 6 | 0.199 |
| IUBMB LIFE | 6 | 0.199 |
| INTERNATIONAL JOURNAL OF BIOCHEMISTRY CELL BIOLOGY | 6 | 0.199 |
| DNA AND CELL BIOLOGY | 6 | 0.199 |
| CELL RESEARCH | 6 | 0.199 |
| CANCER MEDICINE | 6 | 0.199 |
| CANCER CELL INTERNATIONAL | 6 | 0.199 |
| CANCER CELL | 6 | 0.199 |
| ASIAN PACIFIC JOURNAL OF CANCER PREVENTION | 6 | 0.199 |
| ANTICANCER RESEARCH | 6 | 0.199 |
| PFLUGERS ARCHIV EUROPEAN JOURNAL OF PHYSIOLOGY | 5 | 0.166 |
| OPEN LIFE SCIENCES | 5 | 0.166 |
| ONCOLOGY RESEARCH | 5 | 0.166 |
| NATURE GENETICS | 5 | 0.166 |
| MOLECULAR CANCER RESEARCH | 5 | 0.166 |
| MOLECULAR AND CELLULAR BIOCHEMISTRY | 5 | 0.166 |
| JOURNAL OF CLINICAL INVESTIGATION | 5 | 0.166 |
| INTERNATIONAL JOURNAL OF MOLECULAR MEDICINE | 5 | 0.166 |
| INTERNATIONAL JOURNAL OF GYNECOLOGICAL CANCER | 5 | 0.166 |
| GENOME MEDICINE | 5 | 0.166 |
| FRONTIERS IN CELLULAR NEUROSCIENCE | 5 | 0.166 |
| EMBO REPORTS | 5 | 0.166 |
| DEVELOPMENTAL CELL | 5 | 0.166 |
| CIRCULATION | 5 | 0.166 |
| CHROMOSOME RESEARCH | 5 | 0.166 |
| CELLULAR SIGNALLING | 5 | 0.166 |
| CELL AND BIOSCIENCE | 5 | 0.166 |
| BLOOD | 5 | 0.166 |
| BIOCHIMIE | 5 | 0.166 |
| BIOCHIMICA ET BIOPHYSICA ACTA MOLECULAR CELL RESEARCH | 5 | 0.166 |
| BIOCHIMICA ET BIOPHYSICA ACTA MOLECULAR BASIS OF DISEASE | 5 | 0.166 |
| ADVANCES IN EXPERIMENTAL MEDICINE AND BIOLOGY | 5 | 0.166 |
| TRENDS IN CELL BIOLOGY | 4 | 0.133 |
| TRENDS IN BIOCHEMICAL SCIENCES | 4 | 0.133 |
| TOXICOLOGY AND APPLIED PHARMACOLOGY | 4 | 0.133 |
| STEM CELLS INTERNATIONAL | 4 | 0.133 |
| SEMINARS IN CELL DEVELOPMENTAL BIOLOGY | 4 | 0.133 |
| PURINERGIC SIGNALLING | 4 | 0.133 |
| PLANT JOURNAL | 4 | 0.133 |
| NEW PHYTOLOGIST | 4 | 0.133 |
| NEUROSCIENCE | 4 | 0.133 |
| NEUROBIOLOGY OF DISEASE | 4 | 0.133 |
| NEOPLASIA | 4 | 0.133 |
| NATURE REVIEWS GENETICS | 4 | 0.133 |
| NATURE BIOTECHNOLOGY | 4 | 0.133 |
| MOLECULAR AND CELLULAR ENDOCRINOLOGY | 4 | 0.133 |
| METHODS | 4 | 0.133 |
| JOURNAL OF VIROLOGY | 4 | 0.133 |
| JOURNAL OF THORACIC ONCOLOGY | 4 | 0.133 |
| JOURNAL OF MOLECULAR NEUROSCIENCE | 4 | 0.133 |
| JOURNAL OF CELLULAR PHYSIOLOGY | 4 | 0.133 |
| GENOMICS | 4 | 0.133 |
| GENETICS AND MOLECULAR RESEARCH | 4 | 0.133 |
| EXPERIMENTAL DERMATOLOGY | 4 | 0.133 |
| EXPERIMENTAL AND THERAPEUTIC MEDICINE | 4 | 0.133 |
| EXPERIMENTAL AND MOLECULAR PATHOLOGY | 4 | 0.133 |
| DISCOVERY MEDICINE | 4 | 0.133 |
| DATABASE THE JOURNAL OF BIOLOGICAL DATABASES AND CURATION | 4 | 0.133 |
| CURRENT OPINION IN PLANT BIOLOGY | 4 | 0.133 |
| CURRENT OPINION IN GENETICS DEVELOPMENT | 4 | 0.133 |
| CELLULAR AND MOLECULAR LIFE SCIENCES | 4 | 0.133 |
| CANCER BIOMARKERS | 4 | 0.133 |
| BRITISH JOURNAL OF CANCER | 4 | 0.133 |
| BMC BIOINFORMATICS | 4 | 0.133 |
| BIOSCIENCE REPORTS | 4 | 0.133 |
| ANIMAL GENETICS | 4 | 0.133 |
| AMERICAN JOURNAL OF HUMAN GENETICS | 4 | 0.133 |
| AGING US | 4 | 0.133 |
| WORLD JOURNAL OF SURGICAL ONCOLOGY | 3 | 0.1 |
| TOXICOLOGY LETTERS | 3 | 0.1 |
| REPRODUCTION | 3 | 0.1 |
| PROSTATE CANCER AND PROSTATIC DISEASES | 3 | 0.1 |
| PHARMAZIE | 3 | 0.1 |
| PATHOLOGY RESEARCH AND PRACTICE | 3 | 0.1 |
| PATHOLOGY ONCOLOGY RESEARCH | 3 | 0.1 |
| ORAL ONCOLOGY | 3 | 0.1 |
| NATURE METHODS | 3 | 0.1 |
| NATURE CELL BIOLOGY | 3 | 0.1 |
| MOLECULES AND CELLS | 3 | 0.1 |
| MOLECULAR ENDOCRINOLOGY | 3 | 0.1 |
| MOLECULAR BIOLOGY REPORTS | 3 | 0.1 |
| MOLECULAR BIOLOGY OF THE CELL | 3 | 0.1 |
| MOLECULAR BIOLOGY AND EVOLUTION | 3 | 0.1 |
| MODERN PATHOLOGY | 3 | 0.1 |
| MEDIATORS OF INFLAMMATION | 3 | 0.1 |
| LUNG CANCER | 3 | 0.1 |
| JOURNAL OF STEROID BIOCHEMISTRY AND MOLECULAR BIOLOGY | 3 | 0.1 |
| JOURNAL OF NEUROCHEMISTRY | 3 | 0.1 |
| JOURNAL OF NEURO ONCOLOGY | 3 | 0.1 |
| JOURNAL OF MOLECULAR MEDICINE JMM | 3 | 0.1 |
| JOURNAL OF MOLECULAR AND CELLULAR CARDIOLOGY | 3 | 0.1 |
| JOURNAL OF INVESTIGATIVE DERMATOLOGY | 3 | 0.1 |
| JOURNAL OF IMMUNOLOGY | 3 | 0.1 |
| JOURNAL OF HUAZHONG UNIVERSITY OF SCIENCE AND TECHNOLOGY MEDICAL SCIENCES | 3 | 0.1 |
| JOURNAL OF GENETICS | 3 | 0.1 |
| JOURNAL OF EXPERIMENTAL BOTANY | 3 | 0.1 |
| INTERNATIONAL JOURNAL OF MEDICAL SCIENCES | 3 | 0.1 |
| INTERNATIONAL JOURNAL OF GENOMICS | 3 | 0.1 |
| INTERNATIONAL JOURNAL OF ENVIRONMENTAL RESEARCH AND PUBLIC HEALTH | 3 | 0.1 |
| INTERNATIONAL JOURNAL OF DEVELOPMENTAL NEUROSCIENCE | 3 | 0.1 |
| INTERNATIONAL JOURNAL OF CANCER | 3 | 0.1 |
| INTERNATIONAL JOURNAL OF BIOLOGICAL SCIENCES | 3 | 0.1 |
| GYNECOLOGIC ONCOLOGY | 3 | 0.1 |
| GUT | 3 | 0.1 |
| GENOME BIOLOGY AND EVOLUTION | 3 | 0.1 |
| FRONTIERS IN MOLECULAR NEUROSCIENCE | 3 | 0.1 |
| FEBS OPEN BIO | 3 | 0.1 |
| FASEB JOURNAL | 3 | 0.1 |
| EPIGENOMICS | 3 | 0.1 |
| EMBO MOLECULAR MEDICINE | 3 | 0.1 |
| DIGESTIVE DISEASES AND SCIENCES | 3 | 0.1 |
| CURRENT OPINION IN CELL BIOLOGY | 3 | 0.1 |
| COLD SPRING HARBOR PERSPECTIVES IN BIOLOGY | 3 | 0.1 |
| CLINICAL SCIENCE | 3 | 0.1 |
| CLINICAL LABORATORY | 3 | 0.1 |
| CLINICAL EPIGENETICS | 3 | 0.1 |
| CLINICA CHIMICA ACTA | 3 | 0.1 |
| CELL METABOLISM | 3 | 0.1 |
| CELL CYCLE | 3 | 0.1 |
| CELL AND TISSUE RESEARCH | 3 | 0.1 |
| CARDIOVASCULAR RESEARCH | 3 | 0.1 |
| CANCERS | 3 | 0.1 |
| CANCER BIOLOGY THERAPY | 3 | 0.1 |
| BREAST CANCER RESEARCH AND TREATMENT | 3 | 0.1 |
| BIOESSAYS | 3 | 0.1 |
| BIOCHEMISTRY | 3 | 0.1 |
| ARTHRITIS RESEARCH THERAPY | 3 | 0.1 |
| ARTERIOSCLEROSIS THROMBOSIS AND VASCULAR BIOLOGY | 3 | 0.1 |
| ARCHIVES OF ORAL BIOLOGY | 3 | 0.1 |
| AMERICAN JOURNAL OF REPRODUCTIVE IMMUNOLOGY | 3 | 0.1 |
| VIRUSES BASEL | 2 | 0.066 |
| VIROLOGY | 2 | 0.066 |
| UROLOGIC ONCOLOGY SEMINARS AND ORIGINAL INVESTIGATIONS | 2 | 0.066 |
| TRENDS IN MOLECULAR MEDICINE | 2 | 0.066 |
| THESCIENTIFICWORLDJOURNAL | 2 | 0.066 |
| STROKE | 2 | 0.066 |
| STEM CELLS AND DEVELOPMENT | 2 | 0.066 |
| SCIENTIFIC WORLD JOURNAL | 2 | 0.066 |
| RSC ADVANCES | 2 | 0.066 |
| RNA INFRASTRUCTURE AND NETWORKS | 2 | 0.066 |
| REPRODUCTIVE SCIENCES | 2 | 0.066 |
| PROSTATE | 2 | 0.066 |
| PLANT SCIENCE | 2 | 0.066 |
| PHILOSOPHICAL TRANSACTIONS OF THE ROYAL SOCIETY B BIOLOGICAL SCIENCES | 2 | 0.066 |
| NUCLEUS | 2 | 0.066 |
| NUCLEIC ACID THERAPEUTICS | 2 | 0.066 |
| NEUROSCIENCE LETTERS | 2 | 0.066 |
| NEURON | 2 | 0.066 |
| NEUROGASTROENTEROLOGY AND MOTILITY | 2 | 0.066 |
| NEUROCOMPUTING | 2 | 0.066 |
| NEUROBIOLOGY OF AGING | 2 | 0.066 |
| NATURE REVIEWS MOLECULAR CELL BIOLOGY | 2 | 0.066 |
| NATURE MEDICINE | 2 | 0.066 |
| NATURE IMMUNOLOGY | 2 | 0.066 |
| MUTATION RESEARCH REVIEWS IN MUTATION RESEARCH | 2 | 0.066 |
| MUTAGENESIS | 2 | 0.066 |
| MOLECULAR THERAPY NUCLEIC ACIDS | 2 | 0.066 |
| MOLECULAR REPRODUCTION AND DEVELOPMENT | 2 | 0.066 |
| MOLECULAR ONCOLOGY | 2 | 0.066 |
| MOLECULAR CELLULAR PROTEOMICS | 2 | 0.066 |
| MOLECULAR BRAIN | 2 | 0.066 |
| MOLECULAR BIOLOGY | 2 | 0.066 |
| MOLECULAR AND BIOCHEMICAL PARASITOLOGY | 2 | 0.066 |
| MEDICAL HYPOTHESES | 2 | 0.066 |
| MBIO | 2 | 0.066 |
| MARINE BIOTECHNOLOGY | 2 | 0.066 |
| LONG AND SHORT NONCODING RNAS IN CANCER BIOLOGY | 2 | 0.066 |
| JOURNAL OF UROLOGY | 2 | 0.066 |
| JOURNAL OF THORACIC DISEASE | 2 | 0.066 |
| JOURNAL OF THE AMERICAN COLLEGE OF CARDIOLOGY | 2 | 0.066 |
| JOURNAL OF ORTHOPAEDIC RESEARCH | 2 | 0.066 |
| JOURNAL OF MOLECULAR HISTOLOGY | 2 | 0.066 |
| JOURNAL OF LEUKOCYTE BIOLOGY | 2 | 0.066 |
| JOURNAL OF GASTROENTEROLOGY AND HEPATOLOGY | 2 | 0.066 |
| JOURNAL OF CLINICAL LABORATORY ANALYSIS | 2 | 0.066 |
| JOURNAL OF CLINICAL ENDOCRINOLOGY METABOLISM | 2 | 0.066 |
| JOURNAL OF CELL BIOLOGY | 2 | 0.066 |
| JOURNAL OF BIOMEDICAL SCIENCE | 2 | 0.066 |
| JOURNAL OF BIOCHEMISTRY | 2 | 0.066 |
| JOURNAL OF ALZHEIMERS DISEASE | 2 | 0.066 |
| JNCI JOURNAL OF THE NATIONAL CANCER INSTITUTE | 2 | 0.066 |
| JAPANESE JOURNAL OF CLINICAL ONCOLOGY | 2 | 0.066 |
| INTERNATIONAL REVIEW OF CELL AND MOLECULAR BIOLOGY | 2 | 0.066 |
| INFLAMMATORY BOWEL DISEASES | 2 | 0.066 |
| INDIAN JOURNAL OF CANCER | 2 | 0.066 |
| IMMUNOLOGIC RESEARCH | 2 | 0.066 |
| HYPERTENSION | 2 | 0.066 |
| GENETICS | 2 | 0.066 |
| GENES TO CELLS | 2 | 0.066 |
| GENES GENOMICS | 2 | 0.066 |
| GENES AND IMMUNITY | 2 | 0.066 |
| GASTROENTEROLOGY | 2 | 0.066 |
| G3 GENES GENOMES GENETICS | 2 | 0.066 |
| FRONTIERS IN PLANT SCIENCE | 2 | 0.066 |
| FRONTIERS IN PHYSIOLOGY | 2 | 0.066 |
| FRONTIERS IN IMMUNOLOGY | 2 | 0.066 |
| FISH SHELLFISH IMMUNOLOGY | 2 | 0.066 |
| EXPERT REVIEW OF GASTROENTEROLOGY HEPATOLOGY | 2 | 0.066 |
| EUROPEAN UROLOGY | 2 | 0.066 |
| EUROPEAN JOURNAL OF IMMUNOLOGY | 2 | 0.066 |
| EPIGENETICS CHROMATIN | 2 | 0.066 |
| ENDOCRINE RELATED CANCER | 2 | 0.066 |
| DISEASE MARKERS | 2 | 0.066 |
| DIFFERENTIATION | 2 | 0.066 |
| DIAGNOSTIC PATHOLOGY | 2 | 0.066 |
| CURRENT MOLECULAR MEDICINE | 2 | 0.066 |
| CNS NEUROSCIENCE THERAPEUTICS | 2 | 0.066 |
| CLINICAL CHEMISTRY | 2 | 0.066 |
| CLINICAL AND EXPERIMENTAL MEDICINE | 2 | 0.066 |
| CIRCULATION CARDIOVASCULAR GENETICS | 2 | 0.066 |
| CHINESE MEDICAL JOURNAL | 2 | 0.066 |
| CHEMMEDCHEM | 2 | 0.066 |
| CEREBRAL CORTEX | 2 | 0.066 |
| CELLULAR ONCOLOGY | 2 | 0.066 |
| CELLULAR MOLECULAR BIOLOGY LETTERS | 2 | 0.066 |
| CELLULAR AND MOLECULAR BIOLOGY | 2 | 0.066 |
| CELL PROLIFERATION | 2 | 0.066 |
| CELL DEATH AND DIFFERENTIATION | 2 | 0.066 |
| CELL BIOLOGY INTERNATIONAL | 2 | 0.066 |
| CANCER GENETICS | 2 | 0.066 |
| CANCER DISCOVERY | 2 | 0.066 |
| CANCER CHEMOTHERAPY AND PHARMACOLOGY | 2 | 0.066 |
| BRIEFINGS IN BIOINFORMATICS | 2 | 0.066 |
| BRAIN RESEARCH | 2 | 0.066 |
| BMC SYSTEMS BIOLOGY | 2 | 0.066 |
| BMC PLANT BIOLOGY | 2 | 0.066 |
| BMC NEUROSCIENCE | 2 | 0.066 |
| BMC MEDICAL GENOMICS | 2 | 0.066 |
| BMC EVOLUTIONARY BIOLOGY | 2 | 0.066 |
| BMC DEVELOPMENTAL BIOLOGY | 2 | 0.066 |
| BMC BIOLOGY | 2 | 0.066 |
| BMB REPORTS | 2 | 0.066 |
| BIOMATERIALS | 2 | 0.066 |
| BIOLOGY OF REPRODUCTION | 2 | 0.066 |
| BIOLOGY DIRECT | 2 | 0.066 |
| BIOLOGICAL CHEMISTRY | 2 | 0.066 |
| BIOCHIMICA ET BIOPHYSICA ACTA GENERAL SUBJECTS | 2 | 0.066 |
| BIOCHEMICAL PHARMACOLOGY | 2 | 0.066 |
| AUTONOMIC NEUROSCIENCE BASIC CLINICAL | 2 | 0.066 |
| ARTHRITIS RHEUMATOLOGY | 2 | 0.066 |
| ARCHIVES OF BIOCHEMISTRY AND BIOPHYSICS | 2 | 0.066 |
| ANTI CANCER DRUGS | 2 | 0.066 |
| ANTI CANCER AGENTS IN MEDICINAL CHEMISTRY | 2 | 0.066 |
| ANNALS OF SURGICAL ONCOLOGY | 2 | 0.066 |
| AMERICAN JOURNAL OF PATHOLOGY | 2 | 0.066 |
| AGEING RESEARCH REVIEWS | 2 | 0.066 |
| ACTA BIOCHIMICA POLONICA | 2 | 0.066 |
| ACTA BIOCHIMICA ET BIOPHYSICA SINICA | 2 | 0.066 |
| ZEBRAFISH | 1 | 0.033 |
| WORLD NEUROSURGERY | 1 | 0.033 |
| VIROLOGY JOURNAL | 1 | 0.033 |
| VIROLOGICA SINICA | 1 | 0.033 |
| VETERINARY QUARTERLY | 1 | 0.033 |
| UROLOGY | 1 | 0.033 |
| ULUSAL TRAVMA VE ACIL CERRAHI DERGISI TURKISH JOURNAL OF TRAUMA EMERGENCY SURGERY | 1 | 0.033 |
| TRENDS IN PLANT SCIENCE | 1 | 0.033 |
| TRENDS IN IMMUNOLOGY | 1 | 0.033 |
| TRENDS IN CARDIOVASCULAR MEDICINE | 1 | 0.033 |
| TREE GENETICS GENOMES | 1 | 0.033 |
| TRANSLATIONAL RESEARCH | 1 | 0.033 |
| TRANSLATIONAL PSYCHIATRY | 1 | 0.033 |
| TRANSLATIONAL ONCOLOGY | 1 | 0.033 |
| TRANSLATIONAL CANCER RESEARCH | 1 | 0.033 |
| TOXICOLOGY IN VITRO | 1 | 0.033 |
| TOXICOLOGY | 1 | 0.033 |
| TOXICOLOGICAL SCIENCES | 1 | 0.033 |
| THYROID | 1 | 0.033 |
| SYSTEMS BIOLOGY OF RNA BINDING PROTEINS | 1 | 0.033 |
| STEROIDS | 1 | 0.033 |
| STEM CELL REPORTS | 1 | 0.033 |
| SPRINGERPLUS | 1 | 0.033 |
| SKELETAL MUSCLE | 1 | 0.033 |
| SCIENTIFIC DATA | 1 | 0.033 |
| SCIENCEASIA | 1 | 0.033 |
| SCIENCE TRANSLATIONAL MEDICINE | 1 | 0.033 |
| SCIENCE SIGNALING | 1 | 0.033 |
| SCIENCE BULLETIN | 1 | 0.033 |
| SCHIZOPHRENIA RESEARCH | 1 | 0.033 |
| ROLE OF NON CODING RNAS IN BIOLOGY | 1 | 0.033 |
| REVIEWS IN THE NEUROSCIENCES | 1 | 0.033 |
| RESPIRATORY MEDICINE | 1 | 0.033 |
| REPRODUCTION FERTILITY AND DEVELOPMENT | 1 | 0.033 |
| RADIOLOGY AND ONCOLOGY | 1 | 0.033 |
| RADIOLOGY | 1 | 0.033 |
| RADIATION RESEARCH | 1 | 0.033 |
| PULMONARY CIRCULATION | 1 | 0.033 |
| PSYCHONEUROENDOCRINOLOGY | 1 | 0.033 |
| PSYCHIATRIC GENETICS | 1 | 0.033 |
| PROTEOMICS | 1 | 0.033 |
| PROGRESS IN NEURO PSYCHOPHARMACOLOGY BIOLOGICAL PSYCHIATRY | 1 | 0.033 |
| PROGRESS IN MOLECULAR BIOLOGY AND TRANSLATIONAL SCIENCE | 1 | 0.033 |
| PROCEEDINGS OF THE ROYAL SOCIETY B BIOLOGICAL SCIENCES | 1 | 0.033 |
| PLOS MEDICINE | 1 | 0.033 |
| PLOS COMPUTATIONAL BIOLOGY | 1 | 0.033 |
| PLOS BIOLOGY | 1 | 0.033 |
| PLANTA | 1 | 0.033 |
| PLANT PHYSIOLOGY | 1 | 0.033 |
| PLANT BIOTECHNOLOGY REPORTS | 1 | 0.033 |
| PLANT BIOTECHNOLOGY JOURNAL | 1 | 0.033 |
| PLANT AND CELL PHYSIOLOGY | 1 | 0.033 |
| PLACENTA | 1 | 0.033 |
| PITUITARY | 1 | 0.033 |
| PIGMENT CELL MELANOMA RESEARCH | 1 | 0.033 |
| PHYTOMEDICINE | 1 | 0.033 |
| PHYSIOLOGY BEHAVIOR | 1 | 0.033 |
| PHYSIOLOGICAL GENOMICS | 1 | 0.033 |
| PHARMACOLOGY THERAPEUTICS | 1 | 0.033 |
| PHARMACOLOGICAL RESEARCH | 1 | 0.033 |
| PHARMACEUTICAL RESEARCH | 1 | 0.033 |
| PERIODICUM BIOLOGORUM | 1 | 0.033 |
| PATHOLOGY | 1 | 0.033 |
| PATHOLOGIE BIOLOGIE | 1 | 0.033 |
| PARASITOLOGY RESEARCH | 1 | 0.033 |
| PANMINERVA MEDICA | 1 | 0.033 |
| PANCREATOLOGY | 1 | 0.033 |
| OSTEOARTHRITIS AND CARTILAGE | 1 | 0.033 |
| ORTHOPAEDIC SURGERY | 1 | 0.033 |
| ORAL SURGERY ORAL MEDICINE ORAL PATHOLOGY ORAL RADIOLOGY | 1 | 0.033 |
| OPEN MEDICINE | 1 | 0.033 |
| OPEN BIOLOGY | 1 | 0.033 |
| ONCOLOGIST | 1 | 0.033 |
| ONCOGENESIS | 1 | 0.033 |
| OBESITY | 1 | 0.033 |
| NEUROTHERAPEUTICS | 1 | 0.033 |
| NEUROCHEMISTRY INTERNATIONAL | 1 | 0.033 |
| NEUROBIOLOGY OF AUTISM | 1 | 0.033 |
| NEURO ONCOLOGY | 1 | 0.033 |
| NEURAL DEVELOPMENT | 1 | 0.033 |
| NATURE REVIEWS NEPHROLOGY | 1 | 0.033 |
| NATURE REVIEWS ENDOCRINOLOGY | 1 | 0.033 |
| NATURE REVIEWS DRUG DISCOVERY | 1 | 0.033 |
| NATURE REVIEWS CARDIOLOGY | 1 | 0.033 |
| NATURE NEUROSCIENCE | 1 | 0.033 |
| NATURE CHEMICAL BIOLOGY | 1 | 0.033 |
| MUTATION RESEARCH FUNDAMENTAL AND MOLECULAR MECHANISMS OF MUTAGENESIS | 1 | 0.033 |
| MOLECULAR VISION | 1 | 0.033 |
| MOLECULAR PSYCHIATRY | 1 | 0.033 |
| MOLECULAR PLANT | 1 | 0.033 |
| MOLECULAR PAIN | 1 | 0.033 |
| MOLECULAR NEUROBIOLOGY | 1 | 0.033 |
| MOLECULAR METABOLISM | 1 | 0.033 |
| MOLECULAR AND CELLULAR PROBES | 1 | 0.033 |
| MOLECULAR AND CELLULAR NEUROSCIENCE | 1 | 0.033 |
| MOLECULAR AND CELLULAR CHANGES IN THE CANCER CELL | 1 | 0.033 |
| METHODS IN ENZYMOLOGY | 1 | 0.033 |
| METABOLIC BRAIN DISEASE | 1 | 0.033 |
| MELANOMA RESEARCH | 1 | 0.033 |
| MEDICINAL RESEARCH REVIEWS | 1 | 0.033 |
| MAMMALIAN GENOME | 1 | 0.033 |
| LIVER INTERNATIONAL | 1 | 0.033 |
| LIFE SCIENCES | 1 | 0.033 |
| LEUKEMIA LYMPHOMA | 1 | 0.033 |
| LEUKEMIA | 1 | 0.033 |
| LABORATORY INVESTIGATION | 1 | 0.033 |
| LABORATORIUMSMEDIZIN JOURNAL OF LABORATORY MEDICINE | 1 | 0.033 |
| JUNDISHAPUR JOURNAL OF MICROBIOLOGY | 1 | 0.033 |
| JOURNAL OF TOXICOLOGY AND ENVIRONMENTAL HEALTH PART A CURRENT ISSUES | 1 | 0.033 |
| JOURNAL OF THE NEUROLOGICAL SCIENCES | 1 | 0.033 |
| JOURNAL OF RHEUMATOLOGY | 1 | 0.033 |
| JOURNAL OF PINEAL RESEARCH | 1 | 0.033 |
| JOURNAL OF PHYSIOLOGY AND BIOCHEMISTRY | 1 | 0.033 |
| JOURNAL OF PERIODONTOLOGY | 1 | 0.033 |
| JOURNAL OF OVARIAN RESEARCH | 1 | 0.033 |
| JOURNAL OF NUTRITIONAL BIOCHEMISTRY | 1 | 0.033 |
| JOURNAL OF NEUROSURGERY | 1 | 0.033 |
| JOURNAL OF NEUROSCIENCE | 1 | 0.033 |
| JOURNAL OF NEUROIMMUNOLOGY | 1 | 0.033 |
| JOURNAL OF NEURAL TRANSMISSION | 1 | 0.033 |
| JOURNAL OF NEPHROLOGY | 1 | 0.033 |
| JOURNAL OF NANOSCIENCE AND NANOTECHNOLOGY | 1 | 0.033 |
| JOURNAL OF MOLECULAR DIAGNOSTICS | 1 | 0.033 |
| JOURNAL OF MOLECULAR CELL BIOLOGY | 1 | 0.033 |
| JOURNAL OF MEDICAL BIOCHEMISTRY | 1 | 0.033 |
| JOURNAL OF MATERNAL FETAL NEONATAL MEDICINE | 1 | 0.033 |
| JOURNAL OF LIPID RESEARCH | 1 | 0.033 |
| JOURNAL OF KOREAN MEDICAL SCIENCE | 1 | 0.033 |
| JOURNAL OF INTERFERON AND CYTOKINE RESEARCH | 1 | 0.033 |
| JOURNAL OF INFECTIOUS DISEASES | 1 | 0.033 |
| JOURNAL OF INFECTION | 1 | 0.033 |
| JOURNAL OF IMMUNOLOGY RESEARCH | 1 | 0.033 |
| JOURNAL OF HEPATOLOGY | 1 | 0.033 |
| JOURNAL OF GENERAL VIROLOGY | 1 | 0.033 |
| JOURNAL OF GASTROENTEROLOGY | 1 | 0.033 |
| JOURNAL OF DERMATOLOGICAL SCIENCE | 1 | 0.033 |
| JOURNAL OF COMPARATIVE PHYSIOLOGY A NEUROETHOLOGY SENSORY NEURAL AND BEHAVIORAL PHYSIOLOGY | 1 | 0.033 |
| JOURNAL OF CLINICAL PATHOLOGY | 1 | 0.033 |
| JOURNAL OF CELL SCIENCE | 1 | 0.033 |
| JOURNAL OF CARDIOVASCULAR TRANSLATIONAL RESEARCH | 1 | 0.033 |
| JOURNAL OF CARDIOVASCULAR MEDICINE | 1 | 0.033 |
| JOURNAL OF BUON | 1 | 0.033 |
| JOURNAL OF BREAST CANCER | 1 | 0.033 |
| JOURNAL OF BONE ONCOLOGY | 1 | 0.033 |
| JOURNAL OF BIOSCIENCES | 1 | 0.033 |
| JOURNAL OF BIOSCIENCE AND BIOENGINEERING | 1 | 0.033 |
| JOURNAL OF BIOMOLECULAR SCREENING | 1 | 0.033 |
| JOURNAL OF BIOINFORMATICS AND COMPUTATIONAL BIOLOGY | 1 | 0.033 |
| JOURNAL OF AUTOIMMUNITY | 1 | 0.033 |
| JOURNAL OF ATHEROSCLEROSIS AND THROMBOSIS | 1 | 0.033 |
| JOURNAL OF ASSISTED REPRODUCTION AND GENETICS | 1 | 0.033 |
| JETP LETTERS | 1 | 0.033 |
| JAPANESE JOURNAL OF INFECTIOUS DISEASES | 1 | 0.033 |
| INVESTIGATIVE OPHTHALMOLOGY VISUAL SCIENCE | 1 | 0.033 |
| INTERNATIONAL UROLOGY AND NEPHROLOGY | 1 | 0.033 |
| INTERNATIONAL REVIEW OF NEUROBIOLOGY | 1 | 0.033 |
| INTERNATIONAL REVIEW OF CELL AND MOLECULAR BIOLOGY VOL 326 | 1 | 0.033 |
| INTERNATIONAL REVIEW OF CELL AND MOLECULAR BIOLOGY VOL 324 | 1 | 0.033 |
| INTERNATIONAL JOURNAL OF RHEUMATIC DISEASES | 1 | 0.033 |
| INTERNATIONAL JOURNAL OF RADIATION ONCOLOGY BIOLOGY PHYSICS | 1 | 0.033 |
| INTERNATIONAL JOURNAL OF HEMATOLOGY | 1 | 0.033 |
| INTERNATIONAL JOURNAL OF EXPERIMENTAL PATHOLOGY | 1 | 0.033 |
| INTERNATIONAL JOURNAL OF CHRONIC OBSTRUCTIVE PULMONARY DISEASE | 1 | 0.033 |
| INTERNATIONAL JOURNAL OF CARDIOLOGY | 1 | 0.033 |
| INTERNATIONAL JOURNAL OF BIOLOGICAL MACROMOLECULES | 1 | 0.033 |
| INTERNATIONAL IMMUNOPHARMACOLOGY | 1 | 0.033 |
| INFLAMMATION RESEARCH | 1 | 0.033 |
| IMMUNOLOGY AND CELL BIOLOGY | 1 | 0.033 |
| IMMUNOLOGICAL REVIEWS | 1 | 0.033 |
| IMMUNITY | 1 | 0.033 |
| HYPERTENSION RESEARCH | 1 | 0.033 |
| HUMAN MUTATION | 1 | 0.033 |
| HUMAN GENOMICS | 1 | 0.033 |
| HUMAN GENETICS | 1 | 0.033 |
| HUMAN CELL | 1 | 0.033 |
| HIV MEDICINE | 1 | 0.033 |
| HISTOPATHOLOGY | 1 | 0.033 |
| HISTOLOGY AND HISTOPATHOLOGY | 1 | 0.033 |
| HEPATOLOGY RESEARCH | 1 | 0.033 |
| HEPATITIS MONTHLY | 1 | 0.033 |
| HEMATOLOGY | 1 | 0.033 |
| HAEMATOLOGICA | 1 | 0.033 |
| GENES AND NUTRITION | 1 | 0.033 |
| GASTROENTEROLOGY RESEARCH AND PRACTICE | 1 | 0.033 |
| FUTURE VIROLOGY | 1 | 0.033 |
| FUTURE ONCOLOGY | 1 | 0.033 |
| FRONTIERS IN NEUROSCIENCE | 1 | 0.033 |
| FRONTIERS IN ENDOCRINOLOGY | 1 | 0.033 |
| FRONTIERS IN BIOSCIENCE LANDMARK | 1 | 0.033 |
| FOOD FUNCTION | 1 | 0.033 |
| EXPERT REVIEW OF MOLECULAR DIAGNOSTICS | 1 | 0.033 |
| EXPERT OPINION ON THERAPEUTIC TARGETS | 1 | 0.033 |
| EXPERT OPINION ON DRUG DISCOVERY | 1 | 0.033 |
| EXPERIMENTAL NEUROLOGY | 1 | 0.033 |
| EXPERIMENTAL CELL RESEARCH | 1 | 0.033 |
| EXPERIMENTAL BIOLOGY AND MEDICINE | 1 | 0.033 |
| EXPERIMENTAL AND MOLECULAR MEDICINE | 1 | 0.033 |
| EVOLUTION DEVELOPMENT | 1 | 0.033 |
| EVIDENCE BASED COMPLEMENTARY AND ALTERNATIVE MEDICINE | 1 | 0.033 |
| EUROPEAN JOURNAL OF OBSTETRICS GYNECOLOGY AND REPRODUCTIVE BIOLOGY | 1 | 0.033 |
| EUROPEAN JOURNAL OF NEUROSCIENCE | 1 | 0.033 |
| EUROPEAN JOURNAL OF MEDICAL GENETICS | 1 | 0.033 |
| EUROPEAN JOURNAL OF HUMAN GENETICS | 1 | 0.033 |
| EUROPEAN JOURNAL OF CARDIO THORACIC SURGERY | 1 | 0.033 |
| EUROPEAN JOURNAL OF CANCER | 1 | 0.033 |
| EUROPEAN HEART JOURNAL | 1 | 0.033 |
| ESSAYS IN BIOCHEMISTRY | 1 | 0.033 |
| ENZYMES OF EPIGENETICS PT A | 1 | 0.033 |
| ENVIRONMENTAL AND MOLECULAR MUTAGENESIS | 1 | 0.033 |
| ENDOCRINE REVIEWS | 1 | 0.033 |
| ENDOCRINE | 1 | 0.033 |
| DRUG RESISTANCE UPDATES | 1 | 0.033 |
| DNA REPAIR | 1 | 0.033 |
| DISEASES OF THE ESOPHAGUS | 1 | 0.033 |
| DIGESTIVE AND LIVER DISEASE | 1 | 0.033 |
| DIABETOLOGIA | 1 | 0.033 |
| DIABETES OBESITY METABOLISM | 1 | 0.033 |
| DIABETES | 1 | 0.033 |
| DEVELOPMENTAL NEUROSCIENCE | 1 | 0.033 |
| DEVELOPMENTAL BIOLOGY | 1 | 0.033 |
| DEVELOPMENTAL AND COMPARATIVE IMMUNOLOGY | 1 | 0.033 |
| DEVELOPMENT GROWTH DIFFERENTIATION | 1 | 0.033 |
| CYTOTECHNOLOGY | 1 | 0.033 |
| CYTOKINE | 1 | 0.033 |
| CURRENT TOPICS IN MEDICINAL CHEMISTRY | 1 | 0.033 |
| CURRENT PHARMACEUTICAL DESIGN | 1 | 0.033 |
| CURRENT OPINION IN INSECT SCIENCE | 1 | 0.033 |
| CURRENT OPINION IN IMMUNOLOGY | 1 | 0.033 |
| CURRENT NEUROVASCULAR RESEARCH | 1 | 0.033 |
| CURRENT GENOMICS | 1 | 0.033 |
| CURRENT GENETICS | 1 | 0.033 |
| CURRENT DRUG TARGETS | 1 | 0.033 |
| CURRENT ALZHEIMER RESEARCH | 1 | 0.033 |
| CTS CLINICAL AND TRANSLATIONAL SCIENCE | 1 | 0.033 |
| CRITICAL REVIEWS IN EUKARYOTIC GENE EXPRESSION | 1 | 0.033 |
| CRITICAL REVIEWS IN CLINICAL LABORATORY SCIENCES | 1 | 0.033 |
| CORNEA | 1 | 0.033 |
| COMPUTATIONAL BIOLOGY AND CHEMISTRY | 1 | 0.033 |
| COMPUTATIONAL AND MATHEMATICAL METHODS IN MEDICINE | 1 | 0.033 |
| COLD SPRING HARBOR PERSPECTIVES IN MEDICINE | 1 | 0.033 |
| CLINICAL TRANSLATIONAL ONCOLOGY | 1 | 0.033 |
| CLINICAL PHARMACOLOGY THERAPEUTICS | 1 | 0.033 |
| CLINICAL INTERVENTIONS IN AGING | 1 | 0.033 |
| CLINICAL EXPERIMENTAL METASTASIS | 1 | 0.033 |
| CLINICAL CANCER RESEARCH | 1 | 0.033 |
| CLINICAL BIOCHEMISTRY | 1 | 0.033 |
| CHEST | 1 | 0.033 |
| CHEMICO BIOLOGICAL INTERACTIONS | 1 | 0.033 |
| CHEMICAL BIOLOGY DRUG DESIGN | 1 | 0.033 |
| CELLULAR REPROGRAMMING | 1 | 0.033 |
| CELLULAR MOLECULAR IMMUNOLOGY | 1 | 0.033 |
| CELLULAR AND MOLECULAR NEUROBIOLOGY | 1 | 0.033 |
| CELL JOURNAL | 1 | 0.033 |
| CELL HOST MICROBE | 1 | 0.033 |
| CELL CHEMICAL BIOLOGY | 1 | 0.033 |
| CELL BIOLOGY AND TOXICOLOGY | 1 | 0.033 |
| CELL BIOCHEMISTRY AND FUNCTION | 1 | 0.033 |
| CELL BIOCHEMISTRY AND BIOPHYSICS | 1 | 0.033 |
| CARDIOVASCULAR PATHOLOGY | 1 | 0.033 |
| CARDIOLOGY | 1 | 0.033 |
| CANCER RESEARCH AND TREATMENT | 1 | 0.033 |
| CANCER INVESTIGATION | 1 | 0.033 |
| CANCER GENE THERAPY | 1 | 0.033 |
| CANCER EPIDEMIOLOGY | 1 | 0.033 |
| CANCER BIOTHERAPY AND RADIOPHARMACEUTICALS | 1 | 0.033 |
| CANCER | 1 | 0.033 |
| CANADIAN JOURNAL OF PHYSIOLOGY AND PHARMACOLOGY | 1 | 0.033 |
| BREAST CANCER RESEARCH | 1 | 0.033 |
| BREAST CANCER | 1 | 0.033 |
| BRAIN RESEARCH BULLETIN | 1 | 0.033 |
| BMJ OPEN | 1 | 0.033 |
| BMC UROLOGY | 1 | 0.033 |
| BMC PREGNANCY AND CHILDBIRTH | 1 | 0.033 |
| BMC COMPLEMENTARY AND ALTERNATIVE MEDICINE | 1 | 0.033 |
| BMC CARDIOVASCULAR DISORDERS | 1 | 0.033 |
| BLOOD CELLS MOLECULES AND DISEASES | 1 | 0.033 |
| BLOOD CANCER JOURNAL | 1 | 0.033 |
| BIOSENSORS BIOELECTRONICS | 1 | 0.033 |
| BIOSCIENCE TRENDS | 1 | 0.033 |
| BIOSCIENCE BIOTECHNOLOGY AND BIOCHEMISTRY | 1 | 0.033 |
| BIOMOLECULES THERAPEUTICS | 1 | 0.033 |
| BIOMEDICAL AND ENVIRONMENTAL SCIENCES | 1 | 0.033 |
| BIOMARKERS IN MEDICINE | 1 | 0.033 |
| BIOMARKERS | 1 | 0.033 |
| BIOLOGICAL RESEARCH | 1 | 0.033 |
| BIOLOGICAL PSYCHIATRY | 1 | 0.033 |
| BIOLOGICAL PROCEDURES ONLINE | 1 | 0.033 |
| BIOLOGIA PLANTARUM | 1 | 0.033 |
| BIODATA MINING | 1 | 0.033 |
| BIOCONJUGATE CHEMISTRY | 1 | 0.033 |
| BIOCHIMICA ET BIOPHYSICA ACTA REVIEWS ON CANCER | 1 | 0.033 |
| BIOCHEMICAL SOCIETY TRANSACTIONS | 1 | 0.033 |
| BANGLADESH JOURNAL OF PHARMACOLOGY | 1 | 0.033 |
| AUTOPHAGY | 1 | 0.033 |
| AUTOIMMUNITY REVIEWS | 1 | 0.033 |
| AUTOIMMUNITY | 1 | 0.033 |
| ATHEROSCLEROSIS | 1 | 0.033 |
| ASN NEURO | 1 | 0.033 |
| ASIAN PACIFIC JOURNAL OF TROPICAL MEDICINE | 1 | 0.033 |
| ASIAN JOURNAL OF ANDROLOGY | 1 | 0.033 |
| ARCHIVES OF MEDICAL RESEARCH | 1 | 0.033 |
| ARCHIVES OF IRANIAN MEDICINE | 1 | 0.033 |
| ARCHIVES OF GYNECOLOGY AND OBSTETRICS | 1 | 0.033 |
| AQUATIC TOXICOLOGY | 1 | 0.033 |
| APPLIED ENTOMOLOGY AND ZOOLOGY | 1 | 0.033 |
| APPLIED BIOCHEMISTRY AND BIOTECHNOLOGY | 1 | 0.033 |
| APMIS | 1 | 0.033 |
| ANNUAL REVIEW OF GENOMICS AND HUMAN GENETICS VOL 17 | 1 | 0.033 |
| ANNUAL REVIEW OF GENOMICS AND HUMAN GENETICS | 1 | 0.033 |
| ANNUAL REVIEW OF GENETICS VOL 48 | 1 | 0.033 |
| ANNUAL REVIEW OF GENETICS | 1 | 0.033 |
| ANNUAL REVIEW OF BIOCHEMISTRY VOL 81 | 1 | 0.033 |
| ANNUAL REVIEW OF BIOCHEMISTRY | 1 | 0.033 |
| ANNALS OF CLINICAL AND LABORATORY SCIENCE | 1 | 0.033 |
| AMERICAN JOURNAL OF RESPIRATORY CELL AND MOLECULAR BIOLOGY | 1 | 0.033 |
| AMERICAN JOURNAL OF PHYSIOLOGY RENAL PHYSIOLOGY | 1 | 0.033 |
| AMERICAN JOURNAL OF PHYSIOLOGY REGULATORY INTEGRATIVE AND COMPARATIVE PHYSIOLOGY | 1 | 0.033 |
| AMERICAN JOURNAL OF PHYSIOLOGY HEART AND CIRCULATORY PHYSIOLOGY | 1 | 0.033 |
| AMERICAN JOURNAL OF PHYSIOLOGY GASTROINTESTINAL AND LIVER PHYSIOLOGY | 1 | 0.033 |
| AMERICAN JOURNAL OF PHYSIOLOGY CELL PHYSIOLOGY | 1 | 0.033 |
| AMERICAN JOURNAL OF MEDICAL GENETICS PART A | 1 | 0.033 |
| AGING CELL | 1 | 0.033 |
| ADVANCES IN IMMUNOLOGY VOL 104 | 1 | 0.033 |
| ADVANCES IN IMMUNOLOGY | 1 | 0.033 |
| ADVANCED FUNCTIONAL MATERIALS | 1 | 0.033 |
| ADVANCED DRUG DELIVERY REVIEWS | 1 | 0.033 |
| ACTA PHYSIOLOGICA | 1 | 0.033 |
| ACS CHEMICAL BIOLOGY | 1 | 0.033 |
| (653 Source Titles {0} {1} value(s) outside display options.) | | |
| (0 records (0.000%){0} records{1} do not contain data in the field being analyzed.) | | |
