# Supplementary material for: Trends of long noncoding RNA research from 2007 to 2016: a bibliometric analysis
Source: Oncotarget. 2017 Sep 12;8(47):83114–27. doi: 10.18632/oncotarget.20851 (PMC5669954; doi:10.18632/oncotarget.20851)
Supplement: Supplementary file 3 [file oncotarget-08-83114-s003.docx]

**Supplementary Table 2: Raw data on countries/territories involved in lncRNA publications extracted from the Web of Science Core Collection**

| **Countries/Territories** | **records** | **% of 3008** |
| --- | --- | --- |
| PEOPLES R CHINA | 1843 | 61.27 |
| USA | 779 | 25.898 |
| GERMANY | 108 | 3.59 |
| ENGLAND | 100 | 3.324 |
| JAPAN | 97 | 3.225 |
| AUSTRALIA | 88 | 2.926 |
| ITALY | 78 | 2.593 |
| SPAIN | 57 | 1.895 |
| FRANCE | 51 | 1.695 |
| CANADA | 51 | 1.695 |
| INDIA | 38 | 1.263 |
| SWITZERLAND | 36 | 1.197 |
| NETHERLANDS | 32 | 1.064 |
| SWEDEN | 30 | 0.997 |
| SOUTH KOREA | 29 | 0.964 |
| TAIWAN | 27 | 0.898 |
| SINGAPORE | 23 | 0.765 |
| ISRAEL | 20 | 0.665 |
| BRAZIL | 20 | 0.665 |
| BELGIUM | 20 | 0.665 |
| SCOTLAND | 18 | 0.598 |
| DENMARK | 17 | 0.565 |
| IRAN | 14 | 0.465 |
| AUSTRIA | 14 | 0.465 |
| NORWAY | 9 | 0.299 |
| TURKEY | 8 | 0.266 |
| POLAND | 8 | 0.266 |
| SAUDI ARABIA | 7 | 0.233 |
| IRELAND | 7 | 0.233 |
| SOUTH AFRICA | 6 | 0.199 |
| MEXICO | 6 | 0.199 |
| LUXEMBOURG | 6 | 0.199 |
| CHILE | 6 | 0.199 |
| PORTUGAL | 5 | 0.166 |
| GREECE | 5 | 0.166 |
| EGYPT | 5 | 0.166 |
| RUSSIA | 4 | 0.133 |
| ROMANIA | 4 | 0.133 |
| NEW ZEALAND | 4 | 0.133 |
| FINLAND | 4 | 0.133 |
| CZECH REPUBLIC | 4 | 0.133 |
| SLOVENIA | 3 | 0.1 |
| COLOMBIA | 3 | 0.1 |
| HUNGARY | 2 | 0.066 |
| CROATIA | 2 | 0.066 |
| WALES | 1 | 0.033 |
| THAILAND | 1 | 0.033 |
| SRI LANKA | 1 | 0.033 |
| SLOVAKIA | 1 | 0.033 |
| PAKISTAN | 1 | 0.033 |
| MALAYSIA | 1 | 0.033 |
| KENYA | 1 | 0.033 |
| ICELAND | 1 | 0.033 |
| GHANA | 1 | 0.033 |
| ESTONIA | 1 | 0.033 |
| CYPRUS | 1 | 0.033 |
| ARGENTINA | 1 | 0.033 |
| (47 Countries/Territories {0} {1} value(s) outside display options.) | | |
| (1 records (0.033%){0} records{1} do not contain data in the field being analyzed.) | | |
