# Supplementary material for: Trends of long noncoding RNA research from 2007 to 2016: a bibliometric analysis
Source: Oncotarget. 2017 Sep 12;8(47):83114–27. doi: 10.18632/oncotarget.20851 (PMC5669954; doi:10.18632/oncotarget.20851)
Supplement: Supplementary file 4 [file oncotarget-08-83114-s004.docx]

**Supplementary Table 3: Raw data on institutions involved in lncRNA publications extracted from the Web of Science Core Collection**

| **Organizations** | **records** | **% of 3008** |
| --- | --- | --- |
| NANJING MED UNIV | 225 | 7.48 |
| SHANGHAI JIAO TONG UNIV | 125 | 4.156 |
| CHINESE ACAD SCI | 102 | 3.391 |
| HARBIN MED UNIV | 89 | 2.959 |
| FUDAN UNIV | 80 | 2.66 |
| HARVARD UNIV | 78 | 2.593 |
| SUN YAT SEN UNIV | 75 | 2.493 |
| SECOND MIL MED UNIV | 63 | 2.094 |
| SHANDONG UNIV | 61 | 2.028 |
| SOOCHOW UNIV | 60 | 1.995 |
| TONGJI UNIV | 56 | 1.862 |
| PEKING UNIV | 54 | 1.795 |
| CHINESE ACAD MED SCI | 54 | 1.795 |
| STANFORD UNIV | 52 | 1.729 |
| ZHEJIANG UNIV | 51 | 1.695 |
| UNIV TEXAS MD ANDERSON CANC CTR | 51 | 1.695 |
| CENT S UNIV | 46 | 1.529 |
| UNIV MICHIGAN | 41 | 1.363 |
| SOUTHERN MED UNIV | 41 | 1.363 |
| WENZHOU MED UNIV | 40 | 1.33 |
| MIT | 40 | 1.33 |
| XI AN JIAO TONG UNIV | 39 | 1.297 |
| CHINA MED UNIV | 39 | 1.297 |
| ZHENGZHOU UNIV | 38 | 1.263 |
| SOUTHEAST UNIV | 37 | 1.23 |
| ANHUI MED UNIV | 37 | 1.23 |
| NANJING UNIV | 36 | 1.197 |
| PEKING UNION MED COLL | 35 | 1.164 |
| JILIN UNIV | 35 | 1.164 |
| WUHAN UNIV | 30 | 0.997 |
| UNIV OXFORD | 30 | 0.997 |
| UNIV NEW S WALES | 29 | 0.964 |
| NANCHANG UNIV | 29 | 0.964 |
| GUANGZHOU MED UNIV | 29 | 0.964 |
| CHINESE PEOPLES LIBERAT ARMY GEN HOSP | 29 | 0.964 |
| TIANJIN MED UNIV | 28 | 0.931 |
| NINGBO UNIV | 28 | 0.931 |
| CAPITAL MED UNIV | 28 | 0.931 |
| UNIV QUEENSLAND | 26 | 0.864 |
| THIRD MIL MED UNIV | 24 | 0.798 |
| SICHUAN UNIV | 23 | 0.765 |
| HUAZHONG UNIV SCI TECHNOL | 23 | 0.765 |
| YALE UNIV | 22 | 0.731 |
| UNIV TOKYO | 22 | 0.731 |
| UNIV PENN | 21 | 0.698 |
| UNIV CHINESE ACAD SCI | 21 | 0.698 |
| JOHNS HOPKINS UNIV | 21 | 0.698 |
| FUJIAN MED UNIV | 21 | 0.698 |
| YANGZHOU UNIV | 20 | 0.665 |
| UNIV MIAMI | 19 | 0.632 |
| CHINESE UNIV HONG KONG | 19 | 0.632 |
| UNIV HONG KONG | 18 | 0.598 |
| UNIV CALIF SAN FRANCISCO | 18 | 0.598 |
| SCRIPPS RES INST | 18 | 0.598 |
| NANTONG UNIV | 18 | 0.598 |
| SHANTOU UNIV | 17 | 0.565 |
| RIKEN | 17 | 0.565 |
| WAYNE STATE UNIV | 16 | 0.532 |
| UNIV SCI TECHNOL CHINA | 16 | 0.532 |
| UNIV MASSACHUSETTS | 16 | 0.532 |
| UNIV MARYLAND | 16 | 0.532 |
| TSINGHUA UNIV | 16 | 0.532 |
| NIA | 16 | 0.532 |
| JINAN UNIV | 16 | 0.532 |
| JIANGSU UNIV | 16 | 0.532 |
| CHONGQING MED UNIV | 16 | 0.532 |
| BAYLOR COLL MED | 16 | 0.532 |
| UNIV SAO PAULO | 15 | 0.499 |
| UNIV NAVARRA | 15 | 0.499 |
| UNIV LAUSANNE | 15 | 0.499 |
| UNIV CALIF LOS ANGELES | 15 | 0.499 |
| MAYO CLIN | 15 | 0.499 |
| MASSACHUSETTS GEN HOSP | 15 | 0.499 |
| BROAD INST MIT HARVARD | 15 | 0.499 |
| XINXIANG MED UNIV | 14 | 0.465 |
| WHITEHEAD INST BIOMED RES | 14 | 0.465 |
| SHENZHEN UNIV | 14 | 0.465 |
| NATL UNIV SINGAPORE | 14 | 0.465 |
| HOWARD HUGHES MED INST | 14 | 0.465 |
| GUANGXI MED UNIV | 14 | 0.465 |
| GERMAN CANC RES CTR | 14 | 0.465 |
| GARVAN INST MED RES | 14 | 0.465 |
| FOURTH MIL MED UNIV | 14 | 0.465 |
| CASE WESTERN RESERVE UNIV | 14 | 0.465 |
| UPF | 13 | 0.432 |
| UNIV LONDON IMPERIAL COLL SCI TECHNOL MED | 13 | 0.432 |
| HEBEI MED UNIV | 13 | 0.432 |
| HARBIN INST TECHNOL | 13 | 0.432 |
| HANNOVER MED SCH | 13 | 0.432 |
| WISTAR INST ANAT BIOL | 12 | 0.399 |
| UNIV WASHINGTON | 12 | 0.399 |
| UNIV ROMA LA SAPIENZA | 12 | 0.399 |
| UNIV EDINBURGH | 12 | 0.399 |
| UNIV CALIF SAN DIEGO | 12 | 0.399 |
| OHIO STATE UNIV | 12 | 0.399 |
| KUNMING MED UNIV | 12 | 0.399 |
| HOKKAIDO UNIV | 12 | 0.399 |
| DALIAN MED UNIV | 12 | 0.399 |
| XIAMEN UNIV | 11 | 0.366 |
| UNIV TORONTO | 11 | 0.366 |
| UNIV TEXAS SW MED CTR DALLAS | 11 | 0.366 |
| QINGDAO UNIV | 11 | 0.366 |
| NORTHWESTERN UNIV | 11 | 0.366 |
| NORTHWEST A F UNIV | 11 | 0.366 |
| DUKE UNIV | 11 | 0.366 |
| XINJIANG MED UNIV | 10 | 0.332 |
| VANDERBILT UNIV | 10 | 0.332 |
| UPPSALA UNIV | 10 | 0.332 |
| UNIV UTAH | 10 | 0.332 |
| UNIV ROCHESTER | 10 | 0.332 |
| UNIV MILAN | 10 | 0.332 |
| UNIV ILLINOIS | 10 | 0.332 |
| UNIV GHENT | 10 | 0.332 |
| UNIV COPENHAGEN | 10 | 0.332 |
| UNIV BRITISH COLUMBIA | 10 | 0.332 |
| SEOUL NATL UNIV | 10 | 0.332 |
| SANFORD BURNHAM MED RES INST | 10 | 0.332 |
| NCI | 10 | 0.332 |
| NATL INST ADV IND SCI TECHNOL | 10 | 0.332 |
| MINIST EDUC | 10 | 0.332 |
| MAX PLANCK INST MOL GENET | 10 | 0.332 |
| KAROLINSKA INST | 10 | 0.332 |
| DANA FARBER CANC INST | 10 | 0.332 |
| COLUMBIA UNIV | 10 | 0.332 |
| CNRS | 10 | 0.332 |
| ALBERT EINSTEIN COLL MED | 10 | 0.332 |
| WEIZMANN INST SCI | 9 | 0.299 |
| UNIV WESTERN AUSTRALIA | 9 | 0.299 |
| UNIV PARIS 06 | 9 | 0.299 |
| UNIV MUNICH | 9 | 0.299 |
| UNIV MINNESOTA | 9 | 0.299 |
| UNIV GOTHENBURG | 9 | 0.299 |
| UNIV CHICAGO | 9 | 0.299 |
| UNIV CALIF DAVIS | 9 | 0.299 |
| UNIV BARCELONA | 9 | 0.299 |
| PEOPLES LIBERAT ARMY GEN HOSP | 9 | 0.299 |
| NATL TAIWAN UNIV | 9 | 0.299 |
| MEM SLOAN KETTERING CANC CTR | 9 | 0.299 |
| HENAN UNIV | 9 | 0.299 |
| GOETHE UNIV FRANKFURT | 9 | 0.299 |
| GENOME INST SINGAPORE | 9 | 0.299 |
| COLD SPRING HARBOR LAB | 9 | 0.299 |
| CHINESE PEOPLES LIBERAT ARMY | 9 | 0.299 |
| CHINESE ACAD AGR SCI | 9 | 0.299 |
| ZHEJIANG CANC HOSP | 8 | 0.266 |
| YONSEI UNIV | 8 | 0.266 |
| WASHINGTON UNIV | 8 | 0.266 |
| UNIV MISSISSIPPI | 8 | 0.266 |
| UNIV HEIDELBERG HOSP | 8 | 0.266 |
| UNIV CONNECTICUT | 8 | 0.266 |
| UNIV COLORADO | 8 | 0.266 |
| UNIV CAMBRIDGE | 8 | 0.266 |
| UCL | 8 | 0.266 |
| TEMPLE UNIV | 8 | 0.266 |
| MCGILL UNIV | 8 | 0.266 |
| ISIS PHARMACEUT | 8 | 0.266 |
| ICREA | 8 | 0.266 |
| HUAZHONG AGR UNIV | 8 | 0.266 |
| HENAN UNIV SCI TECHNOL | 8 | 0.266 |
| CSIR INST GENOM INTEGRAT BIOL | 8 | 0.266 |
| CNR | 8 | 0.266 |
| BETH ISRAEL DEACONESS MED CTR | 8 | 0.266 |
| BEIJING UNIV CHEM TECHNOL | 8 | 0.266 |
| WANNAN MED COLL | 7 | 0.233 |
| UNIV TEXAS HLTH SCI CTR HOUSTON | 7 | 0.233 |
| UNIV N CAROLINA | 7 | 0.233 |
| UNIV HOUSTON | 7 | 0.233 |
| UNIV FREIBURG | 7 | 0.233 |
| UNIV ELECT SCI TECHNOL CHINA | 7 | 0.233 |
| UNIV BATH | 7 | 0.233 |
| ST VINCENTS HOSP | 7 | 0.233 |
| PLA | 7 | 0.233 |
| NHLBI | 7 | 0.233 |
| NANYANG TECHNOL UNIV | 7 | 0.233 |
| MICHIGAN STATE UNIV | 7 | 0.233 |
| LOS ALAMOS NATL LAB | 7 | 0.233 |
| ICAHN SCH MED MT SINAI | 7 | 0.233 |
| HUAIAN 2 HOSP | 7 | 0.233 |
| HARVARD MED SCH | 7 | 0.233 |
| GUANGDONG MED COLL | 7 | 0.233 |
| CORNELL UNIV | 7 | 0.233 |
| CHINA AGR UNIV | 7 | 0.233 |
| BINZHOU MED UNIV | 7 | 0.233 |
| BEIJING INST RADIAT MED | 7 | 0.233 |
| BEIJING FORESTRY UNIV | 7 | 0.233 |
| BABRAHAM INST | 7 | 0.233 |
| ACAD SCI INNOVAT RES ACSIR | 7 | 0.233 |
| UNIV WITWATERSRAND | 6 | 0.199 |
| UNIV VIRGINIA | 6 | 0.199 |
| UNIV SYDNEY | 6 | 0.199 |
| UNIV SOUTH CHINA | 6 | 0.199 |
| UNIV SO CALIF | 6 | 0.199 |
| UNIV FERRARA | 6 | 0.199 |
| UNIV CALIF BERKELEY | 6 | 0.199 |
| UNIV ALABAMA BIRMINGHAM | 6 | 0.199 |
| SHANGHAI UNIV TRADIT CHINESE MED | 6 | 0.199 |
| SHANGHAI KEY LAB FEMALE REPROD ENDOCRINE RELATED | 6 | 0.199 |
| SAN FRANCISCO VA MED CTR | 6 | 0.199 |
| NEW YORK STATE INST BASIC RES DEV DISABIL | 6 | 0.199 |
| MINIST HLTH | 6 | 0.199 |
| MAASTRICHT UNIV | 6 | 0.199 |
| LINYI PEOPLES HOSP | 6 | 0.199 |
| KYUSHU UNIV | 6 | 0.199 |
| KEELE UNIV | 6 | 0.199 |
| KATHOLIEKE UNIV LEUVEN | 6 | 0.199 |
| JAWAHARLAL NEHRU UNIV | 6 | 0.199 |
| ISTANBUL UNIV | 6 | 0.199 |
| INST PASTEUR | 6 | 0.199 |
| INSERM | 6 | 0.199 |
| HEBREW UNIV JERUSALEM | 6 | 0.199 |
| HARVARD STEM CELL INST | 6 | 0.199 |
| GUANGDONG MED UNIV | 6 | 0.199 |
| CITY HOPE NATL MED CTR | 6 | 0.199 |
| CHINA MED UNIV HOSP | 6 | 0.199 |
| CHILDRENS HOSP PHILADELPHIA | 6 | 0.199 |
| CALTECH | 6 | 0.199 |
| BRITISH COLUMBIA CANC AGCY | 6 | 0.199 |
| YANTAI YUHUANGDING HOSP | 5 | 0.166 |
| XUZHOU MED UNIV | 5 | 0.166 |
| XUZHOU MED COLL | 5 | 0.166 |
| XIDIAN UNIV | 5 | 0.166 |
| WEILL CORNELL MED COLL | 5 | 0.166 |
| WEIFANG MED UNIV | 5 | 0.166 |
| VIB | 5 | 0.166 |
| UNIV WISCONSIN | 5 | 0.166 |
| UNIV VERMONT | 5 | 0.166 |
| UNIV TURIN | 5 | 0.166 |
| UNIV POMPEU FABRA | 5 | 0.166 |
| UNIV PITTSBURGH | 5 | 0.166 |
| UNIV PARIS 05 | 5 | 0.166 |
| UNIV NAPLES FEDERICO II | 5 | 0.166 |
| UNIV MED CTR UTRECHT | 5 | 0.166 |
| UNIV GRONINGEN | 5 | 0.166 |
| UNIV GENEVA | 5 | 0.166 |
| UNIV DUSSELDORF | 5 | 0.166 |
| UNIV CINCINNATI | 5 | 0.166 |
| UNIV CALIF IRVINE | 5 | 0.166 |
| TOKYO MED DENT UNIV | 5 | 0.166 |
| TARBIAT MODARES UNIV | 5 | 0.166 |
| SHANXI MED UNIV | 5 | 0.166 |
| SAITAMA MED UNIV | 5 | 0.166 |
| ROCKEFELLER UNIV | 5 | 0.166 |
| PENN STATE UNIV | 5 | 0.166 |
| NYU | 5 | 0.166 |
| NANTONG TUMOR HOSP | 5 | 0.166 |
| NANKAI UNIV | 5 | 0.166 |
| NANJING UNIV CHINESE MED | 5 | 0.166 |
| NANJING AGR UNIV | 5 | 0.166 |
| MINIST PUBL HLTH | 5 | 0.166 |
| KYOTO UNIV | 5 | 0.166 |
| KINKI UNIV | 5 | 0.166 |
| JINING 1 PEOPLES HOSP | 5 | 0.166 |
| JIANGNAN UNIV | 5 | 0.166 |
| HUZHOU CENT HOSP | 5 | 0.166 |
| HEIDELBERG UNIV | 5 | 0.166 |
| FRED HUTCHINSON CANC RES CTR | 5 | 0.166 |
| ERASMUS MC | 5 | 0.166 |
| CLEVELAND CLIN | 5 | 0.166 |
| CINCINNATI CHILDRENS HOSP MED CTR | 5 | 0.166 |
| CHINA UNIV MIN TECHNOL | 5 | 0.166 |
| BROAD INST HARVARD MIT | 5 | 0.166 |
| BRIGHAM WOMENS HOSP | 5 | 0.166 |
| BOSTON CHILDRENS HOSP | 5 | 0.166 |
| BELLVITGE BIOMED RES INST IDIBELL | 5 | 0.166 |
| AUSTRIAN ACAD SCI | 5 | 0.166 |
| ACAD SINICA | 5 | 0.166 |
| ZHEJIANG SCI TECH UNIV | 4 | 0.133 |
| XINXIANG CENT HOSP | 4 | 0.133 |
| WELLCOME TRUST SANGER INST | 4 | 0.133 |
| WASHINGTON STATE UNIV | 4 | 0.133 |
| VET AFFAIRS PALO ALTO HEALTHCARE SYST | 4 | 0.133 |
| UNIV ZURICH | 4 | 0.133 |
| UNIV TEXAS AUSTIN | 4 | 0.133 |
| UNIV TEXAS ARLINGTON | 4 | 0.133 |
| UNIV TEHRAN MED SCI | 4 | 0.133 |
| UNIV SOUTHERN CALIF | 4 | 0.133 |
| UNIV S FLORIDA | 4 | 0.133 |
| UNIV REGENSBURG | 4 | 0.133 |
| UNIV PIEMONTE ORIENTALE | 4 | 0.133 |
| UNIV PARIS DIDEROT | 4 | 0.133 |
| UNIV NEW SOUTH WALES | 4 | 0.133 |
| UNIV NACL AUTONOMA MEXICO | 4 | 0.133 |
| UNIV MISSOURI | 4 | 0.133 |
| UNIV LIBRE BRUXELLES | 4 | 0.133 |
| UNIV LAVAL | 4 | 0.133 |
| UNIV HAWAII | 4 | 0.133 |
| UNIV GEORGIA | 4 | 0.133 |
| UNIV CALGARY | 4 | 0.133 |
| UNIV BIRMINGHAM | 4 | 0.133 |
| TOHOKU UNIV | 4 | 0.133 |
| THOMAS JEFFERSON UNIV | 4 | 0.133 |
| TEXAS A M UNIV | 4 | 0.133 |
| SYDNEY CHILDRENS HOSP | 4 | 0.133 |
| SUZHOU UNIV | 4 | 0.133 |
| SUNY STONY BROOK | 4 | 0.133 |
| SUBEI PEOPLES HOSP | 4 | 0.133 |
| ST JUDE CHILDRENS RES HOSP | 4 | 0.133 |
| SOUTHWEST MED UNIV | 4 | 0.133 |
| SO ILLINOIS UNIV | 4 | 0.133 |
| SHENZHEN PKU HKUST MED CTR | 4 | 0.133 |
| SHANDONG ACAD MED SCI | 4 | 0.133 |
| OSAKA UNIV | 4 | 0.133 |
| NIAID | 4 | 0.133 |
| NHGRI | 4 | 0.133 |
| NATL YANG MING UNIV | 4 | 0.133 |
| MT SINAI SCH MED | 4 | 0.133 |
| MONASH UNIV | 4 | 0.133 |
| MED UNIV GRAZ | 4 | 0.133 |
| KAOHSIUNG MED UNIV HOSP | 4 | 0.133 |
| KAOHSIUNG MED UNIV | 4 | 0.133 |
| JINING MED UNIV | 4 | 0.133 |
| JIANGSU PROV CTR DIS CONTROL PREVENT | 4 | 0.133 |
| IST SCI SAN RAFFAELE | 4 | 0.133 |
| IST ITALIANO TECNOL | 4 | 0.133 |
| INST HOSP MAR INVEST MED IMIM | 4 | 0.133 |
| INNER MONGOLIA MED UNIV | 4 | 0.133 |
| HENAN PROV PEOPLES HOSP | 4 | 0.133 |
| HEILONGJIANG ACAD MED SCI | 4 | 0.133 |
| HAMAMATSU UNIV SCH MED | 4 | 0.133 |
| GERMAN CANC CONSORTIUM DKTK | 4 | 0.133 |
| FUJIAN AGR FORESTRY UNIV | 4 | 0.133 |
| FLORIDA STATE UNIV | 4 | 0.133 |
| FIRST PEOPLES HOSP YUNNAN PROV | 4 | 0.133 |
| ECOLE POLYTECH FED LAUSANNE | 4 | 0.133 |
| DKFZ ZMBH ALLIANCE | 4 | 0.133 |
| CTR GENOM REGULAT CRG | 4 | 0.133 |
| CTR GENOM REGULAT | 4 | 0.133 |
| CSIR | 4 | 0.133 |
| CRG | 4 | 0.133 |
| COLORADO STATE UNIV | 4 | 0.133 |
| CHINESE PLA | 4 | 0.133 |
| CHINESE NATL HUMAN GENOME CTR SHANGHAI | 4 | 0.133 |
| CHINA PHARMACEUT UNIV | 4 | 0.133 |
| CHILDRENS CANC INST AUSTRALIA MED RES | 4 | 0.133 |
| CHANG GUNG UNIV | 4 | 0.133 |
| CHANG GUNG MEM HOSP | 4 | 0.133 |
| CANGZHOU CENT HOSP | 4 | 0.133 |
| BROAD INST | 4 | 0.133 |
| BEIJING UNIV CHINESE MED | 4 | 0.133 |
| BARCELONA INST SCI TECHNOL | 4 | 0.133 |
| BAR ILAN UNIV | 4 | 0.133 |
| ADAM MICKIEWICZ UNIV | 4 | 0.133 |
| ZOUCHENG PEOPLES HOSP | 3 | 0.1 |
| ZHEJIANG PROV CANC HOSP | 3 | 0.1 |
| XUZHOU CENT HOSP | 3 | 0.1 |
| XIAN UNIV TECHNOL | 3 | 0.1 |
| WEIFANG PEOPLES HOSP | 3 | 0.1 |
| USDA ARS | 3 | 0.1 |
| US EPA | 3 | 0.1 |
| UNIV ZURICH HOSP | 3 | 0.1 |
| UNIV WURZBURG | 3 | 0.1 |
| UNIV TEXAS DALLAS | 3 | 0.1 |
| UNIV SOUTHERN DENMARK | 3 | 0.1 |
| UNIV SHERBROOKE | 3 | 0.1 |
| UNIV RENNES 1 | 3 | 0.1 |
| UNIV PARIS 11 | 3 | 0.1 |
| UNIV OSLO | 3 | 0.1 |
| UNIV NEWCASTLE | 3 | 0.1 |
| UNIV NEVADA | 3 | 0.1 |
| UNIV MELBOURNE | 3 | 0.1 |
| UNIV MANCHESTER | 3 | 0.1 |
| UNIV LJUBLJANA | 3 | 0.1 |
| UNIV LIVERPOOL | 3 | 0.1 |
| UNIV LEIPZIG | 3 | 0.1 |
| UNIV KIEL | 3 | 0.1 |
| UNIV IOWA | 3 | 0.1 |
| UNIV GLASGOW | 3 | 0.1 |
| UNIV DUNDEE | 3 | 0.1 |
| UNIV DUISBURG ESSEN | 3 | 0.1 |
| UNIV DUBLIN TRINITY COLL | 3 | 0.1 |
| UNIV CALIF RIVERSIDE | 3 | 0.1 |
| UNIV BOLOGNA | 3 | 0.1 |
| UNIV BASEL | 3 | 0.1 |
| UNIV AUCKLAND | 3 | 0.1 |
| UNIV AMSTERDAM | 3 | 0.1 |
| UCSF | 3 | 0.1 |
| TIANJIN MED UNIV GEN HOSP | 3 | 0.1 |
| TEXAS CHILDRENS HOSP | 3 | 0.1 |
| TEXAS A M HLTH SCI CTR | 3 | 0.1 |
| TECH UNIV MUNICH | 3 | 0.1 |
| TECH UNIV DRESDEN | 3 | 0.1 |
| TAIXING PEOPLES HOSP | 3 | 0.1 |
| TAIPEI MED UNIV | 3 | 0.1 |
| SYST BIOSCI | 3 | 0.1 |
| SWISS FED INST TECHNOL | 3 | 0.1 |
| ST LAURENT INST | 3 | 0.1 |
| SIMON FRASER UNIV | 3 | 0.1 |
| SICHUAN PROV PEOPLES HOSP | 3 | 0.1 |
| SICHUAN AGR UNIV | 3 | 0.1 |
| SHANGHAITECH UNIV | 3 | 0.1 |
| SHANDONG CANC HOSP INST | 3 | 0.1 |
| SHAHID BEHESHTI UNIV MED SCI | 3 | 0.1 |
| SAPPORO MED UNIV | 3 | 0.1 |
| RUTGERS STATE UNIV | 3 | 0.1 |
| RUSSIAN ACAD SCI | 3 | 0.1 |
| ROYAL PRINCE ALFRED HOSP | 3 | 0.1 |
| RNCB | 3 | 0.1 |
| RIKEN CTR LIFE SCI TECHNOL | 3 | 0.1 |
| RIKEN ADV RES INST | 3 | 0.1 |
| QUEENSLAND UNIV TECHNOL | 3 | 0.1 |
| PURDUE UNIV | 3 | 0.1 |
| PUMC | 3 | 0.1 |
| PLA 161 CTR HOSP | 3 | 0.1 |
| OSPED MAGGIORE POLICLIN | 3 | 0.1 |
| OREGON STATE UNIV | 3 | 0.1 |
| ODENSE UNIV HOSP | 3 | 0.1 |
| NORWEGIAN UNIV SCI TECHNOL | 3 | 0.1 |
| NORTH CHINA UNIV SCI TECHNOL | 3 | 0.1 |
| NINGBO YINZHOU SECOND HOSP | 3 | 0.1 |
| NINGBO KANGNING HOSP | 3 | 0.1 |
| NINGBO 2 HOSP | 3 | 0.1 |
| NIH | 3 | 0.1 |
| NATL HLTH RES INST | 3 | 0.1 |
| NATL CLIN RES CTR CANC | 3 | 0.1 |
| MED UNIV VIENNA | 3 | 0.1 |
| MAX PLANCK INST MOL CELL BIOL GENET | 3 | 0.1 |
| MAX PLANCK INST HEART LUNG RES | 3 | 0.1 |
| MAX DELBRUCK CTR MOL MED | 3 | 0.1 |
| MASARYK UNIV | 3 | 0.1 |
| LUND UNIV | 3 | 0.1 |
| LOUISIANA STATE UNIV | 3 | 0.1 |
| LEIDEN UNIV | 3 | 0.1 |
| KYOTO PREFECTURAL UNIV MED | 3 | 0.1 |
| KOREA UNIV | 3 | 0.1 |
| KING SAUD UNIV | 3 | 0.1 |
| KING ABDULLAH UNIV SCI TECHNOL | 3 | 0.1 |
| KEIO UNIV | 3 | 0.1 |
| ISIS PHARMACEUT INC | 3 | 0.1 |
| INDIANA UNIV SCH MED | 3 | 0.1 |
| INDIANA UNIV | 3 | 0.1 |
| IMPERIAL COLL | 3 | 0.1 |
| ILLUMINA INC | 3 | 0.1 |
| HUBEI UNIV MED | 3 | 0.1 |
| HUAQIAO UNIV | 3 | 0.1 |
| HEILONGJIANG UNIV CHINESE MED | 3 | 0.1 |
| HARVARD TH CHAN SCH PUBL HLTH | 3 | 0.1 |
| HARBIN MED UNIV DAQING | 3 | 0.1 |
| HANGZHOU CANC HOSP | 3 | 0.1 |
| GOLESTAN UNIV MED SCI | 3 | 0.1 |
| GOETHE UNIV | 3 | 0.1 |
| GEORGIA REGENTS UNIV | 3 | 0.1 |
| GEORGE WASHINGTON UNIV | 3 | 0.1 |
| FREE UNIV BERLIN | 3 | 0.1 |
| FOX CHASE CANC CTR | 3 | 0.1 |
| FIFTH HOSP WUHAN | 3 | 0.1 |
| EUNICE KENNEDY SHRIVER NATL INST CHILD HLTH HUM | 3 | 0.1 |
| DUKE NUS GRAD MED SCH | 3 | 0.1 |
| CUNY HUNTER COLL | 3 | 0.1 |
| CTR HOSP | 3 | 0.1 |
| CSIR IGIB | 3 | 0.1 |
| COLLABORAT INNOVAT CTR CANC MED | 3 | 0.1 |
| CHINESE PLA MED SCH | 3 | 0.1 |
| CHINESE NATL HUMAN GENOME CENTERAT SHANGHAI | 3 | 0.1 |
| CHINESE ALLIANCE TRANSLAT MED MATERNAL CHILDREN | 3 | 0.1 |
| CHINA NATL CLIN RES CTR NEUROL DIS | 3 | 0.1 |
| CHINA JAPAN FRIENDSHIP HOSP | 3 | 0.1 |
| CHARITE | 3 | 0.1 |
| CHANGSHA CENT HOSP | 3 | 0.1 |
| CENT HOSP TAIAN | 3 | 0.1 |
| CEDARS SINAI MED CTR | 3 | 0.1 |
| BROAD INST MASSACHUSETTS INST TECHNOL HARVARD | 3 | 0.1 |
| BIST | 3 | 0.1 |
| BINZHOU MED UNIV HOSP | 3 | 0.1 |
| BEIJING UNION MED COLL HOSP | 3 | 0.1 |
| BEIHUA UNIV | 3 | 0.1 |
| AIN SHAMS UNIV | 3 | 0.1 |
| AGCY SCI TECHNOL RES | 3 | 0.1 |
| 161 HOSP PLA | 3 | 0.1 |
| ZYMO RES CORP | 2 | 0.066 |
| ZUNYI MED COLL | 2 | 0.066 |
| ZHUMADIAN CENT HOSP | 2 | 0.066 |
| ZHEJIANG HOSP | 2 | 0.066 |
| YIXING PEOPLES HOSP | 2 | 0.066 |
| YISHUI CENT HOSP | 2 | 0.066 |
| YESHIVA UNIV ALBERT EINSTEIN COLL MED | 2 | 0.066 |
| YANCHENG HLTH VOCAT TECH COLL | 2 | 0.066 |
| XINJIANG KEY LAB CARDIOVASC DIS RES | 2 | 0.066 |
| XIANYANG CENT HOSP | 2 | 0.066 |
| XIAN MED UNIV | 2 | 0.066 |
| WUHAN UNIV SCI TECHNOL | 2 | 0.066 |
| WONKWANG UNIV | 2 | 0.066 |
| WEIFANG TRADIT CHINESE HOSP | 2 | 0.066 |
| WASEDA UNIV | 2 | 0.066 |
| WAKE FOREST UNIV | 2 | 0.066 |
| WAKE FOREST SCH MED | 2 | 0.066 |
| VIRGINIA TECH | 2 | 0.066 |
| VICTOR CHANG CARDIAC RES INST | 2 | 0.066 |
| VANCOUVER GEN HOSP | 2 | 0.066 |
| VA PALO ALTO HLTH CARE SYST | 2 | 0.066 |
| UZ LEUVEN | 2 | 0.066 |
| UT MD ANDERSON CANC CTR | 2 | 0.066 |
| UNIV WARSAW | 2 | 0.066 |
| UNIV VITA SALUTE SAN RAFFAELE | 2 | 0.066 |
| UNIV VIRGINIA HLTH SYST | 2 | 0.066 |
| UNIV VIENNA | 2 | 0.066 |
| UNIV ULM | 2 | 0.066 |
| UNIV TURKU | 2 | 0.066 |
| UNIV TEXAS HOUSTON | 2 | 0.066 |
| UNIV TAMPERE | 2 | 0.066 |
| UNIV SOUTHAMPTON | 2 | 0.066 |
| UNIV SHEFFIELD | 2 | 0.066 |
| UNIV S ALABAMA | 2 | 0.066 |
| UNIV PORTO | 2 | 0.066 |
| UNIV POMPEU FABRA UPF | 2 | 0.066 |
| UNIV POLITECN VALENCIA | 2 | 0.066 |
| UNIV OTTAWA | 2 | 0.066 |
| UNIV NOTTINGHAM | 2 | 0.066 |
| UNIV NAPLES 2 | 2 | 0.066 |
| UNIV MUNSTER | 2 | 0.066 |
| UNIV MONCTON | 2 | 0.066 |
| UNIV MICHIGAN HLTH SYST | 2 | 0.066 |
| UNIV MED PHARM IULIU HATIEGANU | 2 | 0.066 |
| UNIV MED CTR HAMBURG EPPENDORF | 2 | 0.066 |
| UNIV LILLE | 2 | 0.066 |
| UNIV LAUSANNE HOSP | 2 | 0.066 |
| UNIV JENA | 2 | 0.066 |
| UNIV HOSP GENEVA | 2 | 0.066 |
| UNIV HOSP BRNO | 2 | 0.066 |
| UNIV HOSP BONN | 2 | 0.066 |
| UNIV HOSP BERN | 2 | 0.066 |
| UNIV HOSP | 2 | 0.066 |
| UNIV HAWAII MANOA | 2 | 0.066 |
| UNIV HALLE WITTENBERG | 2 | 0.066 |
| UNIV GOTTINGEN | 2 | 0.066 |
| UNIV FLORIDA | 2 | 0.066 |
| UNIV FED FLUMINENSE | 2 | 0.066 |
| UNIV CRETE | 2 | 0.066 |
| UNIV CONCEPCION | 2 | 0.066 |
| UNIV COLOGNE | 2 | 0.066 |
| UNIV CALIF SANTA CRUZ | 2 | 0.066 |
| UNIV BRESCIA | 2 | 0.066 |
| UNIV BERGEN | 2 | 0.066 |
| UNIV BASQUE COUNTRY UPV EHU | 2 | 0.066 |
| UNIV BASEL HOSP | 2 | 0.066 |
| UNIV AUTONOMA BARCELONA | 2 | 0.066 |
| UNIV ATHENS | 2 | 0.066 |
| UNIV ARKANSAS MED SCI | 2 | 0.066 |
| UNIV ARIZONA | 2 | 0.066 |
| UFZ HELMHOLTZ CTR ENVIRONM RES | 2 | 0.066 |
| TULANE UNIV | 2 | 0.066 |
| TRINITY COLL DUBLIN | 2 | 0.066 |
| TIANJIN UNION MED CTR | 2 | 0.066 |
| TIANJIN MED UNIV CANC INST HOSP | 2 | 0.066 |
| TIANJIN KEY LAB INJURIES VARIAT REGENERAT NERVO | 2 | 0.066 |
| TIANJIN CHILDRENS HOSP | 2 | 0.066 |
| TELETHON INST GENET MED TIGEM | 2 | 0.066 |
| TECHNION ISRAEL INST TECHNOL | 2 | 0.066 |
| TECH UNIV APPL SCI WILDAU | 2 | 0.066 |
| TANGSHAN GONGREN HOSP | 2 | 0.066 |
| TAIZHOU PEOPLES HOSP | 2 | 0.066 |
| TAIZHOU MUNICIPAL HOSP | 2 | 0.066 |
| TAIZHOU MATERN CHILD HLTH CARE HOSP | 2 | 0.066 |
| TAISHAN MED UNIV | 2 | 0.066 |
| SWISS INST BIOINFORMAT | 2 | 0.066 |
| SUZHOU UNIV SCI TECHNOL | 2 | 0.066 |
| STOWERS INST MED RES | 2 | 0.066 |
| STN ZOOL ANTON DOHRN | 2 | 0.066 |
| ST MICHAELS HOSP | 2 | 0.066 |
| ST LUKES EPISCOPAL HOSP | 2 | 0.066 |
| ST JAMES HOSP | 2 | 0.066 |
| SRI AUROBINDO SEVA KENDRA | 2 | 0.066 |
| SO MED UNIV | 2 | 0.066 |
| SISSA | 2 | 0.066 |
| SHENZHEN SECOND PEOPLES HOSP | 2 | 0.066 |
| SHANXI KEY LAB OTOLARYNGOL HEAD NECK CANC | 2 | 0.066 |
| SHANGHAI UNIV | 2 | 0.066 |
| SHANGHAI OCEAN UNIV | 2 | 0.066 |
| SHANGHAI KEY LAB REPROD MED | 2 | 0.066 |
| SHANGHAI KEY LAB PANCREAT DIS | 2 | 0.066 |
| SHANGHAI KEY LAB EMBRYO REPROD ENGN | 2 | 0.066 |
| SHANGHAI CTR BIOINFORMAT TECHNOL | 2 | 0.066 |
| SHANDONG UNIV TRADIT CHINESE MED | 2 | 0.066 |
| SHANDONG PROV TRADIT CHINESE MED HOSP | 2 | 0.066 |
| SHANDONG PROV HOSP | 2 | 0.066 |
| SHANDONG JIAOTONG UNIV | 2 | 0.066 |
| SEOUL NATL UNIV HOSP | 2 | 0.066 |
| SECOND PEOPLES HOSP LIANYUNGANG | 2 | 0.066 |
| SECOND HOSP LONGYAN CITY | 2 | 0.066 |
| SAROJ GUPTA CANC CTR RES INST | 2 | 0.066 |
| SALK INST BIOL STUDIES | 2 | 0.066 |
| RUHR UNIV BOCHUM | 2 | 0.066 |
| RUDJER BOSKOVIC INST | 2 | 0.066 |
| ROYAL NETHERLANDS ACAD ARTS SCI | 2 | 0.066 |
| ROYAL N SHORE HOSP | 2 | 0.066 |
| ROSWELL PK CANC INST | 2 | 0.066 |
| REGENERON PHARMACEUT INC | 2 | 0.066 |
| RADY CHILDRENS HOSP SAN DIEGO | 2 | 0.066 |
| RADBOUD UNIV NIJMEGEN | 2 | 0.066 |
| PROGRAM EPITHELIAL BIOL | 2 | 0.066 |
| PONTIFICIA UNIV JAVERIANA | 2 | 0.066 |
| PINGAN HLTH CLOUD CO LTD CHINA | 2 | 0.066 |
| PEOPLES LIBERAT ARMY | 2 | 0.066 |
| PEOPLES HOSP ZHENGZHOU | 2 | 0.066 |
| PEOPLES HOSP XUANCHENG CITY | 2 | 0.066 |
| PEOPLES HOSP CHIZHOU | 2 | 0.066 |
| PEKING UNIV CANC HOSP INST | 2 | 0.066 |
| OTOLARYNGOL MAJOR DIS RES KEY LAB HUNAN PROV | 2 | 0.066 |
| OSPED SAN RAFFAELE | 2 | 0.066 |
| OSLO UNIV HOSP | 2 | 0.066 |
| OPEN UNIV | 2 | 0.066 |
| ONTARIO INST CANC RES | 2 | 0.066 |
| ONCOL INST ION CHIRICUTA | 2 | 0.066 |
| ONCOL HOSP JILIN PROV | 2 | 0.066 |
| NOTTINGHAM TRENT UNIV | 2 | 0.066 |
| NORTHWESTERN POLYTECH UNIV | 2 | 0.066 |
| NINGXIA MED UNIV | 2 | 0.066 |
| NIDDK | 2 | 0.066 |
| NICHHD | 2 | 0.066 |
| NEW YORK PRESBYTERIAN HOSP | 2 | 0.066 |
| NEUROSCI RES AUSTRALIA | 2 | 0.066 |
| NETHERLANDS CANC INST | 2 | 0.066 |
| NETAJI SUBHAS SANAT | 2 | 0.066 |
| NE NORMAL UNIV | 2 | 0.066 |
| NATL TAIWAN UNIV HOSP | 2 | 0.066 |
| NATL SUN YAT SEN UNIV | 2 | 0.066 |
| NATL INST SCI TECHNOL STEM CELL CELL THERAPY | 2 | 0.066 |
| NATL INST GENET ENGN BIOTECHNOL | 2 | 0.066 |
| NATL CHIAO TUNG UNIV | 2 | 0.066 |
| NATL CANC INST | 2 | 0.066 |
| NATIONWIDE CHILDRENS HOSP | 2 | 0.066 |
| NANJING NORMAL UNIV | 2 | 0.066 |
| NANJING CHEST HOSP | 2 | 0.066 |
| N CAROLINA STATE UNIV | 2 | 0.066 |
| MINIST HLTH CHINA | 2 | 0.066 |
| MILTON S HERSHEY MED CTR | 2 | 0.066 |
| MIL MED ACAD | 2 | 0.066 |
| MIDDLE TENNESSEE STATE UNIV | 2 | 0.066 |
| MICHIGAN CTR TRANSLAT PATHOL | 2 | 0.066 |
| METRON GLOBAL HLTH INITIAT | 2 | 0.066 |
| MELBOURNE CTR CLIN SCI | 2 | 0.066 |
| MED COLL WISCONSIN | 2 | 0.066 |
| MAX PLANCK INST IMMUNOBIOL EPIGENET | 2 | 0.066 |
| MAX DELBRUCK CTR MOL MED MDC | 2 | 0.066 |
| MASARYK MEM CANC INST | 2 | 0.066 |
| MARCH DIMES GLOBAL NETWORK MATERNAL INFANT HLTH | 2 | 0.066 |
| LUZHOU MED COLL | 2 | 0.066 |
| LUXEMBOURG INST HLTH | 2 | 0.066 |
| LONGYAN HUMAN HOSP | 2 | 0.066 |
| LIAOCHENG PEOPLES HOSP | 2 | 0.066 |
| LIANYUNGANG MATERNAL CHILDRENS HOSP | 2 | 0.066 |
| LAWRENCE BERKELEY NATL LAB | 2 | 0.066 |
| LA TROBE UNIV | 2 | 0.066 |
| KYUNGPOOK NATL UNIV | 2 | 0.066 |
| KUMAMOTO UNIV | 2 | 0.066 |
| KULEUVEN | 2 | 0.066 |
| KNAPPSCHAFTSKRANKENHAUS UNIV BOCHUM | 2 | 0.066 |
| KINGS COLL LONDON | 2 | 0.066 |
| KEY LAB CANC PREVENT THERAPY | 2 | 0.066 |
| KEY LAB CANC IMMUNOL BIOTHERAPY | 2 | 0.066 |
| KAOHSIUNG MUNICIPAL TATUNG HOSP | 2 | 0.066 |
| KAIFENG MATERN HOSP | 2 | 0.066 |
| JOHNS HOPKINS SCH MED | 2 | 0.066 |
| JINLING HOSP | 2 | 0.066 |
| JIANGSU PROV HOSP | 2 | 0.066 |
| JIANGSU PROV CTR DIS PREVENT CONTROL | 2 | 0.066 |
| JIANGSU PROV ACAD CLIN MED | 2 | 0.066 |
| JIANGSU CHINESE MED HOSP | 2 | 0.066 |
| JIANGSU ACAD AGR SCI | 2 | 0.066 |
| IWATE MED UNIV | 2 | 0.066 |
| ITALIAN INST TECHNOL | 2 | 0.066 |
| IRCCS BAMBINO GESU CHILDRENS HOSP | 2 | 0.066 |
| IRCCS | 2 | 0.066 |
| IONIS PHARMACEUT INC | 2 | 0.066 |
| IONIS PHARMACEUT | 2 | 0.066 |
| INT AGCY RES CANC | 2 | 0.066 |
| INST ONCOL | 2 | 0.066 |
| INST NACL CIENCIA TECNOL ONCOGEN | 2 | 0.066 |
| INST GUSTAVE ROUSSY | 2 | 0.066 |
| INST CURIE | 2 | 0.066 |
| INST BIOTECHNOL | 2 | 0.066 |
| INRA | 2 | 0.066 |
| INNER MONGOLIA PEOPLES HOSP | 2 | 0.066 |
| INDIAN ASSOC CULTIVAT SCI | 2 | 0.066 |
| ILS | 2 | 0.066 |
| HUNTER COLL HIGH SCH | 2 | 0.066 |
| HUNAN NORMAL UNIV | 2 | 0.066 |
| HUNAN KEY LAB PHARMACOGENET | 2 | 0.066 |
| HUMANITAS CLIN RES CTR | 2 | 0.066 |
| HUAIAN SECOND HOSP | 2 | 0.066 |
| HONG KONG POLYTECH UNIV | 2 | 0.066 |
| HLTH BUR ZHENGZHOU | 2 | 0.066 |
| HIROSHIMA UNIV | 2 | 0.066 |
| HERLEV UNIV HOSP | 2 | 0.066 |
| HENRY FORD HLTH SYST | 2 | 0.066 |
| HENAN UNIV TCM | 2 | 0.066 |
| HENAN NORMAL UNIV | 2 | 0.066 |
| HENAN KEY LAB TUMOR EPIDEMIOL | 2 | 0.066 |
| HELMHOLTZ CTR INFECT RES | 2 | 0.066 |
| HEBEI UNIV TECHNOL | 2 | 0.066 |
| HEBEI UNIV | 2 | 0.066 |
| HEBEI UNITED UNIV | 2 | 0.066 |
| HEBEI GEN HOSP | 2 | 0.066 |
| GYANXET | 2 | 0.066 |
| GUILIN MED UNIV | 2 | 0.066 |
| GUANGZHOU MIL COMMAND CHINESE PEOPLES LIBERAT ARM | 2 | 0.066 |
| GUANGXI NORMAL UNIV | 2 | 0.066 |
| GUANGDONG PHARMACEUT UNIV | 2 | 0.066 |
| GHENT UNIV HOSP | 2 | 0.066 |
| GERMAN CTR CARDIOVASC RES DZHK | 2 | 0.066 |
| GANSU WUWEI TUMOR HOSP | 2 | 0.066 |
| FUZHOU GEN HOSP | 2 | 0.066 |
| FUNDACAO OSWALDO CRUZ | 2 | 0.066 |
| FUJIAN UNIV TRADIT CHINESE MED | 2 | 0.066 |
| FRAUNHOFER INST CELL THERAPY IMMUNOL IZI | 2 | 0.066 |
| FIRST PEOPLES HOSP LIANYUNGANG | 2 | 0.066 |
| FIRST PEOPLES HOSP FOSHAN | 2 | 0.066 |
| FAC MED | 2 | 0.066 |
| EWHA WOMANS UNIV | 2 | 0.066 |
| EUROPEAN MOL BIOL LAB | 2 | 0.066 |
| EASTERN HEPATOBILIARY SURG HOSP | 2 | 0.066 |
| E CAROLINA UNIV | 2 | 0.066 |
| DGIST | 2 | 0.066 |
| DEPT NEUROL SURG | 2 | 0.066 |
| DEPT COMPUTAT MED BIOINFORMAT | 2 | 0.066 |
| DAQING OIL FIELD GEN HOSP | 2 | 0.066 |
| DALIAN UNIV | 2 | 0.066 |
| CTR RECH PUBL SANTE | 2 | 0.066 |
| CTR CELL BASED THERAPY | 2 | 0.066 |
| CSIRO PLANT IND | 2 | 0.066 |
| CSIC | 2 | 0.066 |
| CREIGHTON UNIV | 2 | 0.066 |
| COLLABORAT INNOVAT CTR CANC CHEMOPREVENT | 2 | 0.066 |
| CMST DEV CO LTD | 2 | 0.066 |
| CLIN CTR NANJING RESP DIS IMAGING | 2 | 0.066 |
| CIPF | 2 | 0.066 |
| CHUNGNAM NATL UNIV | 2 | 0.066 |
| CHONGQING INST CARDIOL | 2 | 0.066 |
| CHINA ACAD CHINESE MED SCI | 2 | 0.066 |
| CHILDRENS HOSP LOS ANGELES | 2 | 0.066 |
| CHILDRENS HOSP BOSTON | 2 | 0.066 |
| CHANGSHU 2 PEOPLES HOSP | 2 | 0.066 |
| CHANGHAI HOSP | 2 | 0.066 |
| CHALMERS | 2 | 0.066 |
| CHAIM SHEBA MED CTR | 2 | 0.066 |
| CHA UNIV | 2 | 0.066 |
| CAPITAL UNIV MED SCI | 2 | 0.066 |
| CANC RES UK | 2 | 0.066 |
| BUR ANIM HUSB BIYANG CTY | 2 | 0.066 |
| BROAD INST HARVARD | 2 | 0.066 |
| BOSTON UNIV | 2 | 0.066 |
| BIOSEARCH TECHNOL INC | 2 | 0.066 |
| BINZHOU MED COLL | 2 | 0.066 |
| BGI SHENZHEN | 2 | 0.066 |
| BENGBU MED COLL | 2 | 0.066 |
| BANARAS HINDU UNIV | 2 | 0.066 |
| BALTIMORE VET AFFAIRS MED CTR | 2 | 0.066 |
| AZIENDA OSPED UNIV | 2 | 0.066 |
| AUTONOMOUS UNIV MADRID | 2 | 0.066 |
| AUSTRALIAN NATL UNIV | 2 | 0.066 |
| ASTRAZENECA R D | 2 | 0.066 |
| ARS | 2 | 0.066 |
| ANIM HLTH SUPERVIS HENAN PROV | 2 | 0.066 |
| ANHUI UNIV | 2 | 0.066 |
| ALLEN INST BRAIN SCI | 2 | 0.066 |
| AGILENT TECHNOL | 2 | 0.066 |
| AFFILIATED HOSP NANTONG UNIV | 2 | 0.066 |
| ACAD MIL MED SCI | 2 | 0.066 |
| AARHUS UNIV | 2 | 0.066 |
| AALBORG UNIV HOSP | 2 | 0.066 |
| 253 HOSP PLA | 2 | 0.066 |
| 181ST HOSP | 2 | 0.066 |
| 161TH HOSP PLA | 2 | 0.066 |
| ZOUCHENG HOSP TRADIT CHINESE MED | 1 | 0.033 |
| ZHUZHOU HOSP 1 | 1 | 0.033 |
| ZHUHAI PEOPLES HOSP | 1 | 0.033 |
| ZHONGSHAN HOSP | 1 | 0.033 |
| ZHONGGUANCUN OPEN LAB RES DEV NAT MED HLTH PR | 1 | 0.033 |
| ZHENJIANG EMERGENCY MED CTR | 1 | 0.033 |
| ZHENJIANG CHINESE MED UNIV | 1 | 0.033 |
| ZHENHAI MIDDLE SCH | 1 | 0.033 |
| ZHENGZHOU SIXTH PEOPLES HOSP | 1 | 0.033 |
| ZHENGZHOU PEOPLES HOSP | 1 | 0.033 |
| ZHENGZHOU CTR HOSP | 1 | 0.033 |
| ZHEJIANG PROV PEOPLES HOSP | 1 | 0.033 |
| ZHEJIANG PROV HOSP CHINESE TRADIT MED | 1 | 0.033 |
| ZHEJIANG OCEAN UNIV | 1 | 0.033 |
| ZHEJIANG KEY LAB DIAG TREATMENT TECHNOL THORAC | 1 | 0.033 |
| ZHEJIANG INST MICROBIOL | 1 | 0.033 |
| ZHEJIANG CHINESE MED UNIV | 1 | 0.033 |
| ZHEJIANG CANC RES INST | 1 | 0.033 |
| ZHEJIANG CANC CTR | 1 | 0.033 |
| ZETUP AG ST GALLEN | 1 | 0.033 |
| YUNNAN UNIV CHINESE TRADIT MED | 1 | 0.033 |
| YUNNAN PROV CTR DIS CONTROL PREVENT | 1 | 0.033 |
| YUNNAN INST EXPT DIAG | 1 | 0.033 |
| YUHUANGDING HOSP | 1 | 0.033 |
| YOUYANG ANIM HUSB BUR | 1 | 0.033 |
| YIXING TUMOR HOSP | 1 | 0.033 |
| YIWU CENT HOSP | 1 | 0.033 |
| YINZHOU PEOPLES HOSP | 1 | 0.033 |
| YINAN PEOPLES HOSP | 1 | 0.033 |
| YIJISHAN HOSP | 1 | 0.033 |
| YIDU CENT HOSP WEIFANG | 1 | 0.033 |
| YANTAISHAN HOSP | 1 | 0.033 |
| YANCHENG SECOND HOSP | 1 | 0.033 |
| YANCHENG MATERN CHILD HLTH CARE HOSP | 1 | 0.033 |
| YANCHENG HLTH VOCAT | 1 | 0.033 |
| YANCHENG CITY 1 PEOPLES HOSP | 1 | 0.033 |
| YANBIAN UNIV | 1 | 0.033 |
| YANAN UNIV | 1 | 0.033 |
| YANAN CITY PEOPLES HOSP | 1 | 0.033 |
| YAMAGUCHI UNIV | 1 | 0.033 |
| YALE SCH PUBL HLTH | 1 | 0.033 |
| XUZHOU CHILDRENS HOSP | 1 | 0.033 |
| XINXIANG MED COLL | 1 | 0.033 |
| XINJIANG AGR UNIV | 1 | 0.033 |
| XIANGTAN CENT HOSP | 1 | 0.033 |
| XIANGTAN BUR ANIM HUSB VET MED AQUAT PROD | 1 | 0.033 |
| XIAN YANG CENT HOSP | 1 | 0.033 |
| XIAN JIAOTONG LIVERPOOL UNIV | 1 | 0.033 |
| XIAN CHILDRENS HOSP | 1 | 0.033 |
| XIAMEN CTR DIS CONTROL PREVENT | 1 | 0.033 |
| WUXI MATERN CHILD HLTH HOSP | 1 | 0.033 |
| WUXI CITY HOSP CHINESE MED | 1 | 0.033 |
| WUHAN MED HEALTHCARE CTR WOMEN CHILDREN | 1 | 0.033 |
| WUHAN INST BIOL PROD | 1 | 0.033 |
| WUHAN HOSP PREVENT TREATMENT OCCUPAT DIS | 1 | 0.033 |
| WUHAN GEN HOSP GUANGZHOU MIL REG | 1 | 0.033 |
| WU JIANG FIRST PEOPLES HOSP | 1 | 0.033 |
| WOMENS CLIN OIZUMI GAKUEN | 1 | 0.033 |
| WOMEN CHILDRENS HOSP LINYI | 1 | 0.033 |
| WOMEN CHILDREN HLTH INST FUTIAN | 1 | 0.033 |
| WINSHIP CANC INST | 1 | 0.033 |
| WESTERN GEN HOSP | 1 | 0.033 |
| WENZHOU HEPING PLAST SURG HOSP | 1 | 0.033 |
| WENZHOU CENT HOSP | 1 | 0.033 |
| WELLCOME TRUST RES LABS | 1 | 0.033 |
| WELLCOME TRUST GENOME CAMPUS | 1 | 0.033 |
| WELLCOME TRUST DBT INDIA ALLIANCE | 1 | 0.033 |
| WEIS CTR RES | 1 | 0.033 |
| WEILL CORNELL GRAD SCH MED SCI | 1 | 0.033 |
| WEILL CORENLL MED COLL | 1 | 0.033 |
| WEIHAI MATERNAL CHILD HLTH HOSP | 1 | 0.033 |
| WEIFANG MED COLL | 1 | 0.033 |
| WASHINGTON STATE UNIV SPOKANE | 1 | 0.033 |
| WANBEI COAL ELECT GRP GEN HOSP | 1 | 0.033 |
| WALTER REED NATL MIL MED CTR | 1 | 0.033 |
| WAGENINGEN UNIV | 1 | 0.033 |
| WADSWORTH CTR | 1 | 0.033 |
| W VIRGINIA UNIV | 1 | 0.033 |
| VUB | 1 | 0.033 |
| VRIJE UNIV AMSTERDAM | 1 | 0.033 |
| VIRGINIA POLYTECH INST STATE UNIV | 1 | 0.033 |
| VIB KULEUVEN | 1 | 0.033 |
| VIB CTR BIOL DIS | 1 | 0.033 |
| VET AFFAIRS PALO ALTO HLTH CARE SYST | 1 | 0.033 |
| VET AFFAIRS MED CTR | 1 | 0.033 |
| VET AFFAIRS CONNECTICUT HEALTHCARE SYST | 1 | 0.033 |
| VERGE GENOM | 1 | 0.033 |
| VANDERBILT UNIV SCH MED | 1 | 0.033 |
| VANDERBILT HEART VASC INST | 1 | 0.033 |
| VANDERBILT DEPT MED | 1 | 0.033 |
| VANCOUVER PROSTATE CTR | 1 | 0.033 |
| VAN ANDEL RES INST | 1 | 0.033 |
| VA MED CTR | 1 | 0.033 |
| VA GREATER LOS ANGELES HEALTHCARE SYST | 1 | 0.033 |
| UT SOUTHWESTERN MED CTR | 1 | 0.033 |
| US FDA | 1 | 0.033 |
| UNSW AUSTRALIA | 1 | 0.033 |
| UNIV WESTERN ONTARIO | 1 | 0.033 |
| UNIV WATERLOO | 1 | 0.033 |
| UNIV VERONA | 1 | 0.033 |
| UNIV ULSAN | 1 | 0.033 |
| UNIV TSUKUBA | 1 | 0.033 |
| UNIV TRIESTE | 1 | 0.033 |
| UNIV TRENTO | 1 | 0.033 |
| UNIV TOULOUSE 3 | 1 | 0.033 |
| UNIV TOULOUSE | 1 | 0.033 |
| UNIV TOLEDO | 1 | 0.033 |
| UNIV THESSALY | 1 | 0.033 |
| UNIV TEXAS HLTH SCI CTR | 1 | 0.033 |
| UNIV TEXAS GRAD SCH BIOMED SCI HOUSTON | 1 | 0.033 |
| UNIV TEXAS GRAD SCH BIOMED SCI | 1 | 0.033 |
| UNIV TENNESSEE | 1 | 0.033 |
| UNIV TECHNOL SYDNEY | 1 | 0.033 |
| UNIV TECHNOL | 1 | 0.033 |
| UNIV TARTU | 1 | 0.033 |
| UNIV TABRIZ | 1 | 0.033 |
| UNIV SZEGED | 1 | 0.033 |
| UNIV SURREY | 1 | 0.033 |
| UNIV SUNSHINE COAST | 1 | 0.033 |
| UNIV SUD TOULON VAR | 1 | 0.033 |
| UNIV STIRLING | 1 | 0.033 |
| UNIV STELLENBOSCH | 1 | 0.033 |
| UNIV ST ANDREWS | 1 | 0.033 |
| UNIV SO MISSISSIPPI | 1 | 0.033 |
| UNIV SHENZHEN | 1 | 0.033 |
| UNIV SCI TECHNOL | 1 | 0.033 |
| UNIV SANTO AMARO | 1 | 0.033 |
| UNIV SANTIAGO CHILE | 1 | 0.033 |
| UNIV SAARLAND | 1 | 0.033 |
| UNIV S MANCHESTER HOSP | 1 | 0.033 |
| UNIV RUHUNA | 1 | 0.033 |
| UNIV ROSARIO | 1 | 0.033 |
| UNIV ROME LA SAPIENZA | 1 | 0.033 |
| UNIV ROMA TOR VERGATA | 1 | 0.033 |
| UNIV QUEENSLAND AUSTRALIA | 1 | 0.033 |
| UNIV PUNE | 1 | 0.033 |
| UNIV POLITECN CATALUNA | 1 | 0.033 |
| UNIV PALERMO | 1 | 0.033 |
| UNIV PADUA | 1 | 0.033 |
| UNIV OVIEDO | 1 | 0.033 |
| UNIV OTAGO | 1 | 0.033 |
| UNIV OREGON | 1 | 0.033 |
| UNIV OKLAHOMA HLTH SCI CTR | 1 | 0.033 |
| UNIV NORTH TEXAS HLTH SCI CTR FT WORTH | 1 | 0.033 |
| UNIV NORTH TEXAS HLTH SCI CTR | 1 | 0.033 |
| UNIV NORDLAND | 1 | 0.033 |
| UNIV NEW SOUTH WALES AUSTRALIA | 1 | 0.033 |
| UNIV NEW MEXICO | 1 | 0.033 |
| UNIV NANTES | 1 | 0.033 |
| UNIV NAMUR UNAMUR | 1 | 0.033 |
| UNIV NACL EDUC DISTANCIA | 1 | 0.033 |
| UNIV N TEXAS | 1 | 0.033 |
| UNIV MONTREAL | 1 | 0.033 |
| UNIV MONTPELLIER 2 | 1 | 0.033 |
| UNIV MONTPELLIER 1 | 1 | 0.033 |
| UNIV MODENA REGGIO EMILIA | 1 | 0.033 |
| UNIV MINHO | 1 | 0.033 |
| UNIV MIGUEL HERNANDEZ | 1 | 0.033 |
| UNIV MED PHARM I HATIEGANU | 1 | 0.033 |
| UNIV MED CTR ROTTERDAM | 1 | 0.033 |
| UNIV MED CTR NIJMEGEN | 1 | 0.033 |
| UNIV MED CTR HAMBURG | 1 | 0.033 |
| UNIV MED CTR | 1 | 0.033 |
| UNIV MANITOBA | 1 | 0.033 |
| UNIV MALAYA | 1 | 0.033 |
| UNIV LYON | 1 | 0.033 |
| UNIV LOUISVILLE | 1 | 0.033 |
| UNIV LILLE NORD FRANCE | 1 | 0.033 |
| UNIV LIBRE BRUSSELS | 1 | 0.033 |
| UNIV LEUVEN | 1 | 0.033 |
| UNIV LEICESTER | 1 | 0.033 |
| UNIV LEEDS | 1 | 0.033 |
| UNIV KANSAS | 1 | 0.033 |
| UNIV INNSBRUCK | 1 | 0.033 |
| UNIV ICELAND | 1 | 0.033 |
| UNIV HOSP SCHLESWIG HOLSTEIN | 1 | 0.033 |
| UNIV HOSP NORTH DURHAM | 1 | 0.033 |
| UNIV HOSP MUNSTER | 1 | 0.033 |
| UNIV HOSP MUENSTER | 1 | 0.033 |
| UNIV HOSP MORALES MESEGUER | 1 | 0.033 |
| UNIV HOSP LEUVEN | 1 | 0.033 |
| UNIV HOSP GIESSEN | 1 | 0.033 |
| UNIV HOSP G MARTINO | 1 | 0.033 |
| UNIV HOSP CASE MED CTR | 1 | 0.033 |
| UNIV HOSP 12 OCTUBRE | 1 | 0.033 |
| UNIV HLTH NETWORK | 1 | 0.033 |
| UNIV GUELPH | 1 | 0.033 |
| UNIV GRENOBLE 1 | 1 | 0.033 |
| UNIV GHENT VIB | 1 | 0.033 |
| UNIV FRANCOIS RABELAIS TOURS | 1 | 0.033 |
| UNIV FLORENCE | 1 | 0.033 |
| UNIV FED RIO GRANDE DO NORTE | 1 | 0.033 |
| UNIV EVRY VAL ESSONNE | 1 | 0.033 |
| UNIV ESTADUAL PAULISTA | 1 | 0.033 |
| UNIV ESTADUAL CAMPINAS | 1 | 0.033 |
| UNIV ESSEX | 1 | 0.033 |
| UNIV ERLANGEN NURNBERG | 1 | 0.033 |
| UNIV DURHAM | 1 | 0.033 |
| UNIV DESCARTES | 1 | 0.033 |
| UNIV CONSTANCE | 1 | 0.033 |
| UNIV CONNECTICUT HLTH CTR | 1 | 0.033 |
| UNIV COLORADO DENVER | 1 | 0.033 |
| UNIV COIMBRA | 1 | 0.033 |
| UNIV CLIN NAVARRA CUN | 1 | 0.033 |
| UNIV CITTA SALUTE | 1 | 0.033 |
| UNIV CHILDRENS HOSP | 1 | 0.033 |
| UNIV CATTOLICA | 1 | 0.033 |
| UNIV CATANIA | 1 | 0.033 |
| UNIV CALIF SANTA BARBARA | 1 | 0.033 |
| UNIV CADIZ | 1 | 0.033 |
| UNIV BRISTOL | 1 | 0.033 |
| UNIV BRASILIA | 1 | 0.033 |
| UNIV BRADFORD | 1 | 0.033 |
| UNIV BONN | 1 | 0.033 |
| UNIV BOLOGNA ALMA MATER | 1 | 0.033 |
| UNIV BERN | 1 | 0.033 |
| UNIV AUTONOMA MADRID | 1 | 0.033 |
| UNIV AUTONOMA AGR ANTONIO NARRO | 1 | 0.033 |
| UNIV ANDRES BELLO | 1 | 0.033 |
| UNIV ALBERTA | 1 | 0.033 |
| UNIV AIX MARSEILLE | 1 | 0.033 |
| UNIV ADELAIDE | 1 | 0.033 |
| UNIFORMED SERV UNIV HLTH SCI | 1 | 0.033 |
| UNICANCER | 1 | 0.033 |
| UNICAEN | 1 | 0.033 |
| UMEA UNIV | 1 | 0.033 |
| UIT NORWEGIAN ARCTIC UNIV | 1 | 0.033 |
| UCSD MED CTR | 1 | 0.033 |
| UCSC | 1 | 0.033 |
| UCM | 1 | 0.033 |
| UCLA PATH LAB MED | 1 | 0.033 |
| UCL INST OPHTHALMOL | 1 | 0.033 |
| UCL INST CHILD HLTH | 1 | 0.033 |
| UCL CANC INST | 1 | 0.033 |
| TZU CHI UNIV | 1 | 0.033 |
| TUSHAR PATELS LAB | 1 | 0.033 |
| TURKU UNIV HOSP | 1 | 0.033 |
| TUMOR HOSP JILIN PROV | 1 | 0.033 |
| TUMOR HOSP JILIN | 1 | 0.033 |
| TRANSSINE TECHNOL | 1 | 0.033 |
| TRANSLAT MED CTR NORTHERN CHINA | 1 | 0.033 |
| TRANSLAT LUNG RES CTR HEIDELBERG TLRC | 1 | 0.033 |
| TRANSLAT HLTH SCI TECHNOL INST | 1 | 0.033 |
| TRANSLAT GENOM RES INST | 1 | 0.033 |
| TRADIT CHINESE MED HOSP LESHAN | 1 | 0.033 |
| TOTTORI UNIV | 1 | 0.033 |
| TOKYO UNIV AGR TECHNOL | 1 | 0.033 |
| TOKYO UNIV AGR | 1 | 0.033 |
| TOKYO METROPOLITAN INST GERONTOL | 1 | 0.033 |
| TOKYO METROPOLITAN GERIATR HOSP | 1 | 0.033 |
| TOKUSHIMA BUNRI UNIV | 1 | 0.033 |
| TOHO UNIV | 1 | 0.033 |
| TIANJIN UNIV SCI TECHNOL | 1 | 0.033 |
| TIANJIN UNIV COMMERCE | 1 | 0.033 |
| TIANJIN UNIV | 1 | 0.033 |
| TIANJIN RENMIN HOSP | 1 | 0.033 |
| TIANJIN NEUROL INST | 1 | 0.033 |
| TIANJIN KEY LAB CARDIOVASC REMODELING TARGET OR | 1 | 0.033 |
| TIANJIN HUANHU HOSP | 1 | 0.033 |
| TIANJIN HOSP | 1 | 0.033 |
| TIANJIN GONGAN HOSP | 1 | 0.033 |
| TIANJIN FIRST CENT HOSP | 1 | 0.033 |
| TIANJIN DEPT HEPATOBILIARY TUMOR | 1 | 0.033 |
| TIANJIN CENT HOSP GYNECOL OBSTET | 1 | 0.033 |
| TIANJIN CANC INST | 1 | 0.033 |
| THOMAYER HOSP | 1 | 0.033 |
| THIRD PEOPLES HOSP ZHENGZHOU | 1 | 0.033 |
| THIRD PEOPLES HOSP SHENZHEN | 1 | 0.033 |
| THIRD PEOPLES HOSP NANTONG | 1 | 0.033 |
| THIRD PEOPLES HOSP BENGBU | 1 | 0.033 |
| TGS INST | 1 | 0.033 |
| TEXAS STATE UNIV | 1 | 0.033 |
| TENGZHOU CENT PEOPLES HOSP | 1 | 0.033 |
| TEL AVIV UNIV | 1 | 0.033 |
| TEIKYO UNIV | 1 | 0.033 |
| TCM | 1 | 0.033 |
| TARIM UNIV | 1 | 0.033 |
| TANGSHAN WORKERS HOSP | 1 | 0.033 |
| TANGSHAN CITY WORKERS HOSP HEBEI PROV | 1 | 0.033 |
| TANGSHAN CITY WORKERS HOSP | 1 | 0.033 |
| TAMPERE UNIV TECHNOL | 1 | 0.033 |
| TAMPERE UNIV HOSP | 1 | 0.033 |
| TAIZHOU OUTDO CLIN LAB | 1 | 0.033 |
| TAIYUAN UNIV TECHNOL | 1 | 0.033 |
| TAIYUAN PSYCHIAT HOSP | 1 | 0.033 |
| TAIPEI VET GEN HOSP | 1 | 0.033 |
| TAICHUNG VET GEN HOSP | 1 | 0.033 |
| TAICANG INST LIFE SCI INFORMAT | 1 | 0.033 |
| TAIAN CITY CENT HOSP | 1 | 0.033 |
| TABRIZ UNIV MED SCI | 1 | 0.033 |
| SYNAPTIC LTD | 1 | 0.033 |
| SWISS INST BIOINFORMAT SIB | 1 | 0.033 |
| SUNY BUFFALO | 1 | 0.033 |
| SUNY ALBANY | 1 | 0.033 |
| SUNGKYUNKWAN UNIV | 1 | 0.033 |
| SUN YET SEN UNIV | 1 | 0.033 |
| SUN YAI SEN UNIV | 1 | 0.033 |
| SUBEI PEOPLES HOSP JIANGSU PROV | 1 | 0.033 |
| STN ZOOL A DOHRN | 1 | 0.033 |
| STATE KEY LAB RESP DIS | 1 | 0.033 |
| STATE KEY LAB ONCOL SOUTHERN CHINA | 1 | 0.033 |
| STATE KEY LAB ONCOGENE RELATED GENES | 1 | 0.033 |
| ST JOSEF HOSP | 1 | 0.033 |
| ST JOHNS HOSP | 1 | 0.033 |
| ST JOHN GOD HOSP GRAZ | 1 | 0.033 |
| ST GEORG HOSP LEIPZIG | 1 | 0.033 |
| SOUTHWEST UNIV | 1 | 0.033 |
| SOUTHWEST MED UNIV CHINA | 1 | 0.033 |
| SOUTHERN MED UNIV GUANGZHOU | 1 | 0.033 |
| SOUTHEN MED UNIV | 1 | 0.033 |
| SOUTHEAST UNIV MED COLL | 1 | 0.033 |
| SOUTHAMPTON UNIV HOSP | 1 | 0.033 |
| SOUTH MED UNIV CHINA | 1 | 0.033 |
| SOLOMONBROTHERS MED INST | 1 | 0.033 |
| SOKENDAI GRAD UNIV ADV STUDIES | 1 | 0.033 |
| SODRA ALVSBORG HOSP | 1 | 0.033 |
| SLOAN KETTERING INST | 1 | 0.033 |
| SKOLKOVO INST SCI TECHNOL | 1 | 0.033 |
| SIXTH PEOPLES HOSP NANTONG | 1 | 0.033 |
| SIU | 1 | 0.033 |
| SIR RUN RUN HOSP | 1 | 0.033 |
| SINGAPORE NATL EYE CTR | 1 | 0.033 |
| SINGAPORE GEN HOSP | 1 | 0.033 |
| SIMONS FDN | 1 | 0.033 |
| SIMMONS COLL | 1 | 0.033 |
| SIKSHA O ANUSANDHAN UNIV | 1 | 0.033 |
| SIGNATURE GENOM INC | 1 | 0.033 |
| SICHUAN TECH EXCHANGE CTR | 1 | 0.033 |
| SICHUAN PROV CANC HOSP | 1 | 0.033 |
| SICHUAN MED UNIV | 1 | 0.033 |
| SICHUAN CANC HOSP | 1 | 0.033 |
| SHRINERS HOSP CHILDREN | 1 | 0.033 |
| SHINJO EYE CLIN | 1 | 0.033 |
| SHIMA RIVER COMMUNITY HLTH CTR | 1 | 0.033 |
| SHIJIAZHUANG OBSTET MATERNAL HLTH HOSP | 1 | 0.033 |
| SHIHEZI UNIV | 1 | 0.033 |
| SHENZHEN PEKING UNIV | 1 | 0.033 |
| SHENZHEN LONGGANG DIST CTR DIS CONTROL PREVENT | 1 | 0.033 |
| SHENYANG NORTHERN HOSP | 1 | 0.033 |
| SHENGLI OILFIELD CENT HOSP | 1 | 0.033 |
| SHEHONG HOSP TRADIT CHINESE MED | 1 | 0.033 |
| SHEBA MED CTR | 1 | 0.033 |
| SHAOYANG UNIV | 1 | 0.033 |
| SHAOXING SHANGYU PEOPLES HOSP | 1 | 0.033 |
| SHAOXING SECOND HOSP | 1 | 0.033 |
| SHAOXING PEPOPLES HOSP | 1 | 0.033 |
| SHAOXING PEOPLES HOSP | 1 | 0.033 |
| SHAOXING MUNICIPAL HOSP | 1 | 0.033 |
| SHANTOU UNIV MED COLL | 1 | 0.033 |
| SHANGHE CTY PEOPLES HOSP | 1 | 0.033 |
| SHANGHAI UNIV TRADIT MED | 1 | 0.033 |
| SHANGHAI UNIV TCM | 1 | 0.033 |
| SHANGHAI UNIV SCI TECHNOL | 1 | 0.033 |
| SHANGHAI UNIV CHINESE MED | 1 | 0.033 |
| SHANGHAI TONGJI UNIV | 1 | 0.033 |
| SHANGHAI TONGJI HOSP | 1 | 0.033 |
| SHANGHAI TECH UNIV | 1 | 0.033 |
| SHANGHAI TCM UNIV | 1 | 0.033 |
| SHANGHAI SOUTHGENE TECHNOL CO LTD | 1 | 0.033 |
| SHANGHAI PULM HOSP | 1 | 0.033 |
| SHANGHAI PUDONG HOSP | 1 | 0.033 |
| SHANGHAI PUDONG GONGLI HOSP | 1 | 0.033 |
| SHANGHAI PROTON HEAVY ION CTR | 1 | 0.033 |
| SHANGHAI ORIENTAL HOSP | 1 | 0.033 |
| SHANGHAI MUNICIPAL HOSP TRADIT CHINESE MED | 1 | 0.033 |
| SHANGHAI KEY LAB BIRTH DEFECT | 1 | 0.033 |
| SHANGHAI INST DIGEST DIS | 1 | 0.033 |
| SHANGHAI FU NENG BIOL TECHNOL CO LTD | 1 | 0.033 |
| SHANGHAI FIRST PEOPLES HOSP | 1 | 0.033 |
| SHANGHAI ENGN RES CTR PHARMACEUT TRANSLAT | 1 | 0.033 |
| SHANGHAI CHILDRENS HOSP | 1 | 0.033 |
| SHANGHAI CHANGZHENG HOSP | 1 | 0.033 |
| SHANGHAI ACAD SCI TECHNOL | 1 | 0.033 |
| SHANDONG UNIV TECHNOL | 1 | 0.033 |
| SHANDONG QIANFOSHAN HOSP | 1 | 0.033 |
| SHANDONG PROV CHEST HOSP | 1 | 0.033 |
| SHANDONG MED COLL | 1 | 0.033 |
| SHANDONG CHEST HOSP | 1 | 0.033 |
| SHANDONG AGR UNIV | 1 | 0.033 |
| SHANDONG ACAD CHINESE MED | 1 | 0.033 |
| SHAHID CHAMRAN UNIV AHVAZ | 1 | 0.033 |
| SHAANXI UNIV TRADIT CHINESE MED | 1 | 0.033 |
| SHAANXI UNIV CHINESE MED | 1 | 0.033 |
| SHAANXI NORMAL UNIV | 1 | 0.033 |
| SHAANXI KEY LAB MIL STOMATOL | 1 | 0.033 |
| SHAANNXI PROV PEOPLES HOSP | 1 | 0.033 |
| SEQUENTIA BIOTECH SL | 1 | 0.033 |
| SEMMELWEIS UNIV | 1 | 0.033 |
| SECOND PEOPLES HOSP WUHU | 1 | 0.033 |
| SECOND PEOPLES HOSP HUNAN PROV | 1 | 0.033 |
| SECOND PEOPLES HOSP CHANGZHOU | 1 | 0.033 |
| SECOND MIL UNIV | 1 | 0.033 |
| SECOND HOSP SICHUAN | 1 | 0.033 |
| SECOND HOSP NANJING | 1 | 0.033 |
| SECOND AFFILIATED HOSP | 1 | 0.033 |
| SEATTLE CHILDRENS HOSP | 1 | 0.033 |
| SCUOLA SUPER SANT ANNA | 1 | 0.033 |
| SCUOLA INT SUPERI STUDI AVANZATI | 1 | 0.033 |
| SCUOLA INT SUPER STUDI AVANZATI | 1 | 0.033 |
| SCOTTISH NATL BLOOD TRANSFUS SERV | 1 | 0.033 |
| SCHIZOPHRENIA RES INST | 1 | 0.033 |
| SCH ENGN | 1 | 0.033 |
| SB248 BILKENT UNIV ANKARA | 1 | 0.033 |
| SAPIENZA UNIV ROME | 1 | 0.033 |
| SANTARIS PHARMA | 1 | 0.033 |
| SANTA FE INST | 1 | 0.033 |
| SANQUIN RES LANDSTEINER LAB | 1 | 0.033 |
| SANFORD BURNHAM PREBYS MED DISCOVERY INST | 1 | 0.033 |
| SANFORD BURNHAM MED RES INST LAKE NONA | 1 | 0.033 |
| SANDA UNIV | 1 | 0.033 |
| SAN RAFFAELE PISANA SCI INST RES HOSPITALIZAT H | 1 | 0.033 |
| SAN MARTINO HOSP | 1 | 0.033 |
| SAN FRANCISCO STATE UNIV | 1 | 0.033 |
| SAMUEL ROBERTS NOBLE FDN INC | 1 | 0.033 |
| SALZBURG UNIV | 1 | 0.033 |
| SALESFORCE | 1 | 0.033 |
| SAITO LAB CELL TECHNOL | 1 | 0.033 |
| SAIC FREDERICK INC | 1 | 0.033 |
| SAHLGRENS ACAD | 1 | 0.033 |
| SACKLER FAC MED | 1 | 0.033 |
| S CHINA UNIV TECHNOL | 1 | 0.033 |
| RXGEN INC | 1 | 0.033 |
| RWTH UNIV | 1 | 0.033 |
| RUSH UNIV | 1 | 0.033 |
| ROYAL INST TECHNOL KTH | 1 | 0.033 |
| ROYAL HOSP WOMEN | 1 | 0.033 |
| ROYAL COLL SURGEONS IRELAND | 1 | 0.033 |
| ROYAL CHILDRENS HOSP | 1 | 0.033 |
| ROYAL BRISBANE WOMENS HOSP | 1 | 0.033 |
| ROTTERDAM UNIV | 1 | 0.033 |
| ROSETTA INPHARMAT LLC | 1 | 0.033 |
| ROCHE DIAGNOST | 1 | 0.033 |
| ROBERT BOSCH KRANKENHAUS | 1 | 0.033 |
| RIZZOLI ORTHOPED INST | 1 | 0.033 |
| RIKEN YOKOHAMA INST | 1 | 0.033 |
| RIKEN RES CTR ALLERGY IMMUNOL | 1 | 0.033 |
| RIKEN IMS | 1 | 0.033 |
| RIKEN GENOM SCI CTR | 1 | 0.033 |
| RIKEN CTR INTEGRAT MED SCI IMS RCAI | 1 | 0.033 |
| RIKEN ADV SCI INST | 1 | 0.033 |
| RICE UNIV | 1 | 0.033 |
| RIBEIRAO PRETO MED SCH | 1 | 0.033 |
| RHEIN WESTFAL TH AACHEN | 1 | 0.033 |
| RES NETWORK COMPUTAT BIOL | 1 | 0.033 |
| RES INST MOL PATHOL | 1 | 0.033 |
| RENMIN HOSP | 1 | 0.033 |
| REGENERON PHARMACEUT REGENERON GENET CTR | 1 | 0.033 |
| REG BLOOD CTR RIBEIRAO PRETO | 1 | 0.033 |
| REFERRAL CANC CTR BASILICATA | 1 | 0.033 |
| REAL ACAD CIENCIES ARTS BARCELONA | 1 | 0.033 |
| RAMON Y CAJAL UNIV HOSP | 1 | 0.033 |
| RADIUMHOSP | 1 | 0.033 |
| RADBOUDUMC | 1 | 0.033 |
| RADBOUD UNIV NIJMEGEN MED CTR | 1 | 0.033 |
| RADBOUD INST MOL LIFE SCI | 1 | 0.033 |
| QUEEN SILVIA CHILDRENS HOSP | 1 | 0.033 |
| QUEEN MARY UNIV LONDON | 1 | 0.033 |
| QUEBEC HEART LUNG INST | 1 | 0.033 |
| QIQIHAR MED UNIV | 1 | 0.033 |
| QIQIHAR MED COLL | 1 | 0.033 |
| QINGDAO WOMEN CHILDREN HOSP | 1 | 0.033 |
| QINGDAO MED UNIV | 1 | 0.033 |
| QINGDAO COMMERCIAL EMPLOYEES HOSP | 1 | 0.033 |
| QIMR BERGHOFER MED RES INST | 1 | 0.033 |
| QIDONG LIVER CANC RES INST | 1 | 0.033 |
| QIDONG LIVER CANC INST | 1 | 0.033 |
| QEII MED CTR | 1 | 0.033 |
| PURDUE UNIV LIB | 1 | 0.033 |
| PUKOU DIST CTR DIS CONTROL PREVENT | 1 | 0.033 |
| PUBL RES CTR HLTH CRP SANTE | 1 | 0.033 |
| PUBL HLTH ONTARIO | 1 | 0.033 |
| PUBL HLTH AGCY CANADA | 1 | 0.033 |
| PROVIDENCE REG CANC CTR | 1 | 0.033 |
| POZNAN UNIV MED SCI | 1 | 0.033 |
| POSTECH | 1 | 0.033 |
| PONTIFICIA UNIV CATOLICA CHILE | 1 | 0.033 |
| PONDICHERRY UNIV | 1 | 0.033 |
| POLICLIN SAN DONATO IRCCS | 1 | 0.033 |
| PLA CTR HOSP | 1 | 0.033 |
| PEOPLES HOSP ZOUCHENG CITY | 1 | 0.033 |
| PEOPLES HOSP ZHANGQIU CITY SHANDONG PROV | 1 | 0.033 |
| PEOPLES HOSP YINAN | 1 | 0.033 |
| PEOPLES HOSP XINJIANG UYGHUR AUTONOMOUS REG | 1 | 0.033 |
| PEOPLES HOSP XIN TAI | 1 | 0.033 |
| PEOPLES HOSP TONGLING | 1 | 0.033 |
| PEOPLES HOSP SHOUGUANG CITY | 1 | 0.033 |
| PEOPLES HOSP RIZHAO | 1 | 0.033 |
| PEOPLES HOSP QUZHOU CITY | 1 | 0.033 |
| PEOPLES HOSP PUTUO | 1 | 0.033 |
| PEOPLES HOSP PINGYI CTY | 1 | 0.033 |
| PEOPLES HOSP LISHUI | 1 | 0.033 |
| PEOPLES HOSP KENLI CTY | 1 | 0.033 |
| PEOPLES HOSP JINGJIANG | 1 | 0.033 |
| PEOPLES HOSP JIAOZUO CITY | 1 | 0.033 |
| PEOPLES HOSP HUIXIAN | 1 | 0.033 |
| PEOPLES HOSP HESHAN | 1 | 0.033 |
| PEOPLES HOSP GUANGXI ZHUANG AUTONOMOUS REG | 1 | 0.033 |
| PEOPLES HOSP BINZHOU CITY | 1 | 0.033 |
| PEOPLES HOSP BINZHOU | 1 | 0.033 |
| PEOPLES HOSP | 1 | 0.033 |
| PEOPLE HOSP SUSONG | 1 | 0.033 |
| PENN STATE HERSHEY MED CTR | 1 | 0.033 |
| PENN STATE COLL MED | 1 | 0.033 |
| PENINSULA SCH MED DENT | 1 | 0.033 |
| PENINSULA COLL MED DENT | 1 | 0.033 |
| PEKING UNIV THIRD HOSP | 1 | 0.033 |
| PEKING TSINGHUA CTR LIFE SCI | 1 | 0.033 |
| PARTNER SITE RHEINMAIN | 1 | 0.033 |
| PARTNER SIDE RHEIN MAIN | 1 | 0.033 |
| PARCO TECNOL PADANO | 1 | 0.033 |
| PARACELSUS MED UNIV | 1 | 0.033 |
| PANYU CENT HOSP | 1 | 0.033 |
| PACIFIC NORTHWEST REG CTR EXCELLENCE BIODEF EME | 1 | 0.033 |
| PACIFIC NORTHWEST NATL LAB | 1 | 0.033 |
| OZAKI EYE HOSP | 1 | 0.033 |
| OXFORD UNIV HOSP | 1 | 0.033 |
| OXFORD BROOKES UNIV | 1 | 0.033 |
| OTTAWA HOSP | 1 | 0.033 |
| OSWALDO CRUZ FDN CPQGM FIOCRUZ | 1 | 0.033 |
| ORTHOPAED HOSP ZHENGZHOU | 1 | 0.033 |
| OREGON HLTH SCI UNIV | 1 | 0.033 |
| OPKO HLTH INC | 1 | 0.033 |
| OPKO CURNA | 1 | 0.033 |
| OPEN SOURCE DRUG DISCOVERY UNIT | 1 | 0.033 |
| ONCONOST TECHNOL | 1 | 0.033 |
| ONCOL HOSP CHENGDU | 1 | 0.033 |
| OMORI RED CROSS HOSP | 1 | 0.033 |
| OKLAHOMA STATE UNIV | 1 | 0.033 |
| OKAYAMA UNIV | 1 | 0.033 |
| OITA UNIV HOSP | 1 | 0.033 |
| OITA UNIV | 1 | 0.033 |
| OCEAN UNIV CHINA | 1 | 0.033 |
| NUCL IND 215 HOSP | 1 | 0.033 |
| NTNU | 1 | 0.033 |
| NOVO NORDISK | 1 | 0.033 |
| NOVARTIS INST BIOMED RES | 1 | 0.033 |
| NORWEGIAN RADIUM HOSP | 1 | 0.033 |
| NORTHERN JIANGSU PROV HOSP | 1 | 0.033 |
| NORTHERN JIANGSU PEOPLES HOSP | 1 | 0.033 |
| NORTHEASTERN UNIV | 1 | 0.033 |
| NORTHEAST NORMAL UNIV | 1 | 0.033 |
| NORTH ZEALAND HOSP | 1 | 0.033 |
| NORTH SICHUAN MED COLL | 1 | 0.033 |
| NORTH SHORE CANC INST | 1 | 0.033 |
| NORRIS COTTON CANC CTR | 1 | 0.033 |
| NORMANDIE UNIV | 1 | 0.033 |
| NINTH PEOPLES HOSP XIAN | 1 | 0.033 |
| NINTH HOSP XIAN | 1 | 0.033 |
| NINGBO YINZHOU PEOPLES HOSP | 1 | 0.033 |
| NINGBO UROL NEPHROT HOSP | 1 | 0.033 |
| NINGBO SECOND HOSP | 1 | 0.033 |
| NINGBO PUBL SECUR BUR | 1 | 0.033 |
| NINGBO FIRST HOSP | 1 | 0.033 |
| NINGBO COLL HLTH SCI | 1 | 0.033 |
| NINGBO CLIN PATHOL DIAG CTR | 1 | 0.033 |
| NINGBO 7 HOSP | 1 | 0.033 |
| NINGBO 1 HOSP | 1 | 0.033 |
| NIHON UNIV | 1 | 0.033 |
| NIH UNIV CAMBRIDGE BIOMED SCHOLARS PROGRAM | 1 | 0.033 |
| NIG | 1 | 0.033 |
| NIBB | 1 | 0.033 |
| NEW YORK UNIV CANC INST | 1 | 0.033 |
| NEW YORK BLOOD CTR | 1 | 0.033 |
| NEW MEXICO CONSORTIUM | 1 | 0.033 |
| NETHERLANDS HEART INST ICIN | 1 | 0.033 |
| NEAR EAST UNIV | 1 | 0.033 |
| NAVY GEN HOSP | 1 | 0.033 |
| NATL UNIV LA PLATA | 1 | 0.033 |
| NATL UNIV IRELAND UNIV COLL CORK | 1 | 0.033 |
| NATL TSING HUA UNIV | 1 | 0.033 |
| NATL TECH UNIV ATHENS | 1 | 0.033 |
| NATL RES INST CHILD HLTH DEV | 1 | 0.033 |
| NATL RES CTR FRONTIERS GENET | 1 | 0.033 |
| NATL RES COUNTRY ITALY | 1 | 0.033 |
| NATL RES COUNCIL ITALY CNR | 1 | 0.033 |
| NATL RES COUNCIL ITALY | 1 | 0.033 |
| NATL RES COUNCIL CNR | 1 | 0.033 |
| NATL PLANT GENE RES CTR BEIJING | 1 | 0.033 |
| NATL LOCAL UNITED ENGN RES CTR SANQI RESOURCES | 1 | 0.033 |
| NATL LAB | 1 | 0.033 |
| NATL INST SCI TECHNOL TROP DIS INCT DT | 1 | 0.033 |
| NATL INST SCI TECHNOL | 1 | 0.033 |
| NATL INST NAT SCI | 1 | 0.033 |
| NATL INST MED RES | 1 | 0.033 |
| NATL INST FOOD DRUG CONTROL | 1 | 0.033 |
| NATL INST CHEM | 1 | 0.033 |
| NATL HLTH FAMILY PLANNING COMMISS | 1 | 0.033 |
| NATL ENGN CTR BIOCHIP SHANGHAI | 1 | 0.033 |
| NATL CTR NANOSCI TECHNOL | 1 | 0.033 |
| NATL CTR LIVER CANC | 1 | 0.033 |
| NATL CTR GLOBAL HLTH MED | 1 | 0.033 |
| NATL CREAT RES INITIAT CTR SYMBIOSYST | 1 | 0.033 |
| NATL CLIN RES CTR RESP DIS | 1 | 0.033 |
| NATL CLIN RES CTR DIGEST DIS | 1 | 0.033 |
| NATL CHUNG CHENG UNIV | 1 | 0.033 |
| NATL CANC CTR | 1 | 0.033 |
| NANTONG UNIV MED | 1 | 0.033 |
| NANTONG PRISON HOSP | 1 | 0.033 |
| NANTONG MED UNIV | 1 | 0.033 |
| NANTONG JIANGSU UNIV | 1 | 0.033 |
| NANJINGMED UNIV | 1 | 0.033 |
| NANJING UROL RES CTR | 1 | 0.033 |
| NANJING UNIV TCM | 1 | 0.033 |
| NANJING UNIV AERONAUT ASTRONAUT | 1 | 0.033 |
| NANJING MATERNAL CHILD HLTH INST | 1 | 0.033 |
| NANJING MATERNAL CHILD HLTH HOSP | 1 | 0.033 |
| NANJING MATERNAL CHILD HLTH CARE HOSP | 1 | 0.033 |
| NANJING JINLING HIGH SCH | 1 | 0.033 |
| NANJING HOSP TRADIT CHINESE MED | 1 | 0.033 |
| NANCYCLOTEP EXPT IMAGING PLATFORM | 1 | 0.033 |
| NANCHANG INST SCI TECHNOL | 1 | 0.033 |
| NAN KAI UNIV | 1 | 0.033 |
| NAGOYA UNIV | 1 | 0.033 |
| NAGOYA CITY UNIV | 1 | 0.033 |
| NAGAHAMA INST BIOSCI TECHNOL | 1 | 0.033 |
| NAESTVED HOSP | 1 | 0.033 |
| MUNICH LEUKEMIA LAB GMBH | 1 | 0.033 |
| MUDANJIANG MED UNIV | 1 | 0.033 |
| MUDANJIANG MED COLL | 1 | 0.033 |
| MTA SZTE DERMATOL RES GRP | 1 | 0.033 |
| MT SINAI HOSP | 1 | 0.033 |
| MSD CHINA R D | 1 | 0.033 |
| MOORFIELDS EYE HOSP NHSFT | 1 | 0.033 |
| MOORES UCSD CANC CTR | 1 | 0.033 |
| MOE | 1 | 0.033 |
| MIZOGUCHI EYE HOSP | 1 | 0.033 |
| MIYAZAKI UNIV | 1 | 0.033 |
| MIYATA EYE HOSP | 1 | 0.033 |
| MIYAGI CANC CTR | 1 | 0.033 |
| MITSUBISHI SPACE SOFTWARE CO LTD | 1 | 0.033 |
| MIT HARVARD | 1 | 0.033 |
| MISSISSIPPI STATE UNIV | 1 | 0.033 |
| MINNESOTA NEONATAL PHYS | 1 | 0.033 |
| MINIST PUBL SECUR | 1 | 0.033 |
| MINIST AGR | 1 | 0.033 |
| MIHARA MEM HOSP | 1 | 0.033 |
| MIE UNIV | 1 | 0.033 |
| MIANYANG CENT HOSP | 1 | 0.033 |
| METHODIST HOSP | 1 | 0.033 |
| MERCK CO INC | 1 | 0.033 |
| MED UNIV INNSBRUCK | 1 | 0.033 |
| MED SCH CHINESE PLA | 1 | 0.033 |
| MED HSCH HANNOVER INST BIOCHEM | 1 | 0.033 |
| MED FAC CARL GUSTAV CARUS | 1 | 0.033 |
| MED CONSULTAT OFF INC | 1 | 0.033 |
| MCKUSICK NATHANS INST GENET MED | 1 | 0.033 |
| MAYO CLIN FLORIDA | 1 | 0.033 |
| MAX PLANCK INST PHYS KOMPLEXER SYST | 1 | 0.033 |
| MAX PLANCK INST NEUROL RES | 1 | 0.033 |
| MAX PLANCK INST MATH SCI | 1 | 0.033 |
| MAX PLANCK INST EVOLUTIONARY ANTHROPOL | 1 | 0.033 |
| MAX PLANCK INST BRAIN RES | 1 | 0.033 |
| MAX PLANCK INST BIOPHYS CHEM | 1 | 0.033 |
| MAX DELBRCK CTR MOL MED | 1 | 0.033 |
| MATERNAL CHILDREN HLTH HOSP NANTONG CITY | 1 | 0.033 |
| MATERNAL CHILD HLTH CARE HOSP NANTONG | 1 | 0.033 |
| MATERN CHILD CARE HOSP | 1 | 0.033 |
| MATER MISERICORDIAE UNIV HOSP | 1 | 0.033 |
| MATER HOSP | 1 | 0.033 |
| MASARYK UNIV BRNO | 1 | 0.033 |
| MARSHFIELD CLIN RES FDN | 1 | 0.033 |
| MARCH DIMES FDN | 1 | 0.033 |
| MALLEK ASHTAR UNIV TECHNOL | 1 | 0.033 |
| MAHIDOL UNIV | 1 | 0.033 |
| MAGNA GRAECIA UNIV CATANZARO | 1 | 0.033 |
| LUOYANG ORTHOPED HOSP | 1 | 0.033 |
| LONGSAI HOSP | 1 | 0.033 |
| LONGGANG DIST CENT HOSP | 1 | 0.033 |
| LONG HUA CTR DIS CONTROL PREVENT | 1 | 0.033 |
| LONDON SCH HYG TROP MED | 1 | 0.033 |
| LOMONOSOV MOSCOW STATE UNIV | 1 | 0.033 |
| LOGIST UNIV PEOPLES ARMED POLICE FORCE | 1 | 0.033 |
| LOGIST UNIV | 1 | 0.033 |
| LISHUI PEOPLES HOSP | 1 | 0.033 |
| LISHUI CTR DIS CONTROL PREVENT | 1 | 0.033 |
| LISHUI CENT HOSP | 1 | 0.033 |
| LINYI WOMEN CHILDRENS HOSP | 1 | 0.033 |
| LINYI PEOPLE HOSP | 1 | 0.033 |
| LINYI ONCOSURG HOSP | 1 | 0.033 |
| LINYI CHEST HOSP | 1 | 0.033 |
| LINKOPING UNIV | 1 | 0.033 |
| LIHUILI HOSP | 1 | 0.033 |
| LIAONING RES CTR TRANSLAT MED NERVOUS SYST DIS | 1 | 0.033 |
| LIAONING MED UNIV | 1 | 0.033 |
| LIAONING CANC HOSP INST | 1 | 0.033 |
| LIAONING CANC HOSP INSITITUTE | 1 | 0.033 |
| LIAOCHENG THIRD PEOPLES HOSP | 1 | 0.033 |
| LIAOCHENG HOSP TRADIT CHINESE MED | 1 | 0.033 |
| LIANYUNGANG MATERNAL CHILDRENS HOSP LIANYUNGANG | 1 | 0.033 |
| LI KA SHING CTR | 1 | 0.033 |
| LEIBNIZ INST PLANT BIOCHEM | 1 | 0.033 |
| LAUSANNE UNIV HOSP CHUV | 1 | 0.033 |
| LANXI PEOPLES HOSP | 1 | 0.033 |
| LANKENAU MED CTR | 1 | 0.033 |
| LALLIANCE BOVITEQ INC | 1 | 0.033 |
| LA TROBE UNIV BUNDOORA | 1 | 0.033 |
| KYUSHU INST TECHNOL | 1 | 0.033 |
| KUNMING INST ZOOL | 1 | 0.033 |
| KUNMING GEN HOSP CHENGDU MIL COMMAND | 1 | 0.033 |
| KUNMING GEN HOSP CHENDU MIL COMMAND | 1 | 0.033 |
| KRIBB | 1 | 0.033 |
| KOCH INST INTEGRAT CANC RES | 1 | 0.033 |
| KNAW | 1 | 0.033 |
| KLINIKUM ST GEORG | 1 | 0.033 |
| KING ABDULAZIZ UNIV | 1 | 0.033 |
| KEY LAB XIAMEN MARINE GENE DRUGS | 1 | 0.033 |
| KEY LAB THORAC TUMORS ZHENGZHOU | 1 | 0.033 |
| KEY LAB ORGAN TRANSPLANTAT | 1 | 0.033 |
| KEY LAB MED MOL DIAGNOST GUANGDONG PROV | 1 | 0.033 |
| KEY LAB HEPATOBILIARY PANCREAT SURG DIGEST OR | 1 | 0.033 |
| KEY LAB FUJIAN MOL MED | 1 | 0.033 |
| KEY LAB COMBINED MULTIORGAN TRANSPLANTAT | 1 | 0.033 |
| KEY LAB BONE MARROW STEM CELL | 1 | 0.033 |
| KEMRI WELLCOME TRUST RES PROGRAMME | 1 | 0.033 |
| KARLSRUHE INST TECHNOL | 1 | 0.033 |
| KANTONSSPITAL BADEN AG | 1 | 0.033 |
| KAIFENG CENT HOSP | 1 | 0.033 |
| KAGOSHIMA UNIV | 1 | 0.033 |
| KADLEC REG MED CTR | 1 | 0.033 |
| KACKAR STATE HOSP | 1 | 0.033 |
| JUSTUS LIEBIG UNIV | 1 | 0.033 |
| JUNTENDO UNIV | 1 | 0.033 |
| JOSEP CARRERAS LEUKEMIA RES INST IJC | 1 | 0.033 |
| JOHNS HOPKINS MED INST | 1 | 0.033 |
| JOHN INNES CTR PLANT SCI RES | 1 | 0.033 |
| JOHN HUNTER HOSP | 1 | 0.033 |
| JIYANG HOSP TRADIT CHINESE MED | 1 | 0.033 |
| JINXIANG CTY PEOPLES HOSP | 1 | 0.033 |
| JINING FIRST PEOPLES HOSP | 1 | 0.033 |
| JINAN VOCAT COLL NURSING | 1 | 0.033 |
| JILIN MED UNIV | 1 | 0.033 |
| JIKEI UNIV | 1 | 0.033 |
| JIAO TONG UNIV | 1 | 0.033 |
| JIANGXI UNIV TRADIT CHINESE MED | 1 | 0.033 |
| JIANGXI PROV BLOOD CTR | 1 | 0.033 |
| JIANGXI MATERNAL CHILD HLTH HOSP | 1 | 0.033 |
| JIANGXI CANC HOSP | 1 | 0.033 |
| JIANGSU PROV OFFICIAL HOSP | 1 | 0.033 |
| JIANGSU KEY LAB PREVENT TRANSLAT MED GERIATR DI | 1 | 0.033 |
| JIANGSU HUAIAN HOSP | 1 | 0.033 |
| JIANGSU HIGHER EDUC INST | 1 | 0.033 |
| JIANGSU CTR DIS CONTROL PREVENT | 1 | 0.033 |
| JIANGSU COINNOVAT CTR PREVENT CONTROL IMPORTANT | 1 | 0.033 |
| JIANGSU CANC HOSP | 1 | 0.033 |
| JIAMUSI UNIV | 1 | 0.033 |
| JERSEY SHORE UNIV | 1 | 0.033 |
| JAYPEE UNIV INFORMAT TECHNOL | 1 | 0.033 |
| JAWAHARLAL NEHRU CTR ADV SCI RES | 1 | 0.033 |
| JAPANESE FDN CANC RES | 1 | 0.033 |
| JAPAN SCI TECHNOL AGCY | 1 | 0.033 |
| JAMES HUTTON INST | 1 | 0.033 |
| JAMES COOK UNIV HOSP | 1 | 0.033 |
| JAMES COOK UNIV | 1 | 0.033 |
| JAMES A HALEY VET HOSP | 1 | 0.033 |
| JAGIELLONIAN UNIV | 1 | 0.033 |
| JACKSON LAB GENOM MED | 1 | 0.033 |
| IUPUI | 1 | 0.033 |
| IULIU HATIEGANU UNIV MED PHARM | 1 | 0.033 |
| IU INST ONCOL | 1 | 0.033 |
| ITALIAN INST TECHNOL IIT | 1 | 0.033 |
| IST TOSCANO TUMORI | 1 | 0.033 |
| IST SUPER SANITA | 1 | 0.033 |
| IST SCI ROMAGNOLO STUDIO CURA TUMORI IRST SRL I | 1 | 0.033 |
| IST PASTEUR FDN CENCI BOLOGNETTI | 1 | 0.033 |
| IST ITALIAN TECNOL | 1 | 0.033 |
| IST AUSTRIA | 1 | 0.033 |
| IST ANAT PATOL UNIV | 1 | 0.033 |
| ISMETT IRCCS | 1 | 0.033 |
| ISLAMIC AZAD UNIV | 1 | 0.033 |
| IRISA INRIA GENSCALE | 1 | 0.033 |
| IRCSS NATL CANC INST | 1 | 0.033 |
| IRCS NATL TUMOR INST | 1 | 0.033 |
| IRCCS POLICLIN SAN DONATO | 1 | 0.033 |
| IRCCS IST AUXOL ITALIANO | 1 | 0.033 |
| IRCCS CROB | 1 | 0.033 |
| IRCCS AOU SAN MARTINO IST | 1 | 0.033 |
| IRAN UNIV MED SCI | 1 | 0.033 |
| IOWA STATE UNIV | 1 | 0.033 |
| INTERVENT TREATMENT CLIN RES CTR HENAN PROV | 1 | 0.033 |
| INTEGRATED DNA TECHNOL INC | 1 | 0.033 |
| INTEC INC | 1 | 0.033 |
| INT RES CTR AC | 1 | 0.033 |
| INT CTR GENET ENGN BIOTECHNOL | 1 | 0.033 |
| INST SYST BIOL | 1 | 0.033 |
| INST SKULL BASE SURG NEUROONCOL HUNAN | 1 | 0.033 |
| INST SALUD CARLOS III | 1 | 0.033 |
| INST POLITECN NACL | 1 | 0.033 |
| INST PATHOL GENET | 1 | 0.033 |
| INST NEUROL | 1 | 0.033 |
| INST NACL CIENCIA TECNOL ONCOGENOM | 1 | 0.033 |
| INST NACL CANCEROL | 1 | 0.033 |
| INST NACL CANC JOSE ALENCAR GOMES DA SILVA | 1 | 0.033 |
| INST NACL CANC | 1 | 0.033 |
| INST MOL TRANSLAT THERAPEUT STRATEGIES IMTTS | 1 | 0.033 |
| INST MOL CELL BIOL | 1 | 0.033 |
| INST MOL BIOTECHNOL | 1 | 0.033 |
| INST MED RES | 1 | 0.033 |
| INST MED GENET | 1 | 0.033 |
| INST MATERNAL CHILD HLTH IRCCS BURLO GAROFOLO | 1 | 0.033 |
| INST INVEST AUGUST PI SUNYER IDIBAPS | 1 | 0.033 |
| INST HLTH RES NAVARRA IDISNA | 1 | 0.033 |
| INST HENAN PROV | 1 | 0.033 |
| INST GULBENKIAN CIENCIAS | 1 | 0.033 |
| INST GERONTOL | 1 | 0.033 |
| INST GENET GENOM GENEVA IGE3 | 1 | 0.033 |
| INST FORENS SCI | 1 | 0.033 |
| INST EPIDEMIOL 1 | 1 | 0.033 |
| INST COLOR SCI TECHNOL | 1 | 0.033 |
| INST CHEM BIOL | 1 | 0.033 |
| INST CATALANA RECERCA ESTUDIS AVANCATS ICREA | 1 | 0.033 |
| INST CATALANA RECERCA ESTUDIS AVANCATS | 1 | 0.033 |
| INST CANC RES | 1 | 0.033 |
| INST BUTANTAN | 1 | 0.033 |
| INST BIOINFORMAT BIOTECHNOL 2BIO | 1 | 0.033 |
| INST BIOINFORMAT | 1 | 0.033 |
| INST BIOCHEM CELL BIOL | 1 | 0.033 |
| INST ALBERT BONNIOT | 1 | 0.033 |
| INRA AUZEVILLE | 1 | 0.033 |
| INNER MONGOLIA MED COLL | 1 | 0.033 |
| INNER MONGOLIA AUTONOMOUS REG HOSP | 1 | 0.033 |
| INGM | 1 | 0.033 |
| INGHAM INST APPL MED RES | 1 | 0.033 |
| INDIAN INST SCI | 1 | 0.033 |
| INDEPTH NETWORK | 1 | 0.033 |
| INCELL CORP | 1 | 0.033 |
| IMB | 1 | 0.033 |
| ILS GENOM | 1 | 0.033 |
| IGIB | 1 | 0.033 |
| IGBMC | 1 | 0.033 |
| IDIBELL | 1 | 0.033 |
| ICAR NATL RES CTR BANANA | 1 | 0.033 |
| IBM THOMAS J WATSON RES CTR | 1 | 0.033 |
| HUZHOU TEACHERS COLL | 1 | 0.033 |
| HUNAN UNIV | 1 | 0.033 |
| HUNAN PROV TUMOR HOSP | 1 | 0.033 |
| HUNAN PROV PEOPLES HOSP | 1 | 0.033 |
| HUNAN PROV KEY LAB GENET IMPROVEMENT DOMEST ANIM | 1 | 0.033 |
| HUNAN PROV COOPERAT INNOVAT CTR MOL TARGET NEW DR | 1 | 0.033 |
| HUNAN POLYTECH ENVIRONM BIOL | 1 | 0.033 |
| HUNAN CHILDRENS HOSP | 1 | 0.033 |
| HUNAN AGR UNIV | 1 | 0.033 |
| HUMBOLDT UNIV | 1 | 0.033 |
| HUMAN GENET FDN | 1 | 0.033 |
| HUDSONALPHA INST BIOTECHNOL | 1 | 0.033 |
| HUBRECHT INST | 1 | 0.033 |
| HUBEI XINHUA HOSP | 1 | 0.033 |
| HUBEI UNIV TRADIT CHINESE MED | 1 | 0.033 |
| HUBEI UNIV SCI TECHNOL | 1 | 0.033 |
| HUBEI PROV KEY LAB ALLERGY IMMUNOL | 1 | 0.033 |
| HUBEI ENGN UNIV | 1 | 0.033 |
| HUBEI CANC HOSP | 1 | 0.033 |
| HUAZHONG CENT CHINA AGR UNIV | 1 | 0.033 |
| HUAIYANG CTY PEOPLES HOSP ZHOUKOU | 1 | 0.033 |
| HUAIAN SECOND PEOPLES HOSP | 1 | 0.033 |
| HUAIAN MATERN CHILD HEALTHCARE HOSP | 1 | 0.033 |
| HUAIAN FIRST PEOPLES HOSP | 1 | 0.033 |
| HUAIAN 4TH PEOPLES HOSP | 1 | 0.033 |
| HOSP XINJIANG PROD CONSTRUCT CORPS | 1 | 0.033 |
| HOSP VIRGEN DE LA VICTORIA | 1 | 0.033 |
| HOSP UNIV PUERTA HIERRO MAJADAHONDA | 1 | 0.033 |
| HOSP UNIV LA PAZ | 1 | 0.033 |
| HOSP UNIV ELECT SCI TECHNOL CHINA | 1 | 0.033 |
| HOSP UNIV BELLVITGE | 1 | 0.033 |
| HOSP SUZHOU SCI TECHNOL TOWN | 1 | 0.033 |
| HOSP SPECIAL SURG | 1 | 0.033 |
| HOSP SON LLATZER | 1 | 0.033 |
| HOSP SON ESPASES | 1 | 0.033 |
| HOSP SAN JUAN DIOS | 1 | 0.033 |
| HOSP MUNICIPAL BADALONA | 1 | 0.033 |
| HOSP MATERN CHILD CARE JINAN CITY | 1 | 0.033 |
| HOSP JOAN 23 | 1 | 0.033 |
| HOSP HARBIN INST TECHNOL | 1 | 0.033 |
| HOSP FUDAN UNIV | 1 | 0.033 |
| HOSP DURAN REYNALS | 1 | 0.033 |
| HOSP CLIN BARCELONA | 1 | 0.033 |
| HOSP CLIN | 1 | 0.033 |
| HOSP BARROS LUCO | 1 | 0.033 |
| HOSP BADALONA GERMANS TRIAS PUJOL | 1 | 0.033 |
| HOP ST LOUIS | 1 | 0.033 |
| HOP EUROPEEN GEORGES POMPIDOU | 1 | 0.033 |
| HOP BEAUJON | 1 | 0.033 |
| HONGHE AUTONOMOUS PREFECTURE HANI YI ETHN MINOR | 1 | 0.033 |
| HONG KONG UNIV SCI TECHNOL | 1 | 0.033 |
| HONG KONG BAPTIST UNIV | 1 | 0.033 |
| HLTH CANADA | 1 | 0.033 |
| HITACHI LTD | 1 | 0.033 |
| HILLSDALE COLL | 1 | 0.033 |
| HENAN PROVINCAL CANC HOSP | 1 | 0.033 |
| HENAN POLYTECH UNIV | 1 | 0.033 |
| HENAN ACAD AGR SCI | 1 | 0.033 |
| HELWAN UNIV | 1 | 0.033 |
| HELMHOLTZ ZENTRUM MUNICH | 1 | 0.033 |
| HELICOS BIOSCI | 1 | 0.033 |
| HEILONGJIANG PROV HOSP | 1 | 0.033 |
| HEILONGJIANG MED SCI ACAD | 1 | 0.033 |
| HEFEI UNIV TECHNOL | 1 | 0.033 |
| HEBEI UNIV SCI TECHNOL | 1 | 0.033 |
| HEBEI NORTH UNIV | 1 | 0.033 |
| HEBEI CHEST HOSP | 1 | 0.033 |
| HE NAN UNIV SCI TECHNOL | 1 | 0.033 |
| HAYASHI EYE HOSP | 1 | 0.033 |
| HARVARD SCH PUBL HLTH | 1 | 0.033 |
| HARVARD MASSACHUSETTS INST TECHNOL | 1 | 0.033 |
| HARRY PERKINS INST MED RES | 1 | 0.033 |
| HARBIN MED COLL | 1 | 0.033 |
| HARBIN ENGN UNIV | 1 | 0.033 |
| HARBIN BINGHUA HOSP | 1 | 0.033 |
| HANZHONG MUNICIPAL CENT HOSP | 1 | 0.033 |
| HANYANG UNIV | 1 | 0.033 |
| HANGZHOU NORMAL UNIV | 1 | 0.033 |
| HANGZHOU FIRST PEOPLES HOSP | 1 | 0.033 |
| HAMAMATSU UNIV | 1 | 0.033 |
| HAMADAN UNIV MED SCI | 1 | 0.033 |
| HADASSAH UNIV HOSP | 1 | 0.033 |
| HADASSAH HEBREW UNIV | 1 | 0.033 |
| GUSTAVE ROUSSY COMPREHENS CANC CTR | 1 | 0.033 |
| GUSTAVE ROUSSY | 1 | 0.033 |
| GUIZHOU PROV OSTEOL HOSP | 1 | 0.033 |
| GUIZHOU MED UNIV | 1 | 0.033 |
| GUIYANG MED UNIV | 1 | 0.033 |
| GUILIN MED COLL | 1 | 0.033 |
| GUANGZHOU UNIV TRADIT CHINESE MED | 1 | 0.033 |
| GUANGZHOU UNIV CHINESE MED | 1 | 0.033 |
| GUANGZHOU RIBOBIO CO LTD | 1 | 0.033 |
| GUANGZHOU MIL COMMAND PLA | 1 | 0.033 |
| GUANGZHOU INST OBSTET GYNECOL | 1 | 0.033 |
| GUANGZHOU INST CARDIOVASC DIS | 1 | 0.033 |
| GUANGZHOU INST BIOMED HLTH | 1 | 0.033 |
| GUANGZHOU FIRST PEOPLES HOSP | 1 | 0.033 |
| GUANGZHOU 8 PEOPLES HOSP | 1 | 0.033 |
| GUANGXI TEACHERS EDUC UNIV | 1 | 0.033 |
| GUANGDONG PROV MATERN CHILD CARE CTR | 1 | 0.033 |
| GUANGDONG PROV KEY LAB STOMATOL | 1 | 0.033 |
| GUANGDONG PROV KEY LAB MED MOL DIAGNOST | 1 | 0.033 |
| GUANGDONG PROV KEY LAB ARRHYTHMIA ELECTROPHYSIO | 1 | 0.033 |
| GUANGDONG OCEAN UNIV | 1 | 0.033 |
| GUANGDONG ACAD MED SCI | 1 | 0.033 |
| GUANGDONG 999 BRAIN HOSP | 1 | 0.033 |
| GRP HOSP LYON NORD | 1 | 0.033 |
| GREAT ORMOND ST HOSP SICK CHILDREN | 1 | 0.033 |
| GRAD UNIV ADV STUDIES SOKENDAI | 1 | 0.033 |
| GRAD UNIV ADV STUDIES | 1 | 0.033 |
| GOVT COLL UNIV | 1 | 0.033 |
| GOTHENBURG UNIV | 1 | 0.033 |
| GOPATH LABS LLC | 1 | 0.033 |
| GOPATH DIAGNOST LAB CO LTD | 1 | 0.033 |
| GLOBAL ROBOT INST | 1 | 0.033 |
| GLADSTONE INST CARDIOVASC DIS | 1 | 0.033 |
| GFE BLUT MBH | 1 | 0.033 |
| GERMAN RES CTR ENVIRONM HLTH | 1 | 0.033 |
| GERMAN CTR LUNG RES DZL | 1 | 0.033 |
| GERMAN CTR INFECT RES DZIF | 1 | 0.033 |
| GERMAN CTR CARDIOVASC RES | 1 | 0.033 |
| GERMAN CANC CONSORTIUM | 1 | 0.033 |
| GEORGETOWN UNIV | 1 | 0.033 |
| GEORG SPEYER HAUS | 1 | 0.033 |
| GENZYME GENET INC | 1 | 0.033 |
| GENXPRO GMBH | 1 | 0.033 |
| GENOUEST | 1 | 0.033 |
| GENOMNIA SRL | 1 | 0.033 |
| GENOMEDX BIOSCI INC | 1 | 0.033 |
| GENOMEDX BIOSCI | 1 | 0.033 |
| GENOME QUEBEC INNOVAT CTR | 1 | 0.033 |
| GENOMATIX SOFTWARE GMBH | 1 | 0.033 |
| GENOLUTION INC | 1 | 0.033 |
| GENENTECH INC | 1 | 0.033 |
| GEN STAFF DEPT CHINESE PEOPLES LIBERAT ARMY | 1 | 0.033 |
| GEN HOSP PLA | 1 | 0.033 |
| GEN HOSP PEOPLES LIBERAT ARMY | 1 | 0.033 |
| GEN HOSP CHINESE PLA | 1 | 0.033 |
| GEISEL SCH MED DARTMOUTH | 1 | 0.033 |
| GBPA | 1 | 0.033 |
| GAOZHOU PEOPLES HOSP | 1 | 0.033 |
| GAOMINGHESHUI HOSP | 1 | 0.033 |
| GANNAN MED UNIV | 1 | 0.033 |
| FUZHOU GEN HOSP NANJING MIL AREA | 1 | 0.033 |
| FUYONG PEOPLES HOSP | 1 | 0.033 |
| FUNDACAO CTR UNIV ESTADUAL ZONA OESTE | 1 | 0.033 |
| FUKUSHIMA MED UNIV | 1 | 0.033 |
| FUKUOKA UNIV | 1 | 0.033 |
| FUKUOKA NATL HOSP | 1 | 0.033 |
| FUJITA HLTH UNIV | 1 | 0.033 |
| FUJIAN PROV KEY LAB TRANSLAT CANC MED | 1 | 0.033 |
| FUJIAN PROV KEY LAB CHRON LIVER DIS HEPATOCELLU | 1 | 0.033 |
| FUJIAN PROV HOSP | 1 | 0.033 |
| FUJIAN PROV CANC HOSP | 1 | 0.033 |
| FUJIAN JIANOU HOSP | 1 | 0.033 |
| FUDAN UNIV SHANGHAI CANC CTR | 1 | 0.033 |
| FRIEDRICH MIESCHER INST BIOMED RES | 1 | 0.033 |
| FRIEDRICH EBERT HOSP NEUMUNSTER | 1 | 0.033 |
| FREDERICK NATL LAB CANC RES | 1 | 0.033 |
| FRANCIS CRICK INST | 1 | 0.033 |
| FOURTH PEOPLES HOSP JINAN | 1 | 0.033 |
| FOURTH PEOPLES HOSP | 1 | 0.033 |
| FOURTH HOSP NINGBO | 1 | 0.033 |
| FORSCHUNGSZENTRUM JULICH | 1 | 0.033 |
| FOOD IND RES DEV INST | 1 | 0.033 |
| FLORIDA UROL ASSOCIATES | 1 | 0.033 |
| FLORIDA INT UNIV | 1 | 0.033 |
| FLORIDA HOSP ORLANDO | 1 | 0.033 |
| FLORIDA HOSP CELEBRAT HLTH | 1 | 0.033 |
| FLORIDA HOSP | 1 | 0.033 |
| FLINDERS UNIV S AUSTRALIA | 1 | 0.033 |
| FLINDERS UNIV FMC | 1 | 0.033 |
| FLINDERS MED CTR | 1 | 0.033 |
| FLEVOZIEKENHUIS | 1 | 0.033 |
| FLANDERS INST BIOTECHNOL VIB | 1 | 0.033 |
| FIRST PEOPLES HOSP YUNNAN | 1 | 0.033 |
| FIRST PEOPLES HOSP TAIAN | 1 | 0.033 |
| FIRST PEOPLES HOSP HEFEI | 1 | 0.033 |
| FIRST HOSP ZIBO CITY | 1 | 0.033 |
| FIRST HOSP WUHAN | 1 | 0.033 |
| FIRST HOSP QINHUANGDAO | 1 | 0.033 |
| FIRST HOSP HARBIN CITY | 1 | 0.033 |
| FIRST CENT HOSP TIANJIN | 1 | 0.033 |
| FIFTH PEOPLES HOSP CHENGDU | 1 | 0.033 |
| FERRAROTTO HOSP | 1 | 0.033 |
| FENGCHENG HOSP | 1 | 0.033 |
| FEICHENG MIN IND CENT HOSP | 1 | 0.033 |
| FED INST TECHNOL EPFL | 1 | 0.033 |
| FDN RES TECHNOL HELLAS | 1 | 0.033 |
| FDN RES TECHNOL | 1 | 0.033 |
| FDN IRCCS SDN | 1 | 0.033 |
| FDN IRCCS CA GRANDA | 1 | 0.033 |
| FDN DON CARLO GNOCCHI | 1 | 0.033 |
| FDN CIENCIA VIDA | 1 | 0.033 |
| FAMILY PLANNING RES INST HUNAN PROV | 1 | 0.033 |
| FAMILY PLANNING INST HUNAN PROV | 1 | 0.033 |
| FAC MED LILLE | 1 | 0.033 |
| FAC MED 1 | 1 | 0.033 |
| EYE INST XUZHOU | 1 | 0.033 |
| EXPT CLIN RES CTR ECRC | 1 | 0.033 |
| EUROPEAN SCH MOL MED | 1 | 0.033 |
| EUROPEAN INST ONCOL | 1 | 0.033 |
| EUROPEAN BIOINFORMAT INST | 1 | 0.033 |
| EUPHERIA BIOTECH GMBH | 1 | 0.033 |
| ETH | 1 | 0.033 |
| ERASMUS UNIV MED | 1 | 0.033 |
| ERASMUS UNIV | 1 | 0.033 |
| ERASMUS MC SOPHIA | 1 | 0.033 |
| EQUIPE LABELLISEE LIGUE NATL CONTRE CANC | 1 | 0.033 |
| ENGN KEY LAB CELL THERAPY HENAN PROV | 1 | 0.033 |
| EMORY UNIV | 1 | 0.033 |
| EMBL HEIDELBERG | 1 | 0.033 |
| EMBL EUROPEAN BIOINFORMAT INST | 1 | 0.033 |
| EMBL EBI | 1 | 0.033 |
| EMBL | 1 | 0.033 |
| ELI EDYTHE L BROAD INST | 1 | 0.033 |
| ELI EDYTHE BROAD CTR REGENERAT MED STEM CELL | 1 | 0.033 |
| EIGHTH PEOPLES HOSP SHANGHAI | 1 | 0.033 |
| EGE UNIV | 1 | 0.033 |
| ECSEQ BIOINFORMAT | 1 | 0.033 |
| ECOLE NORMALE SUPER | 1 | 0.033 |
| EBI | 1 | 0.033 |
| EASTERN COOPERAT ONCOL GRP | 1 | 0.033 |
| E TENNESSEE STATE UNIV | 1 | 0.033 |
| E CHINA NORMAL UNIV | 1 | 0.033 |
| DZHK GERMAN CTR CARDIOVASC RES | 1 | 0.033 |
| DURHAM VA MED CTR | 1 | 0.033 |
| DUKE NUS | 1 | 0.033 |
| DUKE NATL UNIV SINGAPORE | 1 | 0.033 |
| DREXEL UNIV | 1 | 0.033 |
| DR MARGARETE FISCHER BOSCH INST CLIN PHARMACOL | 1 | 0.033 |
| DOUGLAS HOSP | 1 | 0.033 |
| DONGFANG HOSP | 1 | 0.033 |
| DONG A UNIV | 1 | 0.033 |
| DONDERS INST BRAIN COGNIT BEHAV | 1 | 0.033 |
| DKU THERAGEN INST NGS ANAL DTINA | 1 | 0.033 |
| DKFZ GERMAN CANC RES CTR | 1 | 0.033 |
| DFG RES CTR | 1 | 0.033 |
| DEZHOU PEOPLES HOSP | 1 | 0.033 |
| DEPT VET AFFAIRS MED CTR | 1 | 0.033 |
| DEPT NEUROSCI | 1 | 0.033 |
| DEPT NEUROL | 1 | 0.033 |
| DEPT MED | 1 | 0.033 |
| DEPT INTERNAL MED | 1 | 0.033 |
| DEPT CLIN PATHOL | 1 | 0.033 |
| DEPT CLIN ONCOL | 1 | 0.033 |
| DEPT CELLULAR MOL PHARMACOL | 1 | 0.033 |
| DENT HOSP YANTAI CITY | 1 | 0.033 |
| DENT CLIN XUHUI DIST | 1 | 0.033 |
| DEHUA CTY HOSP | 1 | 0.033 |
| DECODE GENET | 1 | 0.033 |
| DAVID H KOCH INST INTEGRAT CANC RES | 1 | 0.033 |
| DARTMOUTH COLL | 1 | 0.033 |
| DAQING OILFIELD GEN HOSP | 1 | 0.033 |
| DAQING LONGNAN HOSP | 1 | 0.033 |
| DANKOOK UNIV | 1 | 0.033 |
| DAIRY FUTURES COOPERAT RES CTR | 1 | 0.033 |
| DAIICHI SANKYO CO LTD | 1 | 0.033 |
| CZECH UNIV LIFE SCI | 1 | 0.033 |
| CUNY QUEENSBOROUGH COMMUNITY COLL | 1 | 0.033 |
| CUNY | 1 | 0.033 |
| CUHK SHENZHEN RES INST | 1 | 0.033 |
| CUHK | 1 | 0.033 |
| CU SYST MED | 1 | 0.033 |
| CTR RNA SYST BIOL | 1 | 0.033 |
| CTR REGENERAT MED STEM CELL RES | 1 | 0.033 |
| CTR REG HEMOTERAPIA RIBEIRAO PRETO | 1 | 0.033 |
| CTR RECH PUBL SANTE CRP SANTE | 1 | 0.033 |
| CTR NACL PESQUISA ENERGIA MAT | 1 | 0.033 |
| CTR INVEST PRINCIPE FELIPE | 1 | 0.033 |
| CTR INVEST ESTUDIOS AVANZADOS INST POLITECN NAC | 1 | 0.033 |
| CTR INTEGRAT SYST BIOL CISBI NAP USP | 1 | 0.033 |
| CTR HOSP REG UNIV LILLE | 1 | 0.033 |
| CTR GENOM REGULAT CRG UPF | 1 | 0.033 |
| CTR DIS CONTROL PREVENT NANTONG | 1 | 0.033 |
| CTR DIS CONTROL PREVENT GUANGDONG PROV | 1 | 0.033 |
| CTR COMPREHENS CANC | 1 | 0.033 |
| CTR BIOL DIS | 1 | 0.033 |
| CTR APPL MED RES CIMA | 1 | 0.033 |
| CSIRO PREVENTAT HLTH FLAGSHIP | 1 | 0.033 |
| CSIRO | 1 | 0.033 |
| CSIR INST MICROBIAL TECHNOL | 1 | 0.033 |
| CSIR IGIB SOUTH CAMPUS | 1 | 0.033 |
| CSIC ICCC | 1 | 0.033 |
| CRS4 BIOINFORMAT LAB | 1 | 0.033 |
| CRP SANTE | 1 | 0.033 |
| CRANFIELD UNIV | 1 | 0.033 |
| COVANCE INC | 1 | 0.033 |
| COUNCIL AGR | 1 | 0.033 |
| CORP SANITARIA PARC TAULI UNIV INST UAB BARCELONA | 1 | 0.033 |
| CONSTELLAT PHARMACEUT | 1 | 0.033 |
| COMPREHENS CANC CTR MAINFRANKEN | 1 | 0.033 |
| COMPLEJO HOSP UNIV SANTIAGO | 1 | 0.033 |
| COMPLEJO HOSP NAVARRA | 1 | 0.033 |
| COMPARAT MED CTR | 1 | 0.033 |
| COMMONWEALTH SCI IND RES ORG PLANT IND | 1 | 0.033 |
| COMMONWEALTH SCI IND RES ORG | 1 | 0.033 |
| COMENIUS UNIV | 1 | 0.033 |
| COLUMBIA MAILMAN SCH PUBL HLTH | 1 | 0.033 |
| COLOGNE EXCELLENCE CLUSTER CELLULAR STRESS RESPON | 1 | 0.033 |
| COLLABORAT INNOVAT CTR DIAG TREATMENT INFECT DI | 1 | 0.033 |
| COLLABORAT INNOVAT CTR BIOTHERAPY | 1 | 0.033 |
| COLL NAT SCI | 1 | 0.033 |
| CNRS URA 2581 | 1 | 0.033 |
| CNRS 5286 | 1 | 0.033 |
| CMC BIOBANK TRANSLAT MED INST | 1 | 0.033 |
| CLUSTER EXCELLENCE REGENERAT THERAPIES | 1 | 0.033 |
| CLUSTER EXCELLENCE FRANKFURT MACROMOL COMPLEXES F | 1 | 0.033 |
| CLIN VET ST CYR | 1 | 0.033 |
| CLIN VET ST BERNARD | 1 | 0.033 |
| CLIN INDISA | 1 | 0.033 |
| CLIN GENOM PTY LTD | 1 | 0.033 |
| CLEVELAND CLIN FDN | 1 | 0.033 |
| CITY UNIV HONG KONG | 1 | 0.033 |
| CITY HOPE BECKMAN RES INST | 1 | 0.033 |
| CINN CSIC | 1 | 0.033 |
| CINCINNATI CHILDRENS HOSP | 1 | 0.033 |
| CIMA | 1 | 0.033 |
| CIEMAT ED70A | 1 | 0.033 |
| CIBER ENFERMEDADES RESP ISCIII | 1 | 0.033 |
| CHUNGBUK NATL UNIV | 1 | 0.033 |
| CHUNG SHAN MED UNIV HOSP | 1 | 0.033 |
| CHUNG SHAN MED UNIV | 1 | 0.033 |
| CHUNG ANG UNIV | 1 | 0.033 |
| CHU | 1 | 0.033 |
| CHRON LYMPHOCYT LEUKEMIA RES CONSORTIUM | 1 | 0.033 |
| CHRISTIAN MED COLL HOSP | 1 | 0.033 |
| CHONNAM NATL UNIV | 1 | 0.033 |
| CHONGQING UNIV | 1 | 0.033 |
| CHONGQING ENGN RES CTR GOATS | 1 | 0.033 |
| CHONGQING ACAD ANIM SCI | 1 | 0.033 |
| CHONGQIN MED UNIV | 1 | 0.033 |
| CHINESE PEOPLES ARMED POLICE FORCE | 1 | 0.033 |
| CHINESE MINIST HLTH | 1 | 0.033 |
| CHINESE ACAD FORESTRY | 1 | 0.033 |
| CHINA THREE GORGES UNIV | 1 | 0.033 |
| CHINA ORTHOPED REGENERAT MED GRP CORMED | 1 | 0.033 |
| CHINA ASTRONAUT RES TRAINING CTR | 1 | 0.033 |
| CHILDRENS MEM HOSP | 1 | 0.033 |
| CHILDRENS HOSP WESTMEAD | 1 | 0.033 |
| CHILDRENS HOSP | 1 | 0.033 |
| CHILDREN WOMENS HEALTHCARE LAIWU CITY | 1 | 0.033 |
| CHICOUTIMI HOSP | 1 | 0.033 |
| CHENZHOU FIRST PEOPLES HOSP | 1 | 0.033 |
| CHENGDU UNIV TRADIT CHINESE MED | 1 | 0.033 |
| CHENGDU UNIV TCM | 1 | 0.033 |
| CHENDU MIL COMMAND | 1 | 0.033 |
| CHEM GENOM CTR | 1 | 0.033 |
| CHARLES UNIV PRAGUE | 1 | 0.033 |
| CHANGZHOU 2 PEOPLES HOSP | 1 | 0.033 |
| CHANGZHENG HOSP | 1 | 0.033 |
| CHANGSHA MED UNIV | 1 | 0.033 |
| CHANGSHA CITY CENT HOSP | 1 | 0.033 |
| CHANGHUA CHRISTIAN HOSP | 1 | 0.033 |
| CHANG GUNG UNIV SCI TECHNOL | 1 | 0.033 |
| CHAMINADE UNIV HONOLULU | 1 | 0.033 |
| CGCG | 1 | 0.033 |
| CGAT UNIV OXFORD | 1 | 0.033 |
| CENT RES INST EPIDEMIOL | 1 | 0.033 |
| CENT HOSP ZIBO | 1 | 0.033 |
| CENT HOSP SHAOYANG CITY | 1 | 0.033 |
| CENT HOSP PANYU DIST | 1 | 0.033 |
| CENT HOSP MINHANG DIST | 1 | 0.033 |
| CENT HOSP CHENGDE | 1 | 0.033 |
| CENT HOSP CANGZHOU CITY | 1 | 0.033 |
| CENT HOSP | 1 | 0.033 |
| CENT ARKANSAS VET HEALTHCARE SYST | 1 | 0.033 |
| CELLULAR BIOMED GRP INC | 1 | 0.033 |
| CELL GUIDANCE SYST | 1 | 0.033 |
| CEA | 1 | 0.033 |
| CATHOLIC UNIV KOREA | 1 | 0.033 |
| CATHOLIC UNIV DAEGU | 1 | 0.033 |
| CATHOLIC UNIV | 1 | 0.033 |
| CATHOLIC CLIN RUHR PENINSULA | 1 | 0.033 |
| CATALAN INST RES ADV STUDIES | 1 | 0.033 |
| CATALAN INST ONCOL ICO | 1 | 0.033 |
| CAS MPG PARTNER INST COMPUTAT BIOL | 1 | 0.033 |
| CARNEGIE INST SCI | 1 | 0.033 |
| CARLETON UNIV | 1 | 0.033 |
| CARLE CANC CTR | 1 | 0.033 |
| CARDIFF UNIV | 1 | 0.033 |
| CANC RES INST GHENT | 1 | 0.033 |
| CANC HOSP JIANGXI PROV | 1 | 0.033 |
| CANC HOSP JIANGSU PROV | 1 | 0.033 |
| CANADIAN CANC TRIALS GRP | 1 | 0.033 |
| CAMS | 1 | 0.033 |
| CALIF INST QUANTITAT BIOMED RES | 1 | 0.033 |
| BUDDHIST TZU CHI MED FDN | 1 | 0.033 |
| BSGENOMICS CO LTD | 1 | 0.033 |
| BROWN UNIV | 1 | 0.033 |
| BROWN FDN INST MOL MED PREVENT HUMAN DIS | 1 | 0.033 |
| BROTZU HOSP | 1 | 0.033 |
| BROAD INST HARVARD MASSACHUSETTS INST TECHNOL M | 1 | 0.033 |
| BRITISH HEART FDN GLASGOW | 1 | 0.033 |
| BRITISH COLUMBIA CANC RES CTR | 1 | 0.033 |
| BRIGHAM YOUNG UNIV | 1 | 0.033 |
| BOTOU HOSP | 1 | 0.033 |
| BOSTON COLL | 1 | 0.033 |
| BOSE INST | 1 | 0.033 |
| BOISE STATE UNIV | 1 | 0.033 |
| BIRMINGHAM VET AFFAIRS MED CTR | 1 | 0.033 |
| BIRLA INST TECHNOL SCI PILANI | 1 | 0.033 |
| BIOORGAN RES INST | 1 | 0.033 |
| BIONEER AS | 1 | 0.033 |
| BIOMERIEUX SHANGHAI CO LTD | 1 | 0.033 |
| BIOMED SCI RES CTR ALEXANDER FLEMING | 1 | 0.033 |
| BIOKER SRL MULTIMED SPA NAPLES | 1 | 0.033 |
| BIOINFORMAT INST | 1 | 0.033 |
| BIOIND PK SILVANO FUMERO | 1 | 0.033 |
| BIOGEN IDEC INC | 1 | 0.033 |
| BIOGAZELLE | 1 | 0.033 |
| BIODONOSTIA RES INST | 1 | 0.033 |
| BINZHOU PEOPLES HOSP | 1 | 0.033 |
| BINZHOU MED SCH | 1 | 0.033 |
| BGI TECH SOLUT CO LTD | 1 | 0.033 |
| BGI HK RES INST | 1 | 0.033 |
| BGI GUANGZHOU | 1 | 0.033 |
| BEZMIALEM VAKIF GUREBA UNIV | 1 | 0.033 |
| BETHUNE MIL MED COLL | 1 | 0.033 |
| BETHUNE INT PEACE HOSP | 1 | 0.033 |
| BERNARDO OHIGGINS UNIV | 1 | 0.033 |
| BENI SUEF UNIV | 1 | 0.033 |
| BENGBU MED SCH | 1 | 0.033 |
| BENGBU MED COLL BENGBU | 1 | 0.033 |
| BELGIAN CANC REGISTRY | 1 | 0.033 |
| BEIJING NORMAL UNIV | 1 | 0.033 |
| BEIJING MIL REG GEN HOSP | 1 | 0.033 |
| BEIJING KEY LAB TRANSLAT MED CEREBROVASC DIS | 1 | 0.033 |
| BEIJING KEY LAB HEAD NECK MOL DIAGNOST PATHOL | 1 | 0.033 |
| BEIJING KEY LAB DIGITAL STOMATOL | 1 | 0.033 |
| BEIJING INST BRAIN DISORDERS | 1 | 0.033 |
| BEIJING INST BASIC MED SCI | 1 | 0.033 |
| BEIJING HUILONGGUAN HOSP | 1 | 0.033 |
| BEIJING HOSP | 1 | 0.033 |
| BEIJING GENOM INST SHENZHEN BGI SZ | 1 | 0.033 |
| BEIJING GENOM INST SHENZHEN | 1 | 0.033 |
| BEIJING GENOM INST | 1 | 0.033 |
| BEIJING ENGN RES CTR VASC PROSTHESES | 1 | 0.033 |
| BEIJING CHUIYANGLIU HOSP | 1 | 0.033 |
| BECKMAN RES INST CITY HOPE | 1 | 0.033 |
| BEAUMONT HOSP | 1 | 0.033 |
| BC CANC AGCY CANC RES CTR | 1 | 0.033 |
| BC CANC AGCY | 1 | 0.033 |
| BAYLOR UNIV | 1 | 0.033 |
| BAYLOR COLL MED MSTP | 1 | 0.033 |
| BAYLOR BREAST CTR | 1 | 0.033 |
| BASQUE FDN SCI | 1 | 0.033 |
| BARRETOS CANC HOSP | 1 | 0.033 |
| BAOTOU MED COLL | 1 | 0.033 |
| BAOSHAN DIST SHANGHAI INTEGRATED TRADIT CHINESE | 1 | 0.033 |
| BAOJI PEOPLES HOSP | 1 | 0.033 |
| BANKSTOWN HOSP | 1 | 0.033 |
| BAMBINO GESU PEDIAT HOSP | 1 | 0.033 |
| BAKERIDI HEART DIABET INST | 1 | 0.033 |
| AZIENDA OSPED UNIV SAN MARTINO IST | 1 | 0.033 |
| AZIENDA OSPED PERUGIA | 1 | 0.033 |
| AUTOBIO DIAGNOST CO LTD | 1 | 0.033 |
| AUSTRALIAN STEM CELL CTR | 1 | 0.033 |
| AUSTRALIAN CTR NANOMED | 1 | 0.033 |
| AUSTIN HOSP | 1 | 0.033 |
| AUGUSTA UNIV | 1 | 0.033 |
| ATHENA RES CTR | 1 | 0.033 |
| ASTAR | 1 | 0.033 |
| ASSOCIATED MED PROFESS NEW YORK | 1 | 0.033 |
| ASIA UNIV | 1 | 0.033 |
| ASCR | 1 | 0.033 |
| ASAHI GEN HOSP | 1 | 0.033 |
| ARMY GEN HOSP PLA | 1 | 0.033 |
| ARMED FORCES COLL MED | 1 | 0.033 |
| ARCTURUS | 1 | 0.033 |
| APPL BIOSYST INC | 1 | 0.033 |
| AOYANG HOSP | 1 | 0.033 |
| AO COSENZA | 1 | 0.033 |
| AO ASP COSENZA | 1 | 0.033 |
| ANUSANDHAN BHAWAN | 1 | 0.033 |
| ANKANG HOSP TRADIT CHINESE MED | 1 | 0.033 |
| ANKANG CENT HOSP | 1 | 0.033 |
| ANHUI UNIV TRADIT CHINESE MED | 1 | 0.033 |
| ANHUI UNIV CHINESE MED | 1 | 0.033 |
| ANHUI PROV HOSP | 1 | 0.033 |
| ANHUI AGR UNIV | 1 | 0.033 |
| ANDES BIOTECHNOL AS | 1 | 0.033 |
| AMU | 1 | 0.033 |
| ALBERTO SOLS UNIV AUTONOMA MADRID | 1 | 0.033 |
| ALBERTO SOLS CSIC UAM | 1 | 0.033 |
| ALBANY MED COLL | 1 | 0.033 |
| AL QUDS UNIV | 1 | 0.033 |
| AKITA RED CROSS HOSP | 1 | 0.033 |
| AKERSHUS UNIV HOSP | 1 | 0.033 |
| AJOU UNIV | 1 | 0.033 |
| AIX MARSEILLE UNIV | 1 | 0.033 |
| AIR FORCE GEN HOSP | 1 | 0.033 |
| AIN SHAMS UNIV HOSP | 1 | 0.033 |
| AICHI GAKUIN UNIV | 1 | 0.033 |
| AGROPARISTECH | 1 | 0.033 |
| AGRIBIO BUNDOORA | 1 | 0.033 |
| AGILENT LABS | 1 | 0.033 |
| AFFILIATED JIANGRNEN HOSP SUN YAT SEN | 1 | 0.033 |
| AFFILIATED HOSP NINGBO UNIV | 1 | 0.033 |
| AFFILIATED HOSP NANJING MED UNIV | 1 | 0.033 |
| AFFILIATED HOSP INNER MONGOLIA UNIV NATIONALITIES | 1 | 0.033 |
| AFFILIATED HOSP HANGZHOU NORMAL UNIV | 1 | 0.033 |
| ACQUIFER AG | 1 | 0.033 |
| ACECR | 1 | 0.033 |
| ACAD SCI CZECH REPUBLIC | 1 | 0.033 |
| ACAD ATHENS | 1 | 0.033 |
| ABO AKAD UNIV | 1 | 0.033 |
| AARHUS UNIV HOSP | 1 | 0.033 |
| AALTO UNIV | 1 | 0.033 |
| AALBORG UNIV CAMPUS COPENHAGEN | 1 | 0.033 |
| AALBORG UNIV | 1 | 0.033 |
| A STAR INST MED BIOL | 1 | 0.033 |
| 81ST HOSP PLA | 1 | 0.033 |
| 4TH PEOPLES HOSP WUXI | 1 | 0.033 |
| 452ND HOSP PLA | 1 | 0.033 |
| 451TH HOSP PEOPLES LIBERAT ARMY | 1 | 0.033 |
| 451ST HOSP PEOPLES LIBERAT ARMY | 1 | 0.033 |
| 323RD HOSP PEOPLES LIBERAT ARMY | 1 | 0.033 |
| 3 HOSP JINAN | 1 | 0.033 |
| 202 HOSP PLA | 1 | 0.033 |
| 2 PEOPLES HOSP FUYANG | 1 | 0.033 |
| 1ST RENMIN HOSP SHANGQIU | 1 | 0.033 |
| 181 HOSP | 1 | 0.033 |
| 161ST HOSP PLA | 1 | 0.033 |
| 153 HOSP LIBERAT ARMY | 1 | 0.033 |
| 148 CENT HOSP PLA | 1 | 0.033 |
| 107 P L HOSP | 1 | 0.033 |
| 102 HOSP CHINESE PEOPLES LIBERAT ARMY | 1 | 0.033 |
| 102 HOSP CHINESE PEOPLES LIBERAT | 1 | 0.033 |
| 1 PEOPLES HOSP YUEYANG CITY | 1 | 0.033 |
| 1 PEOPLES HOSP XUZHOU | 1 | 0.033 |
| (2115 Organizations {0} {1} value(s) outside display options.) | | |
| (1 records (0.033%){0} records{1} do not contain data in the field being analyzed.) | | |
