# Supplementary material for: Trends of long noncoding RNA research from 2007 to 2016: a bibliometric analysis
Source: Oncotarget. 2017 Sep 12;8(47):83114–27. doi: 10.18632/oncotarget.20851 (PMC5669954; doi:10.18632/oncotarget.20851)
Supplement: Supplementary file 5 [file oncotarget-08-83114-s005.docx]

**Supplementary Table 4: Raw data on authors involved in lncRNA publications extracted from Web of Science Core Collection**

| **Authors** | **records** | **% of 3008** | **Authors** | **records** | **% of 3008** |
| --- | --- | --- | --- | --- | --- |
| ZHANG Y | 72 | 2.394 | SHEN YF | 1 | 0.033 |
| WANG Y | 67 | 2.227 | SHEN XL | 1 | 0.033 |
| WANG J | 63 | 2.094 | SHEN XK | 1 | 0.033 |
| LI J | 60 | 1.995 | SHEN XJ | 1 | 0.033 |
| LI X | 58 | 1.928 | SHEN WS | 1 | 0.033 |
| ZHANG L | 56 | 1.862 | SHEN TT | 1 | 0.033 |
| ZHANG J | 49 | 1.629 | SHEN SL | 1 | 0.033 |
| LI Y | 47 | 1.563 | SHEN SK | 1 | 0.033 |
| WANG L | 41 | 1.363 | SHEN S | 1 | 0.033 |
| LIU Y | 37 | 1.23 | SHEN RZ | 1 | 0.033 |
| LIU L | 36 | 1.197 | SHEN QS | 1 | 0.033 |
| SUN M | 35 | 1.164 | SHEN LQ | 1 | 0.033 |
| YANG L | 34 | 1.13 | SHEN LP | 1 | 0.033 |
| ZHAO Y | 33 | 1.097 | SHEN LJ | 1 | 0.033 |
| CHANG HY | 33 | 1.097 | SHEN KW | 1 | 0.033 |
| WANG P | 32 | 1.064 | SHEN K | 1 | 0.033 |
| ZHANG X | 31 | 1.031 | SHEN HY | 1 | 0.033 |
| DE W | 31 | 1.031 | SHEN H | 1 | 0.033 |
| WANG F | 30 | 0.997 | SHEN F | 1 | 0.033 |
| LI W | 30 | 0.997 | SHEN ED | 1 | 0.033 |
| CHEN J | 30 | 0.997 | SHEN CX | 1 | 0.033 |
| LIU J | 29 | 0.964 | SHEN CT | 1 | 0.033 |
| LI M | 29 | 0.964 | SHEN CQ | 1 | 0.033 |
| XU J | 28 | 0.931 | SHEN CL | 1 | 0.033 |
| LI H | 28 | 0.931 | SHEN AJ | 1 | 0.033 |
| ZHANG H | 27 | 0.898 | SHELTON JM | 1 | 0.033 |
| YANG F | 27 | 0.898 | SHEHTA M | 1 | 0.033 |
| LI L | 27 | 0.898 | SHEARWOOD AMJ | 1 | 0.033 |
| CHEN Y | 27 | 0.898 | SHEARER J | 1 | 0.033 |
| YANG Y | 26 | 0.864 | SHE L | 1 | 0.033 |
| RINN JL | 25 | 0.831 | SHE KL | 1 | 0.033 |
| ZHOU M | 23 | 0.765 | SHCHERBININA EY | 1 | 0.033 |
| WANG H | 23 | 0.765 | SHAVLAKADZE T | 1 | 0.033 |
| DINGER ME | 23 | 0.765 | SHASHATY MGS | 1 | 0.033 |
| CHEN X | 23 | 0.765 | SHARP FR | 1 | 0.033 |
| CHEN W | 23 | 0.765 | SHARMA V | 1 | 0.033 |
| YAO J | 22 | 0.731 | SHARMA N | 1 | 0.033 |
| MATTICK JS | 22 | 0.731 | SHAO ZY | 1 | 0.033 |
| WANG Q | 21 | 0.698 | SHAO ZM | 1 | 0.033 |
| HAN L | 21 | 0.698 | SHAO ZF | 1 | 0.033 |
| CHEN L | 21 | 0.698 | SHAO YB | 1 | 0.033 |
| WANG M | 20 | 0.665 | SHAO X | 1 | 0.033 |
| SUN J | 20 | 0.665 | SHAO W | 1 | 0.033 |
| LIU B | 20 | 0.665 | SHAO TT | 1 | 0.033 |
| LI Z | 20 | 0.665 | SHAO SH | 1 | 0.033 |
| ZHANG EB | 19 | 0.632 | SHAO QX | 1 | 0.033 |
| XU Y | 19 | 0.632 | SHAO QP | 1 | 0.033 |
| LIU F | 19 | 0.632 | SHAO Q | 1 | 0.033 |
| LI F | 19 | 0.632 | SHAO NY | 1 | 0.033 |
| GAO Y | 19 | 0.632 | SHAO N | 1 | 0.033 |
| XU TP | 18 | 0.598 | SHAO MR | 1 | 0.033 |
| XIA R | 18 | 0.598 | SHAO MM | 1 | 0.033 |
| WANG X | 18 | 0.598 | SHAO M | 1 | 0.033 |
| PONTING CP | 18 | 0.598 | SHAO LP | 1 | 0.033 |
| LIU YC | 18 | 0.598 | SHAO L | 1 | 0.033 |
| LI Q | 18 | 0.598 | SHAO HY | 1 | 0.033 |
| GUO JM | 18 | 0.598 | SHAO H | 1 | 0.033 |
| CHEN RS | 18 | 0.598 | SHAO F | 1 | 0.033 |
| CHEN H | 18 | 0.598 | SHAO CS | 1 | 0.033 |
| LIU YH | 17 | 0.565 | SHAO CG | 1 | 0.033 |
| ZHANG F | 16 | 0.532 | SHAO AZ | 1 | 0.033 |
| XU L | 16 | 0.532 | SHANI N | 1 | 0.033 |
| XU H | 16 | 0.532 | SHANG XS | 1 | 0.033 |
| LIU T | 16 | 0.532 | SHANG XL | 1 | 0.033 |
| LI ZH | 16 | 0.532 | SHANG RZ | 1 | 0.033 |
| ZHANG YY | 15 | 0.499 | SHANG QK | 1 | 0.033 |
| XUE Y | 15 | 0.499 | SHANG MH | 1 | 0.033 |
| WU Y | 15 | 0.499 | SHANG JL | 1 | 0.033 |
| WU J | 15 | 0.499 | SHANG FS | 1 | 0.033 |
| WANG SY | 15 | 0.499 | SHANG DH | 1 | 0.033 |
| SHEN Y | 15 | 0.499 | SHANG CL | 1 | 0.033 |
| MA J | 15 | 0.499 | SHAN ZX | 1 | 0.033 |
| LIU HB | 15 | 0.499 | SHAN YX | 1 | 0.033 |
| LI P | 15 | 0.499 | SHAN Y | 1 | 0.033 |
| LI C | 15 | 0.499 | SHAN DK | 1 | 0.033 |
| KONG R | 15 | 0.499 | SHAN CY | 1 | 0.033 |
| ZHOU L | 14 | 0.465 | SHAN BE | 1 | 0.033 |
| ZHOU H | 14 | 0.465 | SHAMSUDHEEN KV | 1 | 0.033 |
| ZHANG W | 14 | 0.465 | SHALCHIAN-TABRIZI K | 1 | 0.033 |
| ZHANG K | 14 | 0.465 | SHAKHOVA O | 1 | 0.033 |
| YUAN J | 14 | 0.465 | SHAHRYARI A | 1 | 0.033 |
| WANG YY | 14 | 0.465 | SHAHAM L | 1 | 0.033 |
| WANG K | 14 | 0.465 | SHAH N | 1 | 0.033 |
| SUN SH | 14 | 0.465 | SHAH M | 1 | 0.033 |
| SUN L | 14 | 0.465 | SHAH A | 1 | 0.033 |
| SCARIA V | 14 | 0.465 | SHAFIQ S | 1 | 0.033 |
| NAKAGAWA S | 14 | 0.465 | SHAFIK A | 1 | 0.033 |
| LIU Q | 14 | 0.465 | SHAFFER LG | 1 | 0.033 |
| LIU C | 14 | 0.465 | SHA ML | 1 | 0.033 |
| HE J | 14 | 0.465 | SEYFODDIN V | 1 | 0.033 |
| GOROSPE M | 14 | 0.465 | SEXTON AN | 1 | 0.033 |
| CHEN T | 14 | 0.465 | SETOYAMA T | 1 | 0.033 |
| CHEN LL | 14 | 0.465 | SETIEN F | 1 | 0.033 |
| CALIN GA | 14 | 0.465 | SETHURAMAN S | 1 | 0.033 |
| ZHOU Y | 13 | 0.432 | SESTILI P | 1 | 0.033 |
| ZHOU XY | 13 | 0.432 | SERVISS JT | 1 | 0.033 |
| ZHOU X | 13 | 0.432 | SERGHIOU S | 1 | 0.033 |
| ZHOU Q | 13 | 0.432 | SERFECZ J | 1 | 0.033 |
| ZHANG XQ | 13 | 0.432 | SEPRAMANIAM S | 1 | 0.033 |
| ZHANG Q | 13 | 0.432 | SEPPALA J | 1 | 0.033 |
| YU B | 13 | 0.432 | SEO S | 1 | 0.033 |
| YIN R | 13 | 0.432 | SENTER L | 1 | 0.033 |
| YANG Z | 13 | 0.432 | SENSEN CW | 1 | 0.033 |
| YANG J | 13 | 0.432 | SENERGIN HB | 1 | 0.033 |
| YAN B | 13 | 0.432 | SENDOREK DH | 1 | 0.033 |
| WANG ZH | 13 | 0.432 | SEN R | 1 | 0.033 |
| WANG Z | 13 | 0.432 | SEMNANI S | 1 | 0.033 |
| WANG W | 13 | 0.432 | SELZMAN CH | 1 | 0.033 |
| WANG T | 13 | 0.432 | SELIMYAN R | 1 | 0.033 |
| SUN Y | 13 | 0.432 | SELES M | 1 | 0.033 |
| SHU YQ | 13 | 0.432 | SEKIMIZU K | 1 | 0.033 |
| MORRIS KV | 13 | 0.432 | SEIM I | 1 | 0.033 |
| LIU XH | 13 | 0.432 | SEGOVIA C | 1 | 0.033 |
| LIU N | 13 | 0.432 | SEGAL E | 1 | 0.033 |
| LIU H | 13 | 0.432 | SEET CS | 1 | 0.033 |
| LIPOVICH L | 13 | 0.432 | SEEMANN SE | 1 | 0.033 |
| LI XM | 13 | 0.432 | SEEGER R | 1 | 0.033 |
| LI XL | 13 | 0.432 | SEDLAK N | 1 | 0.033 |
| LI T | 13 | 0.432 | SEBIRE N | 1 | 0.033 |
| LI S | 13 | 0.432 | SCURRY J | 1 | 0.033 |
| GUO Y | 13 | 0.432 | SCOTTON C | 1 | 0.033 |
| CHEN G | 13 | 0.432 | SCOTT JAG | 1 | 0.033 |
| ZHOU CC | 12 | 0.399 | SCOTT E | 1 | 0.033 |
| ZHANG ZY | 12 | 0.399 | SCORILAS A | 1 | 0.033 |
| ZHANG XJ | 12 | 0.399 | SCHWER B | 1 | 0.033 |
| ZHANG JJ | 12 | 0.399 | SCHWARZOVA L | 1 | 0.033 |
| YANG M | 12 | 0.399 | SCHWARZENBACH H | 1 | 0.033 |
| YANG C | 12 | 0.399 | SCHWARTZ S | 1 | 0.033 |
| WANG ZZ | 12 | 0.399 | SCHWARTZ MG | 1 | 0.033 |
| WANG YL | 12 | 0.399 | SCHWACKE R | 1 | 0.033 |
| WANG YC | 12 | 0.399 | SCHUSTER AS | 1 | 0.033 |
| WANG SH | 12 | 0.399 | SCHUMANN U | 1 | 0.033 |
| WANG S | 12 | 0.399 | SCHULZ WA | 1 | 0.033 |
| SONG Y | 12 | 0.399 | SCHULZ H | 1 | 0.033 |
| LU X | 12 | 0.399 | SCHULTZ N | 1 | 0.033 |
| LU J | 12 | 0.399 | SCHULTZ BM | 1 | 0.033 |
| LIAO Q | 12 | 0.399 | SCHULTE DM | 1 | 0.033 |
| LI N | 12 | 0.399 | SCHULER M | 1 | 0.033 |
| GAO X | 12 | 0.399 | SCHULER A | 1 | 0.033 |
| DING J | 12 | 0.399 | SCHRODTER S | 1 | 0.033 |
| CHENG L | 12 | 0.399 | SCHRODER JO | 1 | 0.033 |
| CHEN P | 12 | 0.399 | SCHREWE H | 1 | 0.033 |
| ZHOU WP | 11 | 0.366 | SCHRANZ ME | 1 | 0.033 |
| ZHOU J | 11 | 0.366 | SCHOTTA G | 1 | 0.033 |
| ZHAO J | 11 | 0.366 | SCHORDERET P | 1 | 0.033 |
| ZHANG XH | 11 | 0.366 | SCHONROCK N | 1 | 0.033 |
| ZHANG S | 11 | 0.366 | SCHONES DE | 1 | 0.033 |
| ZHANG M | 11 | 0.366 | SCHOLZ I | 1 | 0.033 |
| ZHANG JF | 11 | 0.366 | SCHOLES J | 1 | 0.033 |
| ZHANG C | 11 | 0.366 | SCHNEIDEROVA M | 1 | 0.033 |
| YIN DD | 11 | 0.366 | SCHNEIDER R | 1 | 0.033 |
| YANG H | 11 | 0.366 | SCHNEIDER M | 1 | 0.033 |
| WENG MZ | 11 | 0.366 | SCHNABLE PS | 1 | 0.033 |
| WANG ZX | 11 | 0.366 | SCHMITZ SU | 1 | 0.033 |
| WAHLESTEDT C | 11 | 0.366 | SCHMITZ A | 1 | 0.033 |
| QUAN ZW | 11 | 0.366 | SCHMITT N | 1 | 0.033 |
| QIU JJ | 11 | 0.366 | SCHMIDT LH | 1 | 0.033 |
| LV J | 11 | 0.366 | SCHMIDT K | 1 | 0.033 |
| LIU M | 11 | 0.366 | SCHMIDT F | 1 | 0.033 |
| LI HY | 11 | 0.366 | SCHMIDT CJ | 1 | 0.033 |
| LI GL | 11 | 0.366 | SCHLOSSER K | 1 | 0.033 |
| JOHNSON R | 11 | 0.366 | SCHLITTER AM | 1 | 0.033 |
| JIANG Q | 11 | 0.366 | SCHLAEGER TM | 1 | 0.033 |
| JIANG H | 11 | 0.366 | SCHITTNY JC | 1 | 0.033 |
| HUANG Y | 11 | 0.366 | SCHIRMACHER P | 1 | 0.033 |
| HU Y | 11 | 0.366 | SCHILHABEL M | 1 | 0.033 |
| HIROSE T | 11 | 0.366 | SCHILDKRAUT JM | 1 | 0.033 |
| GUTTMAN M | 11 | 0.366 | SCHERF K | 1 | 0.033 |
| FENG FY | 11 | 0.366 | SCHERF A | 1 | 0.033 |
| CHINNAIYAN AM | 11 | 0.366 | SCHELCH K | 1 | 0.033 |
| CHEN Z | 11 | 0.366 | SCHEIN A | 1 | 0.033 |
| CHEN YQ | 11 | 0.366 | SCHAUKOWITCH K | 1 | 0.033 |
| CHEN JJ | 11 | 0.366 | SCHATTGEN SA | 1 | 0.033 |
| ZHU Y | 10 | 0.332 | SCHAMBACH A | 1 | 0.033 |
| YANG X | 10 | 0.332 | SCHAFER A | 1 | 0.033 |
| XIAO Y | 10 | 0.332 | SCARUFFI P | 1 | 0.033 |
| XIAO BX | 10 | 0.332 | SCARPINI E | 1 | 0.033 |
| WANG YZ | 10 | 0.332 | SCARAVILLI M | 1 | 0.033 |
| WANG XY | 10 | 0.332 | SCANLON MJ | 1 | 0.033 |
| WANG XJ | 10 | 0.332 | SBONER A | 1 | 0.033 |
| WANG XH | 10 | 0.332 | SBLATTERO D | 1 | 0.033 |
| WANG R | 10 | 0.332 | SAYOLS S | 1 | 0.033 |
| WANG JL | 10 | 0.332 | SAYIN VI | 1 | 0.033 |
| THUM T | 10 | 0.332 | SAYED A | 1 | 0.033 |
| SHI Y | 10 | 0.332 | SAXENA A | 1 | 0.033 |
| SHEN J | 10 | 0.332 | SAWCER SJ | 1 | 0.033 |
| SHAO YF | 10 | 0.332 | SAWAKAMI T | 1 | 0.033 |
| QU K | 10 | 0.332 | SAWADA J | 1 | 0.033 |
| QIAN J | 10 | 0.332 | SAWADA G | 1 | 0.033 |
| PRENSNER JR | 10 | 0.332 | SAVONEN CL | 1 | 0.033 |
| MA X | 10 | 0.332 | SAVIC N | 1 | 0.033 |
| LIU SM | 10 | 0.332 | SAUS E | 1 | 0.033 |
| LI JF | 10 | 0.332 | SAUNUS JM | 1 | 0.033 |
| LI CY | 10 | 0.332 | SAUER T | 1 | 0.033 |
| HUARTE M | 10 | 0.332 | SAUER S | 1 | 0.033 |
| HUANG J | 10 | 0.332 | SATTARI A | 1 | 0.033 |
| HU J | 10 | 0.332 | SATTAR N | 1 | 0.033 |
| GAO W | 10 | 0.332 | SATOH Y | 1 | 0.033 |
| CHEN JH | 10 | 0.332 | SATOH K | 1 | 0.033 |
| CHEN HY | 10 | 0.332 | SATO I | 1 | 0.033 |
| ABDELMOHSEN K | 10 | 0.332 | SATO H | 1 | 0.033 |
| ZHU H | 9 | 0.299 | SATAKE H | 1 | 0.033 |
| ZHOU D | 9 | 0.299 | SASAKI YF | 1 | 0.033 |
| ZHAO Z | 9 | 0.299 | SASAKI Y | 1 | 0.033 |
| ZHAO W | 9 | 0.299 | SASAKI S | 1 | 0.033 |
| ZHAO H | 9 | 0.299 | SAS-CHEN A | 1 | 0.033 |
| ZHANG ZH | 9 | 0.299 | SARRE A | 1 | 0.033 |
| ZHANG Z | 9 | 0.299 | SARMINI M | 1 | 0.033 |
| ZHANG XD | 9 | 0.299 | SARMA K | 1 | 0.033 |
| ZHANG P | 9 | 0.299 | SARKARIA JN | 1 | 0.033 |
| ZHANG MD | 9 | 0.299 | SARKAR S | 1 | 0.033 |
| ZHANG JY | 9 | 0.299 | SARASWATHI MS | 1 | 0.033 |
| YUAN SX | 9 | 0.299 | SANZ L | 1 | 0.033 |
| YUAN JH | 9 | 0.299 | SANYAL A | 1 | 0.033 |
| YU X | 9 | 0.299 | SANTOYO-LOPEZ J | 1 | 0.033 |
| YANG P | 9 | 0.299 | SANTOS RHB | 1 | 0.033 |
| YANG GS | 9 | 0.299 | SANTOS F | 1 | 0.033 |
| XUE M | 9 | 0.299 | SANTORO M | 1 | 0.033 |
| WU Q | 9 | 0.299 | SANTIAGO-TURLA C | 1 | 0.033 |
| WU B | 9 | 0.299 | SANTELLA RM | 1 | 0.033 |
| WANG YM | 9 | 0.299 | SANSEVERINO W | 1 | 0.033 |
| WANG YF | 9 | 0.299 | SANLI Y | 1 | 0.033 |
| WANG TT | 9 | 0.299 | SANG YH | 1 | 0.033 |
| WANG JD | 9 | 0.299 | SANG Y | 1 | 0.033 |
| WANG CY | 9 | 0.299 | SANG HW | 1 | 0.033 |
| WANG B | 9 | 0.299 | SANDOVAL S | 1 | 0.033 |
| SUN H | 9 | 0.299 | SANDHYA P | 1 | 0.033 |
| SHI L | 9 | 0.299 | SANDHU P | 1 | 0.033 |
| REGEV A | 9 | 0.299 | SANDHU KS | 1 | 0.033 |
| QIN C | 9 | 0.299 | SANDER C | 1 | 0.033 |
| OROM UA | 9 | 0.299 | SANDELIN A | 1 | 0.033 |
| MERCER TR | 9 | 0.299 | SANCHO-MARTINEZ I | 1 | 0.033 |
| LUO HT | 9 | 0.299 | SANCHEZ-PEREZ Y | 1 | 0.033 |
| LIU YW | 9 | 0.299 | SANCHEZ-PARRA C | 1 | 0.033 |
| LIU X | 9 | 0.299 | SANCHEZ AM | 1 | 0.033 |
| LIU W | 9 | 0.299 | SAMUELS M | 1 | 0.033 |
| LI ZQ | 9 | 0.299 | SAMPOL A | 1 | 0.033 |
| LI YJ | 9 | 0.299 | SAMPATH P | 1 | 0.033 |
| LI XY | 9 | 0.299 | SAMIMI G | 1 | 0.033 |
| LI PF | 9 | 0.299 | SAMARTZIS D | 1 | 0.033 |
| LI GY | 9 | 0.299 | SAMAEI NM | 1 | 0.033 |
| LEE JT | 9 | 0.299 | SAM N | 1 | 0.033 |
| KIM J | 9 | 0.299 | SALVEMINI M | 1 | 0.033 |
| KANDURI C | 9 | 0.299 | SALONIA A | 1 | 0.033 |
| IYER MK | 9 | 0.299 | SALOMONIS N | 1 | 0.033 |
| HUANG YQ | 9 | 0.299 | SALMERON CC | 1 | 0.033 |
| CHEN XW | 9 | 0.299 | SALLAM M | 1 | 0.033 |
| CHEN XM | 9 | 0.299 | SALINAS RD | 1 | 0.033 |
| CHEN WM | 9 | 0.299 | SALBERT G | 1 | 0.033 |
| CHEN CY | 9 | 0.299 | SALAMEH A | 1 | 0.033 |
| CHEN C | 9 | 0.299 | SALAH-UDDIN S | 1 | 0.033 |
| CAO XF | 9 | 0.299 | SALA N | 1 | 0.033 |
| ZHUANG CL | 8 | 0.266 | SALA I | 1 | 0.033 |
| ZHU L | 8 | 0.266 | SAKURAI K | 1 | 0.033 |
| ZHENG J | 8 | 0.266 | SAKASHITA A | 1 | 0.033 |
| ZHAO XY | 8 | 0.266 | SAKAMOTO Y | 1 | 0.033 |
| ZHAO X | 8 | 0.266 | SAKAMOTO N | 1 | 0.033 |
| ZHANG XY | 8 | 0.266 | SAKAI S | 1 | 0.033 |
| ZHANG WJ | 8 | 0.266 | SAITO S | 1 | 0.033 |
| ZHANG JW | 8 | 0.266 | SAINI S | 1 | 0.033 |
| ZHANG CL | 8 | 0.266 | SAIGUSA S | 1 | 0.033 |
| ZHANG B | 8 | 0.266 | SAIEVA L | 1 | 0.033 |
| ZENG ZY | 8 | 0.266 | SAIDIJAM M | 1 | 0.033 |
| YU J | 8 | 0.266 | SAIAKHOVA A | 1 | 0.033 |
| YU H | 8 | 0.266 | SAHU D | 1 | 0.033 |
| YOON JH | 8 | 0.266 | SAHINALP CS | 1 | 0.033 |
| YANG HX | 8 | 0.266 | SAHIN D | 1 | 0.033 |
| XUE YX | 8 | 0.266 | SAHA SS | 1 | 0.033 |
| XU YT | 8 | 0.266 | SAHA S | 1 | 0.033 |
| WU XL | 8 | 0.266 | SAHA A | 1 | 0.033 |
| WU WJ | 8 | 0.266 | SAGAR V | 1 | 0.033 |
| WU W | 8 | 0.266 | SAFI A | 1 | 0.033 |
| WU H | 8 | 0.266 | SAFARALIZADEH R | 1 | 0.033 |
| WANG YX | 8 | 0.266 | SAEYS Y | 1 | 0.033 |
| WANG YJ | 8 | 0.266 | SAEIJ JPJ | 1 | 0.033 |
| WANG XQ | 8 | 0.266 | SADREYEV RI | 1 | 0.033 |
| WANG SW | 8 | 0.266 | SADREYEV R | 1 | 0.033 |
| WANG LL | 8 | 0.266 | SADO T | 1 | 0.033 |
| WANG KM | 8 | 0.266 | SADHUKHAN T | 1 | 0.033 |
| WANG JY | 8 | 0.266 | SADAOUI NC | 1 | 0.033 |
| WANG JX | 8 | 0.266 | SACO A | 1 | 0.033 |
| WANG FB | 8 | 0.266 | SACHS F | 1 | 0.033 |
| WANG CX | 8 | 0.266 | SACHLEBEN JR | 1 | 0.033 |
| ULITSKY I | 8 | 0.266 | SABRIPOUR M | 1 | 0.033 |
| SUN WL | 8 | 0.266 | SABONGUI S | 1 | 0.033 |
| SHIEKHATTAR R | 8 | 0.266 | SABLINA AA | 1 | 0.033 |
| SHI HB | 8 | 0.266 | SABERS JN | 1 | 0.033 |
| QIU MT | 8 | 0.266 | SABBAGH A | 1 | 0.033 |
| QI P | 8 | 0.266 | SAAYMAN S | 1 | 0.033 |
| PERERA RJ | 8 | 0.266 | SAAR K | 1 | 0.033 |
| PENG W | 8 | 0.266 | SAAD AS | 1 | 0.033 |
| NIE FQ | 8 | 0.266 | RYU D | 1 | 0.033 |
| MO YY | 8 | 0.266 | RUTENBERG-SCHOENBERG M | 1 | 0.033 |
| LV QL | 8 | 0.266 | RUSSO R | 1 | 0.033 |
| LU KH | 8 | 0.266 | RUSSELL MR | 1 | 0.033 |
| LODISH HF | 8 | 0.266 | RUSAKIEWICZ S | 1 | 0.033 |
| LIU XL | 8 | 0.266 | RUPAIMOOLE R | 1 | 0.033 |
| LIU JY | 8 | 0.266 | RUIZ-ORERA J | 1 | 0.033 |
| LIU JF | 8 | 0.266 | RUIZ C | 1 | 0.033 |
| LIU CY | 8 | 0.266 | RUIJTER AEM | 1 | 0.033 |
| LIN L | 8 | 0.266 | RUI C | 1 | 0.033 |
| LI YH | 8 | 0.266 | RUEDA A | 1 | 0.033 |
| LI R | 8 | 0.266 | RUDERFER D | 1 | 0.033 |
| LI JH | 8 | 0.266 | RUBIO C | 1 | 0.033 |
| LI B | 8 | 0.266 | RUBINSTEIN A | 1 | 0.033 |
| LANDER ES | 8 | 0.266 | RUAN YT | 1 | 0.033 |
| KHALIL AM | 8 | 0.266 | RUAN YJ | 1 | 0.033 |
| JIA H | 8 | 0.266 | RUAN Y | 1 | 0.033 |
| JALALI S | 8 | 0.266 | RUAN XA | 1 | 0.033 |
| HUANG WR | 8 | 0.266 | RUAN WH | 1 | 0.033 |
| HUANG MD | 8 | 0.266 | RUAN WD | 1 | 0.033 |
| HUANG C | 8 | 0.266 | RUAN MH | 1 | 0.033 |
| HUA KQ | 8 | 0.266 | RUAN L | 1 | 0.033 |
| HU ZY | 8 | 0.266 | RUAN KH | 1 | 0.033 |
| HU YW | 8 | 0.266 | RUAN HL | 1 | 0.033 |
| HE ZY | 8 | 0.266 | RUAN HJ | 1 | 0.033 |
| HE AB | 8 | 0.266 | RUAN B | 1 | 0.033 |
| GUO J | 8 | 0.266 | RU K | 1 | 0.033 |
| GUIGO R | 8 | 0.266 | RU G | 1 | 0.033 |
| GU Y | 8 | 0.266 | ROY-CHOWDHURY J | 1 | 0.033 |
| GARBER M | 8 | 0.266 | ROUX B | 1 | 0.033 |
| DU X | 8 | 0.266 | ROUPRET M | 1 | 0.033 |
| DIEDERICHS S | 8 | 0.266 | ROTTER B | 1 | 0.033 |
| DAI Y | 8 | 0.266 | ROTHSCHILD G | 1 | 0.033 |
| CUI QH | 8 | 0.266 | ROTHI MH | 1 | 0.033 |
| CHEN ZC | 8 | 0.266 | ROTHENBURG J | 1 | 0.033 |
| CHEN YX | 8 | 0.266 | ROTH-KLEINER M | 1 | 0.033 |
| CHEN XN | 8 | 0.266 | ROTH WK | 1 | 0.033 |
| CAI ZM | 8 | 0.266 | ROTH A | 1 | 0.033 |
| BU DC | 8 | 0.266 | ROTBLAT B | 1 | 0.033 |
| BOZZONI I | 8 | 0.266 | ROSZIK J | 1 | 0.033 |
| ZOU LF | 7 | 0.233 | ROSSI RL | 1 | 0.033 |
| ZHU J | 7 | 0.233 | ROSSI JJ | 1 | 0.033 |
| ZHOU LY | 7 | 0.233 | ROSSETTO CC | 1 | 0.033 |
| ZHOU LQ | 7 | 0.233 | ROSSETTI G | 1 | 0.033 |
| ZHOU JY | 7 | 0.233 | ROSS JP | 1 | 0.033 |
| ZHOU HH | 7 | 0.233 | ROSS E | 1 | 0.033 |
| ZHENG L | 7 | 0.233 | ROSS A | 1 | 0.033 |
| ZHAO M | 7 | 0.233 | ROSKA B | 1 | 0.033 |
| ZHAO L | 7 | 0.233 | ROSIN MP | 1 | 0.033 |
| ZHAO HT | 7 | 0.233 | ROSIKIEWICZ W | 1 | 0.033 |
| ZHAO HQ | 7 | 0.233 | ROSIC S | 1 | 0.033 |
| ZHAO GP | 7 | 0.233 | ROSENWALD A | 1 | 0.033 |
| ZHAO B | 7 | 0.233 | ROSENSTIEL P | 1 | 0.033 |
| ZHANG YS | 7 | 0.233 | ROSENFELD JA | 1 | 0.033 |
| ZHANG YQ | 7 | 0.233 | ROSENBLUM MG | 1 | 0.033 |
| ZHANG YP | 7 | 0.233 | ROSENBERGER A | 1 | 0.033 |
| ZHANG YJ | 7 | 0.233 | ROSENBERG A | 1 | 0.033 |
| ZHANG XW | 7 | 0.233 | ROSEN JM | 1 | 0.033 |
| ZHANG WH | 7 | 0.233 | ROSEN GD | 1 | 0.033 |
| ZHANG R | 7 | 0.233 | ROSE D | 1 | 0.033 |
| ZHANG LF | 7 | 0.233 | ROSA A | 1 | 0.033 |
| ZHANG JP | 7 | 0.233 | ROOT DE | 1 | 0.033 |
| ZHANG HL | 7 | 0.233 | ROOJ AK | 1 | 0.033 |
| YE LC | 7 | 0.233 | ROOIJERS K | 1 | 0.033 |
| YANG S | 7 | 0.233 | RONG Y | 1 | 0.033 |
| YANG Q | 7 | 0.233 | RONG MH | 1 | 0.033 |
| YANG JH | 7 | 0.233 | RONG LF | 1 | 0.033 |
| YANG JC | 7 | 0.233 | RONG LC | 1 | 0.033 |
| XU YJ | 7 | 0.233 | RONG JJ | 1 | 0.033 |
| XU Q | 7 | 0.233 | RONG CH | 1 | 0.033 |
| XU MD | 7 | 0.233 | RONDOU P | 1 | 0.033 |
| XU M | 7 | 0.233 | ROMERO-BARRIOS N | 1 | 0.033 |
| XU CL | 7 | 0.233 | ROMERO R | 1 | 0.033 |
| XIA Y | 7 | 0.233 | ROMANELLI A | 1 | 0.033 |
| XIA T | 7 | 0.233 | ROLAND BC | 1 | 0.033 |
| WU Z | 7 | 0.233 | ROJAS J | 1 | 0.033 |
| WU XC | 7 | 0.233 | ROHDE M | 1 | 0.033 |
| WU QQ | 7 | 0.233 | ROHDE C | 1 | 0.033 |
| WU F | 7 | 0.233 | ROGOZIN IB | 1 | 0.033 |
| WANG YQ | 7 | 0.233 | ROGLER LE | 1 | 0.033 |
| WANG JP | 7 | 0.233 | ROGLER CE | 1 | 0.033 |
| WANG JJ | 7 | 0.233 | ROGIERS A | 1 | 0.033 |
| VERJOVSKI-ALMEIDA S | 7 | 0.233 | ROGERS JM | 1 | 0.033 |
| TIAN Y | 7 | 0.233 | ROEWLEY MJ | 1 | 0.033 |
| SUN YH | 7 | 0.233 | ROESZLER KN | 1 | 0.033 |
| SUN W | 7 | 0.233 | ROEBUCK P | 1 | 0.033 |
| SUN T | 7 | 0.233 | RODRIGUEZ-PERALTO JL | 1 | 0.033 |
| SUN Q | 7 | 0.233 | RODRIGUEZ BAT | 1 | 0.033 |
| SUN B | 7 | 0.233 | RODOSTHENOUS RS | 1 | 0.033 |
| SIVASUBBU S | 7 | 0.233 | ROCKETT KA | 1 | 0.033 |
| SHI XF | 7 | 0.233 | ROCHE O | 1 | 0.033 |
| SHEN C | 7 | 0.233 | ROCHA D | 1 | 0.033 |
| NING SW | 7 | 0.233 | ROBSON P | 1 | 0.033 |
| MESTDAGH P | 7 | 0.233 | ROBINSON J | 1 | 0.033 |
| MA T | 7 | 0.233 | ROBINSON D | 1 | 0.033 |
| MA L | 7 | 0.233 | ROBINSON BG | 1 | 0.033 |
| LUO JJ | 7 | 0.233 | ROBERTSON GA | 1 | 0.033 |
| LUO F | 7 | 0.233 | ROBERTS RM | 1 | 0.033 |
| LU L | 7 | 0.233 | ROBERTS KA | 1 | 0.033 |
| LIU Z | 7 | 0.233 | ROBERTS AL | 1 | 0.033 |
| LIU QZ | 7 | 0.233 | ROA L | 1 | 0.033 |
| LIU FT | 7 | 0.233 | RIVERA I | 1 | 0.033 |
| LING H | 7 | 0.233 | RIVAS A | 1 | 0.033 |
| LIANG SD | 7 | 0.233 | RIVA P | 1 | 0.033 |
| LI ZG | 7 | 0.233 | RITZ J | 1 | 0.033 |
| LI YQ | 7 | 0.233 | RISUENO RM | 1 | 0.033 |
| LI MX | 7 | 0.233 | RISK B | 1 | 0.033 |
| LI JY | 7 | 0.233 | RISCH HA | 1 | 0.033 |
| LI A | 7 | 0.233 | RISCH H | 1 | 0.033 |
| HUANG QH | 7 | 0.233 | RIPOLI C | 1 | 0.033 |
| HONG J | 7 | 0.233 | RIPEN AM | 1 | 0.033 |
| HE XZ | 7 | 0.233 | RIOS-BARRERA LD | 1 | 0.033 |
| GOFF LA | 7 | 0.233 | RIOS EJ | 1 | 0.033 |
| GENG PL | 7 | 0.233 | RINGEL MD | 1 | 0.033 |
| FLYNN RA | 7 | 0.233 | RIMESSI P | 1 | 0.033 |
| FENG L | 7 | 0.233 | RIJZEWIJK LJ | 1 | 0.033 |
| FENG JF | 7 | 0.233 | RIIS M | 1 | 0.033 |
| DONG SY | 7 | 0.233 | RIIHILA P | 1 | 0.033 |
| DING Y | 7 | 0.233 | RIGO FW | 1 | 0.033 |
| DING JX | 7 | 0.233 | RIESGO-ESCOVAR R | 1 | 0.033 |
| CROCE CM | 7 | 0.233 | RIEHLE K | 1 | 0.033 |
| CHU HY | 7 | 0.233 | RIDOLFI E | 1 | 0.033 |
| CHEN ZH | 7 | 0.233 | RIDDICK G | 1 | 0.033 |
| CHEN S | 7 | 0.233 | RICKLEFS F | 1 | 0.033 |
| CHEN MW | 7 | 0.233 | RICHTER J | 1 | 0.033 |
| CHEN F | 7 | 0.233 | RICHHEIMER S | 1 | 0.033 |
| CAO XH | 7 | 0.233 | RICHARDS A | 1 | 0.033 |
| CAI Y | 7 | 0.233 | RICHARD JLC | 1 | 0.033 |
| ZHU PQ | 6 | 0.199 | RICE AP | 1 | 0.033 |
| ZHU M | 6 | 0.199 | RICCIUTI B | 1 | 0.033 |
| ZHOU C | 6 | 0.199 | RICCI L | 1 | 0.033 |
| ZHOU B | 6 | 0.199 | RIBERA JM | 1 | 0.033 |
| ZHENG XF | 6 | 0.199 | RIABOWOL K | 1 | 0.033 |
| ZHENG SS | 6 | 0.199 | REYMONDIN D | 1 | 0.033 |
| ZHAO YL | 6 | 0.199 | REYMOND A | 1 | 0.033 |
| ZHAO XH | 6 | 0.199 | REYES-VALDES MH | 1 | 0.033 |
| ZHAO GG | 6 | 0.199 | REVERTER F | 1 | 0.033 |
| ZHAO D | 6 | 0.199 | RESNICK R | 1 | 0.033 |
| ZHAO C | 6 | 0.199 | REPULLES J | 1 | 0.033 |
| ZHANG ZJ | 6 | 0.199 | RENGANATHAN A | 1 | 0.033 |
| ZHANG ZD | 6 | 0.199 | REN ZR | 1 | 0.033 |
| ZHANG YC | 6 | 0.199 | REN ZQ | 1 | 0.033 |
| ZHANG WL | 6 | 0.199 | REN YP | 1 | 0.033 |
| ZHANG T | 6 | 0.199 | REN YK | 1 | 0.033 |
| ZHANG JX | 6 | 0.199 | REN XY | 1 | 0.033 |
| ZHANG HH | 6 | 0.199 | REN XX | 1 | 0.033 |
| ZHANG HB | 6 | 0.199 | REN XF | 1 | 0.033 |
| ZHANG G | 6 | 0.199 | REN XC | 1 | 0.033 |
| ZHANG DD | 6 | 0.199 | REN X | 1 | 0.033 |
| YUAN QP | 6 | 0.199 | REN WG | 1 | 0.033 |
| YU Y | 6 | 0.199 | REN W | 1 | 0.033 |
| YOU LH | 6 | 0.199 | REN TY | 1 | 0.033 |
| YAO Y | 6 | 0.199 | REN SJ | 1 | 0.033 |
| YANG YM | 6 | 0.199 | REN PP | 1 | 0.033 |
| YANG XY | 6 | 0.199 | REN N | 1 | 0.033 |
| YANG XL | 6 | 0.199 | REN MM | 1 | 0.033 |
| YANG W | 6 | 0.199 | REN M | 1 | 0.033 |
| YANG R | 6 | 0.199 | REN LL | 1 | 0.033 |
| YANG JS | 6 | 0.199 | REN L | 1 | 0.033 |
| YANG JM | 6 | 0.199 | REN KW | 1 | 0.033 |
| YANG JJ | 6 | 0.199 | REN JJ | 1 | 0.033 |
| XU W | 6 | 0.199 | REN HZ | 1 | 0.033 |
| XU D | 6 | 0.199 | REN HT | 1 | 0.033 |
| XU CS | 6 | 0.199 | REN H | 1 | 0.033 |
| XIONG Y | 6 | 0.199 | REN GS | 1 | 0.033 |
| XIONG W | 6 | 0.199 | REN FH | 1 | 0.033 |
| XIE X | 6 | 0.199 | REN DB | 1 | 0.033 |
| XIE M | 6 | 0.199 | REN CH | 1 | 0.033 |
| XIE HY | 6 | 0.199 | REN C | 1 | 0.033 |
| XIE H | 6 | 0.199 | REMOTTI H | 1 | 0.033 |
| XIAO H | 6 | 0.199 | REMMELE CW | 1 | 0.033 |
| WILLIAMS GT | 6 | 0.199 | REMKE J | 1 | 0.033 |
| WANG ZY | 6 | 0.199 | RELAN V | 1 | 0.033 |
| WANG ZM | 6 | 0.199 | REIS A | 1 | 0.033 |
| WANG ML | 6 | 0.199 | REINMAA E | 1 | 0.033 |
| WANG LJ | 6 | 0.199 | REINHARDT D | 1 | 0.033 |
| WANG HY | 6 | 0.199 | REINERI S | 1 | 0.033 |
| WANG HJ | 6 | 0.199 | REID BM | 1 | 0.033 |
| WANG D | 6 | 0.199 | REGEL I | 1 | 0.033 |
| WANG C | 6 | 0.199 | REESE AL | 1 | 0.033 |
| VANDESOMPELE J | 6 | 0.199 | REEMTSEN B | 1 | 0.033 |
| TONG YS | 6 | 0.199 | REED AEM | 1 | 0.033 |
| TANG XY | 6 | 0.199 | REDDY P | 1 | 0.033 |
| TANG X | 6 | 0.199 | REDDY MA | 1 | 0.033 |
| TANG W | 6 | 0.199 | RECZKO M | 1 | 0.033 |
| TAN C | 6 | 0.199 | RECILLAS-TARGA F | 1 | 0.033 |
| STANTON LW | 6 | 0.199 | RECCHIA AG | 1 | 0.033 |
| SPECTOR DL | 6 | 0.199 | REBOURS E | 1 | 0.033 |
| SONG W | 6 | 0.199 | REBBECK T | 1 | 0.033 |
| SONG HJ | 6 | 0.199 | RE A | 1 | 0.033 |
| SHI WH | 6 | 0.199 | RAZ T | 1 | 0.033 |
| SHEN XH | 6 | 0.199 | RAYMOND CK | 1 | 0.033 |
| SHEN B | 6 | 0.199 | RAY T | 1 | 0.033 |
| SANBONMATSU KY | 6 | 0.199 | RAY PS | 1 | 0.033 |
| REN SC | 6 | 0.199 | RAY MK | 1 | 0.033 |
| PRASANTH KV | 6 | 0.199 | RAVID O | 1 | 0.033 |
| PICKARD MR | 6 | 0.199 | RAVICH W | 1 | 0.033 |
| PEDRAZZINI T | 6 | 0.199 | RAVAL A | 1 | 0.033 |
| PATEL T | 6 | 0.199 | RAUTENBACH RM | 1 | 0.033 |
| PAN H | 6 | 0.199 | RAUTANEN A | 1 | 0.033 |
| MA R | 6 | 0.199 | RAULEFS S | 1 | 0.033 |
| LUO HL | 6 | 0.199 | RAUCK CE | 1 | 0.033 |
| LU YY | 6 | 0.199 | RAUCCI A | 1 | 0.033 |
| LU YJ | 6 | 0.199 | RATTI A | 1 | 0.033 |
| LU LG | 6 | 0.199 | RATNAM M | 1 | 0.033 |
| LIU ZY | 6 | 0.199 | RATHI KS | 1 | 0.033 |
| LIU ZJ | 6 | 0.199 | RATH MF | 1 | 0.033 |
| LIU ZH | 6 | 0.199 | RATH B | 1 | 0.033 |
| LIU YL | 6 | 0.199 | RASTINEJAD F | 1 | 0.033 |
| LIU YJ | 6 | 0.199 | RASSCHAERT D | 1 | 0.033 |
| LIU XX | 6 | 0.199 | RAO SL | 1 | 0.033 |
| LIU LH | 6 | 0.199 | RAO JN | 1 | 0.033 |
| LIU K | 6 | 0.199 | RAO J | 1 | 0.033 |
| LIU JB | 6 | 0.199 | RAO ALN | 1 | 0.033 |
| LIU G | 6 | 0.199 | RANU H | 1 | 0.033 |
| LIU CN | 6 | 0.199 | RANKIN CR | 1 | 0.033 |
| LIN Y | 6 | 0.199 | RANI N | 1 | 0.033 |
| LIN JH | 6 | 0.199 | RANGEL Z | 1 | 0.033 |
| LI ZY | 6 | 0.199 | RANA ZA | 1 | 0.033 |
| LI YX | 6 | 0.199 | RAN ZH | 1 | 0.033 |
| LI YS | 6 | 0.199 | RAN ML | 1 | 0.033 |
| LI XR | 6 | 0.199 | RAN LK | 1 | 0.033 |
| LI TY | 6 | 0.199 | RAMSAY M | 1 | 0.033 |
| LI RH | 6 | 0.199 | RAMOS A | 1 | 0.033 |
| LI LJ | 6 | 0.199 | RAMNARINE VR | 1 | 0.033 |
| LEUNG GKK | 6 | 0.199 | RAMIREZ MS | 1 | 0.033 |
| KRETZ M | 6 | 0.199 | RAMIREZ J | 1 | 0.033 |
| KOTAKE Y | 6 | 0.199 | RAMCHANDRAN R | 1 | 0.033 |
| JIANG YG | 6 | 0.199 | RAMASWAMI G | 1 | 0.033 |
| JIANG Y | 6 | 0.199 | RAMAO A | 1 | 0.033 |
| JIANG L | 6 | 0.199 | RAMANUJAN K | 1 | 0.033 |
| HU WQ | 6 | 0.199 | RAMAN P | 1 | 0.033 |
| HE Y | 6 | 0.199 | RAMACHANDRAN R | 1 | 0.033 |
| HAN Y | 6 | 0.199 | RAKHIT CP | 1 | 0.033 |
| HAN X | 6 | 0.199 | RAJPATHAK SN | 1 | 0.033 |
| GUO YL | 6 | 0.199 | RAJARAJACHOLAN U | 1 | 0.033 |
| GUO XQ | 6 | 0.199 | RAJAPAKSHE K | 1 | 0.033 |
| GUO MZ | 6 | 0.199 | RAJAKANNU P | 1 | 0.033 |
| GUO L | 6 | 0.199 | RAIMONDI I | 1 | 0.033 |
| GUO H | 6 | 0.199 | RAI A | 1 | 0.033 |
| GHOSH S | 6 | 0.199 | RAHMAN MH | 1 | 0.033 |
| FU ZY | 6 | 0.199 | RAHIMZADEH J | 1 | 0.033 |
| FREIER SM | 6 | 0.199 | RAHIMY E | 1 | 0.033 |
| FORTES P | 6 | 0.199 | RAHIM A | 1 | 0.033 |
| FITZGERALD KA | 6 | 0.199 | RAGHAVA GPS | 1 | 0.033 |
| FENG Y | 6 | 0.199 | RAGAN C | 1 | 0.033 |
| FANG JY | 6 | 0.199 | RAFNAR T | 1 | 0.033 |
| FAN Y | 6 | 0.199 | RAE JM | 1 | 0.033 |
| FAN XQ | 6 | 0.199 | RADULOVIC V | 1 | 0.033 |
| FAGHIHI MA | 6 | 0.199 | RADOVICH M | 1 | 0.033 |
| DANG YW | 6 | 0.199 | RADOMSKA KJ | 1 | 0.033 |
| CHEN ZL | 6 | 0.199 | RADOMSKA HS | 1 | 0.033 |
| CHEN SH | 6 | 0.199 | RADINE C | 1 | 0.033 |
| CHEN Q | 6 | 0.199 | RADER JS | 1 | 0.033 |
| CHEN LB | 6 | 0.199 | RACKHAM O | 1 | 0.033 |
| CARNINCI P | 6 | 0.199 | RACCOSTA S | 1 | 0.033 |
| BAI J | 6 | 0.199 | RABU C | 1 | 0.033 |
| ALVAREZ-DOMINGUEZ JR | 6 | 0.199 | RABANI M | 1 | 0.033 |
| ZOU YF | 5 | 0.166 | RABAIA N | 1 | 0.033 |
| ZHU YY | 5 | 0.166 | RABADAN R | 1 | 0.033 |
| ZHU QH | 5 | 0.166 | QUO QH | 1 | 0.033 |
| ZHU LJ | 5 | 0.166 | QUN-WANG X | 1 | 0.033 |
| ZHU JJ | 5 | 0.166 | QUISPE J | 1 | 0.033 |
| ZHOU XF | 5 | 0.166 | QUINODOZ S | 1 | 0.033 |
| ZHOU P | 5 | 0.166 | QUIGNON P | 1 | 0.033 |
| ZHONG N | 5 | 0.166 | QUERTERMOUS T | 1 | 0.033 |
| ZHI QM | 5 | 0.166 | QUENET D | 1 | 0.033 |
| ZHI H | 5 | 0.166 | QUEK XC | 1 | 0.033 |
| ZHENG Y | 5 | 0.166 | QUE HX | 1 | 0.033 |
| ZHENG JJ | 5 | 0.166 | QUE GB | 1 | 0.033 |
| ZHAO XL | 5 | 0.166 | QUE B | 1 | 0.033 |
| ZHAO GQ | 5 | 0.166 | QUAN ZF | 1 | 0.033 |
| ZHANG YZ | 5 | 0.166 | QUAN HY | 1 | 0.033 |
| ZHANG XL | 5 | 0.166 | QUAN C | 1 | 0.033 |
| ZHANG JZ | 5 | 0.166 | QU ZQ | 1 | 0.033 |
| ZHANG GX | 5 | 0.166 | QU YH | 1 | 0.033 |
| ZHANG CP | 5 | 0.166 | QU XY | 1 | 0.033 |
| ZHAN YH | 5 | 0.166 | QU XH | 1 | 0.033 |
| YU Q | 5 | 0.166 | QU XF | 1 | 0.033 |
| YU FJ | 5 | 0.166 | QU X | 1 | 0.033 |
| YIN QF | 5 | 0.166 | QU SH | 1 | 0.033 |
| YIN LH | 5 | 0.166 | QU SB | 1 | 0.033 |
| YE LH | 5 | 0.166 | QU LW | 1 | 0.033 |
| YE J | 5 | 0.166 | QU JJ | 1 | 0.033 |
| YAO YW | 5 | 0.166 | QU HF | 1 | 0.033 |
| YAO YL | 5 | 0.166 | QU H | 1 | 0.033 |
| YANG YW | 5 | 0.166 | QU B | 1 | 0.033 |
| YANG YB | 5 | 0.166 | QIU ZL | 1 | 0.033 |
| YANG T | 5 | 0.166 | QIU YB | 1 | 0.033 |
| YANG N | 5 | 0.166 | QIU XT | 1 | 0.033 |
| YAN J | 5 | 0.166 | QIU XM | 1 | 0.033 |
| YAN GY | 5 | 0.166 | QIU WS | 1 | 0.033 |
| XU ZY | 5 | 0.166 | QIU MZ | 1 | 0.033 |
| XU ZP | 5 | 0.166 | QIU MY | 1 | 0.033 |
| XU XM | 5 | 0.166 | QIU LY | 1 | 0.033 |
| XU S | 5 | 0.166 | QIU LX | 1 | 0.033 |
| XU JM | 5 | 0.166 | QIU L | 1 | 0.033 |
| XU C | 5 | 0.166 | QIU KF | 1 | 0.033 |
| XU B | 5 | 0.166 | QIU J | 1 | 0.033 |
| XIE CY | 5 | 0.166 | QIU HF | 1 | 0.033 |
| XIAO L | 5 | 0.166 | QIU GZ | 1 | 0.033 |
| XIANG JF | 5 | 0.166 | QIU GQ | 1 | 0.033 |
| WU YH | 5 | 0.166 | QIU GP | 1 | 0.033 |
| WU L | 5 | 0.166 | QIU FJ | 1 | 0.033 |
| WU K | 5 | 0.166 | QIU CX | 1 | 0.033 |
| WU JJ | 5 | 0.166 | QIU B | 1 | 0.033 |
| WU HW | 5 | 0.166 | QINGZHANGDU | 1 | 0.033 |
| WU HJ | 5 | 0.166 | QING L | 1 | 0.033 |
| WU CY | 5 | 0.166 | QING C | 1 | 0.033 |
| WIERZBICKI AT | 5 | 0.166 | QIN ZY | 1 | 0.033 |
| WEN SY | 5 | 0.166 | QIN YW | 1 | 0.033 |
| WEI M | 5 | 0.166 | QIN YL | 1 | 0.033 |
| WEBSTER DE | 5 | 0.166 | QIN XB | 1 | 0.033 |
| WANG ZS | 5 | 0.166 | QIN WS | 1 | 0.033 |
| WANG ZJ | 5 | 0.166 | QIN W | 1 | 0.033 |
| WANG YW | 5 | 0.166 | QIN T | 1 | 0.033 |
| WANG YS | 5 | 0.166 | QIN LJ | 1 | 0.033 |
| WANG SB | 5 | 0.166 | QIN HL | 1 | 0.033 |
| WANG OC | 5 | 0.166 | QIN H | 1 | 0.033 |
| WANG MH | 5 | 0.166 | QIN G | 1 | 0.033 |
| WANG LH | 5 | 0.166 | QIN D | 1 | 0.033 |
| WANG KC | 5 | 0.166 | QIN CX | 1 | 0.033 |
| WANG JS | 5 | 0.166 | QIN BM | 1 | 0.033 |
| WANG HT | 5 | 0.166 | QIE FZ | 1 | 0.033 |
| WANG HQ | 5 | 0.166 | QIAO YQ | 1 | 0.033 |
| WANG GJ | 5 | 0.166 | QIAO YM | 1 | 0.033 |
| VOLDERS PJ | 5 | 0.166 | QIAO Y | 1 | 0.033 |
| TIAN J | 5 | 0.166 | QIAO XM | 1 | 0.033 |
| TANG L | 5 | 0.166 | QIAO SS | 1 | 0.033 |
| TANG B | 5 | 0.166 | QIAO Q | 1 | 0.033 |
| SUN YN | 5 | 0.166 | QIAO M | 1 | 0.033 |
| SUN YM | 5 | 0.166 | QIAO JL | 1 | 0.033 |
| SUN XY | 5 | 0.166 | QIAO HP | 1 | 0.033 |
| SONG GX | 5 | 0.166 | QIAO HF | 1 | 0.033 |
| SHI YG | 5 | 0.166 | QIAO GB | 1 | 0.033 |
| SHI XR | 5 | 0.166 | QIAO C | 1 | 0.033 |
| SHI J | 5 | 0.166 | QIANG FL | 1 | 0.033 |
| SHEN L | 5 | 0.166 | QIAN ZD | 1 | 0.033 |
| SENGUPTA S | 5 | 0.166 | QIAN YT | 1 | 0.033 |
| SEGURA V | 5 | 0.166 | QIAN YL | 1 | 0.033 |
| SCHROEN B | 5 | 0.166 | QIAN YJ | 1 | 0.033 |
| SAHU A | 5 | 0.166 | QIAN YB | 1 | 0.033 |
| RAJ A | 5 | 0.166 | QIAN Y | 1 | 0.033 |
| QU LH | 5 | 0.166 | QIAN XJ | 1 | 0.033 |
| QIU C | 5 | 0.166 | QIAN X | 1 | 0.033 |
| QIAN LM | 5 | 0.166 | QIAN WY | 1 | 0.033 |
| PU YP | 5 | 0.166 | QIAN WL | 1 | 0.033 |
| PICHLER M | 5 | 0.166 | QIAN LY | 1 | 0.033 |
| PENG JJ | 5 | 0.166 | QIAN HX | 1 | 0.033 |
| PASTORI C | 5 | 0.166 | QIAN HH | 1 | 0.033 |
| PANG WJ | 5 | 0.166 | QIAN H | 1 | 0.033 |
| PAN Y | 5 | 0.166 | QIAN F | 1 | 0.033 |
| PAN J | 5 | 0.166 | QIAN CY | 1 | 0.033 |
| PAN F | 5 | 0.166 | QIAN CW | 1 | 0.033 |
| OUNZAIN S | 5 | 0.166 | QIAN BY | 1 | 0.033 |
| OLIVER PL | 5 | 0.166 | QIAN AR | 1 | 0.033 |
| NOVIKOVA IV | 5 | 0.166 | QIAN AM | 1 | 0.033 |
| NG SY | 5 | 0.166 | QI ZT | 1 | 0.033 |
| MARQUES AC | 5 | 0.166 | QI ZQ | 1 | 0.033 |
| MARINE JC | 5 | 0.166 | QI YJ | 1 | 0.033 |
| MALIK R | 5 | 0.166 | QI XX | 1 | 0.033 |
| MAGISTRI M | 5 | 0.166 | QI XL | 1 | 0.033 |
| MA MZ | 5 | 0.166 | QI XF | 1 | 0.033 |
| MA GX | 5 | 0.166 | QI KM | 1 | 0.033 |
| LV X | 5 | 0.166 | QI HZ | 1 | 0.033 |
| LV MM | 5 | 0.166 | QI HP | 1 | 0.033 |
| LUO M | 5 | 0.166 | QI HL | 1 | 0.033 |
| LU ZQ | 5 | 0.166 | QI F | 1 | 0.033 |
| LU ZJ | 5 | 0.166 | QI DT | 1 | 0.033 |
| LU XL | 5 | 0.166 | QI DF | 1 | 0.033 |
| LU W | 5 | 0.166 | QI CJ | 1 | 0.033 |
| LU S | 5 | 0.166 | QI C | 1 | 0.033 |
| LU Q | 5 | 0.166 | QI BM | 1 | 0.033 |
| LIU ZL | 5 | 0.166 | PUVVULA PK | 1 | 0.033 |
| LIU YM | 5 | 0.166 | PUTTA S | 1 | 0.033 |
| LIU S | 5 | 0.166 | PUTHANVEETIL P | 1 | 0.033 |
| LIU R | 5 | 0.166 | PURUSHOTHAMAN S | 1 | 0.033 |
| LIU HL | 5 | 0.166 | PUROW B | 1 | 0.033 |
| LIAO W | 5 | 0.166 | PUPPO M | 1 | 0.033 |
| LIANG Y | 5 | 0.166 | PUMMER K | 1 | 0.033 |
| LIAN YF | 5 | 0.166 | PULLAMSETTI SS | 1 | 0.033 |
| LI YM | 5 | 0.166 | PULIDO AH | 1 | 0.033 |
| LI YC | 5 | 0.166 | PUGH TJ | 1 | 0.033 |
| LI SQ | 5 | 0.166 | PU ZJ | 1 | 0.033 |
| LI LP | 5 | 0.166 | PU WT | 1 | 0.033 |
| LI K | 5 | 0.166 | PU M | 1 | 0.033 |
| LI JQ | 5 | 0.166 | PU HL | 1 | 0.033 |
| LI JM | 5 | 0.166 | PROUDFOOT NJ | 1 | 0.033 |
| LI JJ | 5 | 0.166 | PROTIVANKOVA M | 1 | 0.033 |
| LI GJ | 5 | 0.166 | PROTACIO A | 1 | 0.033 |
| LI G | 5 | 0.166 | PROSPER MA | 1 | 0.033 |
| LI CS | 5 | 0.166 | PROSPER F | 1 | 0.033 |
| LI CQ | 5 | 0.166 | PROLL S | 1 | 0.033 |
| LI CH | 5 | 0.166 | PROCINO A | 1 | 0.033 |
| LEE S | 5 | 0.166 | PRITCHARD KI | 1 | 0.033 |
| LAM WL | 5 | 0.166 | PRIOR KK | 1 | 0.033 |
| KITAGAWA M | 5 | 0.166 | PRIMIG M | 1 | 0.033 |
| KIM T | 5 | 0.166 | PREVIS RA | 1 | 0.033 |
| KHAVARI PA | 5 | 0.166 | PREUSSNER J | 1 | 0.033 |
| KAPOOR S | 5 | 0.166 | PRESSER A | 1 | 0.033 |
| JIN HY | 5 | 0.166 | PRENNINGER S | 1 | 0.033 |
| JIAO F | 5 | 0.166 | PREISS T | 1 | 0.033 |
| JIANG XY | 5 | 0.166 | PRATCORONA M | 1 | 0.033 |
| JIANG XM | 5 | 0.166 | PRASAD R | 1 | 0.033 |
| JIANG JF | 5 | 0.166 | PRADO-CABRERO A | 1 | 0.033 |
| JI CB | 5 | 0.166 | PRADEEP S | 1 | 0.033 |
| ISIN M | 5 | 0.166 | POZNER G | 1 | 0.033 |
| HUANG H | 5 | 0.166 | POWELL WT | 1 | 0.033 |
| HU ZB | 5 | 0.166 | POWELL BL | 1 | 0.033 |
| HE XJ | 5 | 0.166 | POUTSMA A | 1 | 0.033 |
| HAO DP | 5 | 0.166 | POULAIN L | 1 | 0.033 |
| HAN SM | 5 | 0.166 | POUEYMIROU WT | 1 | 0.033 |
| HAN J | 5 | 0.166 | POTHOULAKIS C | 1 | 0.033 |
| GUO XR | 5 | 0.166 | POSTEPSKA-IGIELSKA A | 1 | 0.033 |
| GUO XB | 5 | 0.166 | POST AB | 1 | 0.033 |
| GRUMMT I | 5 | 0.166 | POSCA J | 1 | 0.033 |
| GONG ZJ | 5 | 0.166 | PORTELA A | 1 | 0.033 |
| GONG W | 5 | 0.166 | PORSE B | 1 | 0.033 |
| GIBB EA | 5 | 0.166 | POPPE B | 1 | 0.033 |
| GE SF | 5 | 0.166 | POPP MWL | 1 | 0.033 |
| GAO WC | 5 | 0.166 | POPADIN K | 1 | 0.033 |
| GAO SG | 5 | 0.166 | POP V | 1 | 0.033 |
| GAO P | 5 | 0.166 | POON C | 1 | 0.033 |
| FOX AH | 5 | 0.166 | PONTOGLIO M | 1 | 0.033 |
| FENG J | 5 | 0.166 | PONTIS J | 1 | 0.033 |
| FATICA A | 5 | 0.166 | PONTECORVI A | 1 | 0.033 |
| FANG ZY | 5 | 0.166 | POMPILIO G | 1 | 0.033 |
| FAN H | 5 | 0.166 | POMMIER A | 1 | 0.033 |
| FAN DM | 5 | 0.166 | POMERANTZEFF P | 1 | 0.033 |
| ESTELLER M | 5 | 0.166 | POMERANTZ M | 1 | 0.033 |
| DUTTA A | 5 | 0.166 | POLYTARCHOU C | 1 | 0.033 |
| DONG PH | 5 | 0.166 | POLYCARPOU-SCHWARZ M | 1 | 0.033 |
| DENG W | 5 | 0.166 | POLLY P | 1 | 0.033 |
| CUI XW | 5 | 0.166 | POLLOCK C | 1 | 0.033 |
| CLARK MB | 5 | 0.166 | POLLET J | 1 | 0.033 |
| CHEN XJ | 5 | 0.166 | POLLEN AA | 1 | 0.033 |
| CHEN SS | 5 | 0.166 | POLLACK JR | 1 | 0.033 |
| CHEN SL | 5 | 0.166 | POLITIS PK | 1 | 0.033 |
| CHEN M | 5 | 0.166 | POLISENO L | 1 | 0.033 |
| CHEN JY | 5 | 0.166 | POKRZYWA C | 1 | 0.033 |
| CHEN JT | 5 | 0.166 | POGACAR Z | 1 | 0.033 |
| CHEN JL | 5 | 0.166 | PLYS AJ | 1 | 0.033 |
| CHEN JF | 5 | 0.166 | PLOW EF | 1 | 0.033 |
| CAO Q | 5 | 0.166 | PLOMIN R | 1 | 0.033 |
| CAI J | 5 | 0.166 | PLOETNER A | 1 | 0.033 |
| BROWN WT | 5 | 0.166 | PLATH M | 1 | 0.033 |
| BHARTIYA D | 5 | 0.166 | PLAT G | 1 | 0.033 |
| BARLOW DP | 5 | 0.166 | PLASSAIS J | 1 | 0.033 |
| BAI Y | 5 | 0.166 | PLANELLO R | 1 | 0.033 |
| ZUCCHELLI S | 4 | 0.133 | PLANELLES V | 1 | 0.033 |
| ZHU ZS | 4 | 0.133 | PLANA O | 1 | 0.033 |
| ZHU X | 4 | 0.133 | PLAISANCE I | 1 | 0.033 |
| ZHU JG | 4 | 0.133 | PLACA JR | 1 | 0.033 |
| ZHOU YF | 4 | 0.133 | PIZZO M | 1 | 0.033 |
| ZHOU XL | 4 | 0.133 | PITCHIAYA S | 1 | 0.033 |
| ZHOU QY | 4 | 0.133 | PISTERS LL | 1 | 0.033 |
| ZHONG JL | 4 | 0.133 | PISEGNA JR | 1 | 0.033 |
| ZHENG YF | 4 | 0.133 | PISCUOGLIO S | 1 | 0.033 |
| ZHENG XX | 4 | 0.133 | PISCIANZ E | 1 | 0.033 |
| ZHENG SY | 4 | 0.133 | PIRINEN M | 1 | 0.033 |
| ZHENG JH | 4 | 0.133 | PIRES JC | 1 | 0.033 |
| ZHENG HT | 4 | 0.133 | PIOVESAN A | 1 | 0.033 |
| ZHAO Q | 4 | 0.133 | PINTO F | 1 | 0.033 |
| ZHAO JM | 4 | 0.133 | PINNER J | 1 | 0.033 |
| ZHAO JJ | 4 | 0.133 | PINNARO C | 1 | 0.033 |
| ZHAO JH | 4 | 0.133 | PINK RC | 1 | 0.033 |
| ZHAO HY | 4 | 0.133 | PING YY | 1 | 0.033 |
| ZHAO G | 4 | 0.133 | PING P | 1 | 0.033 |
| ZHANG ZG | 4 | 0.133 | PING C | 1 | 0.033 |
| ZHANG YX | 4 | 0.133 | PINET F | 1 | 0.033 |
| ZHANG YF | 4 | 0.133 | PINELLO L | 1 | 0.033 |
| ZHANG XF | 4 | 0.133 | PINEL K | 1 | 0.033 |
| ZHANG SY | 4 | 0.133 | PINEAU P | 1 | 0.033 |
| ZHANG SW | 4 | 0.133 | PIMKIN M | 1 | 0.033 |
| ZHANG SR | 4 | 0.133 | PILYUGIN M | 1 | 0.033 |
| ZHANG LY | 4 | 0.133 | PILOTE L | 1 | 0.033 |
| ZHANG JL | 4 | 0.133 | PILORGE P | 1 | 0.033 |
| ZHANG JH | 4 | 0.133 | PILAKKA-KANTHIKEEL S | 1 | 0.033 |
| ZHANG HM | 4 | 0.133 | PIIPPONEN M | 1 | 0.033 |
| ZHANG GW | 4 | 0.133 | PIGAZZI M | 1 | 0.033 |
| ZHANG DQ | 4 | 0.133 | PIERCONTI F | 1 | 0.033 |
| ZHANG AL | 4 | 0.133 | PIERCE LJ | 1 | 0.033 |
| ZHANG A | 4 | 0.133 | PIENTA KJ | 1 | 0.033 |
| ZENG Y | 4 | 0.133 | PIEKOS S | 1 | 0.033 |
| ZANG WQ | 4 | 0.133 | PICCOLO SR | 1 | 0.033 |
| YUAN Y | 4 | 0.133 | PIBAROT P | 1 | 0.033 |
| YUAN W | 4 | 0.133 | PIAO YL | 1 | 0.033 |
| YUAN BB | 4 | 0.133 | PIAO L | 1 | 0.033 |
| YU YH | 4 | 0.133 | PIAO HL | 1 | 0.033 |
| YU WJ | 4 | 0.133 | PIAO DX | 1 | 0.033 |
| YU W | 4 | 0.133 | PIAN C | 1 | 0.033 |
| YU N | 4 | 0.133 | PFEIFER S | 1 | 0.033 |
| YU JY | 4 | 0.133 | PFEIFER K | 1 | 0.033 |
| YOU YP | 4 | 0.133 | PETTERSSON U | 1 | 0.033 |
| YIN L | 4 | 0.133 | PETSCH K | 1 | 0.033 |
| YIN J | 4 | 0.133 | PETROVICS G | 1 | 0.033 |
| YIN CJ | 4 | 0.133 | PETRI A | 1 | 0.033 |
| YE M | 4 | 0.133 | PETIT JS | 1 | 0.033 |
| YE JR | 4 | 0.133 | PETIT F | 1 | 0.033 |
| YE GL | 4 | 0.133 | PETIMAR JS | 1 | 0.033 |
| YAO ZH | 4 | 0.133 | PETERS G | 1 | 0.033 |
| YAO HR | 4 | 0.133 | PETER S | 1 | 0.033 |
| YANG ZG | 4 | 0.133 | PETELL CJ | 1 | 0.033 |
| YANG XJ | 4 | 0.133 | PETAZZI P | 1 | 0.033 |
| YANG XB | 4 | 0.133 | PESCHANSKY V | 1 | 0.033 |
| YANG TX | 4 | 0.133 | PERSON RE | 1 | 0.033 |
| YANG SM | 4 | 0.133 | PERRY RB | 1 | 0.033 |
| YANG QY | 4 | 0.133 | PERRY AS | 1 | 0.033 |
| YANG JY | 4 | 0.133 | PERROTTI LI | 1 | 0.033 |
| XU YQ | 4 | 0.133 | PERICAY C | 1 | 0.033 |
| XU XL | 4 | 0.133 | PEREZ EM | 1 | 0.033 |
| XU WD | 4 | 0.133 | PERES-DA-SILVA A | 1 | 0.033 |
| XU SP | 4 | 0.133 | PEREIRA S | 1 | 0.033 |
| XU PF | 4 | 0.133 | PEREIRA CG | 1 | 0.033 |
| XU N | 4 | 0.133 | PERDONA S | 1 | 0.033 |
| XU JF | 4 | 0.133 | PERDEAUX ER | 1 | 0.033 |
| XU HM | 4 | 0.133 | PERAKIS S | 1 | 0.033 |
| XU G | 4 | 0.133 | PEPKE S | 1 | 0.033 |
| XU CJ | 4 | 0.133 | PENKALA I | 1 | 0.033 |
| XU CH | 4 | 0.133 | PENIKIS A | 1 | 0.033 |
| XIONG XD | 4 | 0.133 | PENG ZZ | 1 | 0.033 |
| XIE Y | 4 | 0.133 | PENG ZX | 1 | 0.033 |
| XIE W | 4 | 0.133 | PENG ZQ | 1 | 0.033 |
| XIE R | 4 | 0.133 | PENG YZ | 1 | 0.033 |
| XIE HW | 4 | 0.133 | PENG WZ | 1 | 0.033 |
| XIAO K | 4 | 0.133 | PENG WJ | 1 | 0.033 |
| XIANG B | 4 | 0.133 | PENG WH | 1 | 0.033 |
| WU ZD | 4 | 0.133 | PENG SW | 1 | 0.033 |
| WU YY | 4 | 0.133 | PENG RJ | 1 | 0.033 |
| WU YG | 4 | 0.133 | PENG R | 1 | 0.033 |
| WU YF | 4 | 0.133 | PENG QL | 1 | 0.033 |
| WU X | 4 | 0.133 | PENG Q | 1 | 0.033 |
| WU SZ | 4 | 0.133 | PENG M | 1 | 0.033 |
| WU QY | 4 | 0.133 | PENG JY | 1 | 0.033 |
| WU P | 4 | 0.133 | PENG JH | 1 | 0.033 |
| WU M | 4 | 0.133 | PENG JG | 1 | 0.033 |
| WU LM | 4 | 0.133 | PENG HR | 1 | 0.033 |
| WU JB | 4 | 0.133 | PENG FY | 1 | 0.033 |
| WU HC | 4 | 0.133 | PENG F | 1 | 0.033 |
| WU GZ | 4 | 0.133 | PENG CX | 1 | 0.033 |
| WU DD | 4 | 0.133 | PENG CW | 1 | 0.033 |
| WU D | 4 | 0.133 | PENG CJ | 1 | 0.033 |
| WU CH | 4 | 0.133 | PENG BW | 1 | 0.033 |
| WENG WW | 4 | 0.133 | PENG BT | 1 | 0.033 |
| WEN J | 4 | 0.133 | PENG BG | 1 | 0.033 |
| WEINSTEIN JN | 4 | 0.133 | PENAS C | 1 | 0.033 |
| WEI N | 4 | 0.133 | PELTONEN S | 1 | 0.033 |
| WEI GF | 4 | 0.133 | PELTONEN L | 1 | 0.033 |
| WEI F | 4 | 0.133 | PELTONEN J | 1 | 0.033 |
| WANG ZQ | 4 | 0.133 | PELIN M | 1 | 0.033 |
| WANG ZN | 4 | 0.133 | PELICANO H | 1 | 0.033 |
| WANG ZG | 4 | 0.133 | PEI Z | 1 | 0.033 |
| WANG YD | 4 | 0.133 | PEI WW | 1 | 0.033 |
| WANG XX | 4 | 0.133 | PEI J | 1 | 0.033 |
| WANG XM | 4 | 0.133 | PEI D | 1 | 0.033 |
| WANG XL | 4 | 0.133 | PEI CP | 1 | 0.033 |
| WANG XD | 4 | 0.133 | PEFANIS E | 1 | 0.033 |
| WANG WL | 4 | 0.133 | PEDROTTI S | 1 | 0.033 |
| WANG SJ | 4 | 0.133 | PEDROSA E | 1 | 0.033 |
| WANG QY | 4 | 0.133 | PEDERSEN SK | 1 | 0.033 |
| WANG LW | 4 | 0.133 | PEDERSEN JS | 1 | 0.033 |
| WANG JR | 4 | 0.133 | PEARSON RD | 1 | 0.033 |
| WANG HL | 4 | 0.133 | PEARSON MJ | 1 | 0.033 |
| WANG HF | 4 | 0.133 | PAYUMO AY | 1 | 0.033 |
| WANG GX | 4 | 0.133 | PAWLIK KM | 1 | 0.033 |
| WANG GC | 4 | 0.133 | PAUWELS J | 1 | 0.033 |
| WANG CM | 4 | 0.133 | PAULSON A | 1 | 0.033 |
| WAN L | 4 | 0.133 | PAULSEN MT | 1 | 0.033 |
| VELMESHEV D | 4 | 0.133 | PAULI JP | 1 | 0.033 |
| VANCE KW | 4 | 0.133 | PAULES M | 1 | 0.033 |
| VALADKHAN S | 4 | 0.133 | PAUL P | 1 | 0.033 |
| TUO L | 4 | 0.133 | PATRUCCO L | 1 | 0.033 |
| TONG N | 4 | 0.133 | PATIL VS | 1 | 0.033 |
| TEE AE | 4 | 0.133 | PATIL S | 1 | 0.033 |
| TAO QF | 4 | 0.133 | PATELLA F | 1 | 0.033 |
| TANI H | 4 | 0.133 | PATEL R | 1 | 0.033 |
| SUN ZG | 4 | 0.133 | PATEL P | 1 | 0.033 |
| SUN X | 4 | 0.133 | PATEL NA | 1 | 0.033 |
| SUN S | 4 | 0.133 | PATEL LR | 1 | 0.033 |
| SUN K | 4 | 0.133 | PASZKOWSKI-ROGACZ M | 1 | 0.033 |
| SUN JX | 4 | 0.133 | PASUTTO F | 1 | 0.033 |
| SUN HL | 4 | 0.133 | PASQUALINI R | 1 | 0.033 |
| SUN FY | 4 | 0.133 | PASMANT E | 1 | 0.033 |
| SUN F | 4 | 0.133 | PASIERBEK P | 1 | 0.033 |
| SUN D | 4 | 0.133 | PASCUZZI PE | 1 | 0.033 |
| SU XP | 4 | 0.133 | PASCULLI B | 1 | 0.033 |
| SU J | 4 | 0.133 | PARTIN A | 1 | 0.033 |
| SU B | 4 | 0.133 | PARREIRA KS | 1 | 0.033 |
| SPURLOCK CF | 4 | 0.133 | PAROLO S | 1 | 0.033 |
| SPITALE RC | 4 | 0.133 | PARK YJ | 1 | 0.033 |
| SPANO JP | 4 | 0.133 | PARK SS | 1 | 0.033 |
| SONG YX | 4 | 0.133 | PARK SM | 1 | 0.033 |
| SONG X | 4 | 0.133 | PARK SJ | 1 | 0.033 |
| SONG EW | 4 | 0.133 | PARK SC | 1 | 0.033 |
| SKOGERBO G | 4 | 0.133 | PARK SA | 1 | 0.033 |
| SIPRASHVILI Z | 4 | 0.133 | PARK PK | 1 | 0.033 |
| SHI YL | 4 | 0.133 | PARK KY | 1 | 0.033 |
| SHEN ZY | 4 | 0.133 | PARK K | 1 | 0.033 |
| SHEN Z | 4 | 0.133 | PARK JT | 1 | 0.033 |
| SHEN YH | 4 | 0.133 | PARK JK | 1 | 0.033 |
| SHEN X | 4 | 0.133 | PARK JE | 1 | 0.033 |
| SHEN R | 4 | 0.133 | PARK J | 1 | 0.033 |
| SHEN JX | 4 | 0.133 | PARK IH | 1 | 0.033 |
| SHEN HB | 4 | 0.133 | PARK HY | 1 | 0.033 |
| SHEN D | 4 | 0.133 | PARK HJ | 1 | 0.033 |
| SHANG C | 4 | 0.133 | PARK CH | 1 | 0.033 |
| SHAN K | 4 | 0.133 | PARK C | 1 | 0.033 |
| SATI S | 4 | 0.133 | PARIKSHAK NN | 1 | 0.033 |
| SANCHEZ Y | 4 | 0.133 | PARI GS | 1 | 0.033 |
| RUAN XB | 4 | 0.133 | PAREKH VI | 1 | 0.033 |
| REON BJ | 4 | 0.133 | PAREKH C | 1 | 0.033 |
| REN SX | 4 | 0.133 | PARASRAMKA MA | 1 | 0.033 |
| REIS EM | 4 | 0.133 | PARASKEVOPOULOU MD | 1 | 0.033 |
| RATH PC | 4 | 0.133 | PARAMIO JM | 1 | 0.033 |
| QUINN JJ | 4 | 0.133 | PARADIS M | 1 | 0.033 |
| QU Q | 4 | 0.133 | PAPAVASILIOU FN | 1 | 0.033 |
| QIN YY | 4 | 0.133 | PAPAIT R | 1 | 0.033 |
| PRASANTH SG | 4 | 0.133 | PAPAIOANNOU D | 1 | 0.033 |
| PENG ZH | 4 | 0.133 | PAPAGIANNAKOPOULOS T | 1 | 0.033 |
| PENG Y | 4 | 0.133 | PANZERI I | 1 | 0.033 |
| PENG L | 4 | 0.133 | PANWAR B | 1 | 0.033 |
| PAULER FM | 4 | 0.133 | PANJA D | 1 | 0.033 |
| PANG D | 4 | 0.133 | PANIGRAHI AK | 1 | 0.033 |
| PANDEY GK | 4 | 0.133 | PANG SY | 1 | 0.033 |
| PAN YS | 4 | 0.133 | PANG Q | 1 | 0.033 |
| PAN WT | 4 | 0.133 | PANG EJ | 1 | 0.033 |
| PAN T | 4 | 0.133 | PANDEY A | 1 | 0.033 |
| PAN JJ | 4 | 0.133 | PANDA A | 1 | 0.033 |
| OZGUR E | 4 | 0.133 | PANCHAPAKESAN U | 1 | 0.033 |
| NERI A | 4 | 0.133 | PAN ZY | 1 | 0.033 |
| NATARAJAN R | 4 | 0.133 | PAN YZ | 1 | 0.033 |
| NAGANO T | 4 | 0.133 | PAN YT | 1 | 0.033 |
| MORLANDO M | 4 | 0.133 | PAN XM | 1 | 0.033 |
| MONDAL T | 4 | 0.133 | PAN SL | 1 | 0.033 |
| MIMORI K | 4 | 0.133 | PAN SD | 1 | 0.033 |
| MIAO RY | 4 | 0.133 | PAN S | 1 | 0.033 |
| MIANO JM | 4 | 0.133 | PAN P | 1 | 0.033 |
| MERRY CR | 4 | 0.133 | PAN LJ | 1 | 0.033 |
| MENG H | 4 | 0.133 | PAN L | 1 | 0.033 |
| MEHLER MF | 4 | 0.133 | PAN K | 1 | 0.033 |
| MAZAR J | 4 | 0.133 | PAN JH | 1 | 0.033 |
| MARTINDALE JL | 4 | 0.133 | PAN HT | 1 | 0.033 |
| MARSHALL GM | 4 | 0.133 | PAN HL | 1 | 0.033 |
| MARCHESE FP | 4 | 0.133 | PAN HG | 1 | 0.033 |
| MANDAL SS | 4 | 0.133 | PAN GF | 1 | 0.033 |
| MALOUF GG | 4 | 0.133 | PAN BJ | 1 | 0.033 |
| MA P | 4 | 0.133 | PALTI Y | 1 | 0.033 |
| MA N | 4 | 0.133 | PALMER CNA | 1 | 0.033 |
| MA JZ | 4 | 0.133 | PALMA A | 1 | 0.033 |
| MA C | 4 | 0.133 | PALLAS V | 1 | 0.033 |
| LV L | 4 | 0.133 | PALANISAMY K | 1 | 0.033 |
| LUO J | 4 | 0.133 | PAL K | 1 | 0.033 |
| LUO HX | 4 | 0.133 | PAITHANKAR S | 1 | 0.033 |
| LU JH | 4 | 0.133 | PAIDAS M | 1 | 0.033 |
| LU DD | 4 | 0.133 | PAGNANO KBB | 1 | 0.033 |
| LU C | 4 | 0.133 | PAGLIALUNGA L | 1 | 0.033 |
| LORENZEN JM | 4 | 0.133 | PAGANI M | 1 | 0.033 |
| LIU ZS | 4 | 0.133 | PADUA D | 1 | 0.033 |
| LIU ZQ | 4 | 0.133 | PADOVAN O | 1 | 0.033 |
| LIU YY | 4 | 0.133 | PACIULLO F | 1 | 0.033 |
| LIU YT | 4 | 0.133 | PACIK D | 1 | 0.033 |
| LIU YB | 4 | 0.133 | OZKAN G | 1 | 0.033 |
| LIU XS | 4 | 0.133 | OZGUR S | 1 | 0.033 |
| LIU XG | 4 | 0.133 | OZAWA T | 1 | 0.033 |
| LIU XF | 4 | 0.133 | OZANOVA Z | 1 | 0.033 |
| LIU SY | 4 | 0.133 | OZAKI M | 1 | 0.033 |
| LIU SS | 4 | 0.133 | OYAMA M | 1 | 0.033 |
| LIU PY | 4 | 0.133 | OXLEY D | 1 | 0.033 |
| LIU JL | 4 | 0.133 | OWZAR K | 1 | 0.033 |
| LIU JH | 4 | 0.133 | OVSEPIAN SV | 1 | 0.033 |
| LIU GY | 4 | 0.133 | OVERBEEK PA | 1 | 0.033 |
| LIU CJ | 4 | 0.133 | OUYANG YR | 1 | 0.033 |
| LIU CC | 4 | 0.133 | OUYANG YD | 1 | 0.033 |
| LIU BY | 4 | 0.133 | OUYANG L | 1 | 0.033 |
| LIN YY | 4 | 0.133 | OUSAGER LB | 1 | 0.033 |
| LIN CY | 4 | 0.133 | OUDEJANS CBM | 1 | 0.033 |
| LIM DA | 4 | 0.133 | OUCHI Y | 1 | 0.033 |
| LIANG H | 4 | 0.133 | OU ZS | 1 | 0.033 |
| LI YL | 4 | 0.133 | OU YX | 1 | 0.033 |
| LI YG | 4 | 0.133 | OU ML | 1 | 0.033 |
| LI XX | 4 | 0.133 | OU K | 1 | 0.033 |
| LI XP | 4 | 0.133 | OU J | 1 | 0.033 |
| LI XN | 4 | 0.133 | OU CL | 1 | 0.033 |
| LI XJ | 4 | 0.133 | OTTO S | 1 | 0.033 |
| LI XF | 4 | 0.133 | OTT G | 1 | 0.033 |
| LI XD | 4 | 0.133 | OTSUJI E | 1 | 0.033 |
| LI LL | 4 | 0.133 | OSTENSSON M | 1 | 0.033 |
| LI JW | 4 | 0.133 | OSINSKI T | 1 | 0.033 |
| LI JC | 4 | 0.133 | OSHIMURA M | 1 | 0.033 |
| LI HX | 4 | 0.133 | OSAK M | 1 | 0.033 |
| LI D | 4 | 0.133 | OSADA T | 1 | 0.033 |
| LI CX | 4 | 0.133 | OS NF | 1 | 0.033 |
| LI BS | 4 | 0.133 | ORTLUND EA | 1 | 0.033 |
| LEUCCI E | 4 | 0.133 | ORTIN J | 1 | 0.033 |
| LEE JH | 4 | 0.133 | ORSINI M | 1 | 0.033 |
| LEE J | 4 | 0.133 | ORLANDO DA | 1 | 0.033 |
| KOUR S | 4 | 0.133 | ORKIN SH | 1 | 0.033 |
| KITAGAWA K | 4 | 0.133 | ORJALO AV | 1 | 0.033 |
| KIM HJ | 4 | 0.133 | ORJALO A | 1 | 0.033 |
| KIM D | 4 | 0.133 | ORANTIA M | 1 | 0.033 |
| KHAYAT D | 4 | 0.133 | ORANG AV | 1 | 0.033 |
| KELLIS M | 4 | 0.133 | OPITZ I | 1 | 0.033 |
| KAWAGUCHI T | 4 | 0.133 | OONISHI T | 1 | 0.033 |
| KATSAROS D | 4 | 0.133 | OOI HS | 1 | 0.033 |
| JIN L | 4 | 0.133 | ONUMA Y | 1 | 0.033 |
| JIANG X | 4 | 0.133 | ONUCHIC V | 1 | 0.033 |
| JIANG W | 4 | 0.133 | ONO H | 1 | 0.033 |
| JIANG S | 4 | 0.133 | ONGKEKO WM | 1 | 0.033 |
| JIANG QH | 4 | 0.133 | ONDER T | 1 | 0.033 |
| JIANG J | 4 | 0.133 | OLSEN D | 1 | 0.033 |
| JIANG F | 4 | 0.133 | OLLIKAINEN N | 1 | 0.033 |
| JIANG CY | 4 | 0.133 | OLIVIERO S | 1 | 0.033 |
| JIA LF | 4 | 0.133 | OLIVIERI M | 1 | 0.033 |
| JI L | 4 | 0.133 | OLDRIDGE DA | 1 | 0.033 |
| JENKINS EC | 4 | 0.133 | OLDHAM MC | 1 | 0.033 |
| HUANG Z | 4 | 0.133 | OLCESE U | 1 | 0.033 |
| HUANG W | 4 | 0.133 | OLANICH ME | 1 | 0.033 |
| HUANG S | 4 | 0.133 | OKUTAN M | 1 | 0.033 |
| HUANG P | 4 | 0.133 | OKUNO M | 1 | 0.033 |
| HUANG M | 4 | 0.133 | OKUMURA S | 1 | 0.033 |
| HUANG L | 4 | 0.133 | OKUMA K | 1 | 0.033 |
| HUANG JF | 4 | 0.133 | OKUGAWA Y | 1 | 0.033 |
| HUANG DS | 4 | 0.133 | OKANO HJ | 1 | 0.033 |
| HUANG D | 4 | 0.133 | OKANO H | 1 | 0.033 |
| HUA QH | 4 | 0.133 | OKAMOTO K | 1 | 0.033 |
| HUA LX | 4 | 0.133 | OKAMOTO H | 1 | 0.033 |
| HU T | 4 | 0.133 | OKADA T | 1 | 0.033 |
| HU SY | 4 | 0.133 | OJHA S | 1 | 0.033 |
| HU L | 4 | 0.133 | OHYANAGI H | 1 | 0.033 |
| HU JF | 4 | 0.133 | OHTOMO K | 1 | 0.033 |
| HU JC | 4 | 0.133 | OHLER U | 1 | 0.033 |
| HU HY | 4 | 0.133 | OHIRA T | 1 | 0.033 |
| HU GH | 4 | 0.133 | OHGI KA | 1 | 0.033 |
| HOU JG | 4 | 0.133 | OHASHI T | 1 | 0.033 |
| HOFFMAN AR | 4 | 0.133 | OH YM | 1 | 0.033 |
| HEYMANS S | 4 | 0.133 | OH JS | 1 | 0.033 |
| HENNELLY SP | 4 | 0.133 | OH HJ | 1 | 0.033 |
| HE YH | 4 | 0.133 | OGAWA Y | 1 | 0.033 |
| HE L | 4 | 0.133 | OGAWA H | 1 | 0.033 |
| HE B | 4 | 0.133 | OELJEKLAUS S | 1 | 0.033 |
| HAO TF | 4 | 0.133 | ODA A | 1 | 0.033 |
| HAN ZB | 4 | 0.133 | OBSTETER J | 1 | 0.033 |
| HAN P | 4 | 0.133 | OBERDOERFFER P | 1 | 0.033 |
| HAN D | 4 | 0.133 | OAKES C | 1 | 0.033 |
| HAN BW | 4 | 0.133 | O'NEILL LAJ | 1 | 0.033 |
| GUTSCHNER T | 4 | 0.133 | O'MALLEY BW | 1 | 0.033 |
| GUSTINCICH S | 4 | 0.133 | O'LEARY VB | 1 | 0.033 |
| GUO YJ | 4 | 0.133 | O'KEEFFE AJ | 1 | 0.033 |
| GUO SL | 4 | 0.133 | O'CONNOR CL | 1 | 0.033 |
| GUO RH | 4 | 0.133 | O'BYRNE KJ | 1 | 0.033 |
| GUO MN | 4 | 0.133 | NYKTER M | 1 | 0.033 |
| GROTE P | 4 | 0.133 | NYBERG KG | 1 | 0.033 |
| GONG CG | 4 | 0.133 | NWIGWE IJ | 1 | 0.033 |
| GEZER U | 4 | 0.133 | NUSBAUM C | 1 | 0.033 |
| GENG B | 4 | 0.133 | NURNBERG ST | 1 | 0.033 |
| GE YX | 4 | 0.133 | NUOVO G | 1 | 0.033 |
| GAO YZ | 4 | 0.133 | NUNES DN | 1 | 0.033 |
| GAO YJ | 4 | 0.133 | NTZIACHRISTOS P | 1 | 0.033 |
| GAO M | 4 | 0.133 | NSAIBIA MJ | 1 | 0.033 |
| GAO JY | 4 | 0.133 | NOVINA CD | 1 | 0.033 |
| GAO F | 4 | 0.133 | NOVELLINO E | 1 | 0.033 |
| GAO C | 4 | 0.133 | NOTARANGELO LD | 1 | 0.033 |
| FU X | 4 | 0.133 | NORTON L | 1 | 0.033 |
| FU HJ | 4 | 0.133 | NORTHWOOD KS | 1 | 0.033 |
| FRASER P | 4 | 0.133 | NOON A | 1 | 0.033 |
| FENG XS | 4 | 0.133 | NONOMURA KI | 1 | 0.033 |
| FENG WW | 4 | 0.133 | NONNEKENS J | 1 | 0.033 |
| FAN L | 4 | 0.133 | NONES K | 1 | 0.033 |
| DU Z | 4 | 0.133 | NOMDEDEU M | 1 | 0.033 |
| DU Y | 4 | 0.133 | NOMDEDEU J | 1 | 0.033 |
| DU ML | 4 | 0.133 | NOHATA N | 1 | 0.033 |
| DU LT | 4 | 0.133 | NOH SM | 1 | 0.033 |
| DING XY | 4 | 0.133 | NOH SH | 1 | 0.033 |
| DING L | 4 | 0.133 | NOCITI V | 1 | 0.033 |
| DHANASEKARAN SM | 4 | 0.133 | NOBILI L | 1 | 0.033 |
| DEVAUX Y | 4 | 0.133 | NJUGUNA P | 1 | 0.033 |
| DERRIEN T | 4 | 0.133 | NIZRI T | 1 | 0.033 |
| DENG Y | 4 | 0.133 | NIYAZI M | 1 | 0.033 |
| DE S | 4 | 0.133 | NIVET E | 1 | 0.033 |
| DALAY N | 4 | 0.133 | NIU ZQ | 1 | 0.033 |
| CUI W | 4 | 0.133 | NIU ZG | 1 | 0.033 |
| CUI M | 4 | 0.133 | NIU YM | 1 | 0.033 |
| CHU C | 4 | 0.133 | NIU YC | 1 | 0.033 |
| CHENG J | 4 | 0.133 | NIU Y | 1 | 0.033 |
| CHENG G | 4 | 0.133 | NIU XM | 1 | 0.033 |
| CHEN YY | 4 | 0.133 | NIU XL | 1 | 0.033 |
| CHEN YJ | 4 | 0.133 | NIU QW | 1 | 0.033 |
| CHEN XL | 4 | 0.133 | NIU PY | 1 | 0.033 |
| CHEN XB | 4 | 0.133 | NIU LL | 1 | 0.033 |
| CHEN SC | 4 | 0.133 | NIU JJ | 1 | 0.033 |
| CHEN RF | 4 | 0.133 | NIU HT | 1 | 0.033 |
| CHEN R | 4 | 0.133 | NIU H | 1 | 0.033 |
| CHEN QJ | 4 | 0.133 | NIU CN | 1 | 0.033 |
| CHEN LY | 4 | 0.133 | NIU BB | 1 | 0.033 |
| CHEN GY | 4 | 0.133 | NITZSCHE A | 1 | 0.033 |
| CHEN FF | 4 | 0.133 | NITSCHE A | 1 | 0.033 |
| CHEN B | 4 | 0.133 | NISSINEN L | 1 | 0.033 |
| CARMICHAEL GG | 4 | 0.133 | NISSAN A | 1 | 0.033 |
| CAO Y | 4 | 0.133 | NISHITSUJI H | 1 | 0.033 |
| CAO W | 4 | 0.133 | NISHIO M | 1 | 0.033 |
| CAO HM | 4 | 0.133 | NISHIMOTO Y | 1 | 0.033 |
| CAO H | 4 | 0.133 | NISHIDA Y | 1 | 0.033 |
| CAI Q | 4 | 0.133 | NING Y | 1 | 0.033 |
| CAI H | 4 | 0.133 | NING QQ | 1 | 0.033 |
| CAFFREY DR | 4 | 0.133 | NING Q | 1 | 0.033 |
| BUSSOTTI G | 4 | 0.133 | NING L | 1 | 0.033 |
| BOHMDORFER G | 4 | 0.133 | NING JY | 1 | 0.033 |
| BLACKSHAW S | 4 | 0.133 | NING FL | 1 | 0.033 |
| BHAN A | 4 | 0.133 | NING BF | 1 | 0.033 |
| BERINDAN-NEAGOE I | 4 | 0.133 | NIINUMA T | 1 | 0.033 |
| BARTEL DP | 4 | 0.133 | NIIHAMA M | 1 | 0.033 |
| BARRIOCANAL M | 4 | 0.133 | NIGRO P | 1 | 0.033 |
| AUNE TM | 4 | 0.133 | NIEVES B | 1 | 0.033 |
| ASKARIAN-AMIRI ME | 4 | 0.133 | NIELSEN MM | 1 | 0.033 |
| ZONG G | 3 | 0.1 | NIELSEN LK | 1 | 0.033 |
| ZHU ZM | 3 | 0.1 | NIEHRS C | 1 | 0.033 |
| ZHU ZG | 3 | 0.1 | NIEGISCH G | 1 | 0.033 |
| ZHU YS | 3 | 0.1 | NIEDWOROK C | 1 | 0.033 |
| ZHU YP | 3 | 0.1 | NIE YQ | 1 | 0.033 |
| ZHU YN | 3 | 0.1 | NIE XL | 1 | 0.033 |
| ZHU XH | 3 | 0.1 | NIE WP | 1 | 0.033 |
| ZHU W | 3 | 0.1 | NIE Q | 1 | 0.033 |
| ZHU T | 3 | 0.1 | NIE L | 1 | 0.033 |
| ZHU SS | 3 | 0.1 | NIE JW | 1 | 0.033 |
| ZHU QQ | 3 | 0.1 | NIE JH | 1 | 0.033 |
| ZHU N | 3 | 0.1 | NICOLET D | 1 | 0.033 |
| ZHU HL | 3 | 0.1 | NICOLAS A | 1 | 0.033 |
| ZHU DX | 3 | 0.1 | NICLOU SP | 1 | 0.033 |
| ZHU CP | 3 | 0.1 | NICHOLSON SA | 1 | 0.033 |
| ZHOU YX | 3 | 0.1 | NICHOLSON CO | 1 | 0.033 |
| ZHOU YQ | 3 | 0.1 | NIAN X | 1 | 0.033 |
| ZHOU YH | 3 | 0.1 | NI ZH | 1 | 0.033 |
| ZHOU XH | 3 | 0.1 | NI ZF | 1 | 0.033 |
| ZHOU W | 3 | 0.1 | NI YY | 1 | 0.033 |
| ZHOU T | 3 | 0.1 | NI YJ | 1 | 0.033 |
| ZHOU SL | 3 | 0.1 | NI Y | 1 | 0.033 |
| ZHOU RM | 3 | 0.1 | NI XL | 1 | 0.033 |
| ZHOU QB | 3 | 0.1 | NI XH | 1 | 0.033 |
| ZHOU JD | 3 | 0.1 | NI XB | 1 | 0.033 |
| ZHOU CX | 3 | 0.1 | NI WF | 1 | 0.033 |
| ZHI KK | 3 | 0.1 | NI S | 1 | 0.033 |
| ZHENG Z | 3 | 0.1 | NI PZ | 1 | 0.033 |
| ZHENG YH | 3 | 0.1 | NI PH | 1 | 0.033 |
| ZHENG X | 3 | 0.1 | NI LC | 1 | 0.033 |
| ZHENG T | 3 | 0.1 | NI JW | 1 | 0.033 |
| ZHENG S | 3 | 0.1 | NI J | 1 | 0.033 |
| ZHENG QD | 3 | 0.1 | NI HY | 1 | 0.033 |
| ZHENG P | 3 | 0.1 | NI HJ | 1 | 0.033 |
| ZHENG LM | 3 | 0.1 | NI HB | 1 | 0.033 |
| ZHENG GXY | 3 | 0.1 | NI F | 1 | 0.033 |
| ZHENG DY | 3 | 0.1 | NI BB | 1 | 0.033 |
| ZHAO ZXL | 3 | 0.1 | NGUYEN TB | 1 | 0.033 |
| ZHAO SL | 3 | 0.1 | NGUYEN Q | 1 | 0.033 |
| ZHAO SH | 3 | 0.1 | NGUYEN DH | 1 | 0.033 |
| ZHAO S | 3 | 0.1 | NGUYEN A | 1 | 0.033 |
| ZHAO P | 3 | 0.1 | NGOK SP | 1 | 0.033 |
| ZHAO JZ | 3 | 0.1 | NGANKEU A | 1 | 0.033 |
| ZHAO JY | 3 | 0.1 | NG RT | 1 | 0.033 |
| ZHAO JL | 3 | 0.1 | NG IOL | 1 | 0.033 |
| ZHAO JC | 3 | 0.1 | NG HH | 1 | 0.033 |
| ZHAO HX | 3 | 0.1 | NG CP | 1 | 0.033 |
| ZHAO CZ | 3 | 0.1 | NEWSHAM I | 1 | 0.033 |
| ZHANG ZW | 3 | 0.1 | NEWBY DE | 1 | 0.033 |
| ZHANG ZQ | 3 | 0.1 | NEWBURGER PE | 1 | 0.033 |
| ZHANG YL | 3 | 0.1 | NEVO N | 1 | 0.033 |
| ZHANG YE | 3 | 0.1 | NEUMANN RS | 1 | 0.033 |
| ZHANG XT | 3 | 0.1 | NEUMANN K | 1 | 0.033 |
| ZHANG XP | 3 | 0.1 | NEUERT G | 1 | 0.033 |
| ZHANG XO | 3 | 0.1 | NESS RW | 1 | 0.033 |
| ZHANG XN | 3 | 0.1 | NERI M | 1 | 0.033 |
| ZHANG WM | 3 | 0.1 | NEPOMUCENO R | 1 | 0.033 |
| ZHANG TT | 3 | 0.1 | NEPHEW KP | 1 | 0.033 |
| ZHANG SL | 3 | 0.1 | NEPAL C | 1 | 0.033 |
| ZHANG SJ | 3 | 0.1 | NEMIR M | 1 | 0.033 |
| ZHANG SH | 3 | 0.1 | NEMET J | 1 | 0.033 |
| ZHANG RX | 3 | 0.1 | NELSON RG | 1 | 0.033 |
| ZHANG QX | 3 | 0.1 | NELSON C | 1 | 0.033 |
| ZHANG MY | 3 | 0.1 | NELSON ADL | 1 | 0.033 |
| ZHANG ML | 3 | 0.1 | NELLORE A | 1 | 0.033 |
| ZHANG MJ | 3 | 0.1 | NELLES DA | 1 | 0.033 |
| ZHANG LW | 3 | 0.1 | NEGUEMBOR MV | 1 | 0.033 |
| ZHANG LM | 3 | 0.1 | NEGRINI M | 1 | 0.033 |
| ZHANG LL | 3 | 0.1 | NEGISHI M | 1 | 0.033 |
| ZHANG JS | 3 | 0.1 | NEESEN J | 1 | 0.033 |
| ZHANG JB | 3 | 0.1 | NEELAPU S | 1 | 0.033 |
| ZHANG HY | 3 | 0.1 | NDUNGU AW | 1 | 0.033 |
| ZHANG GJ | 3 | 0.1 | NDILA C | 1 | 0.033 |
| ZHANG FR | 3 | 0.1 | NAYLER SP | 1 | 0.033 |
| ZHANG E | 3 | 0.1 | NAVONE NM | 1 | 0.033 |
| ZHANG DY | 3 | 0.1 | NAVONE N | 1 | 0.033 |
| ZHANG DS | 3 | 0.1 | NAVARRO IC | 1 | 0.033 |
| ZHANG D | 3 | 0.1 | NAUME B | 1 | 0.033 |
| ZHANG CJ | 3 | 0.1 | NATOWICZ MR | 1 | 0.033 |
| ZHANG BL | 3 | 0.1 | NATHANSON KL | 1 | 0.033 |
| ZHAI W | 3 | 0.1 | NASU E | 1 | 0.033 |
| ZENG TB | 3 | 0.1 | NARANBHAI V | 1 | 0.033 |
| ZENG J | 3 | 0.1 | NANNI S | 1 | 0.033 |
| ZENG FQ | 3 | 0.1 | NANDA V | 1 | 0.033 |
| ZENG CY | 3 | 0.1 | NAN YM | 1 | 0.033 |
| ZENG CW | 3 | 0.1 | NAN YK | 1 | 0.033 |
| ZEIER Z | 3 | 0.1 | NAN AO | 1 | 0.033 |
| YUE HY | 3 | 0.1 | NAN A | 1 | 0.033 |
| YUAN XL | 3 | 0.1 | NAMEKAWA S | 1 | 0.033 |
| YUAN L | 3 | 0.1 | NAM SW | 1 | 0.033 |
| YUAN JP | 3 | 0.1 | NAM KH | 1 | 0.033 |
| YUAN H | 3 | 0.1 | NAM JW | 1 | 0.033 |
| YU ZB | 3 | 0.1 | NAKKA VP | 1 | 0.033 |
| YU XQ | 3 | 0.1 | NAKAYAMA W | 1 | 0.033 |
| YU WW | 3 | 0.1 | NAKAYA HI | 1 | 0.033 |
| YU M | 3 | 0.1 | NAKASHIMA T | 1 | 0.033 |
| YU LK | 3 | 0.1 | NAKASHIMA K | 1 | 0.033 |
| YU CY | 3 | 0.1 | NAKANO S | 1 | 0.033 |
| YU C | 3 | 0.1 | NAKANO M | 1 | 0.033 |
| YOU ZL | 3 | 0.1 | NAKANO I | 1 | 0.033 |
| YOU ZH | 3 | 0.1 | NAKANISHI N | 1 | 0.033 |
| YING MF | 3 | 0.1 | NAKANISHI H | 1 | 0.033 |
| YIN ZX | 3 | 0.1 | NAKAMURA T | 1 | 0.033 |
| YIN Y | 3 | 0.1 | NAKAMURA K | 1 | 0.033 |
| YIN JY | 3 | 0.1 | NAKAJIMA K | 1 | 0.033 |
| YIN CQ | 3 | 0.1 | NAKAHARA S | 1 | 0.033 |
| YEH CT | 3 | 0.1 | NAKAGAWA M | 1 | 0.033 |
| YE HL | 3 | 0.1 | NAKAGAWA K | 1 | 0.033 |
| YE H | 3 | 0.1 | NAKAGAWA H | 1 | 0.033 |
| YE DW | 3 | 0.1 | NAKABAYASHI K | 1 | 0.033 |
| YARMISHYN AA | 3 | 0.1 | NAJAFI F | 1 | 0.033 |
| YAO WZ | 3 | 0.1 | NAIR V | 1 | 0.033 |
| YAO MD | 3 | 0.1 | NAIR SS | 1 | 0.033 |
| YAO KH | 3 | 0.1 | NAIR RP | 1 | 0.033 |
| YANG ZH | 3 | 0.1 | NAIR M | 1 | 0.033 |
| YANG YY | 3 | 0.1 | NAINAR S | 1 | 0.033 |
| YANG YN | 3 | 0.1 | NAIK S | 1 | 0.033 |
| YANG YJ | 3 | 0.1 | NAGOSHI H | 1 | 0.033 |
| YANG XQ | 3 | 0.1 | NAGATA T | 1 | 0.033 |
| YANG XP | 3 | 0.1 | NAGATA K | 1 | 0.033 |
| YANG LQ | 3 | 0.1 | NAGATA H | 1 | 0.033 |
| YANG LJ | 3 | 0.1 | NAGARAJA AS | 1 | 0.033 |
| YANG HY | 3 | 0.1 | NAGANO H | 1 | 0.033 |
| YANG HJ | 3 | 0.1 | NA XY | 1 | 0.033 |
| YANG G | 3 | 0.1 | NA L | 1 | 0.033 |
| YANG FQ | 3 | 0.1 | NA J | 1 | 0.033 |
| YAN Y | 3 | 0.1 | MYERS RM | 1 | 0.033 |
| YAN W | 3 | 0.1 | MWARUMBA S | 1 | 0.033 |
| YAN TT | 3 | 0.1 | MWANGI I | 1 | 0.033 |
| YAN R | 3 | 0.1 | MUTHUSAMY M | 1 | 0.033 |
| YAN Q | 3 | 0.1 | MUSTO P | 1 | 0.033 |
| YAN M | 3 | 0.1 | MUSOLINO C | 1 | 0.033 |
| YAN L | 3 | 0.1 | MUSKAVITCH MAT | 1 | 0.033 |
| YAN IK | 3 | 0.1 | MUSAHL AS | 1 | 0.033 |
| YAN DW | 3 | 0.1 | MUSACCHIA F | 1 | 0.033 |
| XUE XC | 3 | 0.1 | MURRE A | 1 | 0.033 |
| XUE X | 3 | 0.1 | MURPHY MB | 1 | 0.033 |
| XUE QZ | 3 | 0.1 | MURAYAMA S | 1 | 0.033 |
| XUE H | 3 | 0.1 | MURAT P | 1 | 0.033 |
| XU ZK | 3 | 0.1 | MURASAKI C | 1 | 0.033 |
| XU Z | 3 | 0.1 | MURAKAMI K | 1 | 0.033 |
| XU XJ | 3 | 0.1 | MUNSON PJ | 1 | 0.033 |
| XU XF | 3 | 0.1 | MUNOZ C | 1 | 0.033 |
| XU WY | 3 | 0.1 | MUNK R | 1 | 0.033 |
| XU RX | 3 | 0.1 | MUNITA R | 1 | 0.033 |
| XU P | 3 | 0.1 | MULVEY BB | 1 | 0.033 |
| XU LY | 3 | 0.1 | MULLOY JC | 1 | 0.033 |
| XU LM | 3 | 0.1 | MULLINS E | 1 | 0.033 |
| XU JY | 3 | 0.1 | MULLIKIN JC | 1 | 0.033 |
| XU HX | 3 | 0.1 | MULLER-TIDOW C | 1 | 0.033 |
| XU EW | 3 | 0.1 | MULLER T | 1 | 0.033 |
| XIU YC | 3 | 0.1 | MULLER S | 1 | 0.033 |
| XIONG J | 3 | 0.1 | MULLER N | 1 | 0.033 |
| XING Z | 3 | 0.1 | MULLER M | 1 | 0.033 |
| XING YH | 3 | 0.1 | MULLER J | 1 | 0.033 |
| XING CG | 3 | 0.1 | MULJO SA | 1 | 0.033 |
| XIE QY | 3 | 0.1 | MULDERS J | 1 | 0.033 |
| XIE F | 3 | 0.1 | MUHAMMAD S | 1 | 0.033 |
| XIAO YF | 3 | 0.1 | MUELLER AC | 1 | 0.033 |
| XIAO XY | 3 | 0.1 | MUEHLBAUER GJ | 1 | 0.033 |
| XIAO TF | 3 | 0.1 | MUDGE JM | 1 | 0.033 |
| XIAO HB | 3 | 0.1 | MUCCI LA | 1 | 0.033 |
| XIA WJ | 3 | 0.1 | MU YL | 1 | 0.033 |
| XIA JZ | 3 | 0.1 | MU Y | 1 | 0.033 |
| XIA HP | 3 | 0.1 | MU XY | 1 | 0.033 |
| XI Y | 3 | 0.1 | MU QQ | 1 | 0.033 |
| WU YS | 3 | 0.1 | MU M | 1 | 0.033 |
| WU YL | 3 | 0.1 | MU HL | 1 | 0.033 |
| WU XW | 3 | 0.1 | MU GN | 1 | 0.033 |
| WU TY | 3 | 0.1 | MU DZ | 1 | 0.033 |
| WU T | 3 | 0.1 | MU C | 1 | 0.033 |
| WU R | 3 | 0.1 | MTURI N | 1 | 0.033 |
| WU MY | 3 | 0.1 | MROZEK K | 1 | 0.033 |
| WU MC | 3 | 0.1 | MOYO B | 1 | 0.033 |
| WU LC | 3 | 0.1 | MOYER MP | 1 | 0.033 |
| WU KC | 3 | 0.1 | MOWEL WK | 1 | 0.033 |
| WU JZ | 3 | 0.1 | MOVAFAGH A | 1 | 0.033 |
| WU JY | 3 | 0.1 | MOUTINHO C | 1 | 0.033 |
| WU CL | 3 | 0.1 | MOUSTAKAS A | 1 | 0.033 |
| WU C | 3 | 0.1 | MOURAVIEV V | 1 | 0.033 |
| WONG TS | 3 | 0.1 | MOUNTFORD JC | 1 | 0.033 |
| WOLFSON B | 3 | 0.1 | MOUM T | 1 | 0.033 |
| WITHOFF S | 3 | 0.1 | MOUAWAD R | 1 | 0.033 |
| WILUSZ JE | 3 | 0.1 | MOU LS | 1 | 0.033 |
| WILKINSON B | 3 | 0.1 | MOTTI D | 1 | 0.033 |
| WEST RB | 3 | 0.1 | MOTOLA DL | 1 | 0.033 |
| WEISS MJ | 3 | 0.1 | MOTOI N | 1 | 0.033 |
| WEINBERG MS | 3 | 0.1 | MOSSINK B | 1 | 0.033 |
| WEI Y | 3 | 0.1 | MOSQUERA JM | 1 | 0.033 |
| WEI Q | 3 | 0.1 | MOSKOWITZ D | 1 | 0.033 |
| WEI JY | 3 | 0.1 | MOSHIRI F | 1 | 0.033 |
| WATABE K | 3 | 0.1 | MOSCATO P | 1 | 0.033 |
| WANG ZL | 3 | 0.1 | MOSAMMAPARAST N | 1 | 0.033 |
| WANG YP | 3 | 0.1 | MORTON ML | 1 | 0.033 |
| WANG YNZ | 3 | 0.1 | MORRISEY EE | 1 | 0.033 |
| WANG YB | 3 | 0.1 | MORPETH SC | 1 | 0.033 |
| WANG XW | 3 | 0.1 | MORIZONO T | 1 | 0.033 |
| WANG XG | 3 | 0.1 | MORIN GB | 1 | 0.033 |
| WANG XB | 3 | 0.1 | MORIKAWA T | 1 | 0.033 |
| WANG WZ | 3 | 0.1 | MORIKAWA M | 1 | 0.033 |
| WANG TZ | 3 | 0.1 | MORGAN AL | 1 | 0.033 |
| WANG QL | 3 | 0.1 | MORETTI F | 1 | 0.033 |
| WANG PL | 3 | 0.1 | MORENO-BUENO G | 1 | 0.033 |
| WANG N | 3 | 0.1 | MORENO I | 1 | 0.033 |
| WANG MB | 3 | 0.1 | MOREIRA YB | 1 | 0.033 |
| WANG LY | 3 | 0.1 | MORCILLO G | 1 | 0.033 |
| WANG LP | 3 | 0.1 | MORANT R | 1 | 0.033 |
| WANG LN | 3 | 0.1 | MORAN-JONES K | 1 | 0.033 |
| WANG KJ | 3 | 0.1 | MORAN VA | 1 | 0.033 |
| WANG JH | 3 | 0.1 | MORALES DR | 1 | 0.033 |
| WANG HX | 3 | 0.1 | MORADIAN A | 1 | 0.033 |
| WANG HM | 3 | 0.1 | MORABITO F | 1 | 0.033 |
| WANG HB | 3 | 0.1 | MORA J | 1 | 0.033 |
| WANG GY | 3 | 0.1 | MOR G | 1 | 0.033 |
| WANG G | 3 | 0.1 | MOQADAM FA | 1 | 0.033 |
| WANG DL | 3 | 0.1 | MOORE T | 1 | 0.033 |
| WANG DH | 3 | 0.1 | MOORE JO | 1 | 0.033 |
| WAN Y | 3 | 0.1 | MOORE JM | 1 | 0.033 |
| WALSH MJ | 3 | 0.1 | MOORE JC | 1 | 0.033 |
| WAGNER DR | 3 | 0.1 | MOORE GE | 1 | 0.033 |
| VUCICEVIC D | 3 | 0.1 | MOONKA DK | 1 | 0.033 |
| VEMUGANTI R | 3 | 0.1 | MOON A | 1 | 0.033 |
| VAUSORT M | 3 | 0.1 | MONTOYA NR | 1 | 0.033 |
| VAN OUDENAARDEN A | 3 | 0.1 | MONTORSI F | 1 | 0.033 |
| UCHIDA S | 3 | 0.1 | MONTONE KT | 1 | 0.033 |
| TU JC | 3 | 0.1 | MONTIEL J | 1 | 0.033 |
| TU J | 3 | 0.1 | MONTGOMERY KD | 1 | 0.033 |
| TSUKIYAMA T | 3 | 0.1 | MONTGOMERY K | 1 | 0.033 |
| TSAI MC | 3 | 0.1 | MONTEIRO ANA | 1 | 0.033 |
| TORIMURA M | 3 | 0.1 | MONTAVON T | 1 | 0.033 |
| TIE Y | 3 | 0.1 | MONTASSER IF | 1 | 0.033 |
| TIAN XY | 3 | 0.1 | MONSIOR J | 1 | 0.033 |
| TIAN XL | 3 | 0.1 | MONROIG P | 1 | 0.033 |
| TIAN LL | 3 | 0.1 | MONKS B | 1 | 0.033 |
| TIAN F | 3 | 0.1 | MONIA BP | 1 | 0.033 |
| THOMPSON CL | 3 | 0.1 | MONASOR LS | 1 | 0.033 |
| TAO H | 3 | 0.1 | MONACK DM | 1 | 0.033 |
| TANYI JL | 3 | 0.1 | MOMOZAWA Y | 1 | 0.033 |
| TANG Y | 3 | 0.1 | MOLLOY K | 1 | 0.033 |
| TANG K | 3 | 0.1 | MOLLER P | 1 | 0.033 |
| TANG JY | 3 | 0.1 | MOLLER M | 1 | 0.033 |
| TAN L | 3 | 0.1 | MOLINS L | 1 | 0.033 |
| TAKAHASHI K | 3 | 0.1 | MOLINIE B | 1 | 0.033 |
| SZCZESNIAK MW | 3 | 0.1 | MOISES J | 1 | 0.033 |
| SVOBODA M | 3 | 0.1 | MOHRI Y | 1 | 0.033 |
| SUZUKI Y | 3 | 0.1 | MOHR M | 1 | 0.033 |
| SUZUKI H | 3 | 0.1 | MOHANTY V | 1 | 0.033 |
| SUNG S | 3 | 0.1 | MOHANKUMAR S | 1 | 0.033 |
| SUN YW | 3 | 0.1 | MOHANDAS N | 1 | 0.033 |
| SUN YJ | 3 | 0.1 | MOHAN KN | 1 | 0.033 |
| SUN YF | 3 | 0.1 | MOHAMMED S | 1 | 0.033 |
| SUN XL | 3 | 0.1 | MOHAMMED A | 1 | 0.033 |
| SUN XJ | 3 | 0.1 | MOHAMMADIN S | 1 | 0.033 |
| SUN XC | 3 | 0.1 | MOHAMMADI P | 1 | 0.033 |
| SUN QQ | 3 | 0.1 | MOHAMED JS | 1 | 0.033 |
| SUN JB | 3 | 0.1 | MOHAMADKHANI A | 1 | 0.033 |
| SUN CC | 3 | 0.1 | MOERTL S | 1 | 0.033 |
| SUN BC | 3 | 0.1 | MODISE T | 1 | 0.033 |
| SUI WG | 3 | 0.1 | MODESITT SC | 1 | 0.033 |
| SUI J | 3 | 0.1 | MODDERMAN R | 1 | 0.033 |
| SUGIMACHI K | 3 | 0.1 | MODARRESI F | 1 | 0.033 |
| SUBHASH S | 3 | 0.1 | MODALI SD | 1 | 0.033 |
| SU L | 3 | 0.1 | MOAD CA | 1 | 0.033 |
| SU HF | 3 | 0.1 | MO XY | 1 | 0.033 |
| SU F | 3 | 0.1 | MO WJ | 1 | 0.033 |
| STANDAERT L | 3 | 0.1 | MO M | 1 | 0.033 |
| ST LAURENT G | 3 | 0.1 | MO KL | 1 | 0.033 |
| SOOD AK | 3 | 0.1 | MO JM | 1 | 0.033 |
| SONG YP | 3 | 0.1 | MO JD | 1 | 0.033 |
| SONG YC | 3 | 0.1 | MO F | 1 | 0.033 |
| SONG CH | 3 | 0.1 | MO BX | 1 | 0.033 |
| SONG C | 3 | 0.1 | MLCOCHOVA J | 1 | 0.033 |
| SIVADAS A | 3 | 0.1 | MKANNEZ G | 1 | 0.033 |
| SIMON MD | 3 | 0.1 | MIZUTANI S | 1 | 0.033 |
| SILVA WA | 3 | 0.1 | MIZUTANI R | 1 | 0.033 |
| SILVA JP | 3 | 0.1 | MIZUKAMI T | 1 | 0.033 |
| SIDDIQUI J | 3 | 0.1 | MIZOKAMI M | 1 | 0.033 |
| SHU Y | 3 | 0.1 | MIZOGUCHI T | 1 | 0.033 |
| SHI XJ | 3 | 0.1 | MIYOSHI H | 1 | 0.033 |
| SHENG WQ | 3 | 0.1 | MIYAZONO K | 1 | 0.033 |
| SHEN YY | 3 | 0.1 | MIYAUCHI E | 1 | 0.033 |
| SHEN YJ | 3 | 0.1 | MIYATA K | 1 | 0.033 |
| SHEN WF | 3 | 0.1 | MIYASHITA A | 1 | 0.033 |
| SHELLEY J | 3 | 0.1 | MIYANO S | 1 | 0.033 |
| SHARP PA | 3 | 0.1 | MIYAGAWA R | 1 | 0.033 |
| SHAO PF | 3 | 0.1 | MIURA S | 1 | 0.033 |
| SHANG DS | 3 | 0.1 | MITTAL N | 1 | 0.033 |
| SHAN G | 3 | 0.1 | MITRA R | 1 | 0.033 |
| SHAN B | 3 | 0.1 | MITO M | 1 | 0.033 |
| SAUVAGEAU M | 3 | 0.1 | MITCHELL SM | 1 | 0.033 |
| SANGES R | 3 | 0.1 | MITCHELL CJ | 1 | 0.033 |
| RONCHETTI D | 3 | 0.1 | MISKIMEN KLS | 1 | 0.033 |
| RINN J | 3 | 0.1 | MISHRA T | 1 | 0.033 |
| RIGO F | 3 | 0.1 | MISHRA RK | 1 | 0.033 |
| RENNERT OM | 3 | 0.1 | MISHRA K | 1 | 0.033 |
| REN Y | 3 | 0.1 | MISAWA A | 1 | 0.033 |
| REIK W | 3 | 0.1 | MIRSAFIAN H | 1 | 0.033 |
| RAPICAVOLI NA | 3 | 0.1 | MIRFAKHRAIE R | 1 | 0.033 |
| RAMOS AD | 3 | 0.1 | MIRANDA J | 1 | 0.033 |
| RADAELLI E | 3 | 0.1 | MIRALDI ER | 1 | 0.033 |
| QURESHI IA | 3 | 0.1 | MIRABELLA M | 1 | 0.033 |
| QUAN MY | 3 | 0.1 | MIOTTO E | 1 | 0.033 |
| QU LM | 3 | 0.1 | MING-DONG W | 1 | 0.033 |
| QIU YR | 3 | 0.1 | MING ZJ | 1 | 0.033 |
| QIU SL | 3 | 0.1 | MING X | 1 | 0.033 |
| QIU M | 3 | 0.1 | MING H | 1 | 0.033 |
| QIN XY | 3 | 0.1 | MING GF | 1 | 0.033 |
| QIN XJ | 3 | 0.1 | MINEO M | 1 | 0.033 |
| QIAO YX | 3 | 0.1 | MIN WJ | 1 | 0.033 |
| QI J | 3 | 0.1 | MIN W | 1 | 0.033 |
| QI FZ | 3 | 0.1 | MIN J | 1 | 0.033 |
| PU PY | 3 | 0.1 | MIN F | 1 | 0.033 |
| PU JKS | 3 | 0.1 | MILOSCIO L | 1 | 0.033 |
| POTH EM | 3 | 0.1 | MILOSAVLJEVIC A | 1 | 0.033 |
| PESCHANSKY VJ | 3 | 0.1 | MILLWARD NMZ | 1 | 0.033 |
| PENG SP | 3 | 0.1 | MILLS TC | 1 | 0.033 |
| PENG G | 3 | 0.1 | MILLS NL | 1 | 0.033 |
| PENG CH | 3 | 0.1 | MILLIGAN MJ | 1 | 0.033 |
| PENG C | 3 | 0.1 | MILLER JS | 1 | 0.033 |
| PATEL V | 3 | 0.1 | MILITTI C | 1 | 0.033 |
| PAROLIA A | 3 | 0.1 | MILINKOVITCH M | 1 | 0.033 |
| PARALKAR VR | 3 | 0.1 | MILEVSKIY MJG | 1 | 0.033 |
| PANG LX | 3 | 0.1 | MILAZZO G | 1 | 0.033 |
| PANG KC | 3 | 0.1 | MIKULA-PIETRASIK J | 1 | 0.033 |
| PANG H | 3 | 0.1 | MIKKERS HMM | 1 | 0.033 |
| PAN ZW | 3 | 0.1 | MIKKERS H | 1 | 0.033 |
| PAN XY | 3 | 0.1 | MIKI A | 1 | 0.033 |
| PAN W | 3 | 0.1 | MIKHAIL M | 1 | 0.033 |
| PAN QS | 3 | 0.1 | MIERSCH H | 1 | 0.033 |
| OUYANG XJ | 3 | 0.1 | MIELKE JA | 1 | 0.033 |
| OUYANG J | 3 | 0.1 | MICURA R | 1 | 0.033 |
| OHHATA T | 3 | 0.1 | MICHLEWSKI G | 1 | 0.033 |
| NOWAKOWSKI TJ | 3 | 0.1 | MICHELHAUGH SK | 1 | 0.033 |
| NOTREDAME C | 3 | 0.1 | MICHEL OJ | 1 | 0.033 |
| NOH JH | 3 | 0.1 | MICHALSKI CW | 1 | 0.033 |
| NING QL | 3 | 0.1 | MICHALIK KM | 1 | 0.033 |
| NIKNAFS YS | 3 | 0.1 | MICHAELIDOU K | 1 | 0.033 |
| NIIDA H | 3 | 0.1 | MICHAELI S | 1 | 0.033 |
| NIE YZ | 3 | 0.1 | MIAO ZJ | 1 | 0.033 |
| NIAZI F | 3 | 0.1 | MIAO YF | 1 | 0.033 |
| NI SJ | 3 | 0.1 | MIAO XP | 1 | 0.033 |
| NAVARRO A | 3 | 0.1 | MIAO M | 1 | 0.033 |
| NAEMURA M | 3 | 0.1 | MIAO LY | 1 | 0.033 |
| MULLEN AC | 3 | 0.1 | MIAO L | 1 | 0.033 |
| MOWLA SJ | 3 | 0.1 | MIANO V | 1 | 0.033 |
| MONZO M | 3 | 0.1 | MI RR | 1 | 0.033 |
| MILLS GB | 3 | 0.1 | MEYER N | 1 | 0.033 |
| MENG XM | 3 | 0.1 | METSPALU A | 1 | 0.033 |
| MEN Y | 3 | 0.1 | METRO G | 1 | 0.033 |
| MATHUR R | 3 | 0.1 | MESSERE A | 1 | 0.033 |
| MATBOLI M | 3 | 0.1 | MESSER K | 1 | 0.033 |
| MASOTTI A | 3 | 0.1 | MESSENGER ZJ | 1 | 0.033 |
| MARUYAMA R | 3 | 0.1 | MESSEMAKER TC | 1 | 0.033 |
| MARIN-BEJAR O | 3 | 0.1 | MESSEGUER X | 1 | 0.033 |
| MAQUAT LE | 3 | 0.1 | MESMAR F | 1 | 0.033 |
| MAO YQ | 3 | 0.1 | MESEURE D | 1 | 0.033 |
| MAO WM | 3 | 0.1 | MERYET-FIGUIERE M | 1 | 0.033 |
| MAO JH | 3 | 0.1 | MERT U | 1 | 0.033 |
| MANZONI M | 3 | 0.1 | MERLE P | 1 | 0.033 |
| MAKALOWSKA I | 3 | 0.1 | MERKURJEV D | 1 | 0.033 |
| MACAULAY CE | 3 | 0.1 | MERKENSCHLAGER M | 1 | 0.033 |
| MAAG JLV | 3 | 0.1 | MERKEL A | 1 | 0.033 |
| MA XL | 3 | 0.1 | MERICAN AF | 1 | 0.033 |
| MA XF | 3 | 0.1 | MERIC-BERNSTAM F | 1 | 0.033 |
| MA WY | 3 | 0.1 | MERELO V | 1 | 0.033 |
| MA WL | 3 | 0.1 | MEREDITH EK | 1 | 0.033 |
| MA W | 3 | 0.1 | MEREAU A | 1 | 0.033 |
| LV Z | 3 | 0.1 | MERAL R | 1 | 0.033 |
| LV YB | 3 | 0.1 | MEOLA N | 1 | 0.033 |
| LV XB | 3 | 0.1 | MENSENKAMP AR | 1 | 0.033 |
| LV SX | 3 | 0.1 | MENSCHAERT G | 1 | 0.033 |
| LV P | 3 | 0.1 | MENON S | 1 | 0.033 |
| LUO YB | 3 | 0.1 | MENKO FH | 1 | 0.033 |
| LUO Y | 3 | 0.1 | MENICANTI L | 1 | 0.033 |
| LUO XC | 3 | 0.1 | MENG YS | 1 | 0.033 |
| LUO DZ | 3 | 0.1 | MENG YJ | 1 | 0.033 |
| LUND AH | 3 | 0.1 | MENG YC | 1 | 0.033 |
| LUI WM | 3 | 0.1 | MENG Y | 1 | 0.033 |
| LU Z | 3 | 0.1 | MENG XX | 1 | 0.033 |
| LU YH | 3 | 0.1 | MENG XW | 1 | 0.033 |
| LU Y | 3 | 0.1 | MENG XH | 1 | 0.033 |
| LU XY | 3 | 0.1 | MENG T | 1 | 0.033 |
| LU JC | 3 | 0.1 | MENG SY | 1 | 0.033 |
| LU BB | 3 | 0.1 | MENG QY | 1 | 0.033 |
| LOPEZ-PAJARES V | 3 | 0.1 | MENG QJ | 1 | 0.033 |
| LONG B | 3 | 0.1 | MENG Q | 1 | 0.033 |
| LIYANARACHCHI S | 3 | 0.1 | MENG N | 1 | 0.033 |
| LIU YK | 3 | 0.1 | MENG MJ | 1 | 0.033 |
| LIU YF | 3 | 0.1 | MENG M | 1 | 0.033 |
| LIU XY | 3 | 0.1 | MENG LZ | 1 | 0.033 |
| LIU XW | 3 | 0.1 | MENG LS | 1 | 0.033 |
| LIU XM | 3 | 0.1 | MENG JG | 1 | 0.033 |
| LIU XC | 3 | 0.1 | MENG HY | 1 | 0.033 |
| LIU XB | 3 | 0.1 | MENG HX | 1 | 0.033 |
| LIU WT | 3 | 0.1 | MENG FQ | 1 | 0.033 |
| LIU WJ | 3 | 0.1 | MENG D | 1 | 0.033 |
| LIU SJ | 3 | 0.1 | MENCK CFM | 1 | 0.033 |
| LIU QY | 3 | 0.1 | MENCARONI C | 1 | 0.033 |
| LIU MR | 3 | 0.1 | MELO SA | 1 | 0.033 |
| LIU ML | 3 | 0.1 | MELO MB | 1 | 0.033 |
| LIU LX | 3 | 0.1 | MELO CA | 1 | 0.033 |
| LIU LL | 3 | 0.1 | MELNICK A | 1 | 0.033 |
| LIU HY | 3 | 0.1 | MELL B | 1 | 0.033 |
| LIU HT | 3 | 0.1 | MELISSARI MT | 1 | 0.033 |
| LIU HQ | 3 | 0.1 | MELE M | 1 | 0.033 |
| LIU HJ | 3 | 0.1 | MELDRUM AMR | 1 | 0.033 |
| LIU HH | 3 | 0.1 | MEISTER M | 1 | 0.033 |
| LIU DJ | 3 | 0.1 | MEISTER G | 1 | 0.033 |
| LIU CX | 3 | 0.1 | MEISSNER A | 1 | 0.033 |
| LIU CM | 3 | 0.1 | MEIERHOFER D | 1 | 0.033 |
| LIU CL | 3 | 0.1 | MEIER M | 1 | 0.033 |
| LINDSAY MA | 3 | 0.1 | MEI QY | 1 | 0.033 |
| LIN XJ | 3 | 0.1 | MEI M | 1 | 0.033 |
| LIN MY | 3 | 0.1 | MEI JZ | 1 | 0.033 |
| LIN M | 3 | 0.1 | MEI J | 1 | 0.033 |
| LIN J | 3 | 0.1 | MEHTA SL | 1 | 0.033 |
| LIN D | 3 | 0.1 | MEHTA A | 1 | 0.033 |
| LIN CS | 3 | 0.1 | MEGHA S | 1 | 0.033 |
| LIN CR | 3 | 0.1 | MEERSSEMAN C | 1 | 0.033 |
| LIAO QJ | 3 | 0.1 | MEDVEDEVA YA | 1 | 0.033 |
| LIANG YC | 3 | 0.1 | MEDVEDEV AE | 1 | 0.033 |
| LIANG T | 3 | 0.1 | MEDORO L | 1 | 0.033 |
| LIANG L | 3 | 0.1 | MEDA P | 1 | 0.033 |
| LIANG GY | 3 | 0.1 | MEADOWS S | 1 | 0.033 |
| LI ZW | 3 | 0.1 | MCRAE EKS | 1 | 0.033 |
| LI ZK | 3 | 0.1 | MCMULLEN JR | 1 | 0.033 |
| LI ZJ | 3 | 0.1 | MCLAUGHLIN J | 1 | 0.033 |
| LI YW | 3 | 0.1 | MCKINNON B | 1 | 0.033 |
| LI XQ | 3 | 0.1 | MCKIERNAN PJ | 1 | 0.033 |
| LI XK | 3 | 0.1 | MCKENNA SA | 1 | 0.033 |
| LI XH | 3 | 0.1 | MCKAY J | 1 | 0.033 |
| LI XB | 3 | 0.1 | MCINTYRE L | 1 | 0.033 |
| LI WZ | 3 | 0.1 | MCHUGH CA | 1 | 0.033 |
| LI WQ | 3 | 0.1 | MCGUIRE M | 1 | 0.033 |
| LI WM | 3 | 0.1 | MCGREGOR N | 1 | 0.033 |
| LI WJ | 3 | 0.1 | MCGOWAN JD | 1 | 0.033 |
| LI SF | 3 | 0.1 | MCGINNIS KM | 1 | 0.033 |
| LI QL | 3 | 0.1 | MCFADDEN EJ | 1 | 0.033 |
| LI PL | 3 | 0.1 | MCEVOY A | 1 | 0.033 |
| LI MY | 3 | 0.1 | MCELVANEY NG | 1 | 0.033 |
| LI MM | 3 | 0.1 | MCDONEL PE | 1 | 0.033 |
| LI JP | 3 | 0.1 | MCDANIEL L | 1 | 0.033 |
| LI JG | 3 | 0.1 | MCCUE B | 1 | 0.033 |
| LI HW | 3 | 0.1 | MCCRIGHT SJ | 1 | 0.033 |
| LI GP | 3 | 0.1 | MCCLURE J | 1 | 0.033 |
| LI GF | 3 | 0.1 | MCCAUGHAN B | 1 | 0.033 |
| LI DS | 3 | 0.1 | MCCARTY G | 1 | 0.033 |
| LI CW | 3 | 0.1 | MCCARTHY R | 1 | 0.033 |
| LI CL | 3 | 0.1 | MCBRIDE MW | 1 | 0.033 |
| LI CJ | 3 | 0.1 | MCBRIDE M | 1 | 0.033 |
| LEUNG EY | 3 | 0.1 | MCALLISTER BF | 1 | 0.033 |
| LEUNG A | 3 | 0.1 | MAZA I | 1 | 0.033 |
| LEGNINI I | 3 | 0.1 | MAYSTADT I | 1 | 0.033 |
| LEE SK | 3 | 0.1 | MAYNOR T | 1 | 0.033 |
| LEE H | 3 | 0.1 | MAYEDA A | 1 | 0.033 |
| LEE CS | 3 | 0.1 | MAVRIDIS K | 1 | 0.033 |
| LEE B | 3 | 0.1 | MAUSSION G | 1 | 0.033 |
| LARSSON E | 3 | 0.1 | MAURY F | 1 | 0.033 |
| LARSEN MJ | 3 | 0.1 | MATTER MS | 1 | 0.033 |
| LANTING L | 3 | 0.1 | MATSUURA M | 1 | 0.033 |
| LAN XB | 3 | 0.1 | MATSUURA K | 1 | 0.033 |
| KUROCHKIN IV | 3 | 0.1 | MATSUMURA K | 1 | 0.033 |
| KURASHIGE J | 3 | 0.1 | MATSUMOTO Y | 1 | 0.033 |
| KUNG HJ | 3 | 0.1 | MATSUDA K | 1 | 0.033 |
| KUMARSWAMY R | 3 | 0.1 | MATSUDA A | 1 | 0.033 |
| KRIEGSTEIN AR | 3 | 0.1 | MATSUBARA S | 1 | 0.033 |
| KRAUS WL | 3 | 0.1 | MATKOVICH SJ | 1 | 0.033 |
| KORNIENKO AE | 3 | 0.1 | MATKAR PN | 1 | 0.033 |
| KONG X | 3 | 0.1 | MATISIC JP | 1 | 0.033 |
| KONG LS | 3 | 0.1 | MATIS S | 1 | 0.033 |
| KOHTZ JD | 3 | 0.1 | MATHIEU P | 1 | 0.033 |
| KINGSTON RE | 3 | 0.1 | MATHEW CG | 1 | 0.033 |
| KIMURA AP | 3 | 0.1 | MATHEU A | 1 | 0.033 |
| KIM YT | 3 | 0.1 | MATAK-VINKOVIC D | 1 | 0.033 |
| KIM YJ | 3 | 0.1 | MASUTA C | 1 | 0.033 |
| KIM Y | 3 | 0.1 | MASUGUCHI S | 1 | 0.033 |
| KIM TK | 3 | 0.1 | MASSIMELLI MJ | 1 | 0.033 |
| KIM SW | 3 | 0.1 | MASSALINI S | 1 | 0.033 |
| KIM M | 3 | 0.1 | MASSALHA H | 1 | 0.033 |
| KIANG KMY | 3 | 0.1 | MASKALI F | 1 | 0.033 |
| KAUSHIK K | 3 | 0.1 | MASHIMA T | 1 | 0.033 |
| KAUPPINEN S | 3 | 0.1 | MASETTI R | 1 | 0.033 |
| KANG JH | 3 | 0.1 | MASCIARELLI S | 1 | 0.033 |
| KANG J | 3 | 0.1 | MASAKI J | 1 | 0.033 |
| KANG CS | 3 | 0.1 | MARTINSSON T | 1 | 0.033 |
| JU WN | 3 | 0.1 | MARTINO F | 1 | 0.033 |
| JOHNSSON P | 3 | 0.1 | MARTINEZ-ZAMUDIO RI | 1 | 0.033 |
| JING XJ | 3 | 0.1 | MARTINEZ-INIESTA M | 1 | 0.033 |
| JING W | 3 | 0.1 | MARTINEZ-HERRERA DJ | 1 | 0.033 |
| JIN YH | 3 | 0.1 | MARTINEZ-GUITARTE JL | 1 | 0.033 |
| JIN X | 3 | 0.1 | MARTINEZ-FERNANDEZ M | 1 | 0.033 |
| JIN SL | 3 | 0.1 | MARTINEZ-CARDUS A | 1 | 0.033 |
| JIN FY | 3 | 0.1 | MARTINEZ-CALVILLO S | 1 | 0.033 |
| JIN CY | 3 | 0.1 | MARTINEZ O | 1 | 0.033 |
| JIAO Y | 3 | 0.1 | MARTINET C | 1 | 0.033 |
| JIANG YZ | 3 | 0.1 | MARTIN-BULEY LA | 1 | 0.033 |
| JIANG T | 3 | 0.1 | MARTIN RD | 1 | 0.033 |
| JIANG M | 3 | 0.1 | MARTIN M | 1 | 0.033 |
| JIANG LX | 3 | 0.1 | MARTIN D | 1 | 0.033 |
| JIANG GS | 3 | 0.1 | MARTI-RENOM MA | 1 | 0.033 |
| JIANG CJ | 3 | 0.1 | MARTENS-UZUNOVA ES | 1 | 0.033 |
| JIA Y | 3 | 0.1 | MARTENS JWM | 1 | 0.033 |
| JIA XM | 3 | 0.1 | MARTELOSSI S | 1 | 0.033 |
| JIA RB | 3 | 0.1 | MARSICO A | 1 | 0.033 |
| JI WD | 3 | 0.1 | MARRADES RM | 1 | 0.033 |
| JEON YJ | 3 | 0.1 | MARRA L | 1 | 0.033 |
| IVAN C | 3 | 0.1 | MARRA A | 1 | 0.033 |
| ITO Y | 3 | 0.1 | MARQUES RB | 1 | 0.033 |
| HUO YM | 3 | 0.1 | MARKUS HS | 1 | 0.033 |
| HUO XS | 3 | 0.1 | MARKS JR | 1 | 0.033 |
| HUANG ZK | 3 | 0.1 | MARKOWITZ SD | 1 | 0.033 |
| HUANG YP | 3 | 0.1 | MARIS JM | 1 | 0.033 |
| HUANG XX | 3 | 0.1 | MARINOV GK | 1 | 0.033 |
| HUANG XR | 3 | 0.1 | MARINELLI L | 1 | 0.033 |
| HUANG X | 3 | 0.1 | MARIE PY | 1 | 0.033 |
| HUANG T | 3 | 0.1 | MARGUERAT S | 1 | 0.033 |
| HUANG SY | 3 | 0.1 | MARDAN M | 1 | 0.033 |
| HUANG LL | 3 | 0.1 | MARCUCCI G | 1 | 0.033 |
| HUANG JT | 3 | 0.1 | MARCHO C | 1 | 0.033 |
| HUANG JM | 3 | 0.1 | MARCHIONI M | 1 | 0.033 |
| HUANG JL | 3 | 0.1 | MARCHIO S | 1 | 0.033 |
| HUANG GL | 3 | 0.1 | MARCHIO A | 1 | 0.033 |
| HUANG GJ | 3 | 0.1 | MARCHAND JT | 1 | 0.033 |
| HUA R | 3 | 0.1 | MARAGKAKIS M | 1 | 0.033 |
| HUA L | 3 | 0.1 | MARACAJA-COUTINHO V | 1 | 0.033 |
| HU XY | 3 | 0.1 | MAQBOOL S | 1 | 0.033 |
| HU XW | 3 | 0.1 | MAO ZT | 1 | 0.033 |
| HU XS | 3 | 0.1 | MAO YS | 1 | 0.033 |
| HU XQ | 3 | 0.1 | MAO YM | 1 | 0.033 |
| HU SS | 3 | 0.1 | MAO YJ | 1 | 0.033 |
| HU SF | 3 | 0.1 | MAO XY | 1 | 0.033 |
| HU Q | 3 | 0.1 | MAO XP | 1 | 0.033 |
| HU JJ | 3 | 0.1 | MAO XM | 1 | 0.033 |
| HU JH | 3 | 0.1 | MAO XH | 1 | 0.033 |
| HU H | 3 | 0.1 | MAO WD | 1 | 0.033 |
| HU G | 3 | 0.1 | MAO QS | 1 | 0.033 |
| HU CJ | 3 | 0.1 | MAO Q | 1 | 0.033 |
| HU B | 3 | 0.1 | MAO LP | 1 | 0.033 |
| HRDLICKOVA B | 3 | 0.1 | MAO L | 1 | 0.033 |
| HIRATA H | 3 | 0.1 | MAO JQ | 1 | 0.033 |
| HERRMANN BG | 3 | 0.1 | MAO JF | 1 | 0.033 |
| HE X | 3 | 0.1 | MAO HG | 1 | 0.033 |
| HE W | 3 | 0.1 | MAO CX | 1 | 0.033 |
| HE Q | 3 | 0.1 | MAO CP | 1 | 0.033 |
| HE K | 3 | 0.1 | MAO CM | 1 | 0.033 |
| HE HL | 3 | 0.1 | MAO CC | 1 | 0.033 |
| HE HJ | 3 | 0.1 | MAO C | 1 | 0.033 |
| HE BS | 3 | 0.1 | MAO AQ | 1 | 0.033 |
| HAO JW | 3 | 0.1 | MAO AP | 1 | 0.033 |
| HANSJI H | 3 | 0.1 | MANTOVANI-LOFFLER L | 1 | 0.033 |
| HAN ZJ | 3 | 0.1 | MANTELLA LE | 1 | 0.033 |
| HAN YH | 3 | 0.1 | MANOS PD | 1 | 0.033 |
| HAN T | 3 | 0.1 | MANOR O | 1 | 0.033 |
| HAN SC | 3 | 0.1 | MANNO M | 1 | 0.033 |
| HAN JW | 3 | 0.1 | MANGIAVACCHI A | 1 | 0.033 |
| HAN JQ | 3 | 0.1 | MANDELL J | 1 | 0.033 |
| HAN B | 3 | 0.1 | MANDAL P | 1 | 0.033 |
| HAMMERLE M | 3 | 0.1 | MANDA SS | 1 | 0.033 |
| HAMADA M | 3 | 0.1 | MANCONE C | 1 | 0.033 |
| HAGA H | 3 | 0.1 | MANCIO-SILVA L | 1 | 0.033 |
| HAERTY W | 3 | 0.1 | MANAVSKI Y | 1 | 0.033 |
| HACISULEYMAN E | 3 | 0.1 | MANABE S | 1 | 0.033 |
| GUPTA R | 3 | 0.1 | MAN VOY | 1 | 0.033 |
| GUO Z | 3 | 0.1 | MALTAIS S | 1 | 0.033 |
| GUO YH | 3 | 0.1 | MALQUORI L | 1 | 0.033 |
| GUO XL | 3 | 0.1 | MALLORY AC | 1 | 0.033 |
| GUO X | 3 | 0.1 | MALLARD W | 1 | 0.033 |
| GUO M | 3 | 0.1 | MALIK B | 1 | 0.033 |
| GUO F | 3 | 0.1 | MALIH S | 1 | 0.033 |
| GUO CY | 3 | 0.1 | MALIH N | 1 | 0.033 |
| GUO C | 3 | 0.1 | MALCOLM G | 1 | 0.033 |
| GUI YT | 3 | 0.1 | MALAKAR P | 1 | 0.033 |
| GUENZL PM | 3 | 0.1 | MAKOWSKA Z | 1 | 0.033 |
| GU HY | 3 | 0.1 | MAKOVICKY P | 1 | 0.033 |
| GU DY | 3 | 0.1 | MAKIYA MA | 1 | 0.033 |
| GROFF AF | 3 | 0.1 | MAKHMUTOVA M | 1 | 0.033 |
| GRIMMOND SM | 3 | 0.1 | MAJIDINIA M | 1 | 0.033 |
| GRANDER D | 3 | 0.1 | MAJID S | 1 | 0.033 |
| GONZALEZ J | 3 | 0.1 | MAJI S | 1 | 0.033 |
| GONG C | 3 | 0.1 | MAJI RK | 1 | 0.033 |
| GOMEZ A | 3 | 0.1 | MAJEWSKI T | 1 | 0.033 |
| GLOSS BS | 3 | 0.1 | MAJEWSKI J | 1 | 0.033 |
| GINSBERG D | 3 | 0.1 | MAJERCIAK V | 1 | 0.033 |
| GERNAPUDI R | 3 | 0.1 | MAIMONE B | 1 | 0.033 |
| GERGER A | 3 | 0.1 | MAIMAITI R | 1 | 0.033 |
| GE J | 3 | 0.1 | MAIMAITI A | 1 | 0.033 |
| GAO Q | 3 | 0.1 | MAIA AR | 1 | 0.033 |
| GAO L | 3 | 0.1 | MAI A | 1 | 0.033 |
| GAO HY | 3 | 0.1 | MAHURKAR-JOSHI S | 1 | 0.033 |
| GAN BY | 3 | 0.1 | MAH A | 1 | 0.033 |
| GAETANO C | 3 | 0.1 | MAGLIERI G | 1 | 0.033 |
| GABALDON T | 3 | 0.1 | MAGAOWEIYA S | 1 | 0.033 |
| FU ZQ | 3 | 0.1 | MAETZIG T | 1 | 0.033 |
| FU XB | 3 | 0.1 | MAEHARA Y | 1 | 0.033 |
| FINLAY GJ | 3 | 0.1 | MAEGAWA S | 1 | 0.033 |
| FENG ZB | 3 | 0.1 | MADAR S | 1 | 0.033 |
| FENG X | 3 | 0.1 | MACLEOD AR | 1 | 0.033 |
| FENG C | 3 | 0.1 | MACKLIN JA | 1 | 0.033 |
| FENG B | 3 | 0.1 | MACKENZIE S | 1 | 0.033 |
| FEI ZH | 3 | 0.1 | MACKENZIE R | 1 | 0.033 |
| FEI T | 3 | 0.1 | MACINO G | 1 | 0.033 |
| FANG J | 3 | 0.1 | MACHARIA AW | 1 | 0.033 |
| FANG GE | 3 | 0.1 | MACHADO CA | 1 | 0.033 |
| FAN YR | 3 | 0.1 | MACDONALD TY | 1 | 0.033 |
| FAN LL | 3 | 0.1 | MACDONALD ME | 1 | 0.033 |
| EVANS JR | 3 | 0.1 | MACAULAY A | 1 | 0.033 |
| ENGREITZ JM | 3 | 0.1 | MAASS PG | 1 | 0.033 |
| ENGEL N | 3 | 0.1 | MA ZS | 1 | 0.033 |
| ENFIELD KSS | 3 | 0.1 | MA ZK | 1 | 0.033 |
| DUN SZ | 3 | 0.1 | MA ZH | 1 | 0.033 |
| DUAN YL | 3 | 0.1 | MA ZG | 1 | 0.033 |
| DUAN Y | 3 | 0.1 | MA YT | 1 | 0.033 |
| DUAN SW | 3 | 0.1 | MA YN | 1 | 0.033 |
| DU YW | 3 | 0.1 | MA YD | 1 | 0.033 |
| DU W | 3 | 0.1 | MA YC | 1 | 0.033 |
| DU J | 3 | 0.1 | MA Y | 1 | 0.033 |
| DOU J | 3 | 0.1 | MA XX | 1 | 0.033 |
| DONG ZM | 3 | 0.1 | MA XS | 1 | 0.033 |
| DONG WW | 3 | 0.1 | MA XK | 1 | 0.033 |
| DONG R | 3 | 0.1 | MA XE | 1 | 0.033 |
| DONG L | 3 | 0.1 | MA XD | 1 | 0.033 |
| DONG J | 3 | 0.1 | MA XC | 1 | 0.033 |
| DONG GC | 3 | 0.1 | MA TT | 1 | 0.033 |
| DING XF | 3 | 0.1 | MA TL | 1 | 0.033 |
| DING F | 3 | 0.1 | MA SY | 1 | 0.033 |
| DIMMELER S | 3 | 0.1 | MA SM | 1 | 0.033 |
| DI C | 3 | 0.1 | MA SJ | 1 | 0.033 |
| DHARAP A | 3 | 0.1 | MA SC | 1 | 0.033 |
| DENG XX | 3 | 0.1 | MA S | 1 | 0.033 |
| DENG KY | 3 | 0.1 | MA RX | 1 | 0.033 |
| DENG JQ | 3 | 0.1 | MA QG | 1 | 0.033 |
| DENG J | 3 | 0.1 | MA Q | 1 | 0.033 |
| DENG H | 3 | 0.1 | MA PH | 1 | 0.033 |
| DE LA CHAPELLE A | 3 | 0.1 | MA ML | 1 | 0.033 |
| DANG Y | 3 | 0.1 | MA M | 1 | 0.033 |
| DAMMERT MA | 3 | 0.1 | MA LT | 1 | 0.033 |
| DAI JB | 3 | 0.1 | MA LM | 1 | 0.033 |
| DAI B | 3 | 0.1 | MA LJ | 1 | 0.033 |
| CUI ZL | 3 | 0.1 | MA LB | 1 | 0.033 |
| CUI R | 3 | 0.1 | MA KX | 1 | 0.033 |
| CUI L | 3 | 0.1 | MA KF | 1 | 0.033 |
| CUI JW | 3 | 0.1 | MA JY | 1 | 0.033 |
| CUI D | 3 | 0.1 | MA JX | 1 | 0.033 |
| CROOKE PS | 3 | 0.1 | MA JT | 1 | 0.033 |
| CREA F | 3 | 0.1 | MA HZ | 1 | 0.033 |
| COLOMBO T | 3 | 0.1 | MA HW | 1 | 0.033 |
| COLLINS C | 3 | 0.1 | MA HD | 1 | 0.033 |
| COARFA C | 3 | 0.1 | MA H | 1 | 0.033 |
| CHUA NH | 3 | 0.1 | MA GT | 1 | 0.033 |
| CHENG Y | 3 | 0.1 | MA GL | 1 | 0.033 |
| CHENG XL | 3 | 0.1 | MA DJ | 1 | 0.033 |
| CHENG NN | 3 | 0.1 | MA DH | 1 | 0.033 |
| CHEN ZZ | 3 | 0.1 | MA DD | 1 | 0.033 |
| CHEN ZY | 3 | 0.1 | MA D | 1 | 0.033 |
| CHEN YM | 3 | 0.1 | MA CX | 1 | 0.033 |
| CHEN YH | 3 | 0.1 | MA CJ | 1 | 0.033 |
| CHEN YF | 3 | 0.1 | MA CG | 1 | 0.033 |
| CHEN YC | 3 | 0.1 | MA CF | 1 | 0.033 |
| CHEN XX | 3 | 0.1 | MA BB | 1 | 0.033 |
| CHEN XH | 3 | 0.1 | LYU XJ | 1 | 0.033 |
| CHEN XD | 3 | 0.1 | LYU SX | 1 | 0.033 |
| CHEN WJ | 3 | 0.1 | LYU J | 1 | 0.033 |
| CHEN RP | 3 | 0.1 | LYU H | 1 | 0.033 |
| CHEN K | 3 | 0.1 | LYONS SM | 1 | 0.033 |
| CHEN JG | 3 | 0.1 | LYONS E | 1 | 0.033 |
| CHEN JC | 3 | 0.1 | LYNCH VJ | 1 | 0.033 |
| CHEN HM | 3 | 0.1 | LYNCH TH | 1 | 0.033 |
| CHEN GJ | 3 | 0.1 | LV ZW | 1 | 0.033 |
| CHEN FJ | 3 | 0.1 | LV ZQ | 1 | 0.033 |
| CHEN D | 3 | 0.1 | LV YL | 1 | 0.033 |
| CHEN BL | 3 | 0.1 | LV YD | 1 | 0.033 |
| CHE JP | 3 | 0.1 | LV W | 1 | 0.033 |
| CHANG CP | 3 | 0.1 | LV TJ | 1 | 0.033 |
| CARPENTER S | 3 | 0.1 | LV TF | 1 | 0.033 |
| CARNERO E | 3 | 0.1 | LV SS | 1 | 0.033 |
| CAO YX | 3 | 0.1 | LV SK | 1 | 0.033 |
| CAO L | 3 | 0.1 | LV RR | 1 | 0.033 |
| CAO GH | 3 | 0.1 | LV N | 1 | 0.033 |
| CAMPBELL DB | 3 | 0.1 | LV MX | 1 | 0.033 |
| CAI XL | 3 | 0.1 | LV ML | 1 | 0.033 |
| CAI WJ | 3 | 0.1 | LV KZ | 1 | 0.033 |
| CAI MY | 3 | 0.1 | LV JJ | 1 | 0.033 |
| CAI HF | 3 | 0.1 | LV HB | 1 | 0.033 |
| CAI BZ | 3 | 0.1 | LV GX | 1 | 0.033 |
| CAI B | 3 | 0.1 | LV FQ | 1 | 0.033 |
| BROWN M | 3 | 0.1 | LV DS | 1 | 0.033 |
| BROWN JB | 3 | 0.1 | LV DK | 1 | 0.033 |
| BOYER LA | 3 | 0.1 | LV DC | 1 | 0.033 |
| BOGU GK | 3 | 0.1 | LUSSER A | 1 | 0.033 |
| BO H | 3 | 0.1 | LUPIEN M | 1 | 0.033 |
| BIERHOFF H | 3 | 0.1 | LUPEY LN | 1 | 0.033 |
| BIAN EB | 3 | 0.1 | LUONG A | 1 | 0.033 |
| BI JW | 3 | 0.1 | LUO ZW | 1 | 0.033 |
| BERGMANN JH | 3 | 0.1 | LUO ZQ | 1 | 0.033 |
| BENOIT Y | 3 | 0.1 | LUO ZL | 1 | 0.033 |
| BECKEDORFF FC | 3 | 0.1 | LUO ZJ | 1 | 0.033 |
| BASU S | 3 | 0.1 | LUO Z | 1 | 0.033 |
| BALDASSARRE A | 3 | 0.1 | LUO YQ | 1 | 0.033 |
| BAKER AH | 3 | 0.1 | LUO YP | 1 | 0.033 |
| BAJIC VB | 3 | 0.1 | LUO YM | 1 | 0.033 |
| BAGULEY BC | 3 | 0.1 | LUO YJ | 1 | 0.033 |
| AYUPE AC | 3 | 0.1 | LUO YC | 1 | 0.033 |
| ATIANAND MK | 3 | 0.1 | LUO XQ | 1 | 0.033 |
| ATHIE A | 3 | 0.1 | LUO XH | 1 | 0.033 |
| ASANGANI IA | 3 | 0.1 | LUO XG | 1 | 0.033 |
| ASAI K | 3 | 0.1 | LUO XB | 1 | 0.033 |
| ARUN G | 3 | 0.1 | LUO X | 1 | 0.033 |
| ARNAN C | 3 | 0.1 | LUO WL | 1 | 0.033 |
| ANSARI KI | 3 | 0.1 | LUO W | 1 | 0.033 |
| AN Y | 3 | 0.1 | LUO SJ | 1 | 0.033 |
| AN JH | 3 | 0.1 | LUO S | 1 | 0.033 |
| AMARAL MS | 3 | 0.1 | LUO QY | 1 | 0.033 |
| ALDER H | 3 | 0.1 | LUO P | 1 | 0.033 |
| ALCID EA | 3 | 0.1 | LUO N | 1 | 0.033 |
| AKIMITSU N | 3 | 0.1 | LUO ML | 1 | 0.033 |
| AGNELLI L | 3 | 0.1 | LUO MC | 1 | 0.033 |
| AFTAB MN | 3 | 0.1 | LUO LL | 1 | 0.033 |
| ACKLEY A | 3 | 0.1 | LUO LH | 1 | 0.033 |
| ZUO S | 2 | 0.066 | LUO JC | 1 | 0.033 |
| ZUO Q | 2 | 0.066 | LUO HY | 1 | 0.033 |
| ZU XY | 2 | 0.066 | LUO HR | 1 | 0.033 |
| ZOU ZW | 2 | 0.066 | LUO HQ | 1 | 0.033 |
| ZOU YX | 2 | 0.066 | LUO H | 1 | 0.033 |
| ZOU WP | 2 | 0.066 | LUO GX | 1 | 0.033 |
| ZOU SB | 2 | 0.066 | LUO GJ | 1 | 0.033 |
| ZOU H | 2 | 0.066 | LUO FK | 1 | 0.033 |
| ZOU C | 2 | 0.066 | LUO DL | 1 | 0.033 |
| ZOU A | 2 | 0.066 | LUO C | 1 | 0.033 |
| ZONG XY | 2 | 0.066 | LUO BH | 1 | 0.033 |
| ZHUANG ZX | 2 | 0.066 | LUNDIN E | 1 | 0.033 |
| ZHUANG Y | 2 | 0.066 | LUND SH | 1 | 0.033 |
| ZHUANG X | 2 | 0.066 | LUND RJ | 1 | 0.033 |
| ZHUANG WZ | 2 | 0.066 | LULLI M | 1 | 0.033 |
| ZHUANG WY | 2 | 0.066 | LUKANOVA A | 1 | 0.033 |
| ZHUANG L | 2 | 0.066 | LUI WO | 1 | 0.033 |
| ZHU ZX | 2 | 0.066 | LUI JH | 1 | 0.033 |
| ZHU ZF | 2 | 0.066 | LUFT FC | 1 | 0.033 |
| ZHU Z | 2 | 0.066 | LUE M | 1 | 0.033 |
| ZHU YF | 2 | 0.066 | LUDERS T | 1 | 0.033 |
| ZHU YC | 2 | 0.066 | LUCO RF | 1 | 0.033 |
| ZHU XX | 2 | 0.066 | LUCCHINI M | 1 | 0.033 |
| ZHU XQ | 2 | 0.066 | LUCAS JM | 1 | 0.033 |
| ZHU XP | 2 | 0.066 | LUCAS AB | 1 | 0.033 |
| ZHU XD | 2 | 0.066 | LUCAFO M | 1 | 0.033 |
| ZHU WJ | 2 | 0.066 | LUBELSKY Y | 1 | 0.033 |
| ZHU WH | 2 | 0.066 | LUAN XK | 1 | 0.033 |
| ZHU SK | 2 | 0.066 | LU ZP | 1 | 0.033 |
| ZHU S | 2 | 0.066 | LU ZN | 1 | 0.033 |
| ZHU Q | 2 | 0.066 | LU ZG | 1 | 0.033 |
| ZHU PW | 2 | 0.066 | LU YR | 1 | 0.033 |
| ZHU LC | 2 | 0.066 | LU YQ | 1 | 0.033 |
| ZHU KP | 2 | 0.066 | LU YM | 1 | 0.033 |
| ZHU JL | 2 | 0.066 | LU YF | 1 | 0.033 |
| ZHU JF | 2 | 0.066 | LU YC | 1 | 0.033 |
| ZHU HX | 2 | 0.066 | LU XZ | 1 | 0.033 |
| ZHU HC | 2 | 0.066 | LU XW | 1 | 0.033 |
| ZHU F | 2 | 0.066 | LU XK | 1 | 0.033 |
| ZHU B | 2 | 0.066 | LU XG | 1 | 0.033 |
| ZHOU ZY | 2 | 0.066 | LU WW | 1 | 0.033 |
| ZHOU ZW | 2 | 0.066 | LU WQ | 1 | 0.033 |
| ZHOU Z | 2 | 0.066 | LU WL | 1 | 0.033 |
| ZHOU YS | 2 | 0.066 | LU T | 1 | 0.033 |
| ZHOU YM | 2 | 0.066 | LU SS | 1 | 0.033 |
| ZHOU YL | 2 | 0.066 | LU SM | 1 | 0.033 |
| ZHOU YJ | 2 | 0.066 | LU RZ | 1 | 0.033 |
| ZHOU WZ | 2 | 0.066 | LU RF | 1 | 0.033 |
| ZHOU WQ | 2 | 0.066 | LU RB | 1 | 0.033 |
| ZHOU NJ | 2 | 0.066 | LU QS | 1 | 0.033 |
| ZHOU N | 2 | 0.066 | LU QJ | 1 | 0.033 |
| ZHOU JC | 2 | 0.066 | LU PC | 1 | 0.033 |
| ZHOU GZ | 2 | 0.066 | LU MJ | 1 | 0.033 |
| ZHOU F | 2 | 0.066 | LU MH | 1 | 0.033 |
| ZHOU DK | 2 | 0.066 | LU MC | 1 | 0.033 |
| ZHOU CY | 2 | 0.066 | LU LY | 1 | 0.033 |
| ZHONG Y | 2 | 0.066 | LU LN | 1 | 0.033 |
| ZHONG M | 2 | 0.066 | LU K | 1 | 0.033 |
| ZHONG J | 2 | 0.066 | LU JW | 1 | 0.033 |
| ZHONG D | 2 | 0.066 | LU JR | 1 | 0.033 |
| ZHONG AF | 2 | 0.066 | LU JL | 1 | 0.033 |
| ZHERNAKOVA DV | 2 | 0.066 | LU JF | 1 | 0.033 |
| ZHENG WL | 2 | 0.066 | LU HZ | 1 | 0.033 |
| ZHENG MY | 2 | 0.066 | LU HY | 1 | 0.033 |
| ZHENG M | 2 | 0.066 | LU HB | 1 | 0.033 |
| ZHENG JS | 2 | 0.066 | LU GM | 1 | 0.033 |
| ZHENG JL | 2 | 0.066 | LU GJ | 1 | 0.033 |
| ZHENG JF | 2 | 0.066 | LU DK | 1 | 0.033 |
| ZHENG H | 2 | 0.066 | LU DH | 1 | 0.033 |
| ZHENG GQ | 2 | 0.066 | LU CY | 1 | 0.033 |
| ZHENG DL | 2 | 0.066 | LU CX | 1 | 0.033 |
| ZHENG DH | 2 | 0.066 | LU CR | 1 | 0.033 |
| ZHENG DC | 2 | 0.066 | LU CM | 1 | 0.033 |
| ZHENG D | 2 | 0.066 | LU CJ | 1 | 0.033 |
| ZHENG BY | 2 | 0.066 | LU BJ | 1 | 0.033 |
| ZHAO ZM | 2 | 0.066 | LOWE JK | 1 | 0.033 |
| ZHAO ZL | 2 | 0.066 | LOWE BS | 1 | 0.033 |
| ZHAO ZJ | 2 | 0.066 | LOURENCO GF | 1 | 0.033 |
| ZHAO YM | 2 | 0.066 | LOU ZK | 1 | 0.033 |
| ZHAO YF | 2 | 0.066 | LOU Y | 1 | 0.033 |
| ZHAO YB | 2 | 0.066 | LOU GY | 1 | 0.033 |
| ZHAO WM | 2 | 0.066 | LOU G | 1 | 0.033 |
| ZHAO TT | 2 | 0.066 | LOSKO M | 1 | 0.033 |
| ZHAO SX | 2 | 0.066 | LOSAVIO FA | 1 | 0.033 |
| ZHAO SG | 2 | 0.066 | LORENZI JCC | 1 | 0.033 |
| ZHAO RC | 2 | 0.066 | LOPEZ-SERRA P | 1 | 0.033 |
| ZHAO QC | 2 | 0.066 | LOPEZ-DOMINGO FJ | 1 | 0.033 |
| ZHAO MC | 2 | 0.066 | LOPEZ-CALDERON FF | 1 | 0.033 |
| ZHAO LY | 2 | 0.066 | LOPEZ JP | 1 | 0.033 |
| ZHAO LM | 2 | 0.066 | LOPEZ CB | 1 | 0.033 |
| ZHAO KJ | 2 | 0.066 | LOPES R | 1 | 0.033 |
| ZHAO K | 2 | 0.066 | LOOSO M | 1 | 0.033 |
| ZHAO JS | 2 | 0.066 | LOOK MP | 1 | 0.033 |
| ZHAO JP | 2 | 0.066 | LOO LWM | 1 | 0.033 |
| ZHAO HL | 2 | 0.066 | LONGERICH T | 1 | 0.033 |
| ZHAO HJ | 2 | 0.066 | LONG ZQ | 1 | 0.033 |
| ZHAO FS | 2 | 0.066 | LONG YP | 1 | 0.033 |
| ZHAO FJ | 2 | 0.066 | LONG X | 1 | 0.033 |
| ZHAO F | 2 | 0.066 | LONG RW | 1 | 0.033 |
| ZHAO BX | 2 | 0.066 | LONG NY | 1 | 0.033 |
| ZHANG ZZ | 2 | 0.066 | LONG L | 1 | 0.033 |
| ZHANG ZS | 2 | 0.066 | LONG JY | 1 | 0.033 |
| ZHANG ZK | 2 | 0.066 | LONG HB | 1 | 0.033 |
| ZHANG ZC | 2 | 0.066 | LOJEK L | 1 | 0.033 |
| ZHANG YW | 2 | 0.066 | LOHR CV | 1 | 0.033 |
| ZHANG YM | 2 | 0.066 | LOH YH | 1 | 0.033 |
| ZHANG YH | 2 | 0.066 | LOEWER S | 1 | 0.033 |
| ZHANG YG | 2 | 0.066 | LOEWEN G | 1 | 0.033 |
| ZHANG XZ | 2 | 0.066 | LOEFFLER M | 1 | 0.033 |
| ZHANG XG | 2 | 0.066 | LOEB DM | 1 | 0.033 |
| ZHANG WS | 2 | 0.066 | LODATO S | 1 | 0.033 |
| ZHANG WG | 2 | 0.066 | LODA M | 1 | 0.033 |
| ZHANG TJ | 2 | 0.066 | LO PK | 1 | 0.033 |
| ZHANG SZ | 2 | 0.066 | LO DICO A | 1 | 0.033 |
| ZHANG SX | 2 | 0.066 | LLORENTE-CORTES V | 1 | 0.033 |
| ZHANG SS | 2 | 0.066 | LJUNGMAN M | 1 | 0.033 |
| ZHANG RY | 2 | 0.066 | LIU ZP | 1 | 0.033 |
| ZHANG RJ | 2 | 0.066 | LIU ZN | 1 | 0.033 |
| ZHANG RF | 2 | 0.066 | LIU YZ | 1 | 0.033 |
| ZHANG QY | 2 | 0.066 | LIU YP | 1 | 0.033 |
| ZHANG QS | 2 | 0.066 | LIU XR | 1 | 0.033 |
| ZHANG QP | 2 | 0.066 | LIU XLS | 1 | 0.033 |
| ZHANG QF | 2 | 0.066 | LIU WY | 1 | 0.033 |
| ZHANG PP | 2 | 0.066 | LIU WH | 1 | 0.033 |
| ZHANG PJ | 2 | 0.066 | LIU WB | 1 | 0.033 |
| ZHANG MX | 2 | 0.066 | LIU TX | 1 | 0.033 |
| ZHANG MQ | 2 | 0.066 | LIU TT | 1 | 0.033 |
| ZHANG LZ | 2 | 0.066 | LIU TM | 1 | 0.033 |
| ZHANG LX | 2 | 0.066 | LIU TL | 1 | 0.033 |
| ZHANG LQ | 2 | 0.066 | LIU SK | 1 | 0.033 |
| ZHANG LH | 2 | 0.066 | LIU SG | 1 | 0.033 |
| ZHANG KL | 2 | 0.066 | LIU SD | 1 | 0.033 |
| ZHANG HR | 2 | 0.066 | LIU RY | 1 | 0.033 |
| ZHANG HQ | 2 | 0.066 | LIU RM | 1 | 0.033 |
| ZHANG HP | 2 | 0.066 | LIU QW | 1 | 0.033 |
| ZHANG HF | 2 | 0.066 | LIU QQ | 1 | 0.033 |
| ZHANG GQ | 2 | 0.066 | LIU QH | 1 | 0.033 |
| ZHANG GM | 2 | 0.066 | LIU QG | 1 | 0.033 |
| ZHANG GL | 2 | 0.066 | LIU PJ | 1 | 0.033 |
| ZHANG GH | 2 | 0.066 | LIU PG | 1 | 0.033 |
| ZHANG FF | 2 | 0.066 | LIU PF | 1 | 0.033 |
| ZHANG DX | 2 | 0.066 | LIU MY | 1 | 0.033 |
| ZHANG DM | 2 | 0.066 | LIU MW | 1 | 0.033 |
| ZHANG DL | 2 | 0.066 | LIU MJ | 1 | 0.033 |
| ZHANG CR | 2 | 0.066 | LIU MH | 1 | 0.033 |
| ZHANG CF | 2 | 0.066 | LIU LW | 1 | 0.033 |
| ZHANG BY | 2 | 0.066 | LIU LT | 1 | 0.033 |
| ZHANG AQ | 2 | 0.066 | LIU LS | 1 | 0.033 |
| ZHAN Q | 2 | 0.066 | LIU LF | 1 | 0.033 |
| ZHAN P | 2 | 0.066 | LIU LC | 1 | 0.033 |
| ZHAN C | 2 | 0.066 | LIU LB | 1 | 0.033 |
| ZHAI XJ | 2 | 0.066 | LIU KY | 1 | 0.033 |
| ZHAI H | 2 | 0.066 | LIU KR | 1 | 0.033 |
| ZHAI B | 2 | 0.066 | LIU JT | 1 | 0.033 |
| ZENG YY | 2 | 0.066 | LIU JP | 1 | 0.033 |
| ZENG X | 2 | 0.066 | LIU JN | 1 | 0.033 |
| ZENG S | 2 | 0.066 | LIU JG | 1 | 0.033 |
| ZENG P | 2 | 0.066 | LIU HW | 1 | 0.033 |
| ZENG JL | 2 | 0.066 | LIU HS | 1 | 0.033 |
| ZEHNDER A | 2 | 0.066 | LIU HR | 1 | 0.033 |
| ZANGRANDO J | 2 | 0.066 | LIU HM | 1 | 0.033 |
| ZACCAGNINI G | 2 | 0.066 | LIU HG | 1 | 0.033 |
| YUE XL | 2 | 0.066 | LIU GB | 1 | 0.033 |
| YUE M | 2 | 0.066 | LIU FS | 1 | 0.033 |
| YUE B | 2 | 0.066 | LIU FL | 1 | 0.033 |
| YUAN YF | 2 | 0.066 | LIU FJ | 1 | 0.033 |
| YUAN XY | 2 | 0.066 | LIU FF | 1 | 0.033 |
| YUAN TY | 2 | 0.066 | LIU FB | 1 | 0.033 |
| YUAN JL | 2 | 0.066 | LIU DZ | 1 | 0.033 |
| YUAN JC | 2 | 0.066 | LIU DY | 1 | 0.033 |
| YUAN CC | 2 | 0.066 | LIU DP | 1 | 0.033 |
| YU ZX | 2 | 0.066 | LIU DM | 1 | 0.033 |
| YU ZJ | 2 | 0.066 | LIU DF | 1 | 0.033 |
| YU ZH | 2 | 0.066 | LIU DC | 1 | 0.033 |
| YU YW | 2 | 0.066 | LIU CT | 1 | 0.033 |
| YU YT | 2 | 0.066 | LIU CF | 1 | 0.033 |
| YU XX | 2 | 0.066 | LIU CB | 1 | 0.033 |
| YU XB | 2 | 0.066 | LIU BW | 1 | 0.033 |
| YU WG | 2 | 0.066 | LIU BR | 1 | 0.033 |
| YU WB | 2 | 0.066 | LIU BL | 1 | 0.033 |
| YU SZ | 2 | 0.066 | LIU BJ | 1 | 0.033 |
| YU R | 2 | 0.066 | LIU BH | 1 | 0.033 |
| YU PF | 2 | 0.066 | LIU BF | 1 | 0.033 |
| YU L | 2 | 0.066 | LIU BD | 1 | 0.033 |
| YU KT | 2 | 0.066 | LIU AW | 1 | 0.033 |
| YU JP | 2 | 0.066 | LIU AH | 1 | 0.033 |
| YU G | 2 | 0.066 | LIU AG | 1 | 0.033 |
| YU FD | 2 | 0.066 | LITTMAN DR | 1 | 0.033 |
| YU CL | 2 | 0.066 | LISKA V | 1 | 0.033 |
| YU CH | 2 | 0.066 | LISITSYN NA | 1 | 0.033 |
| YOUNG RS | 2 | 0.066 | LISI V | 1 | 0.033 |
| YOU YH | 2 | 0.066 | LISEC K | 1 | 0.033 |
| YOU L | 2 | 0.066 | LISA A | 1 | 0.033 |
| YORK SR | 2 | 0.066 | LIPSKA L | 1 | 0.033 |
| YOO JS | 2 | 0.066 | LIPKIN SM | 1 | 0.033 |
| YONEDA R | 2 | 0.066 | LIP V | 1 | 0.033 |
| YING KJ | 2 | 0.066 | LIOU J | 1 | 0.033 |
| YIN YF | 2 | 0.066 | LIONETTI M | 1 | 0.033 |
| YIN HL | 2 | 0.066 | LING ZQ | 1 | 0.033 |
| YIN C | 2 | 0.066 | LING M | 1 | 0.033 |
| YI ZJ | 2 | 0.066 | LING HB | 1 | 0.033 |
| YI ZH | 2 | 0.066 | LING D | 1 | 0.033 |
| YI K | 2 | 0.066 | LINDSEY K | 1 | 0.033 |
| YI F | 2 | 0.066 | LINDROTH AM | 1 | 0.033 |
| YE ZQ | 2 | 0.066 | LINDOW M | 1 | 0.033 |
| YE YC | 2 | 0.066 | LINDGREN A | 1 | 0.033 |
| YE S | 2 | 0.066 | LINDEN PA | 1 | 0.033 |
| YE N | 2 | 0.066 | LIND SB | 1 | 0.033 |
| YE FQ | 2 | 0.066 | LIN ZQ | 1 | 0.033 |
| YE F | 2 | 0.066 | LIN ZM | 1 | 0.033 |
| YE DQ | 2 | 0.066 | LIN ZJ | 1 | 0.033 |
| YE C | 2 | 0.066 | LIN ZH | 1 | 0.033 |
| YAVIN E | 2 | 0.066 | LIN ZG | 1 | 0.033 |
| YAO X | 2 | 0.066 | LIN Z | 1 | 0.033 |
| YAO WM | 2 | 0.066 | LIN YX | 1 | 0.033 |
| YAO R | 2 | 0.066 | LIN YW | 1 | 0.033 |
| YAO D | 2 | 0.066 | LIN YN | 1 | 0.033 |
| YANG ZS | 2 | 0.066 | LIN YL | 1 | 0.033 |
| YANG ZJ | 2 | 0.066 | LIN YH | 1 | 0.033 |
| YANG ZC | 2 | 0.066 | LIN YC | 1 | 0.033 |
| YANG YT | 2 | 0.066 | LIN XY | 1 | 0.033 |
| YANG YH | 2 | 0.066 | LIN XX | 1 | 0.033 |
| YANG YF | 2 | 0.066 | LIN XR | 1 | 0.033 |
| YANG YA | 2 | 0.066 | LIN XB | 1 | 0.033 |
| YANG XH | 2 | 0.066 | LIN V | 1 | 0.033 |
| YANG XF | 2 | 0.066 | LIN SM | 1 | 0.033 |
| YANG XD | 2 | 0.066 | LIN SF | 1 | 0.033 |
| YANG WL | 2 | 0.066 | LIN SC | 1 | 0.033 |
| YANG WJ | 2 | 0.066 | LIN S | 1 | 0.033 |
| YANG WH | 2 | 0.066 | LIN RY | 1 | 0.033 |
| YANG SQ | 2 | 0.066 | LIN RX | 1 | 0.033 |
| YANG SL | 2 | 0.066 | LIN RF | 1 | 0.033 |
| YANG RC | 2 | 0.066 | LIN QS | 1 | 0.033 |
| YANG QQ | 2 | 0.066 | LIN QF | 1 | 0.033 |
| YANG MH | 2 | 0.066 | LIN PC | 1 | 0.033 |
| YANG LT | 2 | 0.066 | LIN MH | 1 | 0.033 |
| YANG LH | 2 | 0.066 | LIN MF | 1 | 0.033 |
| YANG KY | 2 | 0.066 | LIN LZ | 1 | 0.033 |
| YANG K | 2 | 0.066 | LIN LW | 1 | 0.033 |
| YANG JX | 2 | 0.066 | LIN LG | 1 | 0.033 |
| YANG JW | 2 | 0.066 | LIN KC | 1 | 0.033 |
| YANG JK | 2 | 0.066 | LIN JY | 1 | 0.033 |
| YANG JB | 2 | 0.066 | LIN JX | 1 | 0.033 |
| YANG HW | 2 | 0.066 | LIN HX | 1 | 0.033 |
| YANG HL | 2 | 0.066 | LIN HS | 1 | 0.033 |
| YANG DY | 2 | 0.066 | LIN HK | 1 | 0.033 |
| YANG CF | 2 | 0.066 | LIN GZ | 1 | 0.033 |
| YANG BF | 2 | 0.066 | LIN GW | 1 | 0.033 |
| YANG B | 2 | 0.066 | LIN GG | 1 | 0.033 |
| YANCOPOULOS GD | 2 | 0.066 | LIN FP | 1 | 0.033 |
| YANAKA K | 2 | 0.066 | LIN F | 1 | 0.033 |
| YAN YY | 2 | 0.066 | LIN E | 1 | 0.033 |
| YAN XY | 2 | 0.066 | LIN DX | 1 | 0.033 |
| YAN WW | 2 | 0.066 | LIN DP | 1 | 0.033 |
| YAN TH | 2 | 0.066 | LIN CW | 1 | 0.033 |
| YAN PX | 2 | 0.066 | LIN CM | 1 | 0.033 |
| YAN LL | 2 | 0.066 | LIN CLS | 1 | 0.033 |
| YAN JB | 2 | 0.066 | LIN CJ | 1 | 0.033 |
| YAN F | 2 | 0.066 | LIN CH | 1 | 0.033 |
| YAN C | 2 | 0.066 | LIN C | 1 | 0.033 |
| YAN BA | 2 | 0.066 | LIN BJ | 1 | 0.033 |
| YAMAZAKI T | 2 | 0.066 | LIN BC | 1 | 0.033 |
| XUE WJ | 2 | 0.066 | LIN AF | 1 | 0.033 |
| XUE L | 2 | 0.066 | LIMA LA | 1 | 0.033 |
| XUE F | 2 | 0.066 | LIM YP | 1 | 0.033 |
| XUE D | 2 | 0.066 | LIM YC | 1 | 0.033 |
| XUE CY | 2 | 0.066 | LIM JA | 1 | 0.033 |
| XUAN ZY | 2 | 0.066 | LIM J | 1 | 0.033 |
| XU YZ | 2 | 0.066 | LIM BL | 1 | 0.033 |
| XU YL | 2 | 0.066 | LIM B | 1 | 0.033 |
| XU XQ | 2 | 0.066 | LIGUORI GL | 1 | 0.033 |
| XU XH | 2 | 0.066 | LIECHTI A | 1 | 0.033 |
| XU WH | 2 | 0.066 | LIEBERMAN D | 1 | 0.033 |
| XU WC | 2 | 0.066 | LICKISS T | 1 | 0.033 |
| XU T | 2 | 0.066 | LIAW CC | 1 | 0.033 |
| XU SJ | 2 | 0.066 | LIAO YW | 1 | 0.033 |
| XU RF | 2 | 0.066 | LIAO Y | 1 | 0.033 |
| XU QH | 2 | 0.066 | LIAO XY | 1 | 0.033 |
| XU QG | 2 | 0.066 | LIAO XH | 1 | 0.033 |
| XU K | 2 | 0.066 | LIAO WM | 1 | 0.033 |
| XU JZ | 2 | 0.066 | LIAO WJ | 1 | 0.033 |
| XU JW | 2 | 0.066 | LIAO RW | 1 | 0.033 |
| XU JH | 2 | 0.066 | LIAO MZ | 1 | 0.033 |
| XU JB | 2 | 0.066 | LIAO MM | 1 | 0.033 |
| XU HD | 2 | 0.066 | LIAO M | 1 | 0.033 |
| XU HB | 2 | 0.066 | LIAO LM | 1 | 0.033 |
| XU DL | 2 | 0.066 | LIAO LK | 1 | 0.033 |
| XU AM | 2 | 0.066 | LIAO K | 1 | 0.033 |
| XIONG YL | 2 | 0.066 | LIAO JQ | 1 | 0.033 |
| XIONG XY | 2 | 0.066 | LIAO J | 1 | 0.033 |
| XIONG F | 2 | 0.066 | LIANG ZW | 1 | 0.033 |
| XING Y | 2 | 0.066 | LIANG ZK | 1 | 0.033 |
| XIN Y | 2 | 0.066 | LIANG YJ | 1 | 0.033 |
| XIE L | 2 | 0.066 | LIANG XW | 1 | 0.033 |
| XIE JY | 2 | 0.066 | LIANG XS | 1 | 0.033 |
| XIE JK | 2 | 0.066 | LIANG XL | 1 | 0.033 |
| XIE JJ | 2 | 0.066 | LIANG XB | 1 | 0.033 |
| XIE HJ | 2 | 0.066 | LIANG WJ | 1 | 0.033 |
| XIAO ZL | 2 | 0.066 | LIANG W | 1 | 0.033 |
| XIAO ZD | 2 | 0.066 | LIANG TY | 1 | 0.033 |
| XIAO YL | 2 | 0.066 | LIANG SH | 1 | 0.033 |
| XIAO YB | 2 | 0.066 | LIANG Q | 1 | 0.033 |
| XIAO XS | 2 | 0.066 | LIANG MY | 1 | 0.033 |
| XIAO W | 2 | 0.066 | LIANG M | 1 | 0.033 |
| XIAO J | 2 | 0.066 | LIANG LX | 1 | 0.033 |
| XIAO C | 2 | 0.066 | LIANG LL | 1 | 0.033 |
| XIAO B | 2 | 0.066 | LIANG KH | 1 | 0.033 |
| XIANG Y | 2 | 0.066 | LIANG JQ | 1 | 0.033 |
| XIANG X | 2 | 0.066 | LIANG JJ | 1 | 0.033 |
| XIANG MQ | 2 | 0.066 | LIANG HX | 1 | 0.033 |
| XIANG JY | 2 | 0.066 | LIANG HH | 1 | 0.033 |
| XIANG GM | 2 | 0.066 | LIANG GQ | 1 | 0.033 |
| XIA Z | 2 | 0.066 | LIANG FJ | 1 | 0.033 |
| XIA YK | 2 | 0.066 | LIANG E | 1 | 0.033 |
| XIA W | 2 | 0.066 | LIANG D | 1 | 0.033 |
| XIA SY | 2 | 0.066 | LIANG CL | 1 | 0.033 |
| XIA GF | 2 | 0.066 | LIANG C | 1 | 0.033 |
| XI Z | 2 | 0.066 | LIANG BH | 1 | 0.033 |
| WYSOCKA J | 2 | 0.066 | LIANG B | 1 | 0.033 |
| WU ZZ | 2 | 0.066 | LIAN XQ | 1 | 0.033 |
| WU ZQ | 2 | 0.066 | LIAN T | 1 | 0.033 |
| WU YT | 2 | 0.066 | LIAN M | 1 | 0.033 |
| WU YQ | 2 | 0.066 | LIAN JC | 1 | 0.033 |
| WU YM | 2 | 0.066 | LIAN JB | 1 | 0.033 |
| WU YB | 2 | 0.066 | LIAN GY | 1 | 0.033 |
| WU XF | 2 | 0.066 | LIAN D | 1 | 0.033 |
| WU WQ | 2 | 0.066 | LI YZ | 1 | 0.033 |
| WU WKK | 2 | 0.066 | LI YR | 1 | 0.033 |
| WU SY | 2 | 0.066 | LI YK | 1 | 0.033 |
| WU SX | 2 | 0.066 | LI YF | 1 | 0.033 |
| WU SJ | 2 | 0.066 | LI YB | 1 | 0.033 |
| WU SC | 2 | 0.066 | LI WT | 1 | 0.033 |
| WU N | 2 | 0.066 | LI WS | 1 | 0.033 |
| WU MJ | 2 | 0.066 | LI WL | 1 | 0.033 |
| WU MH | 2 | 0.066 | LI WG | 1 | 0.033 |
| WU LH | 2 | 0.066 | LI WD | 1 | 0.033 |
| WU LF | 2 | 0.066 | LI WC | 1 | 0.033 |
| WU JW | 2 | 0.066 | LI WB | 1 | 0.033 |
| WU JQ | 2 | 0.066 | LI TW | 1 | 0.033 |
| WU JC | 2 | 0.066 | LI TT | 1 | 0.033 |
| WU HX | 2 | 0.066 | LI TS | 1 | 0.033 |
| WU HL | 2 | 0.066 | LI TP | 1 | 0.033 |
| WU GQ | 2 | 0.066 | LI TL | 1 | 0.033 |
| WU DG | 2 | 0.066 | LI SX | 1 | 0.033 |
| WU DC | 2 | 0.066 | LI ST | 1 | 0.033 |
| WU BM | 2 | 0.066 | LI SP | 1 | 0.033 |
| WRIGHT CM | 2 | 0.066 | LI SM | 1 | 0.033 |
| WONG M | 2 | 0.066 | LI SK | 1 | 0.033 |
| WONG KY | 2 | 0.066 | LI SJ | 1 | 0.033 |
| WOLVETANG EJ | 2 | 0.066 | LI SH | 1 | 0.033 |
| WIRTH D | 2 | 0.066 | LI SD | 1 | 0.033 |
| WINKLE M | 2 | 0.066 | LI RY | 1 | 0.033 |
| WILLIAMS A | 2 | 0.066 | LI RQ | 1 | 0.033 |
| WILDMAN DE | 2 | 0.066 | LI RK | 1 | 0.033 |
| WILDER-ROMANS K | 2 | 0.066 | LI RJ | 1 | 0.033 |
| WILBER A | 2 | 0.066 | LI RF | 1 | 0.033 |
| WIJMENGA C | 2 | 0.066 | LI QW | 1 | 0.033 |
| WENTZEL K | 2 | 0.066 | LI QH | 1 | 0.033 |
| WENG WH | 2 | 0.066 | LI PW | 1 | 0.033 |
| WEN XY | 2 | 0.066 | LI NF | 1 | 0.033 |
| WEN X | 2 | 0.066 | LI MZ | 1 | 0.033 |
| WEN LW | 2 | 0.066 | LI MW | 1 | 0.033 |
| WEN H | 2 | 0.066 | LI MR | 1 | 0.033 |
| WEI WJ | 2 | 0.066 | LI MJ | 1 | 0.033 |
| WEI W | 2 | 0.066 | LI MF | 1 | 0.033 |
| WEI PP | 2 | 0.066 | LI LX | 1 | 0.033 |
| WARD AJ | 2 | 0.066 | LI LT | 1 | 0.033 |
| WANG ZW | 2 | 0.066 | LI LS | 1 | 0.033 |
| WANG ZC | 2 | 0.066 | LI LQ | 1 | 0.033 |
| WANG YT | 2 | 0.066 | LI LB | 1 | 0.033 |
| WANG YN | 2 | 0.066 | LI KX | 1 | 0.033 |
| WANG YH | 2 | 0.066 | LI KR | 1 | 0.033 |
| WANG XS | 2 | 0.066 | LI KQ | 1 | 0.033 |
| WANG XC | 2 | 0.066 | LI KN | 1 | 0.033 |
| WANG WW | 2 | 0.066 | LI KG | 1 | 0.033 |
| WANG WT | 2 | 0.066 | LI JT | 1 | 0.033 |
| WANG WQ | 2 | 0.066 | LI JN | 1 | 0.033 |
| WANG WM | 2 | 0.066 | LI JD | 1 | 0.033 |
| WANG TH | 2 | 0.066 | LI HZ | 1 | 0.033 |
| WANG SL | 2 | 0.066 | LI HP | 1 | 0.033 |
| WANG SK | 2 | 0.066 | LI HH | 1 | 0.033 |
| WANG QS | 2 | 0.066 | LI HG | 1 | 0.033 |
| WANG QJ | 2 | 0.066 | LI HB | 1 | 0.033 |
| WANG QG | 2 | 0.066 | LI GZ | 1 | 0.033 |
| WANG PY | 2 | 0.066 | LI GH | 1 | 0.033 |
| WANG PC | 2 | 0.066 | LI GD | 1 | 0.033 |
| WANG NN | 2 | 0.066 | LI GC | 1 | 0.033 |
| WANG MR | 2 | 0.066 | LI GB | 1 | 0.033 |
| WANG LT | 2 | 0.066 | LI FY | 1 | 0.033 |
| WANG LS | 2 | 0.066 | LI FX | 1 | 0.033 |
| WANG LQ | 2 | 0.066 | LI EM | 1 | 0.033 |
| WANG LG | 2 | 0.066 | LI DY | 1 | 0.033 |
| WANG KK | 2 | 0.066 | LI DX | 1 | 0.033 |
| WANG JQ | 2 | 0.066 | LI DW | 1 | 0.033 |
| WANG JM | 2 | 0.066 | LI DP | 1 | 0.033 |
| WANG JF | 2 | 0.066 | LI DM | 1 | 0.033 |
| WANG HZ | 2 | 0.066 | LI DD | 1 | 0.033 |
| WANG HH | 2 | 0.066 | LI DC | 1 | 0.033 |
| WANG GQ | 2 | 0.066 | LI CT | 1 | 0.033 |
| WANG GF | 2 | 0.066 | LI CSR | 1 | 0.033 |
| WANG FL | 2 | 0.066 | LI CR | 1 | 0.033 |
| WANG DZ | 2 | 0.066 | LI CP | 1 | 0.033 |
| WANG DY | 2 | 0.066 | LI CF | 1 | 0.033 |
| WANG DS | 2 | 0.066 | LI CC | 1 | 0.033 |
| WANG DB | 2 | 0.066 | LI BY | 1 | 0.033 |
| WANG CQ | 2 | 0.066 | LI BX | 1 | 0.033 |
| WANG CJ | 2 | 0.066 | LI BL | 1 | 0.033 |
| WANG CF | 2 | 0.066 | LI BJ | 1 | 0.033 |
| WANG BR | 2 | 0.066 | LI AJ | 1 | 0.033 |
| WANG BH | 2 | 0.066 | LI AD | 1 | 0.033 |
| WANG BD | 2 | 0.066 | LEWITUS E | 1 | 0.033 |
| WANG AQ | 2 | 0.066 | LEWIS A | 1 | 0.033 |
| WAN XC | 2 | 0.066 | LEVY S | 1 | 0.033 |
| WAN GH | 2 | 0.066 | LEVY M | 1 | 0.033 |
| WAN DW | 2 | 0.066 | LEVEILLE N | 1 | 0.033 |
| WALLAERT A | 2 | 0.066 | LESHKOWITZ D | 1 | 0.033 |
| VOELLENKLE C | 2 | 0.066 | LESCHE M | 1 | 0.033 |
| VISSER L | 2 | 0.066 | LESCALLETTE AR | 1 | 0.033 |
| VISAKORPI T | 2 | 0.066 | LEPRIVIER G | 1 | 0.033 |
| VILLAMIZAR O | 2 | 0.066 | LEPPANEN SP | 1 | 0.033 |
| VERMA S | 2 | 0.066 | LEPPA V | 1 | 0.033 |
| VERMA A | 2 | 0.066 | LEONELLI C | 1 | 0.033 |
| VERGARA IA | 2 | 0.066 | LEONARDI T | 1 | 0.033 |
| VENNIN C | 2 | 0.066 | LENZI L | 1 | 0.033 |
| VASEI M | 2 | 0.066 | LENZE D | 1 | 0.033 |
| VAN VLIERBERGHE P | 2 | 0.066 | LENVIK TR | 1 | 0.033 |
| VAN ROY N | 2 | 0.066 | LENNOX KA | 1 | 0.033 |
| VAN ROOSBROECK K | 2 | 0.066 | LENG XC | 1 | 0.033 |
| VAN LOOCKE W | 2 | 0.066 | LENG WB | 1 | 0.033 |
| VAN DEN OORD J | 2 | 0.066 | LENG JY | 1 | 0.033 |
| VAN DEN BERG A | 2 | 0.066 | LENG F | 1 | 0.033 |
| VALERO V | 2 | 0.066 | LEMZE D | 1 | 0.033 |
| VALENZUELA-MIRANDA D | 2 | 0.066 | LEMOS AEG | 1 | 0.033 |
| VALENZUELA DM | 2 | 0.066 | LEMMON VP | 1 | 0.033 |
| VALEN E | 2 | 0.066 | LEMIRE-BRACHAT S | 1 | 0.033 |
| URANO T | 2 | 0.066 | LEMESLE G | 1 | 0.033 |
| UEDA M | 2 | 0.066 | LELLOUCHE JP | 1 | 0.033 |
| UDDIN M | 2 | 0.066 | LELLOUCHE E | 1 | 0.033 |
| UDAGER AM | 2 | 0.066 | LELEU M | 1 | 0.033 |
| UCHI R | 2 | 0.066 | LELAY-TAHA MN | 1 | 0.033 |
| TU G | 2 | 0.066 | LEISEGANG MS | 1 | 0.033 |
| TSUTSUMI S | 2 | 0.066 | LEIDNER RS | 1 | 0.033 |
| TSUNODA T | 2 | 0.066 | LEIBOVICH L | 1 | 0.033 |
| TSAI MH | 2 | 0.066 | LEI ZJ | 1 | 0.033 |
| TRIPATHI V | 2 | 0.066 | LEI YX | 1 | 0.033 |
| TRAPNELL C | 2 | 0.066 | LEI YL | 1 | 0.033 |
| TRAN EJ | 2 | 0.066 | LEI S | 1 | 0.033 |
| TRAMONTANO A | 2 | 0.066 | LEI RH | 1 | 0.033 |
| TONG X | 2 | 0.066 | LEI MG | 1 | 0.033 |
| TOKINO T | 2 | 0.066 | LEI JB | 1 | 0.033 |
| TODOERTI K | 2 | 0.066 | LEI FR | 1 | 0.033 |
| TILI E | 2 | 0.066 | LEI CZ | 1 | 0.033 |
| TIBBIT C | 2 | 0.066 | LEI CY | 1 | 0.033 |
| TIAN YQ | 2 | 0.066 | LEI B | 1 | 0.033 |
| TIAN YJ | 2 | 0.066 | LEGEAI F | 1 | 0.033 |
| TIAN X | 2 | 0.066 | LEE YY | 1 | 0.033 |
| TIAN W | 2 | 0.066 | LEE YS | 1 | 0.033 |
| TIAN T | 2 | 0.066 | LEE YC | 1 | 0.033 |
| TIAN LQ | 2 | 0.066 | LEE TJ | 1 | 0.033 |
| TIAN JX | 2 | 0.066 | LEE SW | 1 | 0.033 |
| TIAN H | 2 | 0.066 | LEE SM | 1 | 0.033 |
| TIAN BN | 2 | 0.066 | LEE SJ | 1 | 0.033 |
| THOMASSEN M | 2 | 0.066 | LEE SH | 1 | 0.033 |
| THOMAS MJ | 2 | 0.066 | LEE SC | 1 | 0.033 |
| THOMAS K | 2 | 0.066 | LEE SB | 1 | 0.033 |
| TERPSTRA M | 2 | 0.066 | LEE RT | 1 | 0.033 |
| TEOH JP | 2 | 0.066 | LEE NK | 1 | 0.033 |
| TEOH H | 2 | 0.066 | LEE MGS | 1 | 0.033 |
| TENG YQ | 2 | 0.066 | LEE M | 1 | 0.033 |
| TENG H | 2 | 0.066 | LEE KY | 1 | 0.033 |
| TAZON-VEGA B | 2 | 0.066 | LEE JMF | 1 | 0.033 |
| TAYLOR HS | 2 | 0.066 | LEE JK | 1 | 0.033 |
| TARCA AL | 2 | 0.066 | LEE JJ | 1 | 0.033 |
| TAO ZF | 2 | 0.066 | LEE EK | 1 | 0.033 |
| TAO YG | 2 | 0.066 | LEE DW | 1 | 0.033 |
| TAO X | 2 | 0.066 | LEE DS | 1 | 0.033 |
| TAO T | 2 | 0.066 | LEE DM | 1 | 0.033 |
| TAO JG | 2 | 0.066 | LEE CC | 1 | 0.033 |
| TANZER A | 2 | 0.066 | LEE BY | 1 | 0.033 |
| TANNIR NM | 2 | 0.066 | LEE BB | 1 | 0.033 |
| TANIGAWA A | 2 | 0.066 | LEE AK | 1 | 0.033 |
| TANG Z | 2 | 0.066 | LEBOEUF D | 1 | 0.033 |
| TANG YL | 2 | 0.066 | LE CAO KA | 1 | 0.033 |
| TANG YF | 2 | 0.066 | LE CALVEZ-KELM F | 1 | 0.033 |
| TANG XX | 2 | 0.066 | LE BOURHIS X | 1 | 0.033 |
| TANG XH | 2 | 0.066 | LAZAR Z | 1 | 0.033 |
| TANG SS | 2 | 0.066 | LAZAR DC | 1 | 0.033 |
| TANG RX | 2 | 0.066 | LAYER R | 1 | 0.033 |
| TANG R | 2 | 0.066 | LAXMAN B | 1 | 0.033 |
| TANG JT | 2 | 0.066 | LAWRENSON K | 1 | 0.033 |
| TANG J | 2 | 0.066 | LAWRENCE M | 1 | 0.033 |
| TANAKA Y | 2 | 0.066 | LAWRENCE JB | 1 | 0.033 |
| TANAKA N | 2 | 0.066 | LAWLESS MW | 1 | 0.033 |
| TAN XM | 2 | 0.066 | LAWERENZ C | 1 | 0.033 |
| TAN RJ | 2 | 0.066 | LAW IKM | 1 | 0.033 |
| TAN QH | 2 | 0.066 | LAVORGNA G | 1 | 0.033 |
| TAN Q | 2 | 0.066 | LAVI S | 1 | 0.033 |
| TAN LY | 2 | 0.066 | LAURI A | 1 | 0.033 |
| TAN LX | 2 | 0.066 | LAURETTE P | 1 | 0.033 |
| TAN JZ | 2 | 0.066 | LAUGIER L | 1 | 0.033 |
| TAN JY | 2 | 0.066 | LAUER RC | 1 | 0.033 |
| TAN H | 2 | 0.066 | LAUDES M | 1 | 0.033 |
| TAN B | 2 | 0.066 | LAU P | 1 | 0.033 |
| TAKAYAMA K | 2 | 0.066 | LATRONICO MVG | 1 | 0.033 |
| TAKANO Y | 2 | 0.066 | LATOS PA | 1 | 0.033 |
| TAIANA E | 2 | 0.066 | LATONEN L | 1 | 0.033 |
| TAHIRA AC | 2 | 0.066 | LATIL M | 1 | 0.033 |
| TAFT RJ | 2 | 0.066 | LASSMANN T | 1 | 0.033 |
| SZAFRANSKI P | 2 | 0.066 | LASALLE JM | 1 | 0.033 |
| SYRING I | 2 | 0.066 | LARDENOIS A | 1 | 0.033 |
| SUZUKI T | 2 | 0.066 | LAQUERRE J | 1 | 0.033 |
| SUZUKI S | 2 | 0.066 | LAPOUGE G | 1 | 0.033 |
| SUSSEL L | 2 | 0.066 | LAPORTE P | 1 | 0.033 |
| SUNWOO JS | 2 | 0.066 | LAPOINTE LC | 1 | 0.033 |
| SUNWOO H | 2 | 0.066 | LANZA G | 1 | 0.033 |
| SUNKIN SM | 2 | 0.066 | LANZ RB | 1 | 0.033 |
| SUN Z | 2 | 0.066 | LANSU N | 1 | 0.033 |
| SUN YZ | 2 | 0.066 | LANGSTON C | 1 | 0.033 |
| SUN XM | 2 | 0.066 | LANGSTEIN J | 1 | 0.033 |
| SUN XG | 2 | 0.066 | LANGFORD C | 1 | 0.033 |
| SUN WJ | 2 | 0.066 | LANG F | 1 | 0.033 |
| SUN TT | 2 | 0.066 | LANEVE P | 1 | 0.033 |
| SUN SQ | 2 | 0.066 | LANDRY-VOYER AM | 1 | 0.033 |
| SUN SL | 2 | 0.066 | LANDI MT | 1 | 0.033 |
| SUN RF | 2 | 0.066 | LANDERER E | 1 | 0.033 |
| SUN R | 2 | 0.066 | LANDERAS-BUENO S | 1 | 0.033 |
| SUN QZ | 2 | 0.066 | LANDAN G | 1 | 0.033 |
| SUN P | 2 | 0.066 | LAN YZ | 1 | 0.033 |
| SUN N | 2 | 0.066 | LAN YJ | 1 | 0.033 |
| SUN ML | 2 | 0.066 | LAN X | 1 | 0.033 |
| SUN LZ | 2 | 0.066 | LAN WG | 1 | 0.033 |
| SUN LH | 2 | 0.066 | LAN TB | 1 | 0.033 |
| SUN JN | 2 | 0.066 | LAN T | 1 | 0.033 |
| SUN CY | 2 | 0.066 | LAN Q | 1 | 0.033 |
| SUN BD | 2 | 0.066 | LAN FH | 1 | 0.033 |
| SUI SY | 2 | 0.066 | LAN F | 1 | 0.033 |
| SUI H | 2 | 0.066 | LAMBRECHTS D | 1 | 0.033 |
| SUI CJ | 2 | 0.066 | LAMBERT B | 1 | 0.033 |
| SUDO T | 2 | 0.066 | LAMB HJ | 1 | 0.033 |
| SU ZW | 2 | 0.066 | LAM W | 1 | 0.033 |
| SU Z | 2 | 0.066 | LAM S | 1 | 0.033 |
| SU YY | 2 | 0.066 | LAM KF | 1 | 0.033 |
| SU YJ | 2 | 0.066 | LAL A | 1 | 0.033 |
| SU YH | 2 | 0.066 | LAKHANI SR | 1 | 0.033 |
| SU Y | 2 | 0.066 | LAJOIE BR | 1 | 0.033 |
| SU JL | 2 | 0.066 | LAI YX | 1 | 0.033 |
| SU HB | 2 | 0.066 | LAI YS | 1 | 0.033 |
| SU H | 2 | 0.066 | LAI YQ | 1 | 0.033 |
| SU CJ | 2 | 0.066 | LAI YH | 1 | 0.033 |
| STOTZ M | 2 | 0.066 | LAI XB | 1 | 0.033 |
| STOJIC L | 2 | 0.066 | LAI V | 1 | 0.033 |
| STOCKFLETH E | 2 | 0.066 | LAI SY | 1 | 0.033 |
| STEWART GL | 2 | 0.066 | LAI S | 1 | 0.033 |
| STEINMETZ LM | 2 | 0.066 | LAI QW | 1 | 0.033 |
| STANKIEWICZ P | 2 | 0.066 | LAI PBS | 1 | 0.033 |
| SPIZZO R | 2 | 0.066 | LAI NS | 1 | 0.033 |
| SPELEMAN F | 2 | 0.066 | LAI MD | 1 | 0.033 |
| SOUDYAB M | 2 | 0.066 | LAI M | 1 | 0.033 |
| SORENSEN KP | 2 | 0.066 | LAI LC | 1 | 0.033 |
| SONG XY | 2 | 0.066 | LAI KY | 1 | 0.033 |
| SONG XM | 2 | 0.066 | LAI KMV | 1 | 0.033 |
| SONG XJ | 2 | 0.066 | LAI JYC | 1 | 0.033 |
| SONG XD | 2 | 0.066 | LAI JY | 1 | 0.033 |
| SONG WQ | 2 | 0.066 | LAI J | 1 | 0.033 |
| SONG WJ | 2 | 0.066 | LAHESMAA R | 1 | 0.033 |
| SONG SS | 2 | 0.066 | LAHDESMAKI H | 1 | 0.033 |
| SONG P | 2 | 0.066 | LAGOUTTE L | 1 | 0.033 |
| SONG LJ | 2 | 0.066 | LAFONTAINE DLJ | 1 | 0.033 |
| SONG L | 2 | 0.066 | LAFLAMME I | 1 | 0.033 |
| SONG JH | 2 | 0.066 | LAEDERACH A | 1 | 0.033 |
| SONG H | 2 | 0.066 | LACHMEIJER AMA | 1 | 0.033 |
| SONG D | 2 | 0.066 | LACHMAN HM | 1 | 0.033 |
| SONG B | 2 | 0.066 | LABOCHA S | 1 | 0.033 |
| SOLER M | 2 | 0.066 | LABERGE RM | 1 | 0.033 |
| SOLE C | 2 | 0.066 | LABARRIERE N | 1 | 0.033 |
| SOH BS | 2 | 0.066 | LABAFF AM | 1 | 0.033 |
| SLABY O | 2 | 0.066 | LA T | 1 | 0.033 |
| SINGHAL U | 2 | 0.066 | KYSELOVIC J | 1 | 0.033 |
| SINGH P | 2 | 0.066 | KYRIAKOPOULOU A | 1 | 0.033 |
| SINGH KK | 2 | 0.066 | KYBA M | 1 | 0.033 |
| SINGH DK | 2 | 0.066 | KWON OH | 1 | 0.033 |
| SIMARD AR | 2 | 0.066 | KWON JH | 1 | 0.033 |
| SIGOVA AA | 2 | 0.066 | KWON HW | 1 | 0.033 |
| SIGNAL B | 2 | 0.066 | KWOK YKY | 1 | 0.033 |
| SIETZEMA J | 2 | 0.066 | KWOK JBJ | 1 | 0.033 |
| SI XX | 2 | 0.066 | KWELDAM C | 1 | 0.033 |
| SHUAI P | 2 | 0.066 | KWAK LW | 1 | 0.033 |
| SHKUMATAVA A | 2 | 0.066 | KUZNETSOV Y | 1 | 0.033 |
| SHISHKIN AA | 2 | 0.066 | KUZNETSOV VA | 1 | 0.033 |
| SHINDEN Y | 2 | 0.066 | KUZAWA CW | 1 | 0.033 |
| SHIEH TM | 2 | 0.066 | KUWAKO K | 1 | 0.033 |
| SHIBATA K | 2 | 0.066 | KUTTER C | 1 | 0.033 |
| SHI ZM | 2 | 0.066 | KUTCHKO K | 1 | 0.033 |
| SHI ZH | 2 | 0.066 | KUSUNOKI M | 1 | 0.033 |
| SHI ZD | 2 | 0.066 | KUSDIAN G | 1 | 0.033 |
| SHI YY | 2 | 0.066 | KURREEMAN F | 1 | 0.033 |
| SHI YJ | 2 | 0.066 | KURODA J | 1 | 0.033 |
| SHI YB | 2 | 0.066 | KURLANDER E | 1 | 0.033 |
| SHI XL | 2 | 0.066 | KURIMOTO A | 1 | 0.033 |
| SHI R | 2 | 0.066 | KURIAN L | 1 | 0.033 |
| SHI QY | 2 | 0.066 | KURATA N | 1 | 0.033 |
| SHI QX | 2 | 0.066 | KUPPERS R | 1 | 0.033 |
| SHI Q | 2 | 0.066 | KUO YT | 1 | 0.033 |
| SHI M | 2 | 0.066 | KUO SZ | 1 | 0.033 |
| SHI LY | 2 | 0.066 | KUO MD | 1 | 0.033 |
| SHI KH | 2 | 0.066 | KUO KK | 1 | 0.033 |
| SHI JF | 2 | 0.066 | KUO HC | 1 | 0.033 |
| SHI H | 2 | 0.066 | KUO CC | 1 | 0.033 |
| SHI GH | 2 | 0.066 | KUNZE R | 1 | 0.033 |
| SHI FF | 2 | 0.066 | KUNJU LP | 1 | 0.033 |
| SHI FC | 2 | 0.066 | KUNG JTY | 1 | 0.033 |
| SHI DB | 2 | 0.066 | KUNEJ T | 1 | 0.033 |
| SHI CX | 2 | 0.066 | KUNDERFRANCO P | 1 | 0.033 |
| SHI CM | 2 | 0.066 | KUNDAJE A | 1 | 0.033 |
| SHENG XJ | 2 | 0.066 | KUMEGAWA K | 1 | 0.033 |
| SHENDURE J | 2 | 0.066 | KUMARSWARMY R | 1 | 0.033 |
| SHEN YQ | 2 | 0.066 | KUMARASAMY S | 1 | 0.033 |
| SHEN WJ | 2 | 0.066 | KUMAR M | 1 | 0.033 |
| SHEN SY | 2 | 0.066 | KUMAR DBU | 1 | 0.033 |
| SHEN SQ | 2 | 0.066 | KUMAKURA S | 1 | 0.033 |
| SHEN SP | 2 | 0.066 | KUMAKURA M | 1 | 0.033 |
| SHEN QW | 2 | 0.066 | KULLANDER K | 1 | 0.033 |
| SHEN N | 2 | 0.066 | KULCZYNSKA K | 1 | 0.033 |
| SHEN M | 2 | 0.066 | KUIPER EG | 1 | 0.033 |
| SHEN GQ | 2 | 0.066 | KUINTZLE R | 1 | 0.033 |
| SHEN BY | 2 | 0.066 | KUHN CD | 1 | 0.033 |
| SHEN BR | 2 | 0.066 | KUH HJ | 1 | 0.033 |
| SHARMA S | 2 | 0.066 | KUGOH H | 1 | 0.033 |
| SHAO K | 2 | 0.066 | KUGEL JF | 1 | 0.033 |
| SHAO CX | 2 | 0.066 | KUERBAN M | 1 | 0.033 |
| SHAN WW | 2 | 0.066 | KUDO H | 1 | 0.033 |
| SHAHRYARI V | 2 | 0.066 | KUCINSKI J | 1 | 0.033 |
| SHAFIEE M | 2 | 0.066 | KUCHEROV V | 1 | 0.033 |
| SHA YH | 2 | 0.066 | KUBBEN N | 1 | 0.033 |
| SHA N | 2 | 0.066 | KUANG Y | 1 | 0.033 |
| SEOL MY | 2 | 0.066 | KUANG W | 1 | 0.033 |
| SEN P | 2 | 0.066 | KUANG DW | 1 | 0.033 |
| SELLERS TA | 2 | 0.066 | KUANG B | 1 | 0.033 |
| SEHGAL L | 2 | 0.066 | KU JL | 1 | 0.033 |
| SCHWARZENBACHER D | 2 | 0.066 | KSIAZEK K | 1 | 0.033 |
| SCHWARZ TM | 2 | 0.066 | KRZYSZTON M | 1 | 0.033 |
| SCHROTH GP | 2 | 0.066 | KRUMM A | 1 | 0.033 |
| SCHREIBER S | 2 | 0.066 | KRUHLAK M | 1 | 0.033 |
| SCHMITT AM | 2 | 0.066 | KRUEGER JM | 1 | 0.033 |
| SCHMIDT D | 2 | 0.066 | KRUEGER C | 1 | 0.033 |
| SCHIER AF | 2 | 0.066 | KRONENBERG Z | 1 | 0.033 |
| SCHEUERMANN JC | 2 | 0.066 | KROL J | 1 | 0.033 |
| SCHAUERTE C | 2 | 0.066 | KROL I | 1 | 0.033 |
| SCANTLAND S | 2 | 0.066 | KROESE FGM | 1 | 0.033 |
| SCACHERI PC | 2 | 0.066 | KROEMER G | 1 | 0.033 |
| SATPATHY AT | 2 | 0.066 | KRISHNAN J | 1 | 0.033 |
| SARKAR MK | 2 | 0.066 | KREUZ M | 1 | 0.033 |
| SANTORO R | 2 | 0.066 | KRETZMER H | 1 | 0.033 |
| SANTORO C | 2 | 0.066 | KRESOJA-RAKIC J | 1 | 0.033 |
| SANTINI T | 2 | 0.066 | KREMPF M | 1 | 0.033 |
| SANSOM SN | 2 | 0.066 | KREGEL S | 1 | 0.033 |
| SANG XT | 2 | 0.066 | KREFT SG | 1 | 0.033 |
| SANDOVAL J | 2 | 0.066 | KRAUSE MN | 1 | 0.033 |
| SAND M | 2 | 0.066 | KRAUSE HM | 1 | 0.033 |
| SAND D | 2 | 0.066 | KRATCHMAROV R | 1 | 0.033 |
| SANCHEZ-GOMEZ DB | 2 | 0.066 | KRALOVICS R | 1 | 0.033 |
| SAMANIEGO F | 2 | 0.066 | KRAJEWSKI K | 1 | 0.033 |
| SALVATORI B | 2 | 0.066 | KRAINER AR | 1 | 0.033 |
| SALEM M | 2 | 0.066 | KOZUKA-HATA H | 1 | 0.033 |
| SAKIMURA S | 2 | 0.066 | KOZIOL M | 1 | 0.033 |
| SAITO T | 2 | 0.066 | KOZICZ T | 1 | 0.033 |
| SAIDY NRN | 2 | 0.066 | KOWALCZYK MS | 1 | 0.033 |
| SAFE S | 2 | 0.066 | KOVACS J | 1 | 0.033 |
| SAETROM P | 2 | 0.066 | KOVAC M | 1 | 0.033 |
| SADEGHIZADEH M | 2 | 0.066 | KOUFARIOTIS LT | 1 | 0.033 |
| SACHIDANANDAN C | 2 | 0.066 | KOU L | 1 | 0.033 |
| SABETI PC | 2 | 0.066 | KOTZIN JJ | 1 | 0.033 |
| SAAYMAN SM | 2 | 0.066 | KOTLINOWSKI J | 1 | 0.033 |
| RUI YJ | 2 | 0.066 | KOTHARI V | 1 | 0.033 |
| RUI Q | 2 | 0.066 | KOTELIANSKY V | 1 | 0.033 |
| RUI K | 2 | 0.066 | KOTELEVTSEV YV | 1 | 0.033 |
| RUBIN MA | 2 | 0.066 | KOTB YM | 1 | 0.033 |
| RUAN ZB | 2 | 0.066 | KOTANI T | 1 | 0.033 |
| ROY S | 2 | 0.066 | KOSTOULAS N | 1 | 0.033 |
| ROWLEY MJ | 2 | 0.066 | KOSTADINOVA L | 1 | 0.033 |
| ROUGEULLE C | 2 | 0.066 | KOSSAI M | 1 | 0.033 |
| ROSENFELD MG | 2 | 0.066 | KOSMYNA B | 1 | 0.033 |
| RODRIGUEZ-MALAVE NI | 2 | 0.066 | KOSIR MA | 1 | 0.033 |
| RODRIGUEZ-AGUAYO C | 2 | 0.066 | KOSIK KS | 1 | 0.033 |
| ROBINSON DR | 2 | 0.066 | KOSALAI ST | 1 | 0.033 |
| ROBERTS TC | 2 | 0.066 | KORTH MJ | 1 | 0.033 |
| ROBERT C | 2 | 0.066 | KORSWAGEN HC | 1 | 0.033 |
| RISCH A | 2 | 0.066 | KORNMANN M | 1 | 0.033 |
| RICHARDS EJ | 2 | 0.066 | KORNFELD JW | 1 | 0.033 |
| RICCI EP | 2 | 0.066 | KORNER C | 1 | 0.033 |
| RIBERDY JM | 2 | 0.066 | KORKMAZ G | 1 | 0.033 |
| RIBACKE U | 2 | 0.066 | KOPPSTEIN D | 1 | 0.033 |
| REN YL | 2 | 0.066 | KOOK H | 1 | 0.033 |
| REN XM | 2 | 0.066 | KOO M | 1 | 0.033 |
| REN XB | 2 | 0.066 | KONUTHULA N | 1 | 0.033 |
| REN SL | 2 | 0.066 | KONO T | 1 | 0.033 |
| REN JY | 2 | 0.066 | KONISHI H | 1 | 0.033 |
| REN JS | 2 | 0.066 | KONG Z | 1 | 0.033 |
| REN J | 2 | 0.066 | KONG XZ | 1 | 0.033 |
| REN HX | 2 | 0.066 | KONG XP | 1 | 0.033 |
| REN CC | 2 | 0.066 | KONG QP | 1 | 0.033 |
| REINIUS B | 2 | 0.066 | KONG LP | 1 | 0.033 |
| REILLY MP | 2 | 0.066 | KONG JY | 1 | 0.033 |
| REID G | 2 | 0.066 | KONG JL | 1 | 0.033 |
| REICHE K | 2 | 0.066 | KONG J | 1 | 0.033 |
| REGAZZI R | 2 | 0.066 | KONG H | 1 | 0.033 |
| REDRUP L | 2 | 0.066 | KONG GY | 1 | 0.033 |
| REDIS RS | 2 | 0.066 | KONG FQ | 1 | 0.033 |
| RECIO L | 2 | 0.066 | KONG F | 1 | 0.033 |
| RECHAVI G | 2 | 0.066 | KONG CZ | 1 | 0.033 |
| RAVEH E | 2 | 0.066 | KONDO K | 1 | 0.033 |
| RATNU VS | 2 | 0.066 | KOMUNE S | 1 | 0.033 |
| RAO SQ | 2 | 0.066 | KOMOTAR R | 1 | 0.033 |
| RAO MRS | 2 | 0.066 | KOMIYA R | 1 | 0.033 |
| RAO DS | 2 | 0.066 | KOMIYA M | 1 | 0.033 |
| RANA TM | 2 | 0.066 | KOMINSKY HD | 1 | 0.033 |
| RAMSAY L | 2 | 0.066 | KOMATSU S | 1 | 0.033 |
| QUAN A | 2 | 0.066 | KOMATSU M | 1 | 0.033 |
| QUAGLIATA L | 2 | 0.066 | KOLONIN MG | 1 | 0.033 |
| QU Z | 2 | 0.066 | KOLODZIEJSKA KE | 1 | 0.033 |
| QU Y | 2 | 0.066 | KOLLING M | 1 | 0.033 |
| QU J | 2 | 0.066 | KOLITZ JE | 1 | 0.033 |
| QIU YZ | 2 | 0.066 | KOLEVZON N | 1 | 0.033 |
| QIU JX | 2 | 0.066 | KOKUSHO R | 1 | 0.033 |
| QIN Y | 2 | 0.066 | KOKUDO N | 1 | 0.033 |
| QIN R | 2 | 0.066 | KOJETIN DJ | 1 | 0.033 |
| QIN Q | 2 | 0.066 | KOIRALA P | 1 | 0.033 |
| QIN J | 2 | 0.066 | KOINUMA D | 1 | 0.033 |
| QIN CZ | 2 | 0.066 | KOHVAKKA A | 1 | 0.033 |
| QIAO L | 2 | 0.066 | KOHLSCHMIDT J | 1 | 0.033 |
| QIAO HQ | 2 | 0.066 | KOGNER P | 1 | 0.033 |
| QIAN TM | 2 | 0.066 | KOGANTI PP | 1 | 0.033 |
| QIAN KQ | 2 | 0.066 | KOERNER MV | 1 | 0.033 |
| QIAN GX | 2 | 0.066 | KOENIG RJ | 1 | 0.033 |
| QI Y | 2 | 0.066 | KOENEN KC | 1 | 0.033 |
| QI XW | 2 | 0.066 | KODAMA T | 1 | 0.033 |
| QI WC | 2 | 0.066 | KOCH I | 1 | 0.033 |
| QI L | 2 | 0.066 | KOCH C | 1 | 0.033 |
| QI HX | 2 | 0.066 | KOBERSTEIN JN | 1 | 0.033 |
| PYLE AM | 2 | 0.066 | KOBAYASHI T | 1 | 0.033 |
| PURCELL S | 2 | 0.066 | KOBAYASHI R | 1 | 0.033 |
| PU XY | 2 | 0.066 | KOBAYASHI M | 1 | 0.033 |
| PU H | 2 | 0.066 | KOBAYASHI H | 1 | 0.033 |
| PRIOR C | 2 | 0.066 | KO SB | 1 | 0.033 |
| PRESTON B | 2 | 0.066 | KNOWLES DG | 1 | 0.033 |
| POSAS F | 2 | 0.066 | KNAUSS JL | 1 | 0.033 |
| PONOMAREVA Y | 2 | 0.066 | KNAPP M | 1 | 0.033 |
| PONJAVIC J | 2 | 0.066 | KLUSMANN JH | 1 | 0.033 |
| PONDICK JV | 2 | 0.066 | KLOPP C | 1 | 0.033 |
| POLIAKOV A | 2 | 0.066 | KLION AD | 1 | 0.033 |
| POCIOT F | 2 | 0.066 | KLIBANSKI A | 1 | 0.033 |
| PLATH K | 2 | 0.066 | KLEO K | 1 | 0.033 |
| PLASS C | 2 | 0.066 | KLEMENT RM | 1 | 0.033 |
| PIKOR L | 2 | 0.066 | KLEIVERDA G | 1 | 0.033 |
| PIERRON G | 2 | 0.066 | KLEINOVA R | 1 | 0.033 |
| PHILLIPS SL | 2 | 0.066 | KLEINMAN CL | 1 | 0.033 |
| PFANNE A | 2 | 0.066 | KLEINER DE | 1 | 0.033 |
| PEZZUTO I | 2 | 0.066 | KLEINBRINK EL | 1 | 0.033 |
| PETROSINO G | 2 | 0.066 | KLEIN RJ | 1 | 0.033 |
| PETERS T | 2 | 0.066 | KLEIN DC | 1 | 0.033 |
| PERSONS DA | 2 | 0.066 | KLEEFF J | 1 | 0.033 |
| PERSICHETTI F | 2 | 0.066 | KLEBE S | 1 | 0.033 |
| PERNER S | 2 | 0.066 | KLATTENHOFF CA | 1 | 0.033 |
| PERMUTH-WEY J | 2 | 0.066 | KLAPPER W | 1 | 0.033 |
| PERKINS AC | 2 | 0.066 | KLAPPER M | 1 | 0.033 |
| PERINI G | 2 | 0.066 | KLADI-SKANDALI A | 1 | 0.033 |
| PERFETTI A | 2 | 0.066 | KJELDSEN AD | 1 | 0.033 |
| PENG ZY | 2 | 0.066 | KIVISAARI A | 1 | 0.033 |
| PENG XX | 2 | 0.066 | KIVINUMMI K | 1 | 0.033 |
| PENG XL | 2 | 0.066 | KITSAO BS | 1 | 0.033 |
| PENG X | 2 | 0.066 | KITOW J | 1 | 0.033 |
| PENG T | 2 | 0.066 | KITAMOTO T | 1 | 0.033 |
| PENG P | 2 | 0.066 | KITAJIMA H | 1 | 0.033 |
| PENG LP | 2 | 0.066 | KITABAYASHI N | 1 | 0.033 |
| PENG J | 2 | 0.066 | KISHORE S | 1 | 0.033 |
| PENG HY | 2 | 0.066 | KIRKEGAARD K | 1 | 0.033 |
| PENG H | 2 | 0.066 | KIPPS TJ | 1 | 0.033 |
| PENG BY | 2 | 0.066 | KINZIG CG | 1 | 0.033 |
| PEI DQ | 2 | 0.066 | KINNEBREW G | 1 | 0.033 |
| PEGUEROLES C | 2 | 0.066 | KING MJ | 1 | 0.033 |
| PAULI A | 2 | 0.066 | KIMURA H | 1 | 0.033 |
| PATOWARY A | 2 | 0.066 | KIM YS | 1 | 0.033 |
| PATEL L | 2 | 0.066 | KIM YH | 1 | 0.033 |
| PASQUIER E | 2 | 0.066 | KIM WW | 1 | 0.033 |
| PARK DK | 2 | 0.066 | KIM WS | 1 | 0.033 |
| PARK D | 2 | 0.066 | KIM WK | 1 | 0.033 |
| PARISIEN M | 2 | 0.066 | KIM VN | 1 | 0.033 |
| PANERU B | 2 | 0.066 | KIM TS | 1 | 0.033 |
| PANDYA-JONES A | 2 | 0.066 | KIM TJ | 1 | 0.033 |
| PANDA AC | 2 | 0.066 | KIM TH | 1 | 0.033 |
| PAN YQ | 2 | 0.066 | KIM SY | 1 | 0.033 |
| PAN YF | 2 | 0.066 | KIM SV | 1 | 0.033 |
| PAN XL | 2 | 0.066 | KIM ST | 1 | 0.033 |
| PAN X | 2 | 0.066 | KIM SK | 1 | 0.033 |
| PAN SH | 2 | 0.066 | KIM S | 1 | 0.033 |
| PAN QH | 2 | 0.066 | KIM MY | 1 | 0.033 |
| PAN JY | 2 | 0.066 | KIM LK | 1 | 0.033 |
| PAN HF | 2 | 0.066 | KIM KY | 1 | 0.033 |
| PAN CF | 2 | 0.066 | KIM KS | 1 | 0.033 |
| PALANISAMY N | 2 | 0.066 | KIM KH | 1 | 0.033 |
| PACI P | 2 | 0.066 | KIM IH | 1 | 0.033 |
| OUYANG Y | 2 | 0.066 | KIM HP | 1 | 0.033 |
| OLSON EN | 2 | 0.066 | KIM H | 1 | 0.033 |
| OKADA-HATAKEYAMA M | 2 | 0.066 | KIM EZ | 1 | 0.033 |
| OHTA K | 2 | 0.066 | KIM ED | 1 | 0.033 |
| ODOM DT | 2 | 0.066 | KIM E | 1 | 0.033 |
| NTINI E | 2 | 0.066 | KIM DS | 1 | 0.033 |
| NORRIS MD | 2 | 0.066 | KIM CR | 1 | 0.033 |
| NOBLE WS | 2 | 0.066 | KIM C | 1 | 0.033 |
| NIU W | 2 | 0.066 | KIM BM | 1 | 0.033 |
| NIEMCZYK M | 2 | 0.066 | KIM BG | 1 | 0.033 |
| NIE Y | 2 | 0.066 | KILEDJIAN M | 1 | 0.033 |
| NIE W | 2 | 0.066 | KIKYO N | 1 | 0.033 |
| NI LX | 2 | 0.066 | KIESSLICH T | 1 | 0.033 |
| NI CH | 2 | 0.066 | KIELSTEIN T | 1 | 0.033 |
| NELSON BR | 2 | 0.066 | KIEFER J | 1 | 0.033 |
| NEGISHI L | 2 | 0.066 | KHURANA S | 1 | 0.033 |
| NECSULEA A | 2 | 0.066 | KHOURY N | 1 | 0.033 |
| NAMBARA S | 2 | 0.066 | KHOSROHEIDARI M | 1 | 0.033 |
| NAM EJ | 2 | 0.066 | KHOSHNEVISAN A | 1 | 0.033 |
| NAKAYAMA Y | 2 | 0.066 | KHOSHAB N | 1 | 0.033 |
| NAKAMURA Y | 2 | 0.066 | KHOR CC | 1 | 0.033 |
| NAKAGAWA T | 2 | 0.066 | KHODADADI-JAMAYRAN A | 1 | 0.033 |
| NAGY R | 2 | 0.066 | KHEDR G | 1 | 0.033 |
| NAGASHIMA T | 2 | 0.066 | KHAVARI P | 1 | 0.033 |
| NAGANUMA T | 2 | 0.066 | KHATUN J | 1 | 0.033 |
| NADAL-RIBELLES M | 2 | 0.066 | KHATIB H | 1 | 0.033 |
| MUYS BR | 2 | 0.066 | KHASHAB T | 1 | 0.033 |
| MURRELL A | 2 | 0.066 | KHASHAB M | 1 | 0.033 |
| MUNSON G | 2 | 0.066 | KHANDWALLA I | 1 | 0.033 |
| MULLER SC | 2 | 0.066 | KHANDELWAL A | 1 | 0.033 |
| MULLER F | 2 | 0.066 | KHAN MR | 1 | 0.033 |
| MUELLER M | 2 | 0.066 | KHAN J | 1 | 0.033 |
| MU JS | 2 | 0.066 | KHALI AM | 1 | 0.033 |
| MOURTADA-MAARABOUNI M | 2 | 0.066 | KHAITOVICH P | 1 | 0.033 |
| MOTTERLE A | 2 | 0.066 | KHAITAN D | 1 | 0.033 |
| MORTON CC | 2 | 0.066 | KHACHANE AN | 1 | 0.033 |
| MORSE M | 2 | 0.066 | KERY RE | 1 | 0.033 |
| MORLEY MP | 2 | 0.066 | KEPP O | 1 | 0.033 |
| MORILLON A | 2 | 0.066 | KENZELMANN-BROZ D | 1 | 0.033 |
| MORI Y | 2 | 0.066 | KENSLER TW | 1 | 0.033 |
| MORI M | 2 | 0.066 | KENNEWEG F | 1 | 0.033 |
| MOORE MJ | 2 | 0.066 | KENNETT JY | 1 | 0.033 |
| MOON J | 2 | 0.066 | KENNEDY PJ | 1 | 0.033 |
| MONTONE K | 2 | 0.066 | KENIRY A | 1 | 0.033 |
| MONTES M | 2 | 0.066 | KEMENY L | 1 | 0.033 |
| MONNIER P | 2 | 0.066 | KELLIE S | 1 | 0.033 |
| MONDAL NR | 2 | 0.066 | KELLEY DR | 1 | 0.033 |
| MOLNAR Z | 2 | 0.066 | KELLEY D | 1 | 0.033 |
| MOLLOY PL | 2 | 0.066 | KELLEHER RJ | 1 | 0.033 |
| MOHAMMAD F | 2 | 0.066 | KEIGHTLEY PD | 1 | 0.033 |
| MITRA S | 2 | 0.066 | KEBEBEW E | 1 | 0.033 |
| MISTELI T | 2 | 0.066 | KE XQ | 1 | 0.033 |
| MIRZA AH | 2 | 0.066 | KE PQ | 1 | 0.033 |
| MICHELETTI R | 2 | 0.066 | KE J | 1 | 0.033 |
| MIAO XY | 2 | 0.066 | KE CW | 1 | 0.033 |
| MIAO RZ | 2 | 0.066 | KAZEMZADEH M | 1 | 0.033 |
| MIAO JY | 2 | 0.066 | KAZAMA S | 1 | 0.033 |
| MENTEN B | 2 | 0.066 | KAZADI D | 1 | 0.033 |
| MENG X | 2 | 0.066 | KAYABASI C | 1 | 0.033 |
| MENG LY | 2 | 0.066 | KAWAOKA Y | 1 | 0.033 |
| MENG J | 2 | 0.066 | KAWAMURA S | 1 | 0.033 |
| MENG F | 2 | 0.066 | KAWAHARA K | 1 | 0.033 |
| MELTZER SJ | 2 | 0.066 | KAWAGUCHI Y | 1 | 0.033 |
| MEI YD | 2 | 0.066 | KAWAGUCHI A | 1 | 0.033 |
| MEI Q | 2 | 0.066 | KAVALLARIS M | 1 | 0.033 |
| MEI HB | 2 | 0.066 | KAVALALI ET | 1 | 0.033 |
| MEHRA R | 2 | 0.066 | KAV NNV | 1 | 0.033 |
| MCMAHON S | 2 | 0.066 | KAUR S | 1 | 0.033 |
| MCDONEL P | 2 | 0.066 | KAUR P | 1 | 0.033 |
| MCDONALD RA | 2 | 0.066 | KAUFMAN D | 1 | 0.033 |
| MCANALLY JR | 2 | 0.066 | KATZ M | 1 | 0.033 |
| MATOUK IJ | 2 | 0.066 | KATSUMA S | 1 | 0.033 |
| MARTENS L | 2 | 0.066 | KATSAROU K | 1 | 0.033 |
| MARTELLI F | 2 | 0.066 | KATO M | 1 | 0.033 |
| MAO ZB | 2 | 0.066 | KATO A | 1 | 0.033 |
| MAO Y | 2 | 0.066 | KATHREIN K | 1 | 0.033 |
| MAO QX | 2 | 0.066 | KATAYAMA S | 1 | 0.033 |
| MAO M | 2 | 0.066 | KATARUKA S | 1 | 0.033 |
| MAO HL | 2 | 0.066 | KATAHIRA M | 1 | 0.033 |
| MAO H | 2 | 0.066 | KASIRI S | 1 | 0.033 |
| MALEK E | 2 | 0.066 | KASHIMA L | 1 | 0.033 |
| MAKAREWICH CA | 2 | 0.066 | KASHI K | 1 | 0.033 |
| MAHER CA | 2 | 0.066 | KASARAGOD P | 1 | 0.033 |
| MAGER J | 2 | 0.066 | KASACK K | 1 | 0.033 |
| MAENNER S | 2 | 0.066 | KARTASALO K | 1 | 0.033 |
| MACLARY E | 2 | 0.066 | KARSCH D | 1 | 0.033 |
| MACINNES AW | 2 | 0.066 | KARPOV VL | 1 | 0.033 |
| MA YL | 2 | 0.066 | KAROLINA DS | 1 | 0.033 |
| MA YJ | 2 | 0.066 | KARNI R | 1 | 0.033 |
| MA YH | 2 | 0.066 | KARNES RJ | 1 | 0.033 |
| MA YF | 2 | 0.066 | KARNER H | 1 | 0.033 |
| MA XY | 2 | 0.066 | KARLSSON O | 1 | 0.033 |
| MA XP | 2 | 0.066 | KARASTERGIOU K | 1 | 0.033 |
| MA WX | 2 | 0.066 | KARAKURT S | 1 | 0.033 |
| MA WK | 2 | 0.066 | KAPSETAKI M | 1 | 0.033 |
| MA SL | 2 | 0.066 | KAPPEI D | 1 | 0.033 |
| MA SB | 2 | 0.066 | KAPITANOVIC S | 1 | 0.033 |
| MA RM | 2 | 0.066 | KAO SC | 1 | 0.033 |
| MA QF | 2 | 0.066 | KANTOFF PW | 1 | 0.033 |
| MA LN | 2 | 0.066 | KANTOFF P | 1 | 0.033 |
| MA LF | 2 | 0.066 | KANTO T | 1 | 0.033 |
| MA JH | 2 | 0.066 | KANG YN | 1 | 0.033 |
| MA HX | 2 | 0.066 | KANG YH | 1 | 0.033 |
| MA F | 2 | 0.066 | KANG XX | 1 | 0.033 |
| MA CH | 2 | 0.066 | KANG XD | 1 | 0.033 |
| MA CC | 2 | 0.066 | KANG TW | 1 | 0.033 |
| MA B | 2 | 0.066 | KANG SL | 1 | 0.033 |
| LV ZJ | 2 | 0.066 | KANG SH | 1 | 0.033 |
| LV XW | 2 | 0.066 | KANG S | 1 | 0.033 |
| LV WJ | 2 | 0.066 | KANG QZ | 1 | 0.033 |
| LV HZ | 2 | 0.066 | KANG Q | 1 | 0.033 |
| LV CJ | 2 | 0.066 | KANG MQ | 1 | 0.033 |
| LUO ZH | 2 | 0.066 | KANG MJ | 1 | 0.033 |
| LUO YH | 2 | 0.066 | KANG M | 1 | 0.033 |
| LUO QM | 2 | 0.066 | KANG L | 1 | 0.033 |
| LUO Q | 2 | 0.066 | KANG HM | 1 | 0.033 |
| LUO L | 2 | 0.066 | KANG GL | 1 | 0.033 |
| LUO G | 2 | 0.066 | KANG F | 1 | 0.033 |
| LUO DK | 2 | 0.066 | KANG DM | 1 | 0.033 |
| LUNTER G | 2 | 0.066 | KANG D | 1 | 0.033 |
| LUK ACS | 2 | 0.066 | KANG CY | 1 | 0.033 |
| LUAN WK | 2 | 0.066 | KANEKI K | 1 | 0.033 |
| LUAN J | 2 | 0.066 | KANE M | 1 | 0.033 |
| LU ZL | 2 | 0.066 | KANDURI K | 1 | 0.033 |
| LU ZH | 2 | 0.066 | KANDALAFT LE | 1 | 0.033 |
| LU YX | 2 | 0.066 | KAN YS | 1 | 0.033 |
| LU YL | 2 | 0.066 | KAN X | 1 | 0.033 |
| LU XH | 2 | 0.066 | KAN HP | 1 | 0.033 |
| LU XB | 2 | 0.066 | KAMEL MM | 1 | 0.033 |
| LU WP | 2 | 0.066 | KAMEH D | 1 | 0.033 |
| LU TP | 2 | 0.066 | KAMEDA T | 1 | 0.033 |
| LU SW | 2 | 0.066 | KAMBARA H | 1 | 0.033 |
| LU SH | 2 | 0.066 | KAMATANI N | 1 | 0.033 |
| LU M | 2 | 0.066 | KAM Y | 1 | 0.033 |
| LU LW | 2 | 0.066 | KALWA M | 1 | 0.033 |
| LU JP | 2 | 0.066 | KALLURI R | 1 | 0.033 |
| LU JJ | 2 | 0.066 | KALLIN EM | 1 | 0.033 |
| LU JB | 2 | 0.066 | KALLER M | 1 | 0.033 |
| LU HM | 2 | 0.066 | KALLEN AN | 1 | 0.033 |
| LU H | 2 | 0.066 | KALLAJOKI M | 1 | 0.033 |
| LOPEZ-BERESTEIN G | 2 | 0.066 | KALITA CA | 1 | 0.033 |
| LOPEZ C | 2 | 0.066 | KALIL J | 1 | 0.033 |
| LONG XC | 2 | 0.066 | KALEV P | 1 | 0.033 |
| LONG W | 2 | 0.066 | KALARI KR | 1 | 0.033 |
| LONERGAN KM | 2 | 0.066 | KALANTIDIS K | 1 | 0.033 |
| LOGOTHETIS CJ | 2 | 0.066 | KALA Z | 1 | 0.033 |
| LO KA | 2 | 0.066 | KAJIHARA I | 1 | 0.033 |
| LIZ J | 2 | 0.066 | KAIPAINEN A | 1 | 0.033 |
| LIU ZK | 2 | 0.066 | KAI M | 1 | 0.033 |
| LIU ZC | 2 | 0.066 | KAI C | 1 | 0.033 |
| LIU YS | 2 | 0.066 | KAHL G | 1 | 0.033 |
| LIU YR | 2 | 0.066 | KAHARI VM | 1 | 0.033 |
| LIU YQ | 2 | 0.066 | KAGAN S | 1 | 0.033 |
| LIU YN | 2 | 0.066 | KAECH A | 1 | 0.033 |
| LIU XZ | 2 | 0.066 | KADAKKUZHA BM | 1 | 0.033 |
| LIU XQ | 2 | 0.066 | KACZOROWSKI DC | 1 | 0.033 |
| LIU XJ | 2 | 0.066 | JUTOORU I | 1 | 0.033 |
| LIU WL | 2 | 0.066 | JURACEK J | 1 | 0.033 |
| LIU TY | 2 | 0.066 | JURA J | 1 | 0.033 |
| LIU TC | 2 | 0.066 | JUNG SG | 1 | 0.033 |
| LIU SR | 2 | 0.066 | JUNG JW | 1 | 0.033 |
| LIU SP | 2 | 0.066 | JUNG JH | 1 | 0.033 |
| LIU SN | 2 | 0.066 | JUNG H | 1 | 0.033 |
| LIU SL | 2 | 0.066 | JU XB | 1 | 0.033 |
| LIU SC | 2 | 0.066 | JU W | 1 | 0.033 |
| LIU RL | 2 | 0.066 | JU SQ | 1 | 0.033 |
| LIU P | 2 | 0.066 | JU L | 1 | 0.033 |
| LIU MX | 2 | 0.066 | JU HQ | 1 | 0.033 |
| LIU MM | 2 | 0.066 | JU DH | 1 | 0.033 |
| LIU LP | 2 | 0.066 | JOYE E | 1 | 0.033 |
| LIU LJ | 2 | 0.066 | JOYCE CE | 1 | 0.033 |
| LIU JZ | 2 | 0.066 | JOUSSEN S | 1 | 0.033 |
| LIU JM | 2 | 0.066 | JOURD'HEUIL D | 1 | 0.033 |
| LIU HZ | 2 | 0.066 | JOTHI M | 1 | 0.033 |
| LIU HC | 2 | 0.066 | JOSSET L | 1 | 0.033 |
| LIU GJ | 2 | 0.066 | JOSIPOVIC I | 1 | 0.033 |
| LIU GF | 2 | 0.066 | JOSHUA-TOR L | 1 | 0.033 |
| LIU EL | 2 | 0.066 | JOSHI RK | 1 | 0.033 |
| LIU DQ | 2 | 0.066 | JOSHI N | 1 | 0.033 |
| LIU DL | 2 | 0.066 | JOSHI K | 1 | 0.033 |
| LIU D | 2 | 0.066 | JOSH P | 1 | 0.033 |
| LIU CK | 2 | 0.066 | JOSEPH N | 1 | 0.033 |
| LIU CH | 2 | 0.066 | JORGENSEN TE | 1 | 0.033 |
| LIU BB | 2 | 0.066 | JORGE OC | 1 | 0.033 |
| LIN YS | 2 | 0.066 | JOPLING CL | 1 | 0.033 |
| LIN YM | 2 | 0.066 | JOO JH | 1 | 0.033 |
| LIN YD | 2 | 0.066 | JONSSON P | 1 | 0.033 |
| LIN XC | 2 | 0.066 | JONG RM | 1 | 0.033 |
| LIN WC | 2 | 0.066 | JONES SW | 1 | 0.033 |
| LIN TX | 2 | 0.066 | JONES SJM | 1 | 0.033 |
| LIN Q | 2 | 0.066 | JONES MH | 1 | 0.033 |
| LIN NW | 2 | 0.066 | JOHNSTON RL | 1 | 0.033 |
| LIN MJ | 2 | 0.066 | JOHNSTON D | 1 | 0.033 |
| LIN KY | 2 | 0.066 | JOHNSON JS | 1 | 0.033 |
| LIN JJ | 2 | 0.066 | JOHNSON GS | 1 | 0.033 |
| LIN JD | 2 | 0.066 | JOHNSON AM | 1 | 0.033 |
| LIN HY | 2 | 0.066 | JOHNSEN VL | 1 | 0.033 |
| LIN H | 2 | 0.066 | JOHNSEN HE | 1 | 0.033 |
| LIN CC | 2 | 0.066 | JOHN D | 1 | 0.033 |
| LIAPIS SC | 2 | 0.066 | JOHANSEN TEB | 1 | 0.033 |
| LIAO JY | 2 | 0.066 | JOHANSEN SD | 1 | 0.033 |
| LIANG ZC | 2 | 0.066 | JOHANNES GJ | 1 | 0.033 |
| LIANG YD | 2 | 0.066 | JOE B | 1 | 0.033 |
| LIANG WC | 2 | 0.066 | JO MJ | 1 | 0.033 |
| LIANG K | 2 | 0.066 | JNBAPTISTE CK | 1 | 0.033 |
| LIANG J | 2 | 0.066 | JING YZ | 1 | 0.033 |
| LIANG CZ | 2 | 0.066 | JING YF | 1 | 0.033 |
| LIAN Y | 2 | 0.066 | JING XY | 1 | 0.033 |
| LI ZZ | 2 | 0.066 | JING X | 1 | 0.033 |
| LI ZP | 2 | 0.066 | JING SY | 1 | 0.033 |
| LI ZM | 2 | 0.066 | JING R | 1 | 0.033 |
| LI ZL | 2 | 0.066 | JING Q | 1 | 0.033 |
| LI ZF | 2 | 0.066 | JING LL | 1 | 0.033 |
| LI ZC | 2 | 0.066 | JING L | 1 | 0.033 |
| LI YY | 2 | 0.066 | JING FM | 1 | 0.033 |
| LI YN | 2 | 0.066 | JING DD | 1 | 0.033 |
| LI YD | 2 | 0.066 | JIN ZJ | 1 | 0.033 |
| LI XZ | 2 | 0.066 | JIN Z | 1 | 0.033 |
| LI XT | 2 | 0.066 | JIN YY | 1 | 0.033 |
| LI XG | 2 | 0.066 | JIN YG | 1 | 0.033 |
| LI XC | 2 | 0.066 | JIN XB | 1 | 0.033 |
| LI WY | 2 | 0.066 | JIN WB | 1 | 0.033 |
| LI WR | 2 | 0.066 | JIN UH | 1 | 0.033 |
| LI WP | 2 | 0.066 | JIN SD | 1 | 0.033 |
| LI WH | 2 | 0.066 | JIN S | 1 | 0.033 |
| LI WF | 2 | 0.066 | JIN QQ | 1 | 0.033 |
| LI TQ | 2 | 0.066 | JIN QH | 1 | 0.033 |
| LI TM | 2 | 0.066 | JIN Q | 1 | 0.033 |
| LI SW | 2 | 0.066 | JIN MW | 1 | 0.033 |
| LI SN | 2 | 0.066 | JIN MF | 1 | 0.033 |
| LI SG | 2 | 0.066 | JIN LX | 1 | 0.033 |
| LI QY | 2 | 0.066 | JIN LG | 1 | 0.033 |
| LI QF | 2 | 0.066 | JIN KP | 1 | 0.033 |
| LI QE | 2 | 0.066 | JIN KK | 1 | 0.033 |
| LI PD | 2 | 0.066 | JIN JJ | 1 | 0.033 |
| LI MH | 2 | 0.066 | JIN HF | 1 | 0.033 |
| LI MC | 2 | 0.066 | JIN GH | 1 | 0.033 |
| LI LH | 2 | 0.066 | JIN GF | 1 | 0.033 |
| LI JS | 2 | 0.066 | JIN DY | 1 | 0.033 |
| LI JR | 2 | 0.066 | JIN CS | 1 | 0.033 |
| LI JL | 2 | 0.066 | JIN CJ | 1 | 0.033 |
| LI HT | 2 | 0.066 | JIMENEZ B | 1 | 0.033 |
| LI HQ | 2 | 0.066 | JIE WX | 1 | 0.033 |
| LI HM | 2 | 0.066 | JIE J | 1 | 0.033 |
| LI HL | 2 | 0.066 | JIE HY | 1 | 0.033 |
| LI HJ | 2 | 0.066 | JIE D | 1 | 0.033 |
| LI HF | 2 | 0.066 | JICKLING GC | 1 | 0.033 |
| LI GN | 2 | 0.066 | JIAO YM | 1 | 0.033 |
| LI GM | 2 | 0.066 | JIAO WQ | 1 | 0.033 |
| LI FF | 2 | 0.066 | JIAO TT | 1 | 0.033 |
| LI E | 2 | 0.066 | JIAO TL | 1 | 0.033 |
| LI DJ | 2 | 0.066 | JIAO SC | 1 | 0.033 |
| LI DH | 2 | 0.066 | JIAO CJ | 1 | 0.033 |
| LI CZ | 2 | 0.066 | JIAO BH | 1 | 0.033 |
| LI BZ | 2 | 0.066 | JIANG ZP | 1 | 0.033 |
| LI AP | 2 | 0.066 | JIANG ZH | 1 | 0.033 |
| LI AM | 2 | 0.066 | JIANG Z | 1 | 0.033 |
| LEVIN JZ | 2 | 0.066 | JIANG YY | 1 | 0.033 |
| LEONE S | 2 | 0.066 | JIANG YW | 1 | 0.033 |
| LEONARD VE | 2 | 0.066 | JIANG XW | 1 | 0.033 |
| LENG RX | 2 | 0.066 | JIANG XL | 1 | 0.033 |
| LEIB DE | 2 | 0.066 | JIANG XD | 1 | 0.033 |
| LEI M | 2 | 0.066 | JIANG XB | 1 | 0.033 |
| LEI DP | 2 | 0.066 | JIANG WJ | 1 | 0.033 |
| LEFEVER S | 2 | 0.066 | JIANG WH | 1 | 0.033 |
| LEE WK | 2 | 0.066 | JIANG WF | 1 | 0.033 |
| LEE TL | 2 | 0.066 | JIANG TT | 1 | 0.033 |
| LEE ST | 2 | 0.066 | JIANG TF | 1 | 0.033 |
| LEE JC | 2 | 0.066 | JIANG SL | 1 | 0.033 |
| LEE EJ | 2 | 0.066 | JIANG SB | 1 | 0.033 |
| LEE DY | 2 | 0.066 | JIANG RZ | 1 | 0.033 |
| LEE D | 2 | 0.066 | JIANG RQ | 1 | 0.033 |
| LEE C | 2 | 0.066 | JIANG PT | 1 | 0.033 |
| LAWRENCE TS | 2 | 0.066 | JIANG MJ | 1 | 0.033 |
| LAN XY | 2 | 0.066 | JIANG LW | 1 | 0.033 |
| LAN L | 2 | 0.066 | JIANG LM | 1 | 0.033 |
| LAN HY | 2 | 0.066 | JIANG KW | 1 | 0.033 |
| LAMMENS T | 2 | 0.066 | JIANG KH | 1 | 0.033 |
| LALWANI MK | 2 | 0.066 | JIANG JP | 1 | 0.033 |
| LAKHOTIA SC | 2 | 0.066 | JIANG JJ | 1 | 0.033 |
| LAI YD | 2 | 0.066 | JIANG HZ | 1 | 0.033 |
| LAI MC | 2 | 0.066 | JIANG HP | 1 | 0.033 |
| LAI K | 2 | 0.066 | JIANG HC | 1 | 0.033 |
| LAI HM | 2 | 0.066 | JIANG GZ | 1 | 0.033 |
| LAI F | 2 | 0.066 | JIANG GJ | 1 | 0.033 |
| LAGARDE J | 2 | 0.066 | JIANG D | 1 | 0.033 |
| KUROKAWA R | 2 | 0.066 | JIANG CZ | 1 | 0.033 |
| KURIHARA M | 2 | 0.066 | JIANG CQ | 1 | 0.033 |
| KUNZ M | 2 | 0.066 | JIANG CH | 1 | 0.033 |
| KUMAR V | 2 | 0.066 | JIANG C | 1 | 0.033 |
| KULESZA CA | 2 | 0.066 | JIANG BY | 1 | 0.033 |
| KUBOTA T | 2 | 0.066 | JIANG BC | 1 | 0.033 |
| KUBO M | 2 | 0.066 | JIANG AR | 1 | 0.033 |
| KUANG YT | 2 | 0.066 | JIAN ZX | 1 | 0.033 |
| KUANG XM | 2 | 0.066 | JIAN LG | 1 | 0.033 |
| KUANG DF | 2 | 0.066 | JIAN DD | 1 | 0.033 |
| KRUSE TA | 2 | 0.066 | JIA ZJ | 1 | 0.033 |
| KRISTENSEN VN | 2 | 0.066 | JIA ZF | 1 | 0.033 |
| KRETZSCHMAR HA | 2 | 0.066 | JIA YL | 1 | 0.033 |
| KRAUS TFJ | 2 | 0.066 | JIA YJ | 1 | 0.033 |
| KOZIOL MJ | 2 | 0.066 | JIA YH | 1 | 0.033 |
| KOSSENKOV AV | 2 | 0.066 | JIA YB | 1 | 0.033 |
| KORTMAN G | 2 | 0.066 | JIA XY | 1 | 0.033 |
| KOROSTOWSKI L | 2 | 0.066 | JIA XQ | 1 | 0.033 |
| KONG Y | 2 | 0.066 | JIA XJ | 1 | 0.033 |
| KONG XQ | 2 | 0.066 | JIA X | 1 | 0.033 |
| KONG W | 2 | 0.066 | JIA WH | 1 | 0.033 |
| KONG LM | 2 | 0.066 | JIA W | 1 | 0.033 |
| KONG DX | 2 | 0.066 | JIA TT | 1 | 0.033 |
| KONG B | 2 | 0.066 | JIA SQ | 1 | 0.033 |
| KOMATSU H | 2 | 0.066 | JIA SL | 1 | 0.033 |
| KOK K | 2 | 0.066 | JIA P | 1 | 0.033 |
| KOGURE T | 2 | 0.066 | JIA MQ | 1 | 0.033 |
| KOGO R | 2 | 0.066 | JIA LZ | 1 | 0.033 |
| KNUDSEN KE | 2 | 0.066 | JIA LN | 1 | 0.033 |
| KNOWLES JA | 2 | 0.066 | JIA JP | 1 | 0.033 |
| KNOLL M | 2 | 0.066 | JIA HM | 1 | 0.033 |
| KLUIVER J | 2 | 0.066 | JIA HB | 1 | 0.033 |
| KITAYAMA J | 2 | 0.066 | JIA GM | 1 | 0.033 |
| KIRSCHNER MB | 2 | 0.066 | JIA F | 1 | 0.033 |
| KIM W | 2 | 0.066 | JIA DS | 1 | 0.033 |
| KIM SH | 2 | 0.066 | JIA BQ | 1 | 0.033 |
| KIM KM | 2 | 0.066 | JI ZY | 1 | 0.033 |
| KIM K | 2 | 0.066 | JI Z | 1 | 0.033 |
| KIM JH | 2 | 0.066 | JI YX | 1 | 0.033 |
| KIM JE | 2 | 0.066 | JI Y | 1 | 0.033 |
| KIM IM | 2 | 0.066 | JI WQ | 1 | 0.033 |
| KIM GE | 2 | 0.066 | JI W | 1 | 0.033 |
| KIM DH | 2 | 0.066 | JI TT | 1 | 0.033 |
| KIESEWETTER K | 2 | 0.066 | JI T | 1 | 0.033 |
| KHORKOVA O | 2 | 0.066 | JI R | 1 | 0.033 |
| KEENE JD | 2 | 0.066 | JI Q | 1 | 0.033 |
| KAWASAKI Y | 2 | 0.066 | JI KH | 1 | 0.033 |
| KATZE MG | 2 | 0.066 | JI JS | 1 | 0.033 |
| KATAOKA M | 2 | 0.066 | JI JH | 1 | 0.033 |
| KARJALAINEN J | 2 | 0.066 | JI J | 1 | 0.033 |
| KAPUSTA A | 2 | 0.066 | JI H | 1 | 0.033 |
| KAPRANOV P | 2 | 0.066 | JI GP | 1 | 0.033 |
| KAPATOS G | 2 | 0.066 | JI FQ | 1 | 0.033 |
| KANG Y | 2 | 0.066 | JI DM | 1 | 0.033 |
| KANG CM | 2 | 0.066 | JEYASEELAN K | 1 | 0.033 |
| KANDURI M | 2 | 0.066 | JESSURUN J | 1 | 0.033 |
| KALANTRY S | 2 | 0.066 | JEONG OS | 1 | 0.033 |
| KAESSMANN H | 2 | 0.066 | JEONG M | 1 | 0.033 |
| KADRI S | 2 | 0.066 | JEONG JJ | 1 | 0.033 |
| JUST A | 2 | 0.066 | JEONG JH | 1 | 0.033 |
| JUNG KY | 2 | 0.066 | JEON Y | 1 | 0.033 |
| JUNG KH | 2 | 0.066 | JEON KW | 1 | 0.033 |
| JUAN HF | 2 | 0.066 | JENSEN T | 1 | 0.033 |
| JOSHI A | 2 | 0.066 | JENSEN RV | 1 | 0.033 |
| JOSE PA | 2 | 0.066 | JENSEN L | 1 | 0.033 |
| JO YS | 2 | 0.066 | JENSEN KDC | 1 | 0.033 |
| JINNIN M | 2 | 0.066 | JENKINS AM | 1 | 0.033 |
| JING TL | 2 | 0.066 | JENJAROENPUN P | 1 | 0.033 |
| JIN ZL | 2 | 0.066 | JELIER R | 1 | 0.033 |
| JIN YF | 2 | 0.066 | JEANG KT | 1 | 0.033 |
| JIN Y | 2 | 0.066 | JEAN S | 1 | 0.033 |
| JIN WJ | 2 | 0.066 | JAZIN E | 1 | 0.033 |
| JIN W | 2 | 0.066 | JAZI MS | 1 | 0.033 |
| JIN T | 2 | 0.066 | JAYAWICKRAMARAJAH J | 1 | 0.033 |
| JIN J | 2 | 0.066 | JAYARAJ GG | 1 | 0.033 |
| JIN H | 2 | 0.066 | JAYAKODI M | 1 | 0.033 |
| JIN EJ | 2 | 0.066 | JAUCH R | 1 | 0.033 |
| JIN C | 2 | 0.066 | JARA C | 1 | 0.033 |
| JIN BL | 2 | 0.066 | JANKOWSKI J | 1 | 0.033 |
| JIN B | 2 | 0.066 | JANGA SC | 1 | 0.033 |
| JIANG ZY | 2 | 0.066 | JANG SJ | 1 | 0.033 |
| JIANG ZM | 2 | 0.066 | JANG H | 1 | 0.033 |
| JIANG ZL | 2 | 0.066 | JAN CH | 1 | 0.033 |
| JIANG YT | 2 | 0.066 | JAMSHIDI N | 1 | 0.033 |
| JIANG YQ | 2 | 0.066 | JAMES AR | 1 | 0.033 |
| JIANG YJ | 2 | 0.066 | JAMEEL S | 1 | 0.033 |
| JIANG YH | 2 | 0.066 | JALADANKI SK | 1 | 0.033 |
| JIANG XH | 2 | 0.066 | JAIN V | 1 | 0.033 |
| JIANG XC | 2 | 0.066 | JAIN S | 1 | 0.033 |
| JIANG WL | 2 | 0.066 | JAIN N | 1 | 0.033 |
| JIANG WK | 2 | 0.066 | JAIN AK | 1 | 0.033 |
| JIANG PF | 2 | 0.066 | JAIN A | 1 | 0.033 |
| JIANG PC | 2 | 0.066 | JAHANBANI F | 1 | 0.033 |
| JIANG MT | 2 | 0.066 | JAGT CV | 1 | 0.033 |
| JIANG LY | 2 | 0.066 | JAGER C | 1 | 0.033 |
| JIANG LH | 2 | 0.066 | JAGANNATHAN S | 1 | 0.033 |
| JIANG JH | 2 | 0.066 | JAE N | 1 | 0.033 |
| JIANG DN | 2 | 0.066 | JADALIHA M | 1 | 0.033 |
| JIANG AL | 2 | 0.066 | JACYSYN JF | 1 | 0.033 |
| JIAN S | 2 | 0.066 | JACQUES PE | 1 | 0.033 |
| JIA TY | 2 | 0.066 | JACOBSON D | 1 | 0.033 |
| JIA S | 2 | 0.066 | JACOBS EZ | 1 | 0.033 |
| JIA RN | 2 | 0.066 | JACOBI J | 1 | 0.033 |
| JIA Q | 2 | 0.066 | JACOB MD | 1 | 0.033 |
| JIA M | 2 | 0.066 | JACOB J | 1 | 0.033 |
| JIA J | 2 | 0.066 | JACKSON AR | 1 | 0.033 |
| JI XM | 2 | 0.066 | JACKSON AF | 1 | 0.033 |
| JI AM | 2 | 0.066 | IZUMIYA C | 1 | 0.033 |
| JI A | 2 | 0.066 | IZUMIKAWA K | 1 | 0.033 |
| JEONG S | 2 | 0.066 | IZRAELI S | 1 | 0.033 |
| JEON D | 2 | 0.066 | IWAYA T | 1 | 0.033 |
| JENSTER G | 2 | 0.066 | IWASAKI K | 1 | 0.033 |
| JENKINS RB | 2 | 0.066 | IVKOVIC TC | 1 | 0.033 |
| JENDRZEJEWSKI J | 2 | 0.066 | IVANOV P | 1 | 0.033 |
| JANITZ M | 2 | 0.066 | IVAN M | 1 | 0.033 |
| JACOBSEN A | 2 | 0.066 | ITZKOVITZ S | 1 | 0.033 |
| JACKSON MW | 2 | 0.066 | ITMAN C | 1 | 0.033 |
| JACKS T | 2 | 0.066 | ISRAEL Z | 1 | 0.033 |
| IZUMIYA Y | 2 | 0.066 | ISRAEL LL | 1 | 0.033 |
| IYER HK | 2 | 0.066 | ISMAIL N | 1 | 0.033 |
| IWAKIRI J | 2 | 0.066 | ISIN H | 1 | 0.033 |
| ISOGAI T | 2 | 0.066 | ISHIZUKA A | 1 | 0.033 |
| IRANPOUR M | 2 | 0.066 | ISHIMOTO H | 1 | 0.033 |
| INOUE Y | 2 | 0.066 | ISHIKAWA Y | 1 | 0.033 |
| INOUE S | 2 | 0.066 | ISHIKAWA H | 1 | 0.033 |
| INOUE K | 2 | 0.066 | ISHII N | 1 | 0.033 |
| ILIK IA | 2 | 0.066 | ISHIHARA G | 1 | 0.033 |
| IHN H | 2 | 0.066 | ISHIDA K | 1 | 0.033 |
| IGUCHI T | 2 | 0.066 | ISHIDA A | 1 | 0.033 |
| IBRAHIM S | 2 | 0.066 | ISHIBASHI M | 1 | 0.033 |
| IBBERSON M | 2 | 0.066 | ISAACS WB | 1 | 0.033 |
| HUTTERER GC | 2 | 0.066 | ISAACS SD | 1 | 0.033 |
| HUTCHINS AP | 2 | 0.066 | IRWIN DM | 1 | 0.033 |
| HUSSAIN I | 2 | 0.066 | IRMINGER-FINGER I | 1 | 0.033 |
| HUO R | 2 | 0.066 | IRIMIE A | 1 | 0.033 |
| HUNG T | 2 | 0.066 | IRIMIA M | 1 | 0.033 |
| HUNG MC | 2 | 0.066 | IRAOLA-GUZMAN S | 1 | 0.033 |
| HUGHES JM | 2 | 0.066 | IP JY | 1 | 0.033 |
| HUBNER A | 2 | 0.066 | IP JC | 1 | 0.033 |
| HUANG ZP | 2 | 0.066 | IOANNIDIS JPA | 1 | 0.033 |
| HUANG ZN | 2 | 0.066 | INOUE T | 1 | 0.033 |
| HUANG ZL | 2 | 0.066 | INOSTROZA-PONTA M | 1 | 0.033 |
| HUANG ZJ | 2 | 0.066 | INGLE JN | 1 | 0.033 |
| HUANG YY | 2 | 0.066 | INDRIERI A | 1 | 0.033 |
| HUANG YR | 2 | 0.066 | INDIG FE | 1 | 0.033 |
| HUANG YL | 2 | 0.066 | INCORONATO M | 1 | 0.033 |
| HUANG YC | 2 | 0.066 | INASE N | 1 | 0.033 |
| HUANG YA | 2 | 0.066 | INAOKA D | 1 | 0.033 |
| HUANG XZ | 2 | 0.066 | INAGAKI Y | 1 | 0.033 |
| HUANG XY | 2 | 0.066 | INADA T | 1 | 0.033 |
| HUANG XH | 2 | 0.066 | IMOTO S | 1 | 0.033 |
| HUANG WW | 2 | 0.066 | IMIG J | 1 | 0.033 |
| HUANG RM | 2 | 0.066 | IMANIRAD P | 1 | 0.033 |
| HUANG QJ | 2 | 0.066 | IMAMURA T | 1 | 0.033 |
| HUANG ML | 2 | 0.066 | IMAMURA K | 1 | 0.033 |
| HUANG LH | 2 | 0.066 | IMAMACHI N | 1 | 0.033 |
| HUANG KT | 2 | 0.066 | IMAM H | 1 | 0.033 |
| HUANG K | 2 | 0.066 | IMAI T | 1 | 0.033 |
| HUANG JR | 2 | 0.066 | IMAI K | 1 | 0.033 |
| HUANG JG | 2 | 0.066 | ILOTT NE | 1 | 0.033 |
| HUANG HF | 2 | 0.066 | ILOSKA D | 1 | 0.033 |
| HUANG HC | 2 | 0.066 | ILIOPOULOS D | 1 | 0.033 |
| HUANG GX | 2 | 0.066 | ILIEV R | 1 | 0.033 |
| HUANG G | 2 | 0.066 | ILARDI G | 1 | 0.033 |
| HUANG F | 2 | 0.066 | IKEDA K | 1 | 0.033 |
| HUANG DY | 2 | 0.066 | IJIRI K | 1 | 0.033 |
| HUANG CS | 2 | 0.066 | IIOTT NE | 1 | 0.033 |
| HUANG CQ | 2 | 0.066 | IIJIMA K | 1 | 0.033 |
| HUA YB | 2 | 0.066 | IGARASHI P | 1 | 0.033 |
| HU ZM | 2 | 0.066 | IDOGAWA M | 1 | 0.033 |
| HU YR | 2 | 0.066 | IDESES D | 1 | 0.033 |
| HU YJ | 2 | 0.066 | IDEN M | 1 | 0.033 |
| HU YH | 2 | 0.066 | ICHIKAWA D | 1 | 0.033 |
| HU XX | 2 | 0.066 | ICHIGOZAKI Y | 1 | 0.033 |
| HU W | 2 | 0.066 | IBRAHIM A | 1 | 0.033 |
| HU MH | 2 | 0.066 | IACCARINO I | 1 | 0.033 |
| HU M | 2 | 0.066 | HWANG SM | 1 | 0.033 |
| HU LJ | 2 | 0.066 | HWANG PA | 1 | 0.033 |
| HU JW | 2 | 0.066 | HWANG DW | 1 | 0.033 |
| HU HB | 2 | 0.066 | HWANG D | 1 | 0.033 |
| HU GQ | 2 | 0.066 | HUTVAGNER G | 1 | 0.033 |
| HU GK | 2 | 0.066 | HUTTNER WB | 1 | 0.033 |
| HU DW | 2 | 0.066 | HUTTELMAIER S | 1 | 0.033 |
| HU CY | 2 | 0.066 | HUSTEAD VA | 1 | 0.033 |
| HSUEH C | 2 | 0.066 | HUSSAIN Z | 1 | 0.033 |
| HSU CL | 2 | 0.066 | HURST LD | 1 | 0.033 |
| HSIAO J | 2 | 0.066 | HUR K | 1 | 0.033 |
| HOU ZY | 2 | 0.066 | HUO YX | 1 | 0.033 |
| HOU ZB | 2 | 0.066 | HUO X | 1 | 0.033 |
| HOU YY | 2 | 0.066 | HUO JX | 1 | 0.033 |
| HOU YX | 2 | 0.066 | HUO GX | 1 | 0.033 |
| HOU YL | 2 | 0.066 | HUNTER A | 1 | 0.033 |
| HOU Y | 2 | 0.066 | HUNTEN S | 1 | 0.033 |
| HOSONO Y | 2 | 0.066 | HUNT SE | 1 | 0.033 |
| HORABIN JI | 2 | 0.066 | HUNG RJ | 1 | 0.033 |
| HONG WW | 2 | 0.066 | HUNG GN | 1 | 0.033 |
| HONG W | 2 | 0.066 | HUNG CL | 1 | 0.033 |
| HOMBACH S | 2 | 0.066 | HUMMEL M | 1 | 0.033 |
| HOCHBERG A | 2 | 0.066 | HUME DA | 1 | 0.033 |
| HIYOSHI M | 2 | 0.066 | HUMBERG J | 1 | 0.033 |
| HINTEN M | 2 | 0.066 | HUMANN FC | 1 | 0.033 |
| HEWARD JA | 2 | 0.066 | HULL H | 1 | 0.033 |
| HESSAM S | 2 | 0.066 | HUIZINGA TW | 1 | 0.033 |
| HELGASON CD | 2 | 0.066 | HUISING MO | 1 | 0.033 |
| HEGER A | 2 | 0.066 | HUI RT | 1 | 0.033 |
| HEAD SR | 2 | 0.066 | HUI N | 1 | 0.033 |
| HE ZW | 2 | 0.066 | HUI HL | 1 | 0.033 |
| HE YL | 2 | 0.066 | HUH MI | 1 | 0.033 |
| HE YF | 2 | 0.066 | HUGHES JR | 1 | 0.033 |
| HE XY | 2 | 0.066 | HUGHES J | 1 | 0.033 |
| HE XL | 2 | 0.066 | HUGHES C | 1 | 0.033 |
| HE XH | 2 | 0.066 | HUGE A | 1 | 0.033 |
| HE XF | 2 | 0.066 | HUERTAS D | 1 | 0.033 |
| HE WL | 2 | 0.066 | HUERTA S | 1 | 0.033 |
| HE MJ | 2 | 0.066 | HUEHN A | 1 | 0.033 |
| HE LL | 2 | 0.066 | HUDSON WH | 1 | 0.033 |
| HE JX | 2 | 0.066 | HUDSON QJ | 1 | 0.033 |
| HE JH | 2 | 0.066 | HUDDLESTON J | 1 | 0.033 |
| HE JC | 2 | 0.066 | HUBNER N | 1 | 0.033 |
| HE H | 2 | 0.066 | HUBBARD TJ | 1 | 0.033 |
| HE GQ | 2 | 0.066 | HUANG ZY | 1 | 0.033 |
| HE FW | 2 | 0.066 | HUANG ZH | 1 | 0.033 |
| HE FT | 2 | 0.066 | HUANG YZ | 1 | 0.033 |
| HE DL | 2 | 0.066 | HUANG YS | 1 | 0.033 |
| HE DF | 2 | 0.066 | HUANG YK | 1 | 0.033 |
| HE DD | 2 | 0.066 | HUANG YH | 1 | 0.033 |
| HE D | 2 | 0.066 | HUANG YB | 1 | 0.033 |
| HE CJ | 2 | 0.066 | HUANG XW | 1 | 0.033 |
| HE C | 2 | 0.066 | HUANG XT | 1 | 0.033 |
| HAYBAECK J | 2 | 0.066 | HUANG XJ | 1 | 0.033 |
| HAUPTMAN N | 2 | 0.066 | HUANG XB | 1 | 0.033 |
| HART RP | 2 | 0.066 | HUANG WZ | 1 | 0.033 |
| HARROW J | 2 | 0.066 | HUANG WX | 1 | 0.033 |
| HARRIS LN | 2 | 0.066 | HUANG WT | 1 | 0.033 |
| HARDISON RC | 2 | 0.066 | HUANG WQ | 1 | 0.033 |
| HAO YJ | 2 | 0.066 | HUANG WH | 1 | 0.033 |
| HAO YH | 2 | 0.066 | HUANG TH | 1 | 0.033 |
| HAO Y | 2 | 0.066 | HUANG SX | 1 | 0.033 |
| HAO XS | 2 | 0.066 | HUANG SS | 1 | 0.033 |
| HAN WD | 2 | 0.066 | HUANG SN | 1 | 0.033 |
| HAN W | 2 | 0.066 | HUANG SL | 1 | 0.033 |
| HAN SY | 2 | 0.066 | HUANG SK | 1 | 0.033 |
| HAN SP | 2 | 0.066 | HUANG SH | 1 | 0.033 |
| HAN RH | 2 | 0.066 | HUANG RY | 1 | 0.033 |
| HAN QQ | 2 | 0.066 | HUANG RX | 1 | 0.033 |
| HAN Q | 2 | 0.066 | HUANG RT | 1 | 0.033 |
| HAN GS | 2 | 0.066 | HUANG RL | 1 | 0.033 |
| HALLIDAY GM | 2 | 0.066 | HUANG QX | 1 | 0.033 |
| HALLER H | 2 | 0.066 | HUANG QS | 1 | 0.033 |
| HALLE D | 2 | 0.066 | HUANG Q | 1 | 0.033 |
| HAJJARI M | 2 | 0.066 | HUANG PF | 1 | 0.033 |
| HAHN SA | 2 | 0.066 | HUANG O | 1 | 0.033 |
| HAEMMERLE M | 2 | 0.066 | HUANG NS | 1 | 0.033 |
| HABER M | 2 | 0.066 | HUANG N | 1 | 0.033 |
| HAAS S | 2 | 0.066 | HUANG MJ | 1 | 0.033 |
| GYLLENSTEN U | 2 | 0.066 | HUANG MG | 1 | 0.033 |
| GURUCEAGA E | 2 | 0.066 | HUANG LS | 1 | 0.033 |
| GUPTA RA | 2 | 0.066 | HUANG LR | 1 | 0.033 |
| GUO YY | 2 | 0.066 | HUANG LN | 1 | 0.033 |
| GUO W | 2 | 0.066 | HUANG KY | 1 | 0.033 |
| GUO QY | 2 | 0.066 | HUANG KL | 1 | 0.033 |
| GUO QH | 2 | 0.066 | HUANG KF | 1 | 0.033 |
| GUO LL | 2 | 0.066 | HUANG JZ | 1 | 0.033 |
| GUO HY | 2 | 0.066 | HUANG JX | 1 | 0.033 |
| GUMIREDDY K | 2 | 0.066 | HUANG JQ | 1 | 0.033 |
| GUIL S | 2 | 0.066 | HUANG JB | 1 | 0.033 |
| GUIBOURT V | 2 | 0.066 | HUANG HY | 1 | 0.033 |
| GUI X | 2 | 0.066 | HUANG HX | 1 | 0.033 |
| GU XS | 2 | 0.066 | HUANG HW | 1 | 0.033 |
| GU W | 2 | 0.066 | HUANG HT | 1 | 0.033 |
| GU M | 2 | 0.066 | HUANG HQ | 1 | 0.033 |
| GROSSI E | 2 | 0.066 | HUANG HJ | 1 | 0.033 |
| GROSS M | 2 | 0.066 | HUANG HD | 1 | 0.033 |
| GROMATZKY AA | 2 | 0.066 | HUANG HB | 1 | 0.033 |
| GREPO N | 2 | 0.066 | HUANG GY | 1 | 0.033 |
| GREINER A | 2 | 0.066 | HUANG GM | 1 | 0.033 |
| GREGORY BD | 2 | 0.066 | HUANG FZ | 1 | 0.033 |
| GREENE CM | 2 | 0.066 | HUANG DH | 1 | 0.033 |
| GREEN ED | 2 | 0.066 | HUANG CZ | 1 | 0.033 |
| GRECO S | 2 | 0.066 | HUANG BJ | 1 | 0.033 |
| GRAY SG | 2 | 0.066 | HUANG B | 1 | 0.033 |
| GRAMMATIKAKIS I | 2 | 0.066 | HUANG AF | 1 | 0.033 |
| GRAHAM LD | 2 | 0.066 | HUAN XK | 1 | 0.033 |
| GRAHAM B | 2 | 0.066 | HUAI MF | 1 | 0.033 |
| GOVINDARAJAN S | 2 | 0.066 | HUA ZJ | 1 | 0.033 |
| GOSAI SJ | 2 | 0.066 | HUA Z | 1 | 0.033 |
| GOOD DJ | 2 | 0.066 | HUA XD | 1 | 0.033 |
| GONZALEZ JM | 2 | 0.066 | HUA SF | 1 | 0.033 |
| GONG J | 2 | 0.066 | HUA JJ | 1 | 0.033 |
| GONG GC | 2 | 0.066 | HUA JD | 1 | 0.033 |
| GONG B | 2 | 0.066 | HUA J | 1 | 0.033 |
| GONG AY | 2 | 0.066 | HUA HB | 1 | 0.033 |
| GLAVAC D | 2 | 0.066 | HUA D | 1 | 0.033 |
| GIMBA ERP | 2 | 0.066 | HU ZW | 1 | 0.033 |
| GILON M | 2 | 0.066 | HU ZT | 1 | 0.033 |
| GILBERT I | 2 | 0.066 | HU ZQ | 1 | 0.033 |
| GHOSAL S | 2 | 0.066 | HU ZL | 1 | 0.033 |
| GHAFOURI-FARD S | 2 | 0.066 | HU YY | 1 | 0.033 |
| GHADESSI M | 2 | 0.066 | HU YP | 1 | 0.033 |
| GEVAERT K | 2 | 0.066 | HU YL | 1 | 0.033 |
| GERHARDINGER C | 2 | 0.066 | HU YF | 1 | 0.033 |
| GEORGIEV P | 2 | 0.066 | HU YC | 1 | 0.033 |
| GENG L | 2 | 0.066 | HU YB | 1 | 0.033 |
| GEISLER S | 2 | 0.066 | HU XM | 1 | 0.033 |
| GEFFERS R | 2 | 0.066 | HU XL | 1 | 0.033 |
| GE MQ | 2 | 0.066 | HU XD | 1 | 0.033 |
| GE JP | 2 | 0.066 | HU X | 1 | 0.033 |
| GAYEN S | 2 | 0.066 | HU WX | 1 | 0.033 |
| GAUTHERET D | 2 | 0.066 | HU WH | 1 | 0.033 |
| GARDINI A | 2 | 0.066 | HU WG | 1 | 0.033 |
| GARCIA-FONCILLAS J | 2 | 0.066 | HU SX | 1 | 0.033 |
| GARCIA R | 2 | 0.066 | HU SD | 1 | 0.033 |
| GAO YP | 2 | 0.066 | HU SB | 1 | 0.033 |
| GAO YF | 2 | 0.066 | HU RF | 1 | 0.033 |
| GAO TY | 2 | 0.066 | HU R | 1 | 0.033 |
| GAO SS | 2 | 0.066 | HU QS | 1 | 0.033 |
| GAO S | 2 | 0.066 | HU P | 1 | 0.033 |
| GAO LY | 2 | 0.066 | HU MJ | 1 | 0.033 |
| GAO LB | 2 | 0.066 | HU MC | 1 | 0.033 |
| GAO JJ | 2 | 0.066 | HU LW | 1 | 0.033 |
| GAO J | 2 | 0.066 | HU LT | 1 | 0.033 |
| GAO G | 2 | 0.066 | HU LR | 1 | 0.033 |
| GAO FY | 2 | 0.066 | HU LF | 1 | 0.033 |
| GAO D | 2 | 0.066 | HU KS | 1 | 0.033 |
| GAO CY | 2 | 0.066 | HU K | 1 | 0.033 |
| GAO CH | 2 | 0.066 | HU JY | 1 | 0.033 |
| GAO CF | 2 | 0.066 | HU HZ | 1 | 0.033 |
| GANDHI P | 2 | 0.066 | HU HL | 1 | 0.033 |
| GAN L | 2 | 0.066 | HU HK | 1 | 0.033 |
| GAN J | 2 | 0.066 | HU HH | 1 | 0.033 |
| GAN HL | 2 | 0.066 | HU GZ | 1 | 0.033 |
| GAMBICHLER T | 2 | 0.066 | HU GX | 1 | 0.033 |
| GALLETTI S | 2 | 0.066 | HU GW | 1 | 0.033 |
| GALLARDO-ESCARATE C | 2 | 0.066 | HU GL | 1 | 0.033 |
| GAGNE D | 2 | 0.066 | HU GC | 1 | 0.033 |
| GABELLINI D | 2 | 0.066 | HU FQ | 1 | 0.033 |
| FUNG CF | 2 | 0.066 | HU FF | 1 | 0.033 |
| FUKUSHIMA S | 2 | 0.066 | HU DZ | 1 | 0.033 |
| FU XL | 2 | 0.066 | HU DM | 1 | 0.033 |
| FU WM | 2 | 0.066 | HU DK | 1 | 0.033 |
| FU R | 2 | 0.066 | HU CX | 1 | 0.033 |
| FU Q | 2 | 0.066 | HU CP | 1 | 0.033 |
| FU M | 2 | 0.066 | HU CG | 1 | 0.033 |
| FU LY | 2 | 0.066 | HU CC | 1 | 0.033 |
| FU JY | 2 | 0.066 | HU BX | 1 | 0.033 |
| FU JX | 2 | 0.066 | HU BG | 1 | 0.033 |
| FU JP | 2 | 0.066 | HSU WM | 1 | 0.033 |
| FU GB | 2 | 0.066 | HSU JM | 1 | 0.033 |
| FU C | 2 | 0.066 | HSU JL | 1 | 0.033 |
| FROBERG JE | 2 | 0.066 | HSU J | 1 | 0.033 |
| FRENDEWEY D | 2 | 0.066 | HSIEH TH | 1 | 0.033 |
| FRANKE L | 2 | 0.066 | HSIAO HH | 1 | 0.033 |
| FOX A | 2 | 0.066 | HSIA SM | 1 | 0.033 |
| FOURNIER E | 2 | 0.066 | HRABOVSKY A | 1 | 0.033 |
| FORREST ME | 2 | 0.066 | HOYOS M | 1 | 0.033 |
| FONG KW | 2 | 0.066 | HOWARTH MM | 1 | 0.033 |
| FODDE R | 2 | 0.066 | HOWARD R | 1 | 0.033 |
| FLOCKHART RJ | 2 | 0.066 | HOWALD C | 1 | 0.033 |
| FLAVELL RA | 2 | 0.066 | HOUSMAN G | 1 | 0.033 |
| FIEDLER J | 2 | 0.066 | HOUSER SR | 1 | 0.033 |
| FESCHOTTE C | 2 | 0.066 | HOULSTON RS | 1 | 0.033 |
| FERRIS MT | 2 | 0.066 | HOU XL | 1 | 0.033 |
| FERNANDEZ-JIMENEZ N | 2 | 0.066 | HOU XB | 1 | 0.033 |
| FENG YH | 2 | 0.066 | HOU X | 1 | 0.033 |
| FENG JL | 2 | 0.066 | HOU WH | 1 | 0.033 |
| FENG D | 2 | 0.066 | HOU WG | 1 | 0.033 |
| FENDRICH J | 2 | 0.066 | HOU TY | 1 | 0.033 |
| FATEMI RP | 2 | 0.066 | HOU SC | 1 | 0.033 |
| FARINA L | 2 | 0.066 | HOU S | 1 | 0.033 |
| FANG Y | 2 | 0.066 | HOU Q | 1 | 0.033 |
| FANG XQ | 2 | 0.066 | HOU P | 1 | 0.033 |
| FANG SS | 2 | 0.066 | HOU MF | 1 | 0.033 |
| FANG N | 2 | 0.066 | HOU M | 1 | 0.033 |
| FANG L | 2 | 0.066 | HOU LL | 1 | 0.033 |
| FANG K | 2 | 0.066 | HOU LK | 1 | 0.033 |
| FANG JG | 2 | 0.066 | HOU LJ | 1 | 0.033 |
| FAN XM | 2 | 0.066 | HOU JY | 1 | 0.033 |
| FAN Q | 2 | 0.066 | HOU JX | 1 | 0.033 |
| FAN JY | 2 | 0.066 | HOU JK | 1 | 0.033 |
| FAN J | 2 | 0.066 | HOU HL | 1 | 0.033 |
| FADDA P | 2 | 0.066 | HOU F | 1 | 0.033 |
| FACHEL AA | 2 | 0.066 | HOU D | 1 | 0.033 |
| EVGRAFOV OV | 2 | 0.066 | HOU BF | 1 | 0.033 |
| ESTEBAN MA | 2 | 0.066 | HOU B | 1 | 0.033 |
| ESSERS PB | 2 | 0.066 | HOSOYA H | 1 | 0.033 |
| ERRIQUEZ D | 2 | 0.066 | HOSONO N | 1 | 0.033 |
| ERHO N | 2 | 0.066 | HORVATH S | 1 | 0.033 |
| ENROTH S | 2 | 0.066 | HORVAT S | 1 | 0.033 |
| ENRIGHT AJ | 2 | 0.066 | HORN F | 1 | 0.033 |
| ELLINGER J | 2 | 0.066 | HORLINGS HM | 1 | 0.033 |
| ELEMENTO O | 2 | 0.066 | HORLBECK MA | 1 | 0.033 |
| EL-TAWDI AHF | 2 | 0.066 | HORIIKE S | 1 | 0.033 |
| EHATA S | 2 | 0.066 | HORIGUCHI K | 1 | 0.033 |
| EGUCHI H | 2 | 0.066 | HORIE-INOUE K | 1 | 0.033 |
| DURU N | 2 | 0.066 | HOPPE S | 1 | 0.033 |
| DUBOULE D | 2 | 0.066 | HOOGLAND AM | 1 | 0.033 |
| DU XL | 2 | 0.066 | HONTI F | 1 | 0.033 |
| DU P | 2 | 0.066 | HONN KA | 1 | 0.033 |
| DU CL | 2 | 0.066 | HONG Y | 1 | 0.033 |
| DU C | 2 | 0.066 | HONG T | 1 | 0.033 |
| DRISCOLL JJ | 2 | 0.066 | HONG SJ | 1 | 0.033 |
| DORN GW | 2 | 0.066 | HONG Q | 1 | 0.033 |
| DONG ZQ | 2 | 0.066 | HONG MY | 1 | 0.033 |
| DONG Y | 2 | 0.066 | HONG LE | 1 | 0.033 |
| DONG XH | 2 | 0.066 | HONG K | 1 | 0.033 |
| DONG X | 2 | 0.066 | HONG JW | 1 | 0.033 |
| DONG P | 2 | 0.066 | HONG HH | 1 | 0.033 |
| DONG KR | 2 | 0.066 | HONDA N | 1 | 0.033 |
| DONG JS | 2 | 0.066 | HOMMA Y | 1 | 0.033 |
| DONG CX | 2 | 0.066 | HOLZMANN A | 1 | 0.033 |
| DONAGHEY J | 2 | 0.066 | HOLT RA | 1 | 0.033 |
| DITTRICH M | 2 | 0.066 | HOLMSTROM K | 1 | 0.033 |
| DISTECHE CM | 2 | 0.066 | HOLMDAHL R | 1 | 0.033 |
| DING XW | 2 | 0.066 | HOLLA P | 1 | 0.033 |
| DING X | 2 | 0.066 | HOLICK MF | 1 | 0.033 |
| DING Q | 2 | 0.066 | HOLDT L | 1 | 0.033 |
| DING N | 2 | 0.066 | HOLDEN T | 1 | 0.033 |
| DING HM | 2 | 0.066 | HOJFELDT J | 1 | 0.033 |
| DING HJ | 2 | 0.066 | HOFSTRAND R | 1 | 0.033 |
| DING CF | 2 | 0.066 | HOFMANN A | 1 | 0.033 |
| DIEPSTRA A | 2 | 0.066 | HOFKER MH | 1 | 0.033 |
| DIAZ-LAGARES A | 2 | 0.066 | HOFFMEIER K | 1 | 0.033 |
| DHARMADHIKARI AV | 2 | 0.066 | HOFFMANN S | 1 | 0.033 |
| DHAMIJA S | 2 | 0.066 | HOFFMANN MJ | 1 | 0.033 |
| DEY BK | 2 | 0.066 | HOFFMAN PN | 1 | 0.033 |
| DEWITT JJ | 2 | 0.066 | HOFFMAN A | 1 | 0.033 |
| DEOCESANO-PEREIRA C | 2 | 0.066 | HOF PR | 1 | 0.033 |
| DENG ZY | 2 | 0.066 | HOELL JI | 1 | 0.033 |
| DENG YF | 2 | 0.066 | HOELL J | 1 | 0.033 |
| DENG QW | 2 | 0.066 | HOEHN KL | 1 | 0.033 |
| DENG M | 2 | 0.066 | HODGIN JB | 1 | 0.033 |
| DENG L | 2 | 0.066 | HODA MA | 1 | 0.033 |
| DENG JJ | 2 | 0.066 | HOBERG E | 1 | 0.033 |
| DENG HL | 2 | 0.066 | HOANG A | 1 | 0.033 |
| DENG GR | 2 | 0.066 | HO T | 1 | 0.033 |
| DENG F | 2 | 0.066 | HO SY | 1 | 0.033 |
| DEB A | 2 | 0.066 | HO E | 1 | 0.033 |
| DE WINDT LJ | 2 | 0.066 | HO ASW | 1 | 0.033 |
| DE STROOPER B | 2 | 0.066 | HO A | 1 | 0.033 |
| DE NADAL E | 2 | 0.066 | HITTEL DS | 1 | 0.033 |
| DE JONG D | 2 | 0.066 | HITTE C | 1 | 0.033 |
| DAVULURI RV | 2 | 0.066 | HISHIDA T | 1 | 0.033 |
| DAVIS CP | 2 | 0.066 | HIRSCH R | 1 | 0.033 |
| DAVICIONI E | 2 | 0.066 | HIRSCH FR | 1 | 0.033 |
| DAS S | 2 | 0.066 | HIROTA K | 1 | 0.033 |
| DANG YN | 2 | 0.066 | HIRIART E | 1 | 0.033 |
| DANG CV | 2 | 0.066 | HIRANO T | 1 | 0.033 |
| DANDOLO L | 2 | 0.066 | HIRAJIMA S | 1 | 0.033 |
| DANDEKAR T | 2 | 0.066 | HINODA Y | 1 | 0.033 |
| DAI XZ | 2 | 0.066 | HINKLE C | 1 | 0.033 |
| DAI XX | 2 | 0.066 | HILLER M | 1 | 0.033 |
| DAI Q | 2 | 0.066 | HILL AVS | 1 | 0.033 |
| DAI M | 2 | 0.066 | HIGGS DR | 1 | 0.033 |
| DAI LH | 2 | 0.066 | HIGGINS MJ | 1 | 0.033 |
| DAI DJ | 2 | 0.066 | HIERLEMANN A | 1 | 0.033 |
| DAHIYA R | 2 | 0.066 | HEZRONI H | 1 | 0.033 |
| DACHET F | 2 | 0.066 | HEYN H | 1 | 0.033 |
| DA SACCO L | 2 | 0.066 | HEWSON C | 1 | 0.033 |
| CUI Y | 2 | 0.066 | HEUBACH J | 1 | 0.033 |
| CUI XX | 2 | 0.066 | HESSON J | 1 | 0.033 |
| CUI XL | 2 | 0.066 | HESS S | 1 | 0.033 |
| CUI XJ | 2 | 0.066 | HERTZ-FOWLER C | 1 | 0.033 |
| CUI P | 2 | 0.066 | HERTWIG F | 1 | 0.033 |
| CUI JY | 2 | 0.066 | HERSCHKOWITZ JI | 1 | 0.033 |
| CUI JJ | 2 | 0.066 | HERRIGES MJ | 1 | 0.033 |
| CROWE ML | 2 | 0.066 | HERRERA NG | 1 | 0.033 |
| CRIPPA S | 2 | 0.066 | HERRERA LA | 1 | 0.033 |
| CRAWFORD J | 2 | 0.066 | HEROLD-MENDE C | 1 | 0.033 |
| CORTON JC | 2 | 0.066 | HERNANDEZ-VARGAS H | 1 | 0.033 |
| CORDEIRO A | 2 | 0.066 | HERNANDEZ-RIVAS R | 1 | 0.033 |
| COPPOLA D | 2 | 0.066 | HERNANDEZ BY | 1 | 0.033 |
| CONDORELLI G | 2 | 0.066 | HERMEKING H | 1 | 0.033 |
| COLLINS S | 2 | 0.066 | HERMANS-BEIJNSBERGER S | 1 | 0.033 |
| COLLER J | 2 | 0.066 | HERMANS E | 1 | 0.033 |
| COLD S | 2 | 0.066 | HERINGTON AC | 1 | 0.033 |
| CLOUTIER SC | 2 | 0.066 | HERCEG Z | 1 | 0.033 |
| CLEMSON M | 2 | 0.066 | HERAS I | 1 | 0.033 |
| CLARK SJ | 2 | 0.066 | HEO JB | 1 | 0.033 |
| CLARK BS | 2 | 0.066 | HENRY-BEZY M | 1 | 0.033 |
| CHUNG HK | 2 | 0.066 | HENNIG S | 1 | 0.033 |
| CHUANG EY | 2 | 0.066 | HENNIG GW | 1 | 0.033 |
| CHU XY | 2 | 0.066 | HENNELLY S | 1 | 0.033 |
| CHU MJ | 2 | 0.066 | HENDRIX DA | 1 | 0.033 |
| CHU K | 2 | 0.066 | HENDRICKSON DG | 1 | 0.033 |
| CHOWDHURY RR | 2 | 0.066 | HENDRICK N | 1 | 0.033 |
| CHOW J | 2 | 0.066 | HENDERSON L | 1 | 0.033 |
| CHODROFF RA | 2 | 0.066 | HENAO-MEJIA J | 1 | 0.033 |
| CHING T | 2 | 0.066 | HEMNES A | 1 | 0.033 |
| CHIM CS | 2 | 0.066 | HEMMELOVA B | 1 | 0.033 |
| CHEUNG BB | 2 | 0.066 | HEMBERG M | 1 | 0.033 |
| CHENG Z | 2 | 0.066 | HELSMOORTEL HH | 1 | 0.033 |
| CHENG YY | 2 | 0.066 | HELSENS K | 1 | 0.033 |
| CHENG YT | 2 | 0.066 | HELMS JA | 1 | 0.033 |
| CHENG YL | 2 | 0.066 | HELMER-CITTERICH M | 1 | 0.033 |
| CHENG YF | 2 | 0.066 | HELM R | 1 | 0.033 |
| CHENG X | 2 | 0.066 | HELLIWELL CA | 1 | 0.033 |
| CHENG W | 2 | 0.066 | HELLEMANS J | 1 | 0.033 |
| CHENG SJ | 2 | 0.066 | HELIN K | 1 | 0.033 |
| CHENG SB | 2 | 0.066 | HEISE MT | 1 | 0.033 |
| CHENG JQ | 2 | 0.066 | HEISE M | 1 | 0.033 |
| CHENG JJ | 2 | 0.066 | HEINTZEN C | 1 | 0.033 |
| CHENG HW | 2 | 0.066 | HEINRICH J | 1 | 0.033 |
| CHENG H | 2 | 0.066 | HEIM MH | 1 | 0.033 |
| CHEN ZS | 2 | 0.066 | HEIDMANN T | 1 | 0.033 |
| CHEN ZM | 2 | 0.066 | HEI KW | 1 | 0.033 |
| CHEN YW | 2 | 0.066 | HEGI ME | 1 | 0.033 |
| CHEN YT | 2 | 0.066 | HEDBORG F | 1 | 0.033 |
| CHEN YP | 2 | 0.066 | HEDAN B | 1 | 0.033 |
| CHEN YL | 2 | 0.066 | HECHT PM | 1 | 0.033 |
| CHEN YB | 2 | 0.066 | HEARN S | 1 | 0.033 |
| CHEN YA | 2 | 0.066 | HE ZS | 1 | 0.033 |
| CHEN XY | 2 | 0.066 | HE ZC | 1 | 0.033 |
| CHEN XF | 2 | 0.066 | HE Z | 1 | 0.033 |
| CHEN XC | 2 | 0.066 | HE YY | 1 | 0.033 |
| CHEN WY | 2 | 0.066 | HE YA | 1 | 0.033 |
| CHEN WS | 2 | 0.066 | HE XX | 1 | 0.033 |
| CHEN WH | 2 | 0.066 | HE XT | 1 | 0.033 |
| CHEN WB | 2 | 0.066 | HE XP | 1 | 0.033 |
| CHEN TC | 2 | 0.066 | HE XM | 1 | 0.033 |
| CHEN SZ | 2 | 0.066 | HE XG | 1 | 0.033 |
| CHEN SG | 2 | 0.066 | HE WW | 1 | 0.033 |
| CHEN SD | 2 | 0.066 | HE WT | 1 | 0.033 |
| CHEN RJ | 2 | 0.066 | HE WM | 1 | 0.033 |
| CHEN RH | 2 | 0.066 | HE TY | 1 | 0.033 |
| CHEN QS | 2 | 0.066 | HE TP | 1 | 0.033 |
| CHEN QH | 2 | 0.066 | HE SX | 1 | 0.033 |
| CHEN N | 2 | 0.066 | HE SW | 1 | 0.033 |
| CHEN MS | 2 | 0.066 | HE SM | 1 | 0.033 |
| CHEN MR | 2 | 0.066 | HE SD | 1 | 0.033 |
| CHEN LX | 2 | 0.066 | HE SB | 1 | 0.033 |
| CHEN LQ | 2 | 0.066 | HE S | 1 | 0.033 |
| CHEN LH | 2 | 0.066 | HE RQ | 1 | 0.033 |
| CHEN JZ | 2 | 0.066 | HE QY | 1 | 0.033 |
| CHEN JX | 2 | 0.066 | HE QM | 1 | 0.033 |
| CHEN JN | 2 | 0.066 | HE PH | 1 | 0.033 |
| CHEN JM | 2 | 0.066 | HE P | 1 | 0.033 |
| CHEN JK | 2 | 0.066 | HE N | 1 | 0.033 |
| CHEN HW | 2 | 0.066 | HE MY | 1 | 0.033 |
| CHEN HL | 2 | 0.066 | HE MX | 1 | 0.033 |
| CHEN GL | 2 | 0.066 | HE ML | 1 | 0.033 |
| CHEN FL | 2 | 0.066 | HE LN | 1 | 0.033 |
| CHEN DW | 2 | 0.066 | HE JY | 1 | 0.033 |
| CHEN DL | 2 | 0.066 | HE JD | 1 | 0.033 |
| CHEN DK | 2 | 0.066 | HE HT | 1 | 0.033 |
| CHEN DH | 2 | 0.066 | HE HSHS | 1 | 0.033 |
| CHEN CX | 2 | 0.066 | HE HS | 1 | 0.033 |
| CHEN CL | 2 | 0.066 | HE F | 1 | 0.033 |
| CHEN CH | 2 | 0.066 | HE EM | 1 | 0.033 |
| CHAUDHRY MA | 2 | 0.066 | HE DX | 1 | 0.033 |
| CHATTERJEE T | 2 | 0.066 | HE CR | 1 | 0.033 |
| CHANG YN | 2 | 0.066 | HE CQ | 1 | 0.033 |
| CHANG L | 2 | 0.066 | HE BZ | 1 | 0.033 |
| CHANG KY | 2 | 0.066 | HE BY | 1 | 0.033 |
| CHANG JG | 2 | 0.066 | HE BM | 1 | 0.033 |
| CHANG CS | 2 | 0.066 | HAYNOR DR | 1 | 0.033 |
| CHANG C | 2 | 0.066 | HAYNES KR | 1 | 0.033 |
| CHAN WY | 2 | 0.066 | HAYES DF | 1 | 0.033 |
| CHAN MTV | 2 | 0.066 | HAYES BJ | 1 | 0.033 |
| CHAN KCC | 2 | 0.066 | HAYASHIZAKI Y | 1 | 0.033 |
| CHAN JYW | 2 | 0.066 | HAYASHI K | 1 | 0.033 |
| CHAMBERS CB | 2 | 0.066 | HAWKINS DS | 1 | 0.033 |
| CHALEI V | 2 | 0.066 | HAWKE DH | 1 | 0.033 |
| CHAKRAVARTY B | 2 | 0.066 | HAUSMAN GJ | 1 | 0.033 |
| CHAKRABARTI J | 2 | 0.066 | HAUSER MA | 1 | 0.033 |
| CASERO D | 2 | 0.066 | HAUG-BATZELL AK | 1 | 0.033 |
| CAO XT | 2 | 0.066 | HATZOGLOU M | 1 | 0.033 |
| CAO J | 2 | 0.066 | HATZIGEORGIOU AG | 1 | 0.033 |
| CAO G | 2 | 0.066 | HATA K | 1 | 0.033 |
| CAO DL | 2 | 0.066 | HASSAN SS | 1 | 0.033 |
| CANUTO EM | 2 | 0.066 | HASSAN MA | 1 | 0.033 |
| CAMPBELL M | 2 | 0.066 | HASHIMOTO Y | 1 | 0.033 |
| CALEGARI F | 2 | 0.066 | HASHAD D | 1 | 0.033 |
| CAJIGAS I | 2 | 0.066 | HASEGAWA Y | 1 | 0.033 |
| CAI Z | 2 | 0.066 | HASEGAWA K | 1 | 0.033 |
| CAI XY | 2 | 0.066 | HARVEY RP | 1 | 0.033 |
| CAI SJ | 2 | 0.066 | HARVEY E | 1 | 0.033 |
| CAI GX | 2 | 0.066 | HARTMANN D | 1 | 0.033 |
| CABILI MN | 2 | 0.066 | HARTL C | 1 | 0.033 |
| BYUN JI | 2 | 0.066 | HARTFELDER K | 1 | 0.033 |
| BUTTIGIEG E | 2 | 0.066 | HART CE | 1 | 0.033 |
| BURTON M | 2 | 0.066 | HARRISON PM | 1 | 0.033 |
| BURDET F | 2 | 0.066 | HARRISON AP | 1 | 0.033 |
| BURATOWSKI S | 2 | 0.066 | HARRIS RA | 1 | 0.033 |
| BU P | 2 | 0.066 | HARRIS DJ | 1 | 0.033 |
| BRUNNER AL | 2 | 0.066 | HARRIS C | 1 | 0.033 |
| BROWN MA | 2 | 0.066 | HARRIS A | 1 | 0.033 |
| BROWN CJ | 2 | 0.066 | HARRIES LW | 1 | 0.033 |
| BROSKOVA Z | 2 | 0.066 | HARMAN CCD | 1 | 0.033 |
| BROMBA M | 2 | 0.066 | HARGROVE AE | 1 | 0.033 |
| BROADBENT KM | 2 | 0.066 | HARADA Y | 1 | 0.033 |
| BRIGGS JA | 2 | 0.066 | HARADA M | 1 | 0.033 |
| BREDY TW | 2 | 0.066 | HARADA H | 1 | 0.033 |
| BRAUN T | 2 | 0.066 | HAQUE S | 1 | 0.033 |
| BRANDON C | 2 | 0.066 | HAO ZQ | 1 | 0.033 |
| BOYD J | 2 | 0.066 | HAO YX | 1 | 0.033 |
| BOURQUE G | 2 | 0.066 | HAO YT | 1 | 0.033 |
| BOTTCHER R | 2 | 0.066 | HAO YL | 1 | 0.033 |
| BORRESEN-DALE AL | 2 | 0.066 | HAO YB | 1 | 0.033 |
| BOON RA | 2 | 0.066 | HAO XB | 1 | 0.033 |
| BOND CS | 2 | 0.066 | HAO WS | 1 | 0.033 |
| BLANPAIN C | 2 | 0.066 | HAO S | 1 | 0.033 |
| BISCHOF O | 2 | 0.066 | HAO L | 1 | 0.033 |
| BIKLE DD | 2 | 0.066 | HAO JL | 1 | 0.033 |
| BIGLIA N | 2 | 0.066 | HAO J | 1 | 0.033 |
| BIECHE I | 2 | 0.066 | HAO DL | 1 | 0.033 |
| BI H | 2 | 0.066 | HAO D | 1 | 0.033 |
| BHATTACHARYA S | 2 | 0.066 | HAO CF | 1 | 0.033 |
| BHATTACHARYA A | 2 | 0.066 | HAO B | 1 | 0.033 |
| BHATT K | 2 | 0.066 | HANZELMANN S | 1 | 0.033 |
| BERGHOFF EG | 2 | 0.066 | HANTRAYE P | 1 | 0.033 |
| BENNETT CF | 2 | 0.066 | HANSON J | 1 | 0.033 |
| BENNER C | 2 | 0.066 | HANSEN K | 1 | 0.033 |
| BEHMANESH M | 2 | 0.066 | HANSEN A | 1 | 0.033 |
| BECKER-SANTOS DD | 2 | 0.066 | HANSCOM C | 1 | 0.033 |
| BECKER PB | 2 | 0.066 | HANNA JH | 1 | 0.033 |
| BECKER KG | 2 | 0.066 | HANG XW | 1 | 0.033 |
| BECHARA FG | 2 | 0.066 | HANG Q | 1 | 0.033 |
| BAYOUMI AS | 2 | 0.066 | HANG DH | 1 | 0.033 |
| BATISTA PJ | 2 | 0.066 | HANG D | 1 | 0.033 |
| BATAGOV AO | 2 | 0.066 | HAND JM | 1 | 0.033 |
| BASU U | 2 | 0.066 | HANASH S | 1 | 0.033 |
| BASSEL-DUBY R | 2 | 0.066 | HANAFUSA T | 1 | 0.033 |
| BARTONICEK N | 2 | 0.066 | HAN ZY | 1 | 0.033 |
| BARRY G | 2 | 0.066 | HAN ZX | 1 | 0.033 |
| BARIC RS | 2 | 0.066 | HAN ZP | 1 | 0.033 |
| BAO YJ | 2 | 0.066 | HAN Z | 1 | 0.033 |
| BAO Y | 2 | 0.066 | HAN YX | 1 | 0.033 |
| BAO XC | 2 | 0.066 | HAN YW | 1 | 0.033 |
| BANNON MJ | 2 | 0.066 | HAN YP | 1 | 0.033 |
| BALLARINO M | 2 | 0.066 | HAN YM | 1 | 0.033 |
| BALLANTYNE MD | 2 | 0.066 | HAN XY | 1 | 0.033 |
| BAK M | 2 | 0.066 | HAN XX | 1 | 0.033 |
| BAI ZQ | 2 | 0.066 | HAN XW | 1 | 0.033 |
| BAI YY | 2 | 0.066 | HAN XR | 1 | 0.033 |
| BAI P | 2 | 0.066 | HAN XL | 1 | 0.033 |
| BAI NX | 2 | 0.066 | HAN XH | 1 | 0.033 |
| BACKOFEN R | 2 | 0.066 | HAN TX | 1 | 0.033 |
| AZUAJE F | 2 | 0.066 | HAN TT | 1 | 0.033 |
| AUDAS TE | 2 | 0.066 | HAN SX | 1 | 0.033 |
| ATTENELLO FJ | 2 | 0.066 | HAN SQ | 1 | 0.033 |
| ATTARDI LD | 2 | 0.066 | HAN S | 1 | 0.033 |
| ATMADIBRATA B | 2 | 0.066 | HAN NN | 1 | 0.033 |
| ARNES L | 2 | 0.066 | HAN LM | 1 | 0.033 |
| ARLOTTA P | 2 | 0.066 | HAN K | 1 | 0.033 |
| APREA J | 2 | 0.066 | HAN JY | 1 | 0.033 |
| AO YF | 2 | 0.066 | HAN JX | 1 | 0.033 |
| ANGUERA MC | 2 | 0.066 | HAN JS | 1 | 0.033 |
| ANDERSON DM | 2 | 0.066 | HAN JL | 1 | 0.033 |
| ANAYA J | 2 | 0.066 | HAN HY | 1 | 0.033 |
| AN T | 2 | 0.066 | HAN HF | 1 | 0.033 |
| AN G | 2 | 0.066 | HAN H | 1 | 0.033 |
| AMIT I | 2 | 0.066 | HAN GM | 1 | 0.033 |
| AMARAL PP | 2 | 0.066 | HAN F | 1 | 0.033 |
| ALVES CP | 2 | 0.066 | HAN DD | 1 | 0.033 |
| ALEYASIN SA | 2 | 0.066 | HAN CY | 1 | 0.033 |
| ALEXANIAN M | 2 | 0.066 | HAN CH | 1 | 0.033 |
| AL-TOBASEI R | 2 | 0.066 | HAN C | 1 | 0.033 |
| AL-OMRAN M | 2 | 0.066 | HAN BM | 1 | 0.033 |
| AKIYAMA T | 2 | 0.066 | HAMOU MF | 1 | 0.033 |
| AKHADE VS | 2 | 0.066 | HAMILTON G | 1 | 0.033 |
| AHN R | 2 | 0.066 | HAMBLIN MH | 1 | 0.033 |
| AGUILO F | 2 | 0.066 | HAMAZAKI N | 1 | 0.033 |
| AERTS S | 2 | 0.066 | HAMAD EA | 1 | 0.033 |
| ADRIAENSSENS E | 2 | 0.066 | HALTER SD | 1 | 0.033 |
| ADRIAENS C | 2 | 0.066 | HALLUPP M | 1 | 0.033 |
| ABURATANI H | 2 | 0.066 | HALLIGAN DL | 1 | 0.033 |
| ABRAHAM JM | 2 | 0.066 | HALLEY P | 1 | 0.033 |
| ABOUNADER R | 2 | 0.066 | HALL N | 1 | 0.033 |
| ZYTNICKI M | 1 | 0.033 | HALL LL | 1 | 0.033 |
| ZYBAILOV BL | 1 | 0.033 | HALL JR | 1 | 0.033 |
| ZWEIGERDT R | 1 | 0.033 | HALL JA | 1 | 0.033 |
| ZUO ZX | 1 | 0.033 | HALL J | 1 | 0.033 |
| ZUO XL | 1 | 0.033 | HAJARNIS SS | 1 | 0.033 |
| ZUO LJ | 1 | 0.033 | HAISLOP K | 1 | 0.033 |
| ZUO LH | 1 | 0.033 | HAINES JE | 1 | 0.033 |
| ZUO CQ | 1 | 0.033 | HAGGARTY SJ | 1 | 0.033 |
| ZUK O | 1 | 0.033 | HAGEGE H | 1 | 0.033 |
| ZUCKERWISE L | 1 | 0.033 | HAGEDORN PH | 1 | 0.033 |
| ZU L | 1 | 0.033 | HAGEDORN CH | 1 | 0.033 |
| ZOU ZM | 1 | 0.033 | HAFER C | 1 | 0.033 |
| ZOU YT | 1 | 0.033 | HADZHIEV Y | 1 | 0.033 |
| ZOU XY | 1 | 0.033 | HADJI F | 1 | 0.033 |
| ZOU T | 1 | 0.033 | HACKETT TA | 1 | 0.033 |
| ZOU RH | 1 | 0.033 | HACKERMULLER J | 1 | 0.033 |
| ZOU QY | 1 | 0.033 | HACKER NF | 1 | 0.033 |
| ZOU Q | 1 | 0.033 | HAAS B | 1 | 0.033 |
| ZOU MX | 1 | 0.033 | HAAKE A | 1 | 0.033 |
| ZOU JL | 1 | 0.033 | HA VL | 1 | 0.033 |
| ZOU JH | 1 | 0.033 | HA T | 1 | 0.033 |
| ZOU J | 1 | 0.033 | GYSLING K | 1 | 0.033 |
| ZOU HW | 1 | 0.033 | GUVELI M | 1 | 0.033 |
| ZOU D | 1 | 0.033 | GUTKIND JS | 1 | 0.033 |
| ZOU CS | 1 | 0.033 | GUTIERREZ-PEREZ I | 1 | 0.033 |
| ZORNIG M | 1 | 0.033 | GUTIERREZ-ARCELUS M | 1 | 0.033 |
| ZONG S | 1 | 0.033 | GUSTAFSSON CM | 1 | 0.033 |
| ZONG JF | 1 | 0.033 | GUSELLA JF | 1 | 0.033 |
| ZONG H | 1 | 0.033 | GURHA P | 1 | 0.033 |
| ZON L | 1 | 0.033 | GURE AO | 1 | 0.033 |
| ZOCHER S | 1 | 0.033 | GUPTA V | 1 | 0.033 |
| ZISKIND A | 1 | 0.033 | GUPTA SK | 1 | 0.033 |
| ZIPORI D | 1 | 0.033 | GUPTA S | 1 | 0.033 |
| ZINN P | 1 | 0.033 | GUPTA M | 1 | 0.033 |
| ZIMMER R | 1 | 0.033 | GUO-LONG C | 1 | 0.033 |
| ZIMMER K | 1 | 0.033 | GUO ZD | 1 | 0.033 |
| ZILTENER G | 1 | 0.033 | GUO YX | 1 | 0.033 |
| ZIEGLER U | 1 | 0.033 | GUO YT | 1 | 0.033 |
| ZIATS MN | 1 | 0.033 | GUO XZ | 1 | 0.033 |
| ZI H | 1 | 0.033 | GUO XW | 1 | 0.033 |
| ZHUO YZ | 1 | 0.033 | GUO XP | 1 | 0.033 |
| ZHUO Y | 1 | 0.033 | GUO XJ | 1 | 0.033 |
| ZHUO XY | 1 | 0.033 | GUO XH | 1 | 0.033 |
| ZHUO W | 1 | 0.033 | GUO XC | 1 | 0.033 |
| ZHUO M | 1 | 0.033 | GUO WX | 1 | 0.033 |
| ZHUO F | 1 | 0.033 | GUO WQ | 1 | 0.033 |
| ZHUO DX | 1 | 0.033 | GUO WN | 1 | 0.033 |
| ZHUO CH | 1 | 0.033 | GUO WM | 1 | 0.033 |
| ZHUGE XJ | 1 | 0.033 | GUO WJ | 1 | 0.033 |
| ZHUANG SL | 1 | 0.033 | GUO WH | 1 | 0.033 |
| ZHUANG QY | 1 | 0.033 | GUO WB | 1 | 0.033 |
| ZHUANG M | 1 | 0.033 | GUO TZ | 1 | 0.033 |
| ZHUANG LK | 1 | 0.033 | GUO TT | 1 | 0.033 |
| ZHUANG K | 1 | 0.033 | GUO SS | 1 | 0.033 |
| ZHUANG JL | 1 | 0.033 | GUO SH | 1 | 0.033 |
| ZHUANG JJ | 1 | 0.033 | GUO SF | 1 | 0.033 |
| ZHUANG C | 1 | 0.033 | GUO S | 1 | 0.033 |
| ZHUANG BX | 1 | 0.033 | GUO R | 1 | 0.033 |
| ZHU ZY | 1 | 0.033 | GUO QN | 1 | 0.033 |
| ZHU ZL | 1 | 0.033 | GUO QL | 1 | 0.033 |
| ZHU ZJ | 1 | 0.033 | GUO Q | 1 | 0.033 |
| ZHU ZC | 1 | 0.033 | GUO PP | 1 | 0.033 |
| ZHU YW | 1 | 0.033 | GUO P | 1 | 0.033 |
| ZHU YT | 1 | 0.033 | GUO LY | 1 | 0.033 |
| ZHU YH | 1 | 0.033 | GUO LX | 1 | 0.033 |
| ZHU XY | 1 | 0.033 | GUO LW | 1 | 0.033 |
| ZHU XT | 1 | 0.033 | GUO LS | 1 | 0.033 |
| ZHU XM | 1 | 0.033 | GUO LN | 1 | 0.033 |
| ZHU XG | 1 | 0.033 | GUO LJ | 1 | 0.033 |
| ZHU XC | 1 | 0.033 | GUO LH | 1 | 0.033 |
| ZHU WT | 1 | 0.033 | GUO JZ | 1 | 0.033 |
| ZHU WP | 1 | 0.033 | GUO JY | 1 | 0.033 |
| ZHU WM | 1 | 0.033 | GUO JT | 1 | 0.033 |
| ZHU WG | 1 | 0.033 | GUO JH | 1 | 0.033 |
| ZHU TT | 1 | 0.033 | GUO JF | 1 | 0.033 |
| ZHU SY | 1 | 0.033 | GUO JC | 1 | 0.033 |
| ZHU SX | 1 | 0.033 | GUO HQ | 1 | 0.033 |
| ZHU SW | 1 | 0.033 | GUO HB | 1 | 0.033 |
| ZHU ST | 1 | 0.033 | GUO GQ | 1 | 0.033 |
| ZHU SQ | 1 | 0.033 | GUO GJ | 1 | 0.033 |
| ZHU SJ | 1 | 0.033 | GUO GG | 1 | 0.033 |
| ZHU SH | 1 | 0.033 | GUO G | 1 | 0.033 |
| ZHU RM | 1 | 0.033 | GUO FX | 1 | 0.033 |
| ZHU RJ | 1 | 0.033 | GUO FJ | 1 | 0.033 |
| ZHU R | 1 | 0.033 | GUO EK | 1 | 0.033 |
| ZHU QR | 1 | 0.033 | GUO DH | 1 | 0.033 |
| ZHU QN | 1 | 0.033 | GUO D | 1 | 0.033 |
| ZHU PP | 1 | 0.033 | GUO B | 1 | 0.033 |
| ZHU MZ | 1 | 0.033 | GUO AZ | 1 | 0.033 |
| ZHU MY | 1 | 0.033 | GUO AY | 1 | 0.033 |
| ZHU MJ | 1 | 0.033 | GUNDUZ C | 1 | 0.033 |
| ZHU MH | 1 | 0.033 | GUNDLING WE | 1 | 0.033 |
| ZHU MG | 1 | 0.033 | GUNAWARDENA HP | 1 | 0.033 |
| ZHU LM | 1 | 0.033 | GUNAWARDANE L | 1 | 0.033 |
| ZHU LH | 1 | 0.033 | GUNARATNE PH | 1 | 0.033 |
| ZHU LG | 1 | 0.033 | GUMUS ZH | 1 | 0.033 |
| ZHU JX | 1 | 0.033 | GULSOY G | 1 | 0.033 |
| ZHU JT | 1 | 0.033 | GULLER S | 1 | 0.033 |
| ZHU JS | 1 | 0.033 | GULATI AA | 1 | 0.033 |
| ZHU JK | 1 | 0.033 | GUL CY | 1 | 0.033 |
| ZHU HM | 1 | 0.033 | GUILLERMIER M | 1 | 0.033 |
| ZHU HG | 1 | 0.033 | GUILLAUD M | 1 | 0.033 |
| ZHU HF | 1 | 0.033 | GUIJARRO-MUNOZ I | 1 | 0.033 |
| ZHU GQ | 1 | 0.033 | GUIANG SF | 1 | 0.033 |
| ZHU GN | 1 | 0.033 | GUI YF | 1 | 0.033 |
| ZHU DY | 1 | 0.033 | GUI Y | 1 | 0.033 |
| ZHU DL | 1 | 0.033 | GUFFANTI G | 1 | 0.033 |
| ZHU DJ | 1 | 0.033 | GUFFANTI A | 1 | 0.033 |
| ZHU DH | 1 | 0.033 | GUERRERO-HERNANDEZ C | 1 | 0.033 |
| ZHU DD | 1 | 0.033 | GUERRERO G | 1 | 0.033 |
| ZHU CY | 1 | 0.033 | GUERNEC G | 1 | 0.033 |
| ZHU CJ | 1 | 0.033 | GUENTHER MG | 1 | 0.033 |
| ZHU CH | 1 | 0.033 | GUENNEWIG B | 1 | 0.033 |
| ZHU CC | 1 | 0.033 | GUDMUNDSSON J | 1 | 0.033 |
| ZHU C | 1 | 0.033 | GUDJONSSON SA | 1 | 0.033 |
| ZHU BZ | 1 | 0.033 | GUDJONSSON JE | 1 | 0.033 |
| ZHU BY | 1 | 0.033 | GUDBJARTSSON DF | 1 | 0.033 |
| ZHU BW | 1 | 0.033 | GUCEK M | 1 | 0.033 |
| ZHU AY | 1 | 0.033 | GUAY SP | 1 | 0.033 |
| ZHOU ZH | 1 | 0.033 | GUAUQUE-OLARTE S | 1 | 0.033 |
| ZHOU ZB | 1 | 0.033 | GUAN ZZ | 1 | 0.033 |
| ZHOU YC | 1 | 0.033 | GUAN YH | 1 | 0.033 |
| ZHOU YB | 1 | 0.033 | GUAN YF | 1 | 0.033 |
| ZHOU YA | 1 | 0.033 | GUAN XW | 1 | 0.033 |
| ZHOU XT | 1 | 0.033 | GUAN XL | 1 | 0.033 |
| ZHOU XK | 1 | 0.033 | GUAN W | 1 | 0.033 |
| ZHOU XJ | 1 | 0.033 | GUAN RH | 1 | 0.033 |
| ZHOU XD | 1 | 0.033 | GUAN QH | 1 | 0.033 |
| ZHOU XB | 1 | 0.033 | GUAN Q | 1 | 0.033 |
| ZHOU WY | 1 | 0.033 | GUAN M | 1 | 0.033 |
| ZHOU WB | 1 | 0.033 | GUAN H | 1 | 0.033 |
| ZHOU TL | 1 | 0.033 | GUAN GF | 1 | 0.033 |
| ZHOU SZ | 1 | 0.033 | GUAN DW | 1 | 0.033 |
| ZHOU SH | 1 | 0.033 | GUALANDI F | 1 | 0.033 |
| ZHOU SG | 1 | 0.033 | GUAGUERE E | 1 | 0.033 |
| ZHOU RZ | 1 | 0.033 | GU ZY | 1 | 0.033 |
| ZHOU QH | 1 | 0.033 | GU ZT | 1 | 0.033 |
| ZHOU PK | 1 | 0.033 | GU ZQ | 1 | 0.033 |
| ZHOU PJ | 1 | 0.033 | GU ZD | 1 | 0.033 |
| ZHOU NG | 1 | 0.033 | GU Z | 1 | 0.033 |
| ZHOU MM | 1 | 0.033 | GU YZ | 1 | 0.033 |
| ZHOU ML | 1 | 0.033 | GU XT | 1 | 0.033 |
| ZHOU LW | 1 | 0.033 | GU X | 1 | 0.033 |
| ZHOU LL | 1 | 0.033 | GU WQ | 1 | 0.033 |
| ZHOU LJ | 1 | 0.033 | GU WL | 1 | 0.033 |
| ZHOU KI | 1 | 0.033 | GU S | 1 | 0.033 |
| ZHOU K | 1 | 0.033 | GU QH | 1 | 0.033 |
| ZHOU JW | 1 | 0.033 | GU PQ | 1 | 0.033 |
| ZHOU JM | 1 | 0.033 | GU PJ | 1 | 0.033 |
| ZHOU JL | 1 | 0.033 | GU PC | 1 | 0.033 |
| ZHOU JK | 1 | 0.033 | GU LY | 1 | 0.033 |
| ZHOU JJ | 1 | 0.033 | GU L | 1 | 0.033 |
| ZHOU JB | 1 | 0.033 | GU JQ | 1 | 0.033 |
| ZHOU HP | 1 | 0.033 | GU JN | 1 | 0.033 |
| ZHOU HJ | 1 | 0.033 | GU J | 1 | 0.033 |
| ZHOU HD | 1 | 0.033 | GU GX | 1 | 0.033 |
| ZHOU GR | 1 | 0.033 | GU F | 1 | 0.033 |
| ZHOU GB | 1 | 0.033 | GU AQ | 1 | 0.033 |
| ZHOU DW | 1 | 0.033 | GRUTZNER F | 1 | 0.033 |
| ZHOU DL | 1 | 0.033 | GRUHL F | 1 | 0.033 |
| ZHOU CQ | 1 | 0.033 | GRUENERT DC | 1 | 0.033 |
| ZHOU BS | 1 | 0.033 | GROUNDS MD | 1 | 0.033 |
| ZHOU BG | 1 | 0.033 | GROSZER M | 1 | 0.033 |
| ZHONG ZY | 1 | 0.033 | GROSSMAN LI | 1 | 0.033 |
| ZHONG ZH | 1 | 0.033 | GROSSENHEIDER TC | 1 | 0.033 |
| ZHONG YR | 1 | 0.033 | GRIMM D | 1 | 0.033 |
| ZHONG YJ | 1 | 0.033 | GRIMALDI C | 1 | 0.033 |
| ZHONG YC | 1 | 0.033 | GRILLI A | 1 | 0.033 |
| ZHONG XW | 1 | 0.033 | GRIFFITHS-JONES S | 1 | 0.033 |
| ZHONG XM | 1 | 0.033 | GRESHOCK J | 1 | 0.033 |
| ZHONG XB | 1 | 0.033 | GRENIER JK | 1 | 0.033 |
| ZHONG WL | 1 | 0.033 | GREMLICH S | 1 | 0.033 |
| ZHONG TY | 1 | 0.033 | GREGORY RI | 1 | 0.033 |
| ZHONG TT | 1 | 0.033 | GREENING DW | 1 | 0.033 |
| ZHONG TF | 1 | 0.033 | GREEN RR | 1 | 0.033 |
| ZHONG T | 1 | 0.033 | GREEN PHR | 1 | 0.033 |
| ZHONG S | 1 | 0.033 | GREEN NH | 1 | 0.033 |
| ZHONG NB | 1 | 0.033 | GREALLY J | 1 | 0.033 |
| ZHONG L | 1 | 0.033 | GRAZIOLI A | 1 | 0.033 |
| ZHONG JJ | 1 | 0.033 | GRAY JM | 1 | 0.033 |
| ZHONG HL | 1 | 0.033 | GRAY E | 1 | 0.033 |
| ZHONG H | 1 | 0.033 | GRAUR D | 1 | 0.033 |
| ZHONG GZ | 1 | 0.033 | GRASSO CS | 1 | 0.033 |
| ZHONG GS | 1 | 0.033 | GRASSI L | 1 | 0.033 |
| ZHONG FL | 1 | 0.033 | GRASSI C | 1 | 0.033 |
| ZHONG FD | 1 | 0.033 | GRANT C | 1 | 0.033 |
| ZHONG DX | 1 | 0.033 | GRALINSKI LE | 1 | 0.033 |
| ZHONG BH | 1 | 0.033 | GRALINSKI L | 1 | 0.033 |
| ZHONG B | 1 | 0.033 | GRADY RM | 1 | 0.033 |
| ZHI XT | 1 | 0.033 | GOYAL A | 1 | 0.033 |
| ZHI XS | 1 | 0.033 | GOVINDARAJAN SS | 1 | 0.033 |
| ZHI XF | 1 | 0.033 | GOUSTIN AS | 1 | 0.033 |
| ZHI F | 1 | 0.033 | GOULD SB | 1 | 0.033 |
| ZHERNAKOVA A | 1 | 0.033 | GOSS PE | 1 | 0.033 |
| ZHENG ZX | 1 | 0.033 | GOSHIMA N | 1 | 0.033 |
| ZHENG ZS | 1 | 0.033 | GOSAL A | 1 | 0.033 |
| ZHENG ZM | 1 | 0.033 | GORODKIN J | 1 | 0.033 |
| ZHENG ZL | 1 | 0.033 | GORLICH D | 1 | 0.033 |
| ZHENG ZJ | 1 | 0.033 | GOREN A | 1 | 0.033 |
| ZHENG ZG | 1 | 0.033 | GORE M | 1 | 0.033 |
| ZHENG YY | 1 | 0.033 | GORDON JAR | 1 | 0.033 |
| ZHENG YX | 1 | 0.033 | GORDEBEKE PM | 1 | 0.033 |
| ZHENG YW | 1 | 0.033 | GORAB E | 1 | 0.033 |
| ZHENG YB | 1 | 0.033 | GOPINATH S | 1 | 0.033 |
| ZHENG XZ | 1 | 0.033 | GOPALAKRISHNAN K | 1 | 0.033 |
| ZHENG SX | 1 | 0.033 | GOOS YJ | 1 | 0.033 |
| ZHENG SL | 1 | 0.033 | GOODSTADT L | 1 | 0.033 |
| ZHENG SH | 1 | 0.033 | GOODRICH LV | 1 | 0.033 |
| ZHENG RP | 1 | 0.033 | GOODRICH JA | 1 | 0.033 |
| ZHENG R | 1 | 0.033 | GOODMAN M | 1 | 0.033 |
| ZHENG Q | 1 | 0.033 | GOODHEAD I | 1 | 0.033 |
| ZHENG PY | 1 | 0.033 | GOODELL MA | 1 | 0.033 |
| ZHENG MH | 1 | 0.033 | GOODE EL | 1 | 0.033 |
| ZHENG LY | 1 | 0.033 | GONZALEZ-RAMIREZ I | 1 | 0.033 |
| ZHENG LL | 1 | 0.033 | GONZALEZ-CELEIRO M | 1 | 0.033 |
| ZHENG LJ | 1 | 0.033 | GONZALEZ M | 1 | 0.033 |
| ZHENG JP | 1 | 0.033 | GONZALEZ I | 1 | 0.033 |
| ZHENG JM | 1 | 0.033 | GONZALES-ROYBAL G | 1 | 0.033 |
| ZHENG JG | 1 | 0.033 | GONZALES C | 1 | 0.033 |
| ZHENG JC | 1 | 0.033 | GONG ZQ | 1 | 0.033 |
| ZHENG HQ | 1 | 0.033 | GONG ZH | 1 | 0.033 |
| ZHENG GX | 1 | 0.033 | GONG ZF | 1 | 0.033 |
| ZHENG F | 1 | 0.033 | GONG YS | 1 | 0.033 |
| ZHENG CP | 1 | 0.033 | GONG YH | 1 | 0.033 |
| ZHENG CJ | 1 | 0.033 | GONG YB | 1 | 0.033 |
| ZHENG CC | 1 | 0.033 | GONG XL | 1 | 0.033 |
| ZHENG BL | 1 | 0.033 | GONG WJ | 1 | 0.033 |
| ZHENG BJ | 1 | 0.033 | GONG WD | 1 | 0.033 |
| ZHENG AB | 1 | 0.033 | GONG QY | 1 | 0.033 |
| ZHEN YF | 1 | 0.033 | GONG QT | 1 | 0.033 |
| ZHEN N | 1 | 0.033 | GONG PH | 1 | 0.033 |
| ZHEN H | 1 | 0.033 | GONG N | 1 | 0.033 |
| ZHDANOV VP | 1 | 0.033 | GONG MZ | 1 | 0.033 |
| ZHAO ZX | 1 | 0.033 | GONG LL | 1 | 0.033 |
| ZHAO ZW | 1 | 0.033 | GONG L | 1 | 0.033 |
| ZHAO YZ | 1 | 0.033 | GONG HB | 1 | 0.033 |
| ZHAO YQ | 1 | 0.033 | GONG DW | 1 | 0.033 |
| ZHAO YP | 1 | 0.033 | GONCALVES A | 1 | 0.033 |
| ZHAO YN | 1 | 0.033 | GOMEZ-RODRIGUEZ MJ | 1 | 0.033 |
| ZHAO YJ | 1 | 0.033 | GOMEZ-RODRIGUEZ J | 1 | 0.033 |
| ZHAO YH | 1 | 0.033 | GOMEZ-MALDONADO L | 1 | 0.033 |
| ZHAO YD | 1 | 0.033 | GOMEZ JA | 1 | 0.033 |
| ZHAO YC | 1 | 0.033 | GOMEZ G | 1 | 0.033 |
| ZHAO XW | 1 | 0.033 | GOLDSTONE A | 1 | 0.033 |
| ZHAO XP | 1 | 0.033 | GOLDGUR Y | 1 | 0.033 |
| ZHAO XK | 1 | 0.033 | GOKMEN-POLAR Y | 1 | 0.033 |
| ZHAO XD | 1 | 0.033 | GOKHAN S | 1 | 0.033 |
| ZHAO XC | 1 | 0.033 | GOKE J | 1 | 0.033 |
| ZHAO XB | 1 | 0.033 | GOFF L | 1 | 0.033 |
| ZHAO WY | 1 | 0.033 | GOETZL L | 1 | 0.033 |
| ZHAO WP | 1 | 0.033 | GOETZ MP | 1 | 0.033 |
| ZHAO WL | 1 | 0.033 | GOEL A | 1 | 0.033 |
| ZHAO WK | 1 | 0.033 | GOEDERT L | 1 | 0.033 |
| ZHAO WJ | 1 | 0.033 | GODSHALK SE | 1 | 0.033 |
| ZHAO WH | 1 | 0.033 | GODLEWSKI J | 1 | 0.033 |
| ZHAO WC | 1 | 0.033 | GNIRKE A | 1 | 0.033 |
| ZHAO WA | 1 | 0.033 | GNANCHANDRAN J | 1 | 0.033 |
| ZHAO SP | 1 | 0.033 | GLOVER AR | 1 | 0.033 |
| ZHAO SHD | 1 | 0.033 | GLOSS B | 1 | 0.033 |
| ZHAO SF | 1 | 0.033 | GLENNON JC | 1 | 0.033 |
| ZHAO SD | 1 | 0.033 | GLAZKO GV | 1 | 0.033 |
| ZHAO RZ | 1 | 0.033 | GLASSER ST | 1 | 0.033 |
| ZHAO RH | 1 | 0.033 | GLASS DJ | 1 | 0.033 |
| ZHAO RCH | 1 | 0.033 | GLASS CK | 1 | 0.033 |
| ZHAO QY | 1 | 0.033 | GIWOJNA A | 1 | 0.033 |
| ZHAO QS | 1 | 0.033 | GIVAN SA | 1 | 0.033 |
| ZHAO QF | 1 | 0.033 | GIT A | 1 | 0.033 |
| ZHAO PZ | 1 | 0.033 | GISSLINGER H | 1 | 0.033 |
| ZHAO NN | 1 | 0.033 | GISSLINGER B | 1 | 0.033 |
| ZHAO N | 1 | 0.033 | GIRI S | 1 | 0.033 |
| ZHAO MY | 1 | 0.033 | GIOVARELLI M | 1 | 0.033 |
| ZHAO MM | 1 | 0.033 | GIORGI FM | 1 | 0.033 |
| ZHAO MG | 1 | 0.033 | GIOIA U | 1 | 0.033 |
| ZHAO LS | 1 | 0.033 | GINGERAS TR | 1 | 0.033 |
| ZHAO LL | 1 | 0.033 | GINELLI E | 1 | 0.033 |
| ZHAO LD | 1 | 0.033 | GILL AJ | 1 | 0.033 |
| ZHAO LC | 1 | 0.033 | GILKS T | 1 | 0.033 |
| ZHAO KX | 1 | 0.033 | GILIANI S | 1 | 0.033 |
| ZHAO JX | 1 | 0.033 | GILCHRIST JJ | 1 | 0.033 |
| ZHAO JT | 1 | 0.033 | GIL N | 1 | 0.033 |
| ZHAO JN | 1 | 0.033 | GIGEK C | 1 | 0.033 |
| ZHAO HS | 1 | 0.033 | GIDONI M | 1 | 0.033 |
| ZHAO GN | 1 | 0.033 | GIDDINGS MC | 1 | 0.033 |
| ZHAO GJ | 1 | 0.033 | GIANNOULATOU E | 1 | 0.033 |
| ZHAO FY | 1 | 0.033 | GIANNOPOULOU E | 1 | 0.033 |
| ZHAO FL | 1 | 0.033 | GIANG K | 1 | 0.033 |
| ZHAO EH | 1 | 0.033 | GIALLOURAKIS CC | 1 | 0.033 |
| ZHAO DH | 1 | 0.033 | GIACOMINI CP | 1 | 0.033 |
| ZHAO DD | 1 | 0.033 | GHOSH Z | 1 | 0.033 |
| ZHAO CX | 1 | 0.033 | GHOSH T | 1 | 0.033 |
| ZHAO CP | 1 | 0.033 | GHERZI R | 1 | 0.033 |
| ZHAO CM | 1 | 0.033 | GHERARDI S | 1 | 0.033 |
| ZHAO CL | 1 | 0.033 | GHAZAVI F | 1 | 0.033 |
| ZHANGYUAN GY | 1 | 0.033 | GHAZAL S | 1 | 0.033 |
| ZHANG ZX | 1 | 0.033 | GHARPURE KM | 1 | 0.033 |
| ZHANG ZT | 1 | 0.033 | GHANBARIAN AT | 1 | 0.033 |
| ZHANG ZR | 1 | 0.033 | GEYER PK | 1 | 0.033 |
| ZHANG ZP | 1 | 0.033 | GESSANI S | 1 | 0.033 |
| ZHANG ZN | 1 | 0.033 | GESCHWIND DH | 1 | 0.033 |
| ZHANG ZB | 1 | 0.033 | GERTZ CC | 1 | 0.033 |
| ZHANG YK | 1 | 0.033 | GERSTEIN MB | 1 | 0.033 |
| ZHANG YD | 1 | 0.033 | GERHAUSER C | 1 | 0.033 |
| ZHANG XM | 1 | 0.033 | GERGELY F | 1 | 0.033 |
| ZHANG XB | 1 | 0.033 | GERBER S | 1 | 0.033 |
| ZHANG WQ | 1 | 0.033 | GERBER AP | 1 | 0.033 |
| ZHANG WF | 1 | 0.033 | GERANPAYEH L | 1 | 0.033 |
| ZHANG WB | 1 | 0.033 | GEORGAKILAS G | 1 | 0.033 |
| ZHANG TL | 1 | 0.033 | GENTILE M | 1 | 0.033 |
| ZHANG TF | 1 | 0.033 | GENG ZM | 1 | 0.033 |
| ZHANG SM | 1 | 0.033 | GENG TT | 1 | 0.033 |
| ZHANG SF | 1 | 0.033 | GENG Q | 1 | 0.033 |
| ZHANG SD | 1 | 0.033 | GENG MY | 1 | 0.033 |
| ZHANG SC | 1 | 0.033 | GENG JF | 1 | 0.033 |
| ZHANG SB | 1 | 0.033 | GENG GN | 1 | 0.033 |
| ZHANG RS | 1 | 0.033 | GENESCA A | 1 | 0.033 |
| ZHANG RL | 1 | 0.033 | GELLERT P | 1 | 0.033 |
| ZHANG QQ | 1 | 0.033 | GEISSLINGER G | 1 | 0.033 |
| ZHANG QFC | 1 | 0.033 | GEHRIG J | 1 | 0.033 |
| ZHANG QD | 1 | 0.033 | GEHRE M | 1 | 0.033 |
| ZHANG QA | 1 | 0.033 | GEGINAT J | 1 | 0.033 |
| ZHANG PZ | 1 | 0.033 | GEEVEN G | 1 | 0.033 |
| ZHANG PF | 1 | 0.033 | GEERDENS E | 1 | 0.033 |
| ZHANG PD | 1 | 0.033 | GEE JMW | 1 | 0.033 |
| ZHANG PB | 1 | 0.033 | GEBAUER F | 1 | 0.033 |
| ZHANG N | 1 | 0.033 | GE ZP | 1 | 0.033 |
| ZHANG MM | 1 | 0.033 | GE YQ | 1 | 0.033 |
| ZHANG MF | 1 | 0.033 | GE YH | 1 | 0.033 |
| ZHANG LS | 1 | 0.033 | GE XS | 1 | 0.033 |
| ZHANG LN | 1 | 0.033 | GE XP | 1 | 0.033 |
| ZHANG LE | 1 | 0.033 | GE XJ | 1 | 0.033 |
| ZHANG LC | 1 | 0.033 | GE XF | 1 | 0.033 |
| ZHANG KT | 1 | 0.033 | GE WS | 1 | 0.033 |
| ZHANG KS | 1 | 0.033 | GE W | 1 | 0.033 |
| ZHANG KR | 1 | 0.033 | GE PL | 1 | 0.033 |
| ZHANG KQ | 1 | 0.033 | GE MH | 1 | 0.033 |
| ZHANG KN | 1 | 0.033 | GE M | 1 | 0.033 |
| ZHANG KJ | 1 | 0.033 | GE JH | 1 | 0.033 |
| ZHANG JTT | 1 | 0.033 | GE JB | 1 | 0.033 |
| ZHANG JR | 1 | 0.033 | GE HJ | 1 | 0.033 |
| ZHANG JN | 1 | 0.033 | GE HA | 1 | 0.033 |
| ZHANG JM | 1 | 0.033 | GE F | 1 | 0.033 |
| ZHANG HZ | 1 | 0.033 | GE D | 1 | 0.033 |
| ZHANG HW | 1 | 0.033 | GE CC | 1 | 0.033 |
| ZHANG HT | 1 | 0.033 | GAYTHER SA | 1 | 0.033 |
| ZHANG HK | 1 | 0.033 | GAY S | 1 | 0.033 |
| ZHANG HJ | 1 | 0.033 | GAWRONSKI A | 1 | 0.033 |
| ZHANG HG | 1 | 0.033 | GAVZY SJ | 1 | 0.033 |
| ZHANG GZ | 1 | 0.033 | GAUTAM A | 1 | 0.033 |
| ZHANG GY | 1 | 0.033 | GAUR U | 1 | 0.033 |
| ZHANG GB | 1 | 0.033 | GAUGHWIN PM | 1 | 0.033 |
| ZHANG ES | 1 | 0.033 | GAUDUCHON P | 1 | 0.033 |
| ZHANG DW | 1 | 0.033 | GAUDREAULT N | 1 | 0.033 |
| ZHANG DJ | 1 | 0.033 | GATTESCO S | 1 | 0.033 |
| ZHANG DH | 1 | 0.033 | GATICA S | 1 | 0.033 |
| ZHANG CY | 1 | 0.033 | GATES K | 1 | 0.033 |
| ZHANG CX | 1 | 0.033 | GASTAMINZA P | 1 | 0.033 |
| ZHANG CW | 1 | 0.033 | GASRI-PLOTNITSKY L | 1 | 0.033 |
| ZHANG CM | 1 | 0.033 | GASIEWICZ TA | 1 | 0.033 |
| ZHANG CH | 1 | 0.033 | GASCOIGNE DK | 1 | 0.033 |
| ZHANG CG | 1 | 0.033 | GARZON R | 1 | 0.033 |
| ZHANG CB | 1 | 0.033 | GARRETT LJ | 1 | 0.033 |
| ZHANG BS | 1 | 0.033 | GARMIRE LX | 1 | 0.033 |
| ZHANG BH | 1 | 0.033 | GARMIRE L | 1 | 0.033 |
| ZHANG BG | 1 | 0.033 | GARITANO-TROJAOLA A | 1 | 0.033 |
| ZHANG AH | 1 | 0.033 | GARDINER BB | 1 | 0.033 |
| ZHAN YP | 1 | 0.033 | GARCIA-MANTEIGA JM | 1 | 0.033 |
| ZHAN Y | 1 | 0.033 | GARCIA-CUELLAR C | 1 | 0.033 |
| ZHAN XH | 1 | 0.033 | GARCIA JT | 1 | 0.033 |
| ZHAN SY | 1 | 0.033 | GARBE JC | 1 | 0.033 |
| ZHAN RC | 1 | 0.033 | GAO ZS | 1 | 0.033 |
| ZHAN QM | 1 | 0.033 | GAO ZK | 1 | 0.033 |
| ZHAN M | 1 | 0.033 | GAO YX | 1 | 0.033 |
| ZHAN L | 1 | 0.033 | GAO YS | 1 | 0.033 |
| ZHAN HX | 1 | 0.033 | GAO YL | 1 | 0.033 |
| ZHAN H | 1 | 0.033 | GAO YB | 1 | 0.033 |
| ZHAN F | 1 | 0.033 | GAO XZ | 1 | 0.033 |
| ZHAI YK | 1 | 0.033 | GAO XX | 1 | 0.033 |
| ZHAI X | 1 | 0.033 | GAO XR | 1 | 0.033 |
| ZHAI WL | 1 | 0.033 | GAO XH | 1 | 0.033 |
| ZHAI QL | 1 | 0.033 | GAO XC | 1 | 0.033 |
| ZHAI NL | 1 | 0.033 | GAO WS | 1 | 0.033 |
| ZHAI LM | 1 | 0.033 | GAO WH | 1 | 0.033 |
| ZHAI JM | 1 | 0.033 | GAO T | 1 | 0.033 |
| ZHAI J | 1 | 0.033 | GAO SY | 1 | 0.033 |
| ZHAI HY | 1 | 0.033 | GAO SM | 1 | 0.033 |
| ZHAI CW | 1 | 0.033 | GAO SL | 1 | 0.033 |
| ZHA WJ | 1 | 0.033 | GAO SH | 1 | 0.033 |
| ZEUNER RA | 1 | 0.033 | GAO R | 1 | 0.033 |
| ZENGLI Z | 1 | 0.033 | GAO MZ | 1 | 0.033 |
| ZENG ZL | 1 | 0.033 | GAO LZ | 1 | 0.033 |
| ZENG YG | 1 | 0.033 | GAO LK | 1 | 0.033 |
| ZENG XQ | 1 | 0.033 | GAO KT | 1 | 0.033 |
| ZENG XF | 1 | 0.033 | GAO KS | 1 | 0.033 |
| ZENG QH | 1 | 0.033 | GAO JZ | 1 | 0.033 |
| ZENG Q | 1 | 0.033 | GAO JP | 1 | 0.033 |
| ZENG MS | 1 | 0.033 | GAO JN | 1 | 0.033 |
| ZENG LY | 1 | 0.033 | GAO JF | 1 | 0.033 |
| ZENG JH | 1 | 0.033 | GAO JB | 1 | 0.033 |
| ZENG HJ | 1 | 0.033 | GAO HQ | 1 | 0.033 |
| ZENG GC | 1 | 0.033 | GAO GJ | 1 | 0.033 |
| ZENG FX | 1 | 0.033 | GAO GF | 1 | 0.033 |
| ZENG F | 1 | 0.033 | GAO FQ | 1 | 0.033 |
| ZENG DH | 1 | 0.033 | GAO FL | 1 | 0.033 |
| ZELLER U | 1 | 0.033 | GAO B | 1 | 0.033 |
| ZEITZ MJ | 1 | 0.033 | GAO A | 1 | 0.033 |
| ZEINODDINI M | 1 | 0.033 | GANESHRAM A | 1 | 0.033 |
| ZAVOLAN M | 1 | 0.033 | GANESH S | 1 | 0.033 |
| ZATSEPIN TS | 1 | 0.033 | GANDHY SU | 1 | 0.033 |
| ZARNEGAR BJ | 1 | 0.033 | GANDHI S | 1 | 0.033 |
| ZARATE R | 1 | 0.033 | GANDAL MJ | 1 | 0.033 |
| ZARABI H | 1 | 0.033 | GAN XN | 1 | 0.033 |
| ZAPHIROPOULOS PG | 1 | 0.033 | GAN WH | 1 | 0.033 |
| ZANOBINI M | 1 | 0.033 | GAN TQ | 1 | 0.033 |
| ZANG W | 1 | 0.033 | GAN SJ | 1 | 0.033 |
| ZANG RJ | 1 | 0.033 | GAN P | 1 | 0.033 |
| ZANG RC | 1 | 0.033 | GAN LM | 1 | 0.033 |
| ZANG R | 1 | 0.033 | GAN JL | 1 | 0.033 |
| ZANG QG | 1 | 0.033 | GAN HY | 1 | 0.033 |
| ZANG CS | 1 | 0.033 | GALVAN L | 1 | 0.033 |
| ZANG C | 1 | 0.033 | GALVAN DL | 1 | 0.033 |
| ZANETTE DL | 1 | 0.033 | GALLOWAY JL | 1 | 0.033 |
| ZAMUDIO JR | 1 | 0.033 | GALLICIO GA | 1 | 0.033 |
| ZAMBONI F | 1 | 0.033 | GALLART AP | 1 | 0.033 |
| ZAMANI M | 1 | 0.033 | GALLARDO D | 1 | 0.033 |
| ZAGO MA | 1 | 0.033 | GALIPON J | 1 | 0.033 |
| YUNUSOV D | 1 | 0.033 | GALIMBERTI D | 1 | 0.033 |
| YUN-BO F | 1 | 0.033 | GALEA S | 1 | 0.033 |
| YUN T | 1 | 0.033 | GALAN M | 1 | 0.033 |
| YUKSEL H | 1 | 0.033 | GAITI F | 1 | 0.033 |
| YUKAWA Y | 1 | 0.033 | GAIOTTO FA | 1 | 0.033 |
| YUE ZJ | 1 | 0.033 | GAILLARD MC | 1 | 0.033 |
| YUE YJ | 1 | 0.033 | GAGO-ZACHERT S | 1 | 0.033 |
| YUE YH | 1 | 0.033 | GAGAN JR | 1 | 0.033 |
| YUE XQ | 1 | 0.033 | GAFA R | 1 | 0.033 |
| YUE JY | 1 | 0.033 | GADAD SS | 1 | 0.033 |
| YUE HS | 1 | 0.033 | FYE S | 1 | 0.033 |
| YUE G | 1 | 0.033 | FUTSCHER BW | 1 | 0.033 |
| YUAN ZX | 1 | 0.033 | FUSTER D | 1 | 0.033 |
| YUAN ZT | 1 | 0.033 | FUSCHI P | 1 | 0.033 |
| YUAN ZS | 1 | 0.033 | FURUKAWA Y | 1 | 0.033 |
| YUAN YY | 1 | 0.033 | FURLAN G | 1 | 0.033 |
| YUAN YX | 1 | 0.033 | FURIO-TARI P | 1 | 0.033 |
| YUAN YW | 1 | 0.033 | FUNG JNT | 1 | 0.033 |
| YUAN YP | 1 | 0.033 | FUNAKOSHI H | 1 | 0.033 |
| YUAN XW | 1 | 0.033 | FULLER-PACE FV | 1 | 0.033 |
| YUAN XR | 1 | 0.033 | FUKUSHIMA T | 1 | 0.033 |
| YUAN XQ | 1 | 0.033 | FUKUHARA S | 1 | 0.033 |
| YUAN WY | 1 | 0.033 | FUKUDA K | 1 | 0.033 |
| YUAN SM | 1 | 0.033 | FUKAYAMA M | 1 | 0.033 |
| YUAN QX | 1 | 0.033 | FUJIYA T | 1 | 0.033 |
| YUAN PF | 1 | 0.033 | FUJIWARA H | 1 | 0.033 |
| YUAN LJ | 1 | 0.033 | FUJITA Y | 1 | 0.033 |
| YUAN HY | 1 | 0.033 | FUJIMURA T | 1 | 0.033 |
| YUAN HQ | 1 | 0.033 | FUJIMORI T | 1 | 0.033 |
| YUAN HJ | 1 | 0.033 | FUCILE C | 1 | 0.033 |
| YUAN HH | 1 | 0.033 | FUCHAROEN S | 1 | 0.033 |
| YUAN GC | 1 | 0.033 | FU YY | 1 | 0.033 |
| YUAN FL | 1 | 0.033 | FU YR | 1 | 0.033 |
| YUAN F | 1 | 0.033 | FU YL | 1 | 0.033 |
| YUAN DY | 1 | 0.033 | FU Y | 1 | 0.033 |
| YUAN DM | 1 | 0.033 | FU XP | 1 | 0.033 |
| YUAN DJ | 1 | 0.033 | FU XG | 1 | 0.033 |
| YUAN DD | 1 | 0.033 | FU W | 1 | 0.033 |
| YUAN CX | 1 | 0.033 | FU SJ | 1 | 0.033 |
| YUAN CL | 1 | 0.033 | FU S | 1 | 0.033 |
| YUAN CH | 1 | 0.033 | FU RF | 1 | 0.033 |
| YUAN C | 1 | 0.033 | FU PF | 1 | 0.033 |
| YU ZY | 1 | 0.033 | FU N | 1 | 0.033 |
| YU ZR | 1 | 0.033 | FU ML | 1 | 0.033 |
| YU ZG | 1 | 0.033 | FU JW | 1 | 0.033 |
| YU Z | 1 | 0.033 | FU JJ | 1 | 0.033 |
| YU YX | 1 | 0.033 | FU J | 1 | 0.033 |
| YU YL | 1 | 0.033 | FU HY | 1 | 0.033 |
| YU YC | 1 | 0.033 | FU HT | 1 | 0.033 |
| YU YB | 1 | 0.033 | FU GH | 1 | 0.033 |
| YU XY | 1 | 0.033 | FU FQ | 1 | 0.033 |
| YU XW | 1 | 0.033 | FU DQ | 1 | 0.033 |
| YU XL | 1 | 0.033 | FU D | 1 | 0.033 |
| YU XJ | 1 | 0.033 | FU CJ | 1 | 0.033 |
| YU XH | 1 | 0.033 | FU BQ | 1 | 0.033 |
| YU XG | 1 | 0.033 | FROMMEL SC | 1 | 0.033 |
| YU XF | 1 | 0.033 | FRITAH S | 1 | 0.033 |
| YU XD | 1 | 0.033 | FRIIS-HANSEN L | 1 | 0.033 |
| YU V | 1 | 0.033 | FRIGERIO CS | 1 | 0.033 |
| YU TY | 1 | 0.033 | FRIEMAN MB | 1 | 0.033 |
| YU TT | 1 | 0.033 | FRIEDERSDORF MB | 1 | 0.033 |
| YU TM | 1 | 0.033 | FRIEDERSDORF M | 1 | 0.033 |
| YU TH | 1 | 0.033 | FRIEDEL CC | 1 | 0.033 |
| YU TC | 1 | 0.033 | FRIED SK | 1 | 0.033 |
| YU SY | 1 | 0.033 | FRENETTE P | 1 | 0.033 |
| YU SR | 1 | 0.033 | FRENCH JD | 1 | 0.033 |
| YU SH | 1 | 0.033 | FREITAG-WOLF S | 1 | 0.033 |
| YU S | 1 | 0.033 | FREEMAN C | 1 | 0.033 |
| YU QY | 1 | 0.033 | FREEDMAN ML | 1 | 0.033 |
| YU QT | 1 | 0.033 | FREDERIKSEN KS | 1 | 0.033 |
| YU QM | 1 | 0.033 | FRAZAO JB | 1 | 0.033 |
| YU PW | 1 | 0.033 | FRASER M | 1 | 0.033 |
| YU NZ | 1 | 0.033 | FRANZEN J | 1 | 0.033 |
| YU MX | 1 | 0.033 | FRANSSON S | 1 | 0.033 |
| YU MM | 1 | 0.033 | FRANKLIN JL | 1 | 0.033 |
| YU LY | 1 | 0.033 | FRANK-BERTONCELJ M | 1 | 0.033 |
| YU LB | 1 | 0.033 | FRANK MR | 1 | 0.033 |
| YU KD | 1 | 0.033 | FRANCO R | 1 | 0.033 |
| YU JX | 1 | 0.033 | FRANCO B | 1 | 0.033 |
| YU JW | 1 | 0.033 | FRANCELLE L | 1 | 0.033 |
| YU JM | 1 | 0.033 | FRAMPTON J | 1 | 0.033 |
| YU JJ | 1 | 0.033 | FRAGA MF | 1 | 0.033 |
| YU JH | 1 | 0.033 | FRADE AF | 1 | 0.033 |
| YU JD | 1 | 0.033 | FOWLER JE | 1 | 0.033 |
| YU JC | 1 | 0.033 | FOULDS CE | 1 | 0.033 |
| YU HY | 1 | 0.033 | FORTUN A | 1 | 0.033 |
| YU HQ | 1 | 0.033 | FORTINI E | 1 | 0.033 |
| YU HL | 1 | 0.033 | FORSYTHE ES | 1 | 0.033 |
| YU HG | 1 | 0.033 | FORREST ARR | 1 | 0.033 |
| YU HC | 1 | 0.033 | FORNE T | 1 | 0.033 |
| YU FY | 1 | 0.033 | FORK C | 1 | 0.033 |
| YU FL | 1 | 0.033 | FOREY N | 1 | 0.033 |
| YU F | 1 | 0.033 | FONTUGNE J | 1 | 0.033 |
| YU DS | 1 | 0.033 | FONSECA-GUZMAN Y | 1 | 0.033 |
| YU DJ | 1 | 0.033 | FONSECA AS | 1 | 0.033 |
| YU DH | 1 | 0.033 | FOLEY JW | 1 | 0.033 |
| YU DD | 1 | 0.033 | FOINQUINOS A | 1 | 0.033 |
| YU D | 1 | 0.033 | FOEKENS JA | 1 | 0.033 |
| YU CW | 1 | 0.033 | FLYGARE J | 1 | 0.033 |
| YU CQ | 1 | 0.033 | FLORY M | 1 | 0.033 |
| YU CC | 1 | 0.033 | FLOREZ-ZAPATA NMV | 1 | 0.033 |
| YU CB | 1 | 0.033 | FLORENCEAU L | 1 | 0.033 |
| YU AL | 1 | 0.033 | FLISER D | 1 | 0.033 |
| YU AD | 1 | 0.033 | FLIPPOT R | 1 | 0.033 |
| YU A | 1 | 0.033 | FLETCHER JI | 1 | 0.033 |
| YOUSEFI B | 1 | 0.033 | FLEMING J | 1 | 0.033 |
| YOUNG RA | 1 | 0.033 | FLAX J | 1 | 0.033 |
| YOUNG GP | 1 | 0.033 | FLAVELL CR | 1 | 0.033 |
| YOUNG G | 1 | 0.033 | FLANNERY C | 1 | 0.033 |
| YOU ZS | 1 | 0.033 | FISKIN E | 1 | 0.033 |
| YOU YW | 1 | 0.033 | FISHER R | 1 | 0.033 |
| YOU YA | 1 | 0.033 | FISCHER M | 1 | 0.033 |
| YOU XT | 1 | 0.033 | FISCELLA M | 1 | 0.033 |
| YOU KY | 1 | 0.033 | FINCK BN | 1 | 0.033 |
| YOU J | 1 | 0.033 | FINATO N | 1 | 0.033 |
| YOU B | 1 | 0.033 | FILIPPOVA GN | 1 | 0.033 |
| YOSHIO S | 1 | 0.033 | FILIPOWICZ W | 1 | 0.033 |
| YOSHIMURA N | 1 | 0.033 | FILIPOVSKA A | 1 | 0.033 |
| YOSHIMOTO R | 1 | 0.033 | FIGUEROA T | 1 | 0.033 |
| YOSHIKAWA R | 1 | 0.033 | FIGUEROA DM | 1 | 0.033 |
| YOSHIDA M | 1 | 0.033 | FIGUEIREDO VC | 1 | 0.033 |
| YOSHIDA I | 1 | 0.033 | FIERS M | 1 | 0.033 |
| YOSHIDA H | 1 | 0.033 | FIELDS PA | 1 | 0.033 |
| YOON SO | 1 | 0.033 | FIEGL H | 1 | 0.033 |
| YOON SK | 1 | 0.033 | FEYDER M | 1 | 0.033 |
| YOON MJ | 1 | 0.033 | FETISCH J | 1 | 0.033 |
| YOON CH | 1 | 0.033 | FERSTER A | 1 | 0.033 |
| YONG ML | 1 | 0.033 | FERRO M | 1 | 0.033 |
| YONG J | 1 | 0.033 | FERRERO G | 1 | 0.033 |
| YONEMOTO H | 1 | 0.033 | FERRER J | 1 | 0.033 |
| YONEDA M | 1 | 0.033 | FERREIRA LRP | 1 | 0.033 |
| YOKOYAMA M | 1 | 0.033 | FERREIRA LB | 1 | 0.033 |
| YOKOYAMA KK | 1 | 0.033 | FERREIRA FM | 1 | 0.033 |
| YOKOTA T | 1 | 0.033 | FERRE F | 1 | 0.033 |
| YOKOTA N | 1 | 0.033 | FERRARINI M | 1 | 0.033 |
| YOKOI T | 1 | 0.033 | FERRARI E | 1 | 0.033 |
| YLIPAA A | 1 | 0.033 | FERRACIN M | 1 | 0.033 |
| YLI-HARJA O | 1 | 0.033 | FERNANDO TR | 1 | 0.033 |
| YING XM | 1 | 0.033 | FERNANDEZ-VALVERDE SL | 1 | 0.033 |
| YING XJ | 1 | 0.033 | FERNANDEZ-REBOLLO E | 1 | 0.033 |
| YING X | 1 | 0.033 | FERNANDEZ-BARRAL A | 1 | 0.033 |
| YING RC | 1 | 0.033 | FERNANDEZ M | 1 | 0.033 |
| YING MG | 1 | 0.033 | FERNANDEZ AF | 1 | 0.033 |
| YING HQ | 1 | 0.033 | FERLINI A | 1 | 0.033 |
| YIN ZY | 1 | 0.033 | FERGUSON JF | 1 | 0.033 |
| YIN ZT | 1 | 0.033 | FERG M | 1 | 0.033 |
| YIN ZJ | 1 | 0.033 | FENWICK PS | 1 | 0.033 |
| YIN YM | 1 | 0.033 | FENOGLIO C | 1 | 0.033 |
| YIN WZ | 1 | 0.033 | FENG YY | 1 | 0.033 |
| YIN WL | 1 | 0.033 | FENG YM | 1 | 0.033 |
| YIN SC | 1 | 0.033 | FENG XX | 1 | 0.033 |
| YIN PH | 1 | 0.033 | FENG XL | 1 | 0.033 |
| YIN P | 1 | 0.033 | FENG WD | 1 | 0.033 |
| YIN MH | 1 | 0.033 | FENG TB | 1 | 0.033 |
| YIN KJ | 1 | 0.033 | FENG SY | 1 | 0.033 |
| YIN K | 1 | 0.033 | FENG SQ | 1 | 0.033 |
| YIN JH | 1 | 0.033 | FENG SJ | 1 | 0.033 |
| YIN JF | 1 | 0.033 | FENG RL | 1 | 0.033 |
| YIN F | 1 | 0.033 | FENG PM | 1 | 0.033 |
| YIN D | 1 | 0.033 | FENG NG | 1 | 0.033 |
| YIN CL | 1 | 0.033 | FENG NA | 1 | 0.033 |
| YIN BS | 1 | 0.033 | FENG LJ | 1 | 0.033 |
| YIM GW | 1 | 0.033 | FENG HF | 1 | 0.033 |
| YI ZP | 1 | 0.033 | FENG GX | 1 | 0.033 |
| YI YF | 1 | 0.033 | FENG FK | 1 | 0.033 |
| YI Y | 1 | 0.033 | FENG DJ | 1 | 0.033 |
| YI XM | 1 | 0.033 | FENG CQ | 1 | 0.033 |
| YI JS | 1 | 0.033 | FENG CH | 1 | 0.033 |
| YI J | 1 | 0.033 | FENG BA | 1 | 0.033 |
| YEO L | 1 | 0.033 | FELSENFELD G | 1 | 0.033 |
| YEO GW | 1 | 0.033 | FELLEY-BOSCO E | 1 | 0.033 |
| YENNU-NANDA VG | 1 | 0.033 | FELDSTEIN O | 1 | 0.033 |
| YEN JC | 1 | 0.033 | FELDSER D | 1 | 0.033 |
| YELKEN BO | 1 | 0.033 | FELDMAN AM | 1 | 0.033 |
| YEH SY | 1 | 0.033 | FEI ZT | 1 | 0.033 |
| YEDAVALLI VSRK | 1 | 0.033 | FEI ZJ | 1 | 0.033 |
| YE ZY | 1 | 0.033 | FEI K | 1 | 0.033 |
| YE ZJ | 1 | 0.033 | FEI JY | 1 | 0.033 |
| YE ZC | 1 | 0.033 | FEI BJ | 1 | 0.033 |
| YE ZBA | 1 | 0.033 | FEHRMANN RSN | 1 | 0.033 |
| YE YJ | 1 | 0.033 | FEFILOVA AS | 1 | 0.033 |
| YE XY | 1 | 0.033 | FEDOROVA D | 1 | 0.033 |
| YE XJ | 1 | 0.033 | FEDORKO M | 1 | 0.033 |
| YE XH | 1 | 0.033 | FEDERATION A | 1 | 0.033 |
| YE X | 1 | 0.033 | FEBER A | 1 | 0.033 |
| YE WW | 1 | 0.033 | FEBBRAIO F | 1 | 0.033 |
| YE SW | 1 | 0.033 | FAZI F | 1 | 0.033 |
| YE SS | 1 | 0.033 | FAYDA M | 1 | 0.033 |
| YE MH | 1 | 0.033 | FAVARO E | 1 | 0.033 |
| YE LT | 1 | 0.033 | FAULHABER-WALTER R | 1 | 0.033 |
| YE LF | 1 | 0.033 | FASTMAN Y | 1 | 0.033 |
| YE JQ | 1 | 0.033 | FASOLOLO F | 1 | 0.033 |
| YE CY | 1 | 0.033 | FASOLD M | 1 | 0.033 |
| YE CX | 1 | 0.033 | FASANARO P | 1 | 0.033 |
| YE CC | 1 | 0.033 | FARSHCHIAN M | 1 | 0.033 |
| YE BQ | 1 | 0.033 | FARSETTI A | 1 | 0.033 |
| YAZDANPARAST SA | 1 | 0.033 | FARRE L | 1 | 0.033 |
| YAUK CL | 1 | 0.033 | FARRAR WL | 1 | 0.033 |
| YATES JR | 1 | 0.033 | FARCI P | 1 | 0.033 |
| YASUI DH | 1 | 0.033 | FANG YP | 1 | 0.033 |
| YARRINGTON RM | 1 | 0.033 | FANG YJ | 1 | 0.033 |
| YARDEN Y | 1 | 0.033 | FANG XY | 1 | 0.033 |
| YAO Z | 1 | 0.033 | FANG XF | 1 | 0.033 |
| YAO YZ | 1 | 0.033 | FANG TT | 1 | 0.033 |
| YAO YY | 1 | 0.033 | FANG S | 1 | 0.033 |
| YAO YT | 1 | 0.033 | FANG RP | 1 | 0.033 |
| YAO YH | 1 | 0.033 | FANG QL | 1 | 0.033 |
| YAO YD | 1 | 0.033 | FANG Q | 1 | 0.033 |
| YAO XD | 1 | 0.033 | FANG H | 1 | 0.033 |
| YAO WY | 1 | 0.033 | FANG FC | 1 | 0.033 |
| YAO WJ | 1 | 0.033 | FANG F | 1 | 0.033 |
| YAO TT | 1 | 0.033 | FANG C | 1 | 0.033 |
| YAO SZ | 1 | 0.033 | FAN ZY | 1 | 0.033 |
| YAO RW | 1 | 0.033 | FAN ZW | 1 | 0.033 |
| YAO QW | 1 | 0.033 | FAN ZS | 1 | 0.033 |
| YAO QL | 1 | 0.033 | FAN ZP | 1 | 0.033 |
| YAO LJ | 1 | 0.033 | FAN ZN | 1 | 0.033 |
| YAO L | 1 | 0.033 | FAN YY | 1 | 0.033 |
| YAO K | 1 | 0.033 | FAN YG | 1 | 0.033 |
| YAO JL | 1 | 0.033 | FAN YF | 1 | 0.033 |
| YAO JB | 1 | 0.033 | FAN YB | 1 | 0.033 |
| YAO HP | 1 | 0.033 | FAN XN | 1 | 0.033 |
| YAO HN | 1 | 0.033 | FAN XL | 1 | 0.033 |
| YAO H | 1 | 0.033 | FAN XG | 1 | 0.033 |
| YAO FY | 1 | 0.033 | FAN XF | 1 | 0.033 |
| YAO CY | 1 | 0.033 | FAN WL | 1 | 0.033 |
| YAO CQ | 1 | 0.033 | FAN WH | 1 | 0.033 |
| YAO C | 1 | 0.033 | FAN TB | 1 | 0.033 |
| YAO BW | 1 | 0.033 | FAN T | 1 | 0.033 |
| YAO B | 1 | 0.033 | FAN RG | 1 | 0.033 |
| YANG ZZ | 1 | 0.033 | FAN QW | 1 | 0.033 |
| YANG ZX | 1 | 0.033 | FAN QS | 1 | 0.033 |
| YANG ZW | 1 | 0.033 | FAN QH | 1 | 0.033 |
| YANG ZT | 1 | 0.033 | FAN PS | 1 | 0.033 |
| YANG ZR | 1 | 0.033 | FAN N | 1 | 0.033 |
| YANG ZP | 1 | 0.033 | FAN M | 1 | 0.033 |
| YANG ZM | 1 | 0.033 | FAN LM | 1 | 0.033 |
| YANG YZ | 1 | 0.033 | FAN LH | 1 | 0.033 |
| YANG YS | 1 | 0.033 | FAN K | 1 | 0.033 |
| YANG YL | 1 | 0.033 | FAN HX | 1 | 0.033 |
| YANG YK | 1 | 0.033 | FAN HN | 1 | 0.033 |
| YANG YG | 1 | 0.033 | FAN DNY | 1 | 0.033 |
| YANG YCT | 1 | 0.033 | FAN DH | 1 | 0.033 |
| YANG YC | 1 | 0.033 | FAN CY | 1 | 0.033 |
| YANG XZ | 1 | 0.033 | FAMIGLIETTI M | 1 | 0.033 |
| YANG XW | 1 | 0.033 | FALZARANO MS | 1 | 0.033 |
| YANG XS | 1 | 0.033 | FALTEJSKOVA-VYCHYTILOVA P | 1 | 0.033 |
| YANG XN | 1 | 0.033 | FALDUM A | 1 | 0.033 |
| YANG XC | 1 | 0.033 | FAIRCHILD L | 1 | 0.033 |
| YANG WC | 1 | 0.033 | FADES G | 1 | 0.033 |
| YANG TW | 1 | 0.033 | FABRIS S | 1 | 0.033 |
| YANG TM | 1 | 0.033 | FABRIS M | 1 | 0.033 |
| YANG TJ | 1 | 0.033 | FABBRI M | 1 | 0.033 |
| YANG TB | 1 | 0.033 | FABBRI G | 1 | 0.033 |
| YANG SZ | 1 | 0.033 | FA-MING T | 1 | 0.033 |
| YANG SY | 1 | 0.033 | EZASHI T | 1 | 0.033 |
| YANG SJ | 1 | 0.033 | EYCKERMAN S | 1 | 0.033 |
| YANG SH | 1 | 0.033 | EVERS M | 1 | 0.033 |
| YANG SB | 1 | 0.033 | EVANS MMS | 1 | 0.033 |
| YANG RX | 1 | 0.033 | EVANS MF | 1 | 0.033 |
| YANG QL | 1 | 0.033 | EVANS CP | 1 | 0.033 |
| YANG PY | 1 | 0.033 | ESTEVE J | 1 | 0.033 |
| YANG PX | 1 | 0.033 | ESTEVA FJ | 1 | 0.033 |
| YANG NN | 1 | 0.033 | ESTEBAN CR | 1 | 0.033 |
| YANG MY | 1 | 0.033 | ESSAWY NOE | 1 | 0.033 |
| YANG MW | 1 | 0.033 | ESQUERRE D | 1 | 0.033 |
| YANG MS | 1 | 0.033 | ESPREAFICO EM | 1 | 0.033 |
| YANG ML | 1 | 0.033 | ESPOSTI DD | 1 | 0.033 |
| YANG LY | 1 | 0.033 | ESPADA J | 1 | 0.033 |
| YANG LX | 1 | 0.033 | ESKO T | 1 | 0.033 |
| YANG LP | 1 | 0.033 | ESHAR S | 1 | 0.033 |
| YANG LL | 1 | 0.033 | ESGUERRA JLS | 1 | 0.033 |
| YANG LB | 1 | 0.033 | ESCODA L | 1 | 0.033 |
| YANG KP | 1 | 0.033 | ESCOBAR TM | 1 | 0.033 |
| YANG KJ | 1 | 0.033 | ESCHENHAGEN T | 1 | 0.033 |
| YANG JR | 1 | 0.033 | ERTEN N | 1 | 0.033 |
| YANG JP | 1 | 0.033 | ERNST C | 1 | 0.033 |
| YANG HT | 1 | 0.033 | ERHARDT S | 1 | 0.033 |
| YANG HM | 1 | 0.033 | ERHARD F | 1 | 0.033 |
| YANG HG | 1 | 0.033 | ERGUN A | 1 | 0.033 |
| YANG HD | 1 | 0.033 | EOH KJ | 1 | 0.033 |
| YANG HB | 1 | 0.033 | ENUKA Y | 1 | 0.033 |
| YANG GX | 1 | 0.033 | ENOKIDA H | 1 | 0.033 |
| YANG GL | 1 | 0.033 | ENGUITA M | 1 | 0.033 |
| YANG GD | 1 | 0.033 | ENGELHARDT S | 1 | 0.033 |
| YANG FF | 1 | 0.033 | ENG C | 1 | 0.033 |
| YANG EC | 1 | 0.033 | ENDO H | 1 | 0.033 |
| YANG CS | 1 | 0.033 | EMST C | 1 | 0.033 |
| YANG CR | 1 | 0.033 | EMMRICH S | 1 | 0.033 |
| YANG CQ | 1 | 0.033 | EMANUELI C | 1 | 0.033 |
| YANG CP | 1 | 0.033 | ELOWITZ MB | 1 | 0.033 |
| YANG BX | 1 | 0.033 | ELLIS MJ | 1 | 0.033 |
| YANG BH | 1 | 0.033 | ELLIS BC | 1 | 0.033 |
| YANG BC | 1 | 0.033 | ELLIOTT O | 1 | 0.033 |
| YANDELL M | 1 | 0.033 | ELLIOTT EN | 1 | 0.033 |
| YANAI I | 1 | 0.033 | ELLIOT G | 1 | 0.033 |
| YANAGISAWA E | 1 | 0.033 | ELLING R | 1 | 0.033 |
| YAN ZM | 1 | 0.033 | ELIASSON L | 1 | 0.033 |
| YAN ZC | 1 | 0.033 | ELDER JT | 1 | 0.033 |
| YAN YW | 1 | 0.033 | ELDE NC | 1 | 0.033 |
| YAN YK | 1 | 0.033 | ELCAVAGE LE | 1 | 0.033 |
| YAN YC | 1 | 0.033 | ELBANNA A | 1 | 0.033 |
| YAN XQ | 1 | 0.033 | ELALOUF JM | 1 | 0.033 |
| YAN XL | 1 | 0.033 | EL-NAKEEP S | 1 | 0.033 |
| YAN XJ | 1 | 0.033 | EISSA S | 1 | 0.033 |
| YAN XH | 1 | 0.033 | EISFELD AK | 1 | 0.033 |
| YAN XF | 1 | 0.033 | EISFELD AJ | 1 | 0.033 |
| YAN XD | 1 | 0.033 | EISCHEN CM | 1 | 0.033 |
| YAN WH | 1 | 0.033 | EICHTEN SR | 1 | 0.033 |
| YAN T | 1 | 0.033 | EICHNER N | 1 | 0.033 |
| YAN SZ | 1 | 0.033 | EGHTESADY P | 1 | 0.033 |
| YAN SX | 1 | 0.033 | EGGAN K | 1 | 0.033 |
| YAN QG | 1 | 0.033 | EGEBLAD M | 1 | 0.033 |
| YAN PS | 1 | 0.033 | EFSTATHIOU E | 1 | 0.033 |
| YAN P | 1 | 0.033 | EFRONI S | 1 | 0.033 |
| YAN K | 1 | 0.033 | EDWARDS SL | 1 | 0.033 |
| YAN JS | 1 | 0.033 | EDWARDS JR | 1 | 0.033 |
| YAN JH | 1 | 0.033 | EDWARDS B | 1 | 0.033 |
| YAN JD | 1 | 0.033 | EDRIS B | 1 | 0.033 |
| YAN JC | 1 | 0.033 | EDKINS S | 1 | 0.033 |
| YAN I | 1 | 0.033 | EDGER PP | 1 | 0.033 |
| YAN HY | 1 | 0.033 | EDDY E | 1 | 0.033 |
| YAN HL | 1 | 0.033 | ECONOMIDES AN | 1 | 0.033 |
| YAN HB | 1 | 0.033 | ECKERSLEY-MASLIN MA | 1 | 0.033 |
| YAN H | 1 | 0.033 | ECHEVERRY N | 1 | 0.033 |
| YAN GQ | 1 | 0.033 | ECHENIQUE J | 1 | 0.033 |
| YAN GG | 1 | 0.033 | EASTLACK SC | 1 | 0.033 |
| YAN G | 1 | 0.033 | EADES G | 1 | 0.033 |
| YAN DL | 1 | 0.033 | DZIKOWSKI R | 1 | 0.033 |
| YAN DH | 1 | 0.033 | DZANANOVIC E | 1 | 0.033 |
| YAN DD | 1 | 0.033 | DYKSTRA-AIELLO C | 1 | 0.033 |
| YAN CGC | 1 | 0.033 | DYCKHOFF G | 1 | 0.033 |
| YAN CF | 1 | 0.033 | DYBKAER K | 1 | 0.033 |
| YAN BC | 1 | 0.033 | DWECK MR | 1 | 0.033 |
| YAMASHITA J | 1 | 0.033 | DURSO M | 1 | 0.033 |
| YAMASHITA H | 1 | 0.033 | DURINCK K | 1 | 0.033 |
| YAMASHITA A | 1 | 0.033 | DURAND D | 1 | 0.033 |
| YAMANO H | 1 | 0.033 | DURAN RCD | 1 | 0.033 |
| YAMANAMI H | 1 | 0.033 | DURAISINGH M | 1 | 0.033 |
| YAMAMURA S | 1 | 0.033 | DUNCANSON A | 1 | 0.033 |
| YAMAMOTO-SUGITANI M | 1 | 0.033 | DUNAGIN MC | 1 | 0.033 |
| YAMAMOTO S | 1 | 0.033 | DUNAGIN M | 1 | 0.033 |
| YAMAMOTO M | 1 | 0.033 | DUGGIMPUDI S | 1 | 0.033 |
| YAMAMOTO F | 1 | 0.033 | DUFOUR N | 1 | 0.033 |
| YAMAMOTO E | 1 | 0.033 | DUESTER G | 1 | 0.033 |
| YAMAGUCHI K | 1 | 0.033 | DUENAS M | 1 | 0.033 |
| YAMADA T | 1 | 0.033 | DUECK A | 1 | 0.033 |
| YAMADA NA | 1 | 0.033 | DUE EU | 1 | 0.033 |
| YAMADA M | 1 | 0.033 | DUDLER R | 1 | 0.033 |
| YAKHINI Z | 1 | 0.033 | DUDEKULA DB | 1 | 0.033 |
| YADA T | 1 | 0.033 | DUARTE R | 1 | 0.033 |
| YACQUB-USMAN K | 1 | 0.033 | DUARTE J | 1 | 0.033 |
| YA-BI Z | 1 | 0.033 | DUAN ZJ | 1 | 0.033 |
| YA SZ | 1 | 0.033 | DUAN Z | 1 | 0.033 |
| XYNOS A | 1 | 0.033 | DUAN XN | 1 | 0.033 |
| XUE-LIANG J | 1 | 0.033 | DUAN WL | 1 | 0.033 |
| XUE ZY | 1 | 0.033 | DUAN WK | 1 | 0.033 |
| XUE ZH | 1 | 0.033 | DUAN LH | 1 | 0.033 |
| XUE YZ | 1 | 0.033 | DUAN JY | 1 | 0.033 |
| XUE YQ | 1 | 0.033 | DUAN JL | 1 | 0.033 |
| XUE YM | 1 | 0.033 | DUAN HW | 1 | 0.033 |
| XUE XY | 1 | 0.033 | DUAN FJ | 1 | 0.033 |
| XUE W | 1 | 0.033 | DUAN CJ | 1 | 0.033 |
| XUE SY | 1 | 0.033 | DU ZW | 1 | 0.033 |
| XUE SL | 1 | 0.033 | DU ZM | 1 | 0.033 |
| XUE S | 1 | 0.033 | DU YN | 1 | 0.033 |
| XUE RL | 1 | 0.033 | DU YE | 1 | 0.033 |
| XUE P | 1 | 0.033 | DU YA | 1 | 0.033 |
| XUE LH | 1 | 0.033 | DU XD | 1 | 0.033 |
| XUE JY | 1 | 0.033 | DU T | 1 | 0.033 |
| XUE JX | 1 | 0.033 | DU SS | 1 | 0.033 |
| XUE JH | 1 | 0.033 | DU QZ | 1 | 0.033 |
| XUE JD | 1 | 0.033 | DU Q | 1 | 0.033 |
| XUE JC | 1 | 0.033 | DU N | 1 | 0.033 |
| XUE J | 1 | 0.033 | DU M | 1 | 0.033 |
| XUE HZ | 1 | 0.033 | DU L | 1 | 0.033 |
| XUE HB | 1 | 0.033 | DU JL | 1 | 0.033 |
| XUE BX | 1 | 0.033 | DU JH | 1 | 0.033 |
| XUAN YW | 1 | 0.033 | DU HJ | 1 | 0.033 |
| XUAN LN | 1 | 0.033 | DU CX | 1 | 0.033 |
| XUAN LJ | 1 | 0.033 | DRONOV S | 1 | 0.033 |
| XUAN HD | 1 | 0.033 | DROGUETT G | 1 | 0.033 |
| XUAN G | 1 | 0.033 | DREW BG | 1 | 0.033 |
| XU ZZ | 1 | 0.033 | DRESCHER KM | 1 | 0.033 |
| XU ZC | 1 | 0.033 | DREPPER F | 1 | 0.033 |
| XU YH | 1 | 0.033 | DRAYTON RM | 1 | 0.033 |
| XU YG | 1 | 0.033 | DRAY E | 1 | 0.033 |
| XU YF | 1 | 0.033 | DRAPKIN RI | 1 | 0.033 |
| XU YC | 1 | 0.033 | DRAKOS SG | 1 | 0.033 |
| XU XY | 1 | 0.033 | DOYLE B | 1 | 0.033 |
| XU XG | 1 | 0.033 | DOWHAN DH | 1 | 0.033 |
| XU XE | 1 | 0.033 | DOU KF | 1 | 0.033 |
| XU XD | 1 | 0.033 | DOU CW | 1 | 0.033 |
| XU XC | 1 | 0.033 | DOU C | 1 | 0.033 |
| XU WT | 1 | 0.033 | DOU BB | 1 | 0.033 |
| XU WR | 1 | 0.033 | DOTTER CP | 1 | 0.033 |
| XU SZ | 1 | 0.033 | DOS REMEDIOS CG | 1 | 0.033 |
| XU SY | 1 | 0.033 | DORING F | 1 | 0.033 |
| XU SS | 1 | 0.033 | DORI M | 1 | 0.033 |
| XU SQ | 1 | 0.033 | DOPAZO J | 1 | 0.033 |
| XU SC | 1 | 0.033 | DOOSE G | 1 | 0.033 |
| XU RH | 1 | 0.033 | DONNELLY P | 1 | 0.033 |
| XU RG | 1 | 0.033 | DONNELLY LE | 1 | 0.033 |
| XU RA | 1 | 0.033 | DONIZETTI A | 1 | 0.033 |
| XU R | 1 | 0.033 | DONIGER T | 1 | 0.033 |
| XU QQ | 1 | 0.033 | DONG YZ | 1 | 0.033 |
| XU PC | 1 | 0.033 | DONG YQ | 1 | 0.033 |
| XU NY | 1 | 0.033 | DONG YH | 1 | 0.033 |
| XU NW | 1 | 0.033 | DONG YC | 1 | 0.033 |
| XU NB | 1 | 0.033 | DONG XZ | 1 | 0.033 |
| XU MY | 1 | 0.033 | DONG XY | 1 | 0.033 |
| XU MQ | 1 | 0.033 | DONG XS | 1 | 0.033 |
| XU LX | 1 | 0.033 | DONG XR | 1 | 0.033 |
| XU LT | 1 | 0.033 | DONG XM | 1 | 0.033 |
| XU LQ | 1 | 0.033 | DONG XG | 1 | 0.033 |
| XU LN | 1 | 0.033 | DONG W | 1 | 0.033 |
| XU LL | 1 | 0.033 | DONG TY | 1 | 0.033 |
| XU LJ | 1 | 0.033 | DONG SW | 1 | 0.033 |
| XU LF | 1 | 0.033 | DONG RZ | 1 | 0.033 |
| XU KP | 1 | 0.033 | DONG RF | 1 | 0.033 |
| XU KL | 1 | 0.033 | DONG PDS | 1 | 0.033 |
| XU JJ | 1 | 0.033 | DONG NZ | 1 | 0.033 |
| XU HZ | 1 | 0.033 | DONG MH | 1 | 0.033 |
| XU HY | 1 | 0.033 | DONG M | 1 | 0.033 |
| XU HT | 1 | 0.033 | DONG LY | 1 | 0.033 |
| XU HG | 1 | 0.033 | DONG LP | 1 | 0.033 |
| XU GZ | 1 | 0.033 | DONG LL | 1 | 0.033 |
| XU GX | 1 | 0.033 | DONG LF | 1 | 0.033 |
| XU GQ | 1 | 0.033 | DONG K | 1 | 0.033 |
| XU GH | 1 | 0.033 | DONG JH | 1 | 0.033 |
| XU FY | 1 | 0.033 | DONG H | 1 | 0.033 |
| XU FJ | 1 | 0.033 | DONG FS | 1 | 0.033 |
| XU FF | 1 | 0.033 | DONG FL | 1 | 0.033 |
| XU F | 1 | 0.033 | DONG D | 1 | 0.033 |
| XU EYJ | 1 | 0.033 | DONG CY | 1 | 0.033 |
| XU EP | 1 | 0.033 | DONG BW | 1 | 0.033 |
| XU DZ | 1 | 0.033 | DONG BB | 1 | 0.033 |
| XU DQ | 1 | 0.033 | DONALDSON CJ | 1 | 0.033 |
| XU DK | 1 | 0.033 | DONAKONDA S | 1 | 0.033 |
| XU DH | 1 | 0.033 | DOMINGUEZ M | 1 | 0.033 |
| XU DF | 1 | 0.033 | DOMINGOS AI | 1 | 0.033 |
| XU CZ | 1 | 0.033 | DOLITZKY A | 1 | 0.033 |
| XU CQ | 1 | 0.033 | DOLEZEL J | 1 | 0.033 |
| XU CG | 1 | 0.033 | DOLD A | 1 | 0.033 |
| XU CF | 1 | 0.033 | DODSON MV | 1 | 0.033 |
| XU CA | 1 | 0.033 | DODDABALLAPUR A | 1 | 0.033 |
| XU BN | 1 | 0.033 | DOBSON-STONE C | 1 | 0.033 |
| XU BM | 1 | 0.033 | DOBROFF AS | 1 | 0.033 |
| XU BJ | 1 | 0.033 | DO KA | 1 | 0.033 |
| XU BH | 1 | 0.033 | DO BT | 1 | 0.033 |
| XU AT | 1 | 0.033 | DKHIL MA | 1 | 0.033 |
| XIU JJ | 1 | 0.033 | DJEBALI S | 1 | 0.033 |
| XIU JC | 1 | 0.033 | DJAVSAROV I | 1 | 0.033 |
| XIONG ZR | 1 | 0.033 | DJARI A | 1 | 0.033 |
| XIONG Z | 1 | 0.033 | DJANGMAH HS | 1 | 0.033 |
| XIONG YQ | 1 | 0.033 | DIXON RAF | 1 | 0.033 |
| XIONG WD | 1 | 0.033 | DIVOUX A | 1 | 0.033 |
| XIONG TL | 1 | 0.033 | DIVER L | 1 | 0.033 |
| XIONG SD | 1 | 0.033 | DITTWALD P | 1 | 0.033 |
| XIONG Q | 1 | 0.033 | DITTMER TA | 1 | 0.033 |
| XIONG P | 1 | 0.033 | DITS N | 1 | 0.033 |
| XIONG MQ | 1 | 0.033 | DISTEL RJ | 1 | 0.033 |
| XIONG MM | 1 | 0.033 | DISKIN SJ | 1 | 0.033 |
| XIONG M | 1 | 0.033 | DIRICAN A | 1 | 0.033 |
| XIONG L | 1 | 0.033 | DING YZ | 1 | 0.033 |
| XIONG K | 1 | 0.033 | DING YQ | 1 | 0.033 |
| XIONG JH | 1 | 0.033 | DING YC | 1 | 0.033 |
| XIONG JG | 1 | 0.033 | DING WQ | 1 | 0.033 |
| XIONG DD | 1 | 0.033 | DING WP | 1 | 0.033 |
| XING YF | 1 | 0.033 | DING R | 1 | 0.033 |
| XING XX | 1 | 0.033 | DING QS | 1 | 0.033 |
| XING R | 1 | 0.033 | DING P | 1 | 0.033 |
| XING J | 1 | 0.033 | DING MH | 1 | 0.033 |
| XING GF | 1 | 0.033 | DING LY | 1 | 0.033 |
| XING D | 1 | 0.033 | DING LW | 1 | 0.033 |
| XING CY | 1 | 0.033 | DING LL | 1 | 0.033 |
| XING CL | 1 | 0.033 | DING LJ | 1 | 0.033 |
| XING CE | 1 | 0.033 | DING K | 1 | 0.033 |
| XIN XR | 1 | 0.033 | DING JY | 1 | 0.033 |
| XIN WJ | 1 | 0.033 | DING JW | 1 | 0.033 |
| XIN WF | 1 | 0.033 | DING JH | 1 | 0.033 |
| XIN SM | 1 | 0.033 | DING HY | 1 | 0.033 |
| XIN LL | 1 | 0.033 | DING GW | 1 | 0.033 |
| XIN L | 1 | 0.033 | DING GH | 1 | 0.033 |
| XIN D | 1 | 0.033 | DING G | 1 | 0.033 |
| XIE ZY | 1 | 0.033 | DING FZ | 1 | 0.033 |
| XIE ZQ | 1 | 0.033 | DING FB | 1 | 0.033 |
| XIE YW | 1 | 0.033 | DING CL | 1 | 0.033 |
| XIE YS | 1 | 0.033 | DING CH | 1 | 0.033 |
| XIE YR | 1 | 0.033 | DINARTE-SANTOS AR | 1 | 0.033 |
| XIE YP | 1 | 0.033 | DIMON M | 1 | 0.033 |
| XIE YJ | 1 | 0.033 | DIMITROVA N | 1 | 0.033 |
| XIE XX | 1 | 0.033 | DIMITROVA L | 1 | 0.033 |
| XIE XW | 1 | 0.033 | DIMITROULAKOS J | 1 | 0.033 |
| XIE XJ | 1 | 0.033 | DIGHE SN | 1 | 0.033 |
| XIE XH | 1 | 0.033 | DIEZ-FRAILE A | 1 | 0.033 |
| XIE XD | 1 | 0.033 | DIERMEIER S | 1 | 0.033 |
| XIE WX | 1 | 0.033 | DIENEMANN H | 1 | 0.033 |
| XIE WQ | 1 | 0.033 | DIELI F | 1 | 0.033 |
| XIE WP | 1 | 0.033 | DICCIANNI MB | 1 | 0.033 |
| XIE WL | 1 | 0.033 | DIAZ-BEYA M | 1 | 0.033 |
| XIE TC | 1 | 0.033 | DIAZ AA | 1 | 0.033 |
| XIE T | 1 | 0.033 | DIAZ A | 1 | 0.033 |
| XIE SH | 1 | 0.033 | DIATCHENKO L | 1 | 0.033 |
| XIE SG | 1 | 0.033 | DIAS-NETO E | 1 | 0.033 |
| XIE P | 1 | 0.033 | DIAS SMG | 1 | 0.033 |
| XIE N | 1 | 0.033 | DIAO ZY | 1 | 0.033 |
| XIE MJ | 1 | 0.033 | DIAO RY | 1 | 0.033 |
| XIE LY | 1 | 0.033 | DIAO LX | 1 | 0.033 |
| XIE LW | 1 | 0.033 | DIAO JX | 1 | 0.033 |
| XIE LS | 1 | 0.033 | DIAO HY | 1 | 0.033 |
| XIE LG | 1 | 0.033 | DIAMOND M | 1 | 0.033 |
| XIE KP | 1 | 0.033 | DIALLO AB | 1 | 0.033 |
| XIE JB | 1 | 0.033 | DI ZHOU | 1 | 0.033 |
| XIE J | 1 | 0.033 | DI WY | 1 | 0.033 |
| XIE HL | 1 | 0.033 | DI SILVESTRE A | 1 | 0.033 |
| XIE GZ | 1 | 0.033 | DI SALVO TG | 1 | 0.033 |
| XIE GC | 1 | 0.033 | DI RAIMONDO F | 1 | 0.033 |
| XIE FY | 1 | 0.033 | DI GESUALDO F | 1 | 0.033 |
| XIE DY | 1 | 0.033 | DI CROCE L | 1 | 0.033 |
| XIE D | 1 | 0.033 | DI CECILIA S | 1 | 0.033 |
| XIE CQ | 1 | 0.033 | DI CARLO V | 1 | 0.033 |
| XIE CH | 1 | 0.033 | DHOONDIA Z | 1 | 0.033 |
| XIE C | 1 | 0.033 | DHIR S | 1 | 0.033 |
| XIE BS | 1 | 0.033 | DHIR A | 1 | 0.033 |
| XIE BJ | 1 | 0.033 | DHIMAN H | 1 | 0.033 |
| XIE BB | 1 | 0.033 | DHANDAPANI V | 1 | 0.033 |
| XIAO-PO L | 1 | 0.033 | DEVISETTY UK | 1 | 0.033 |
| XIAO-LI L | 1 | 0.033 | DEVAUX RS | 1 | 0.033 |
| XIAO ZZ | 1 | 0.033 | DEVARAJ S | 1 | 0.033 |
| XIAO ZM | 1 | 0.033 | DEUTSCH G | 1 | 0.033 |
| XIAO YS | 1 | 0.033 | DESHPANDE S | 1 | 0.033 |
| XIAO YJ | 1 | 0.033 | DESETTY RD | 1 | 0.033 |
| XIAO XK | 1 | 0.033 | DESCRIMES M | 1 | 0.033 |
| XIAO XF | 1 | 0.033 | DESCAMPS B | 1 | 0.033 |
| XIAO WJ | 1 | 0.033 | DESAI K | 1 | 0.033 |
| XIAO WF | 1 | 0.033 | DERVEAUX S | 1 | 0.033 |
| XIAO WB | 1 | 0.033 | DERMITZAKIS ET | 1 | 0.033 |
| XIAO T | 1 | 0.033 | DERDA AA | 1 | 0.033 |
| XIAO SZ | 1 | 0.033 | DEOBAGKAR DD | 1 | 0.033 |
| XIAO PF | 1 | 0.033 | DEO SR | 1 | 0.033 |
| XIAO P | 1 | 0.033 | DENOYELLE C | 1 | 0.033 |
| XIAO M | 1 | 0.033 | DENNISON JB | 1 | 0.033 |
| XIAO LW | 1 | 0.033 | DENNIS ES | 1 | 0.033 |
| XIAO LL | 1 | 0.033 | DENG ZL | 1 | 0.033 |
| XIAO LJ | 1 | 0.033 | DENG ZG | 1 | 0.033 |
| XIAO LB | 1 | 0.033 | DENG YX | 1 | 0.033 |
| XIAO JJ | 1 | 0.033 | DENG YT | 1 | 0.033 |
| XIAO JH | 1 | 0.033 | DENG YM | 1 | 0.033 |
| XIAO HM | 1 | 0.033 | DENG XZ | 1 | 0.033 |
| XIAO HL | 1 | 0.033 | DENG XY | 1 | 0.033 |
| XIAO HJ | 1 | 0.033 | DENG XH | 1 | 0.033 |
| XIAO F | 1 | 0.033 | DENG X | 1 | 0.033 |
| XIAO DM | 1 | 0.033 | DENG WY | 1 | 0.033 |
| XIAO D | 1 | 0.033 | DENG WL | 1 | 0.033 |
| XIANG Z | 1 | 0.033 | DENG WJ | 1 | 0.033 |
| XIANG YQ | 1 | 0.033 | DENG WH | 1 | 0.033 |
| XIANG XX | 1 | 0.033 | DENG TB | 1 | 0.033 |
| XIANG TX | 1 | 0.033 | DENG SQ | 1 | 0.033 |
| XIANG T | 1 | 0.033 | DENG SJ | 1 | 0.033 |
| XIANG SL | 1 | 0.033 | DENG SC | 1 | 0.033 |
| XIANG K | 1 | 0.033 | DENG RL | 1 | 0.033 |
| XIANG JP | 1 | 0.033 | DENG R | 1 | 0.033 |
| XIANG J | 1 | 0.033 | DENG LM | 1 | 0.033 |
| XIANG HF | 1 | 0.033 | DENG LC | 1 | 0.033 |
| XIANG H | 1 | 0.033 | DENG KQ | 1 | 0.033 |
| XIANG DM | 1 | 0.033 | DENG KL | 1 | 0.033 |
| XIANG C | 1 | 0.033 | DENG JY | 1 | 0.033 |
| XIA ZJ | 1 | 0.033 | DENG HY | 1 | 0.033 |
| XIA YF | 1 | 0.033 | DENG HX | 1 | 0.033 |
| XIA XW | 1 | 0.033 | DENG HP | 1 | 0.033 |
| XIA XM | 1 | 0.033 | DENG DN | 1 | 0.033 |
| XIA XL | 1 | 0.033 | DENECKE B | 1 | 0.033 |
| XIA WK | 1 | 0.033 | DEN BOER ML | 1 | 0.033 |
| XIA SW | 1 | 0.033 | DEMICHELIS F | 1 | 0.033 |
| XIA SJ | 1 | 0.033 | DELPRETTI S | 1 | 0.033 |
| XIA SH | 1 | 0.033 | DELOUKAS P | 1 | 0.033 |
| XIA QY | 1 | 0.033 | DELKER D | 1 | 0.033 |
| XIA QQ | 1 | 0.033 | DELIC D | 1 | 0.033 |
| XIA Q | 1 | 0.033 | DELGADO RN | 1 | 0.033 |
| XIA PY | 1 | 0.033 | DELGADILLO DM | 1 | 0.033 |
| XIA P | 1 | 0.033 | DELABESSE E | 1 | 0.033 |
| XIA MY | 1 | 0.033 | DEL PESO L | 1 | 0.033 |
| XIA M | 1 | 0.033 | DEL CORNO M | 1 | 0.033 |
| XIA LQ | 1 | 0.033 | DEKKER J | 1 | 0.033 |
| XIA L | 1 | 0.033 | DEKAY J | 1 | 0.033 |
| XIA K | 1 | 0.033 | DEJEAN A | 1 | 0.033 |
| XIA JX | 1 | 0.033 | DEHIPAWALA S | 1 | 0.033 |
| XIA JH | 1 | 0.033 | DEGREGORIO SJ | 1 | 0.033 |
| XIA JF | 1 | 0.033 | DEGNAN BM | 1 | 0.033 |
| XIA HY | 1 | 0.033 | DEGLON N | 1 | 0.033 |
| XIA HW | 1 | 0.033 | DEGIRMENCI U | 1 | 0.033 |
| XIA F | 1 | 0.033 | DEFORCE D | 1 | 0.033 |
| XIA DJ | 1 | 0.033 | DEENEN R | 1 | 0.033 |
| XIA D | 1 | 0.033 | DECORTI G | 1 | 0.033 |
| XIA BR | 1 | 0.033 | DEBBANEH M | 1 | 0.033 |
| XIA B | 1 | 0.033 | DEAN C | 1 | 0.033 |
| XI YY | 1 | 0.033 | DE WERKEN HJGV | 1 | 0.033 |
| XI YX | 1 | 0.033 | DE WEERD V | 1 | 0.033 |
| XI YG | 1 | 0.033 | DE VIVO I | 1 | 0.033 |
| XI P | 1 | 0.033 | DE VILLENA FPM | 1 | 0.033 |
| XI MR | 1 | 0.033 | DE VERA IMS | 1 | 0.033 |
| XI ML | 1 | 0.033 | DE TOMA I | 1 | 0.033 |
| XI M | 1 | 0.033 | DE SPIEGELAERE W | 1 | 0.033 |
| XI L | 1 | 0.033 | DE SOUZA JES | 1 | 0.033 |
| XI JL | 1 | 0.033 | DE SOUZA CA | 1 | 0.033 |
| XI JJ | 1 | 0.033 | DE PAULA W | 1 | 0.033 |
| XI HF | 1 | 0.033 | DE OLIVEIRA JF | 1 | 0.033 |
| XI BR | 1 | 0.033 | DE MOLFETTA GA | 1 | 0.033 |
| XENARIOS I | 1 | 0.033 | DE MOERLOOSE B | 1 | 0.033 |
| WUPUTRA K | 1 | 0.033 | DE MEYER T | 1 | 0.033 |
| WUNDERLICH F | 1 | 0.033 | DE LUCIA F | 1 | 0.033 |
| WU ZY | 1 | 0.033 | DE LLANO MPQ | 1 | 0.033 |
| WU ZM | 1 | 0.033 | DE LEO G | 1 | 0.033 |
| WU ZL | 1 | 0.033 | DE LAGRAN IAM | 1 | 0.033 |
| WU ZJ | 1 | 0.033 | DE LAAT W | 1 | 0.033 |
| WU ZH | 1 | 0.033 | DE LA ROSA F | 1 | 0.033 |
| WU ZC | 1 | 0.033 | DE LA FUENTE R | 1 | 0.033 |
| WU YW | 1 | 0.033 | DE KLEIN A | 1 | 0.033 |
| WU YK | 1 | 0.033 | DE IUDICIBUS S | 1 | 0.033 |
| WU YD | 1 | 0.033 | DE GROOTE P | 1 | 0.033 |
| WU YC | 1 | 0.033 | DE GONZALO-CALVO D | 1 | 0.033 |
| WU XQ | 1 | 0.033 | DE FREITAS PP | 1 | 0.033 |
| WU XJ | 1 | 0.033 | DE FINO C | 1 | 0.033 |
| WU XH | 1 | 0.033 | DE COBELLI O | 1 | 0.033 |
| WU XG | 1 | 0.033 | DE CHALDEE M | 1 | 0.033 |
| WU WD | 1 | 0.033 | DE CANDIA P | 1 | 0.033 |
| WU TQ | 1 | 0.033 | DE CABO R | 1 | 0.033 |
| WU TF | 1 | 0.033 | DE BRUIJN E | 1 | 0.033 |
| WU TC | 1 | 0.033 | DE BORTOLI M | 1 | 0.033 |
| WU SS | 1 | 0.033 | DE BLESER P | 1 | 0.033 |
| WU SP | 1 | 0.033 | DE ARAUJO LF | 1 | 0.033 |
| WU SH | 1 | 0.033 | DE ALMEIDA RC | 1 | 0.033 |
| WU S | 1 | 0.033 | DAY YJ | 1 | 0.033 |
| WU RF | 1 | 0.033 | DAVIS CJ | 1 | 0.033 |
| WU QW | 1 | 0.033 | DAVIS CA | 1 | 0.033 |
| WU QL | 1 | 0.033 | DAVILA A | 1 | 0.033 |
| WU QH | 1 | 0.033 | DAVIES SMK | 1 | 0.033 |
| WU QF | 1 | 0.033 | DAVIES MA | 1 | 0.033 |
| WU PC | 1 | 0.033 | DAVIES KE | 1 | 0.033 |
| WU MS | 1 | 0.033 | DAVIES JOJ | 1 | 0.033 |
| WU MR | 1 | 0.033 | DAVIDSON I | 1 | 0.033 |
| WU MA | 1 | 0.033 | DAVIDOVICH C | 1 | 0.033 |
| WU LY | 1 | 0.033 | DAVE RK | 1 | 0.033 |
| WU LQ | 1 | 0.033 | DAUVILLIER J | 1 | 0.033 |
| WU LL | 1 | 0.033 | DATTA S | 1 | 0.033 |
| WU LJ | 1 | 0.033 | DASILVA LF | 1 | 0.033 |
| WU KM | 1 | 0.033 | DASHWOOD RH | 1 | 0.033 |
| WU JT | 1 | 0.033 | DARROW EM | 1 | 0.033 |
| WU JN | 1 | 0.033 | DARLINGTON GJ | 1 | 0.033 |
| WU HZ | 1 | 0.033 | DANIS J | 1 | 0.033 |
| WU HT | 1 | 0.033 | DANG Z | 1 | 0.033 |
| WU HS | 1 | 0.033 | DANG YH | 1 | 0.033 |
| WU HD | 1 | 0.033 | DANG YA | 1 | 0.033 |
| WU GN | 1 | 0.033 | DANG Q | 1 | 0.033 |
| WU GC | 1 | 0.033 | DANG JS | 1 | 0.033 |
| WU FT | 1 | 0.033 | DANG HX | 1 | 0.033 |
| WU FS | 1 | 0.033 | DANG H | 1 | 0.033 |
| WU FP | 1 | 0.033 | DANESHVAR K | 1 | 0.033 |
| WU FL | 1 | 0.033 | DANESH FR | 1 | 0.033 |
| WU FF | 1 | 0.033 | DAN S | 1 | 0.033 |
| WU DL | 1 | 0.033 | DAMSKI C | 1 | 0.033 |
| WU DH | 1 | 0.033 | DAMNON F | 1 | 0.033 |
| WU CX | 1 | 0.033 | DAMLE SS | 1 | 0.033 |
| WU CW | 1 | 0.033 | DAMBACHER S | 1 | 0.033 |
| WU BY | 1 | 0.033 | DALTON S | 1 | 0.033 |
| WU BT | 1 | 0.033 | DALMOLIN RJS | 1 | 0.033 |
| WU BL | 1 | 0.033 | DALLONGEVILLE AS | 1 | 0.033 |
| WU BH | 1 | 0.033 | DALLA-FAVERA R | 1 | 0.033 |
| WU AW | 1 | 0.033 | DALEY GQ | 1 | 0.033 |
| WU AM | 1 | 0.033 | DALAMAGAS TM | 1 | 0.033 |
| WRIGHT RO | 1 | 0.033 | DALAL Y | 1 | 0.033 |
| WRIGHT RJ | 1 | 0.033 | DAKIN R | 1 | 0.033 |
| WRIGHT PW | 1 | 0.033 | DAISH T | 1 | 0.033 |
| WOUTERS J | 1 | 0.033 | DAIGO Y | 1 | 0.033 |
| WORLE H | 1 | 0.033 | DAI-LAN | 1 | 0.033 |
| WOODSIDE DG | 1 | 0.033 | DAI ZX | 1 | 0.033 |
| WOOD WH | 1 | 0.033 | DAI Z | 1 | 0.033 |
| WOOD NW | 1 | 0.033 | DAI XY | 1 | 0.033 |
| WOOD MJA | 1 | 0.033 | DAI XN | 1 | 0.033 |
| WOOD J | 1 | 0.033 | DAI WG | 1 | 0.033 |
| WOOD EJ | 1 | 0.033 | DAI QX | 1 | 0.033 |
| WOOD E | 1 | 0.033 | DAI QH | 1 | 0.033 |
| WONGTRAKOONGATE P | 1 | 0.033 | DAI LP | 1 | 0.033 |
| WONGPALEE SP | 1 | 0.033 | DAI JW | 1 | 0.033 |
| WONG STS | 1 | 0.033 | DAI JC | 1 | 0.033 |
| WONG SS | 1 | 0.033 | DAI HY | 1 | 0.033 |
| WONG PTH | 1 | 0.033 | DAI HG | 1 | 0.033 |
| WONG N | 1 | 0.033 | DAI F | 1 | 0.033 |
| WONG L | 1 | 0.033 | DAI EY | 1 | 0.033 |
| WONG DJ | 1 | 0.033 | DAI DW | 1 | 0.033 |
| WONG CW | 1 | 0.033 | DAI CC | 1 | 0.033 |
| WONG CP | 1 | 0.033 | DAI BH | 1 | 0.033 |
| WONG CM | 1 | 0.033 | DAHL A | 1 | 0.033 |
| WONG CCL | 1 | 0.033 | DAHA N | 1 | 0.033 |
| WON SY | 1 | 0.033 | DA SILVA D | 1 | 0.033 |
| WOLL P | 1 | 0.033 | DA J | 1 | 0.033 |
| WOLF AR | 1 | 0.033 | D'HONT I | 1 | 0.033 |
| WOLD BJ | 1 | 0.033 | D'HERDE K | 1 | 0.033 |
| WOJCIECH F | 1 | 0.033 | D'HAENE E | 1 | 0.033 |
| WOHLER CM | 1 | 0.033 | D'ECCLESSIS MF | 1 | 0.033 |
| WOEHLE C | 1 | 0.033 | D'ANDREA D | 1 | 0.033 |
| WITTFELDT A | 1 | 0.033 | D'ALESSANDRA Y | 1 | 0.033 |
| WITTEN DM | 1 | 0.033 | CZERNIAK B | 1 | 0.033 |
| WITTE DP | 1 | 0.033 | CUTRUPI S | 1 | 0.033 |
| WISNIEWSKI M | 1 | 0.033 | CUTRONA G | 1 | 0.033 |
| WISKOW O | 1 | 0.033 | CUSSENOT O | 1 | 0.033 |
| WISE JF | 1 | 0.033 | CURTIS J | 1 | 0.033 |
| WINTER P | 1 | 0.033 | CURRAN M | 1 | 0.033 |
| WINDHORST S | 1 | 0.033 | CUPPEN E | 1 | 0.033 |
| WINDERS BR | 1 | 0.033 | CUNNINGHAM TJ | 1 | 0.033 |
| WILUSZ J | 1 | 0.033 | CUNHA-NETO E | 1 | 0.033 |
| WILUSZ CJ | 1 | 0.033 | CUI YY | 1 | 0.033 |
| WILSON MD | 1 | 0.033 | CUI YQ | 1 | 0.033 |
| WILSON KD | 1 | 0.033 | CUI YH | 1 | 0.033 |
| WILSON J | 1 | 0.033 | CUI XY | 1 | 0.033 |
| WILSON GW | 1 | 0.033 | CUI XQ | 1 | 0.033 |
| WILSON GM | 1 | 0.033 | CUI XP | 1 | 0.033 |
| WILMING L | 1 | 0.033 | CUI XM | 1 | 0.033 |
| WILLIAMS TN | 1 | 0.033 | CUI WR | 1 | 0.033 |
| WILLIAMS SEI | 1 | 0.033 | CUI T | 1 | 0.033 |
| WILLIAMS RW | 1 | 0.033 | CUI SZ | 1 | 0.033 |
| WILLIAMS RT | 1 | 0.033 | CUI SP | 1 | 0.033 |
| WILLIAMS JM | 1 | 0.033 | CUI N | 1 | 0.033 |
| WILLIAMS DE | 1 | 0.033 | CUI MX | 1 | 0.033 |
| WILLIAMS C | 1 | 0.033 | CUI MH | 1 | 0.033 |
| WILLIAMS B | 1 | 0.033 | CUI JD | 1 | 0.033 |
| WILLERSON JT | 1 | 0.033 | CUI HM | 1 | 0.033 |
| WILLE A | 1 | 0.033 | CUI HC | 1 | 0.033 |
| WILKINSON JE | 1 | 0.033 | CUI H | 1 | 0.033 |
| WILK R | 1 | 0.033 | CUI FA | 1 | 0.033 |
| WILEY MW | 1 | 0.033 | CUI F | 1 | 0.033 |
| WILEY KE | 1 | 0.033 | CUI B | 1 | 0.033 |
| WIJNHOVEN PWG | 1 | 0.033 | CRYAN SA | 1 | 0.033 |
| WIJCHERS PJ | 1 | 0.033 | CRUJEIRAS AB | 1 | 0.033 |
| WIEWRODT R | 1 | 0.033 | CROYAL M | 1 | 0.033 |
| WIENS GD | 1 | 0.033 | CROSTHWAITE SK | 1 | 0.033 |
| WIENER HW | 1 | 0.033 | CROOKS GM | 1 | 0.033 |
| WIEMANN S | 1 | 0.033 | CROMPTON BD | 1 | 0.033 |
| WIDSCHWENDTER M | 1 | 0.033 | CROCCI-SOUZA R | 1 | 0.033 |
| WIDGER WR | 1 | 0.033 | CRINO L | 1 | 0.033 |
| WIDAGDO J | 1 | 0.033 | CRESPI M | 1 | 0.033 |
| WIBRAND K | 1 | 0.033 | CREO P | 1 | 0.033 |
| WIBERG RAW | 1 | 0.033 | CREMASCHI P | 1 | 0.033 |
| WHITESIDE EJ | 1 | 0.033 | CREEMERS EE | 1 | 0.033 |
| WHITEHOUSE A | 1 | 0.033 | CRAWFORD GE | 1 | 0.033 |
| WHITEHEAD RH | 1 | 0.033 | CRARY FK | 1 | 0.033 |
| WHITEHEAD J | 1 | 0.033 | CRAPPER L | 1 | 0.033 |
| WHITE NM | 1 | 0.033 | COX A | 1 | 0.033 |
| WHITE FV | 1 | 0.033 | COWLEY AW | 1 | 0.033 |
| WHITE EJ | 1 | 0.033 | COWAN SL | 1 | 0.033 |
| WHITE D | 1 | 0.033 | COURT F | 1 | 0.033 |
| WHIGHAM BT | 1 | 0.033 | COUPLAND KG | 1 | 0.033 |
| WHERRY EJ | 1 | 0.033 | COUPEAU D | 1 | 0.033 |
| WHEELER J | 1 | 0.033 | COULSON RL | 1 | 0.033 |
| WETZLER M | 1 | 0.033 | COUKOS G | 1 | 0.033 |
| WESTRA HJ | 1 | 0.033 | COTELLA D | 1 | 0.033 |
| WESTON S | 1 | 0.033 | COTE I | 1 | 0.033 |
| WESTERLUND F | 1 | 0.033 | COSTINEAN S | 1 | 0.033 |
| WEST R | 1 | 0.033 | COSTES SV | 1 | 0.033 |
| WEST JA | 1 | 0.033 | COSTA V | 1 | 0.033 |
| WESSENDORF E | 1 | 0.033 | COSTA IG | 1 | 0.033 |
| WESPISER AR | 1 | 0.033 | COSTA FF | 1 | 0.033 |
| WERY M | 1 | 0.033 | CORVIN A | 1 | 0.033 |
| WERNER T | 1 | 0.033 | CORTELEZZI A | 1 | 0.033 |
| WENIGER MA | 1 | 0.033 | CORREARD S | 1 | 0.033 |
| WENG ZL | 1 | 0.033 | CORRADIN O | 1 | 0.033 |
| WENG WK | 1 | 0.033 | CORNELIS G | 1 | 0.033 |
| WENG JK | 1 | 0.033 | CORDERO F | 1 | 0.033 |
| WENG GB | 1 | 0.033 | CORCORAN M | 1 | 0.033 |
| WEN ZS | 1 | 0.033 | CORCES-ZIMMERMAN R | 1 | 0.033 |
| WEN ZM | 1 | 0.033 | COPPOLA G | 1 | 0.033 |
| WEN ZL | 1 | 0.033 | COOPER SE | 1 | 0.033 |
| WEN XT | 1 | 0.033 | COOPER DR | 1 | 0.033 |
| WEN XS | 1 | 0.033 | COON SL | 1 | 0.033 |
| WEN WH | 1 | 0.033 | COOLS J | 1 | 0.033 |
| WEN SX | 1 | 0.033 | COOK S | 1 | 0.033 |
| WEN SJ | 1 | 0.033 | CONN GL | 1 | 0.033 |
| WEN S | 1 | 0.033 | CONIGLIARO A | 1 | 0.033 |
| WEN QR | 1 | 0.033 | CONG PY | 1 | 0.033 |
| WEN Q | 1 | 0.033 | CONG ML | 1 | 0.033 |
| WEN LJ | 1 | 0.033 | CONG H | 1 | 0.033 |
| WEN KJ | 1 | 0.033 | CONESA A | 1 | 0.033 |
| WEN JH | 1 | 0.033 | CONDINO-NETO A | 1 | 0.033 |
| WEN JF | 1 | 0.033 | COMPERAT E | 1 | 0.033 |
| WEN DY | 1 | 0.033 | COLONNA V | 1 | 0.033 |
| WEN B | 1 | 0.033 | COLOMBO M | 1 | 0.033 |
| WELSH P | 1 | 0.033 | COLOMBATTI A | 1 | 0.033 |
| WEISSMAN JS | 1 | 0.033 | COLOGNORI D | 1 | 0.033 |
| WEISS M | 1 | 0.033 | COLLIER SP | 1 | 0.033 |
| WEIRATHER J | 1 | 0.033 | COLLET MA | 1 | 0.033 |
| WEINSHILBOUM RM | 1 | 0.033 | COLI C | 1 | 0.033 |
| WEINHOLD E | 1 | 0.033 | COLE RA | 1 | 0.033 |
| WEIDEMANN F | 1 | 0.033 | COLE KA | 1 | 0.033 |
| WEICHENHAN D | 1 | 0.033 | COLE FS | 1 | 0.033 |
| WEI ZL | 1 | 0.033 | COLANTONI A | 1 | 0.033 |
| WEI ZF | 1 | 0.033 | COITO C | 1 | 0.033 |
| WEI YZ | 1 | 0.033 | COHEN O | 1 | 0.033 |
| WEI YY | 1 | 0.033 | COFFEY RJ | 1 | 0.033 |
| WEI YX | 1 | 0.033 | COE EA | 1 | 0.033 |
| WEI YQ | 1 | 0.033 | COCHRANE J | 1 | 0.033 |
| WEI YP | 1 | 0.033 | COBO-STARK P | 1 | 0.033 |
| WEI YK | 1 | 0.033 | COBB BS | 1 | 0.033 |
| WEI YB | 1 | 0.033 | CLOKIE SJH | 1 | 0.033 |
| WEI XZ | 1 | 0.033 | CLIFT D | 1 | 0.033 |
| WEI XR | 1 | 0.033 | CLERMONT PL | 1 | 0.033 |
| WEI XQ | 1 | 0.033 | CLEARY C | 1 | 0.033 |
| WEI XL | 1 | 0.033 | CLAY I | 1 | 0.033 |
| WEI XK | 1 | 0.033 | CLAVIEZ A | 1 | 0.033 |
| WEI X | 1 | 0.033 | CLAUSEN DS | 1 | 0.033 |
| WEI WS | 1 | 0.033 | CLARK T | 1 | 0.033 |
| WEI SY | 1 | 0.033 | CLARK MF | 1 | 0.033 |
| WEI SJ | 1 | 0.033 | CIUFFI A | 1 | 0.033 |
| WEI QY | 1 | 0.033 | CITARELLA F | 1 | 0.033 |
| WEI P | 1 | 0.033 | CIPRIANO A | 1 | 0.033 |
| WEI MM | 1 | 0.033 | CINELLI P | 1 | 0.033 |
| WEI LX | 1 | 0.033 | CIMMINO A | 1 | 0.033 |
| WEI LP | 1 | 0.033 | CIMATTI L | 1 | 0.033 |
| WEI LL | 1 | 0.033 | CILLO C | 1 | 0.033 |
| WEI L | 1 | 0.033 | CIGLIANO RA | 1 | 0.033 |
| WEI KL | 1 | 0.033 | CICKA D | 1 | 0.033 |
| WEI JW | 1 | 0.033 | CICHOCKI F | 1 | 0.033 |
| WEI JT | 1 | 0.033 | CICHEWICZ MA | 1 | 0.033 |
| WEI JS | 1 | 0.033 | CICCHINI C | 1 | 0.033 |
| WEI JJ | 1 | 0.033 | CI XP | 1 | 0.033 |
| WEI J | 1 | 0.033 | CI D | 1 | 0.033 |
| WEI HY | 1 | 0.033 | CHURKO J | 1 | 0.033 |
| WEI HW | 1 | 0.033 | CHUONG EB | 1 | 0.033 |
| WEI HT | 1 | 0.033 | CHUNHAROJRITH P | 1 | 0.033 |
| WEI HM | 1 | 0.033 | CHUNG WY | 1 | 0.033 |
| WEI HJ | 1 | 0.033 | CHUNG SY | 1 | 0.033 |
| WEI H | 1 | 0.033 | CHUNG RT | 1 | 0.033 |
| WEI GZ | 1 | 0.033 | CHUNG H | 1 | 0.033 |
| WEI GB | 1 | 0.033 | CHUNG ACK | 1 | 0.033 |
| WEI DJ | 1 | 0.033 | CHUN S | 1 | 0.033 |
| WEI CC | 1 | 0.033 | CHUN KH | 1 | 0.033 |
| WEI BJ | 1 | 0.033 | CHUN CH | 1 | 0.033 |
| WEHLING MD | 1 | 0.033 | CHUJO T | 1 | 0.033 |
| WEEKS KM | 1 | 0.033 | CHUGANI HT | 1 | 0.033 |
| WEBER M | 1 | 0.033 | CHU WM | 1 | 0.033 |
| WEBER FA | 1 | 0.033 | CHU TT | 1 | 0.033 |
| WEBBER C | 1 | 0.033 | CHU SS | 1 | 0.033 |
| WEBB CH | 1 | 0.033 | CHU S | 1 | 0.033 |
| WEAVER DL | 1 | 0.033 | CHU QH | 1 | 0.033 |
| WAYE MMY | 1 | 0.033 | CHU PP | 1 | 0.033 |
| WATTS R | 1 | 0.033 | CHU MP | 1 | 0.033 |
| WATT S | 1 | 0.033 | CHU LW | 1 | 0.033 |
| WATT A | 1 | 0.033 | CHU HP | 1 | 0.033 |
| WATSON JE | 1 | 0.033 | CHU HD | 1 | 0.033 |
| WATERS M | 1 | 0.033 | CHU H | 1 | 0.033 |
| WATERS EV | 1 | 0.033 | CHU FH | 1 | 0.033 |
| WATERHOUSE RM | 1 | 0.033 | CHU ETJ | 1 | 0.033 |
| WATANABE T | 1 | 0.033 | CHU BF | 1 | 0.033 |
| WATAHIKI A | 1 | 0.033 | CHRISTY TW | 1 | 0.033 |
| WASHINGTON MK | 1 | 0.033 | CHRISTIANI DC | 1 | 0.033 |
| WASHIETL S | 1 | 0.033 | CHRAST J | 1 | 0.033 |
| WARSCHEID B | 1 | 0.033 | CHOWDHURY T | 1 | 0.033 |
| WARREN RL | 1 | 0.033 | CHOW A | 1 | 0.033 |
| WARNEFORS M | 1 | 0.033 | CHOPRA N | 1 | 0.033 |
| WARE CB | 1 | 0.033 | CHOPRA M | 1 | 0.033 |
| WARD A | 1 | 0.033 | CHOPIN LK | 1 | 0.033 |
| WARCZOK KE | 1 | 0.033 | CHONG C | 1 | 0.033 |
| WARANECKI P | 1 | 0.033 | CHOI Y | 1 | 0.033 |
| WAPINSKI OL | 1 | 0.033 | CHOI SR | 1 | 0.033 |
| WAPINSKI O | 1 | 0.033 | CHOI MY | 1 | 0.033 |
| WANG-YUE W | 1 | 0.033 | CHOI I | 1 | 0.033 |
| WANG-RODRIGUEZ J | 1 | 0.033 | CHO SJ | 1 | 0.033 |
| WANG ZR | 1 | 0.033 | CHO SF | 1 | 0.033 |
| WANG ZD | 1 | 0.033 | CHO NH | 1 | 0.033 |
| WANG ZB | 1 | 0.033 | CHO JH | 1 | 0.033 |
| WANG YR | 1 | 0.033 | CHIYOMARU T | 1 | 0.033 |
| WANG YG | 1 | 0.033 | CHIVUKULA P | 1 | 0.033 |
| WANG XZ | 1 | 0.033 | CHIU DKY | 1 | 0.033 |
| WANG XT | 1 | 0.033 | CHISHOLM KM | 1 | 0.033 |
| WANG XR | 1 | 0.033 | CHIOCCA EA | 1 | 0.033 |
| WANG XA | 1 | 0.033 | CHINNAIYAN A | 1 | 0.033 |
| WANG WY | 1 | 0.033 | CHINEN Y | 1 | 0.033 |
| WANG WX | 1 | 0.033 | CHINAPPI M | 1 | 0.033 |
| WANG WN | 1 | 0.033 | CHILLON I | 1 | 0.033 |
| WANG WJ | 1 | 0.033 | CHIEN HC | 1 | 0.033 |
| WANG WB | 1 | 0.033 | CHIARI R | 1 | 0.033 |
| WANG WA | 1 | 0.033 | CHIANG C | 1 | 0.033 |
| WANG TY | 1 | 0.033 | CHI YY | 1 | 0.033 |
| WANG TJ | 1 | 0.033 | CHI YB | 1 | 0.033 |
| WANG SZ | 1 | 0.033 | CHI XJ | 1 | 0.033 |
| WANG ST | 1 | 0.033 | CHI X | 1 | 0.033 |
| WANG SSW | 1 | 0.033 | CHI KN | 1 | 0.033 |
| WANG SP | 1 | 0.033 | CHI JC | 1 | 0.033 |
| WANG SN | 1 | 0.033 | CHI HY | 1 | 0.033 |
| WANG SM | 1 | 0.033 | CHEW GL | 1 | 0.033 |
| WANG SD | 1 | 0.033 | CHEVILLARD C | 1 | 0.033 |
| WANG RZ | 1 | 0.033 | CHEUNG T | 1 | 0.033 |
| WANG RY | 1 | 0.033 | CHEUNG LH | 1 | 0.033 |
| WANG RR | 1 | 0.033 | CHEUNG E | 1 | 0.033 |
| WANG RQ | 1 | 0.033 | CHEUNG B | 1 | 0.033 |
| WANG RM | 1 | 0.033 | CHEUNG AFP | 1 | 0.033 |
| WANG RK | 1 | 0.033 | CHETTOOR AM | 1 | 0.033 |
| WANG RJ | 1 | 0.033 | CHERUKURI PF | 1 | 0.033 |
| WANG RF | 1 | 0.033 | CHERNYI AA | 1 | 0.033 |
| WANG QX | 1 | 0.033 | CHEONG JH | 1 | 0.033 |
| WANG QT | 1 | 0.033 | CHENG ZY | 1 | 0.033 |
| WANG QR | 1 | 0.033 | CHENG ZX | 1 | 0.033 |
| WANG QF | 1 | 0.033 | CHENG ZP | 1 | 0.033 |
| WANG PR | 1 | 0.033 | CHENG ZL | 1 | 0.033 |
| WANG PJ | 1 | 0.033 | CHENG ZH | 1 | 0.033 |
| WANG PI | 1 | 0.033 | CHENG ZA | 1 | 0.033 |
| WANG NS | 1 | 0.033 | CHENG YX | 1 | 0.033 |
| WANG MS | 1 | 0.033 | CHENG XY | 1 | 0.033 |
| WANG MQ | 1 | 0.033 | CHENG XF | 1 | 0.033 |
| WANG MN | 1 | 0.033 | CHENG XD | 1 | 0.033 |
| WANG MM | 1 | 0.033 | CHENG XC | 1 | 0.033 |
| WANG MJ | 1 | 0.033 | CHENG TC | 1 | 0.033 |
| WANG MD | 1 | 0.033 | CHENG SY | 1 | 0.033 |
| WANG LX | 1 | 0.033 | CHENG SQ | 1 | 0.033 |
| WANG LM | 1 | 0.033 | CHENG SL | 1 | 0.033 |
| WANG LF | 1 | 0.033 | CHENG SD | 1 | 0.033 |
| WANG KZ | 1 | 0.033 | CHENG LH | 1 | 0.033 |
| WANG KX | 1 | 0.033 | CHENG K | 1 | 0.033 |
| WANG KS | 1 | 0.033 | CHENG JZ | 1 | 0.033 |
| WANG KP | 1 | 0.033 | CHENG JX | 1 | 0.033 |
| WANG JZ | 1 | 0.033 | CHENG JT | 1 | 0.033 |
| WANG JW | 1 | 0.033 | CHENG JL | 1 | 0.033 |
| WANG JT | 1 | 0.033 | CHENG HY | 1 | 0.033 |
| WANG JK | 1 | 0.033 | CHENG HR | 1 | 0.033 |
| WANG JG | 1 | 0.033 | CHENG HP | 1 | 0.033 |
| WANG JC | 1 | 0.033 | CHENG HL | 1 | 0.033 |
| WANG JB | 1 | 0.033 | CHENG GQ | 1 | 0.033 |
| WANG IK | 1 | 0.033 | CHENG GC | 1 | 0.033 |
| WANG HS | 1 | 0.033 | CHENG DJ | 1 | 0.033 |
| WANG HR | 1 | 0.033 | CHENG DF | 1 | 0.033 |
| WANG HP | 1 | 0.033 | CHENG D | 1 | 0.033 |
| WANG HC | 1 | 0.033 | CHENG CJ | 1 | 0.033 |
| WANG GZ | 1 | 0.033 | CHENG C | 1 | 0.033 |
| WANG GS | 1 | 0.033 | CHENG B | 1 | 0.033 |
| WANG GH | 1 | 0.033 | CHENG A | 1 | 0.033 |
| WANG FQ | 1 | 0.033 | CHEN ZW | 1 | 0.033 |
| WANG FM | 1 | 0.033 | CHEN ZQ | 1 | 0.033 |
| WANG FH | 1 | 0.033 | CHEN ZP | 1 | 0.033 |
| WANG FG | 1 | 0.033 | CHEN ZJ | 1 | 0.033 |
| WANG DX | 1 | 0.033 | CHEN ZG | 1 | 0.033 |
| WANG DQ | 1 | 0.033 | CHEN YS | 1 | 0.033 |
| WANG DP | 1 | 0.033 | CHEN YPP | 1 | 0.033 |
| WANG DN | 1 | 0.033 | CHEN YN | 1 | 0.033 |
| WANG DJ | 1 | 0.033 | CHEN YE | 1 | 0.033 |
| WANG DG | 1 | 0.033 | CHEN XT | 1 | 0.033 |
| WANG DD | 1 | 0.033 | CHEN XS | 1 | 0.033 |
| WANG CS | 1 | 0.033 | CHEN XQ | 1 | 0.033 |
| WANG CL | 1 | 0.033 | CHEN XP | 1 | 0.033 |
| WANG CH | 1 | 0.033 | CHEN XG | 1 | 0.033 |
| WANG CG | 1 | 0.033 | CHEN WX | 1 | 0.033 |
| WANG CD | 1 | 0.033 | CHEN WW | 1 | 0.033 |
| WANG BZ | 1 | 0.033 | CHEN WT | 1 | 0.033 |
| WANG BY | 1 | 0.033 | CHEN WD | 1 | 0.033 |
| WANG BQ | 1 | 0.033 | CHEN TZ | 1 | 0.033 |
| WANG BL | 1 | 0.033 | CHEN TX | 1 | 0.033 |
| WANG BJ | 1 | 0.033 | CHEN TT | 1 | 0.033 |
| WANG BF | 1 | 0.033 | CHEN TB | 1 | 0.033 |
| WANG AY | 1 | 0.033 | CHEN SY | 1 | 0.033 |
| WAN ZY | 1 | 0.033 | CHEN SX | 1 | 0.033 |
| WAN YJY | 1 | 0.033 | CHEN SW | 1 | 0.033 |
| WAN XX | 1 | 0.033 | CHEN ST | 1 | 0.033 |
| WAN XS | 1 | 0.033 | CHEN SQ | 1 | 0.033 |
| WAN XP | 1 | 0.033 | CHEN SN | 1 | 0.033 |
| WAN XH | 1 | 0.033 | CHEN SJ | 1 | 0.033 |
| WAN XF | 1 | 0.033 | CHEN SF | 1 | 0.033 |
| WAN XD | 1 | 0.033 | CHEN RO | 1 | 0.033 |
| WAN XB | 1 | 0.033 | CHEN RD | 1 | 0.033 |
| WAN LD | 1 | 0.033 | CHEN QQ | 1 | 0.033 |
| WAN J | 1 | 0.033 | CHEN QP | 1 | 0.033 |
| WAN GQ | 1 | 0.033 | CHEN QF | 1 | 0.033 |
| WAN G | 1 | 0.033 | CHEN PX | 1 | 0.033 |
| WAN DCC | 1 | 0.033 | CHEN PS | 1 | 0.033 |
| WAN DC | 1 | 0.033 | CHEN NW | 1 | 0.033 |
| WAN BB | 1 | 0.033 | CHEN NQ | 1 | 0.033 |
| WAMBACH JA | 1 | 0.033 | CHEN MY | 1 | 0.033 |
| WALSH AL | 1 | 0.033 | CHEN MT | 1 | 0.033 |
| WALPOLE CM | 1 | 0.033 | CHEN MK | 1 | 0.033 |
| WAKAMATSU A | 1 | 0.033 | CHEN MJM | 1 | 0.033 |
| WAKABAYASHI G | 1 | 0.033 | CHEN MH | 1 | 0.033 |
| WAGNER W | 1 | 0.033 | CHEN LZ | 1 | 0.033 |
| WADA Y | 1 | 0.033 | CHEN LN | 1 | 0.033 |
| WACKER DA | 1 | 0.033 | CHEN LM | 1 | 0.033 |
| WAAGE J | 1 | 0.033 | CHEN LK | 1 | 0.033 |
| VYCITAL O | 1 | 0.033 | CHEN LJ | 1 | 0.033 |
| VUKCEVIC D | 1 | 0.033 | CHEN LG | 1 | 0.033 |
| VUCIC EA | 1 | 0.033 | CHEN KN | 1 | 0.033 |
| VU JP | 1 | 0.033 | CHEN KK | 1 | 0.033 |
| VRUGT B | 1 | 0.033 | CHEN JW | 1 | 0.033 |
| VRBA L | 1 | 0.033 | CHEN JS | 1 | 0.033 |
| VRATI S | 1 | 0.033 | CHEN JQ | 1 | 0.033 |
| VRANA KE | 1 | 0.033 | CHEN JD | 1 | 0.033 |
| VOSS R | 1 | 0.033 | CHEN I | 1 | 0.033 |
| VOSA U | 1 | 0.033 | CHEN HZ | 1 | 0.033 |
| VOORHEES JJ | 1 | 0.033 | CHEN HT | 1 | 0.033 |
| VONDRISKA TM | 1 | 0.033 | CHEN HSV | 1 | 0.033 |
| VOLPERT O | 1 | 0.033 | CHEN HJ | 1 | 0.033 |
| VOLMAR CH | 1 | 0.033 | CHEN HC | 1 | 0.033 |
| VOLLENWEIDER E | 1 | 0.033 | CHEN HB | 1 | 0.033 |
| VOLKMANN I | 1 | 0.033 | CHEN GW | 1 | 0.033 |
| VOLKMAN S | 1 | 0.033 | CHEN GS | 1 | 0.033 |
| VOLINIA S | 1 | 0.033 | CHEN GG | 1 | 0.033 |
| VOGT N | 1 | 0.033 | CHEN FY | 1 | 0.033 |
| VOGEL T | 1 | 0.033 | CHEN ES | 1 | 0.033 |
| VOEGELE C | 1 | 0.033 | CHEN DZ | 1 | 0.033 |
| VODICKOVA L | 1 | 0.033 | CHEN DY | 1 | 0.033 |
| VODICKA P | 1 | 0.033 | CHEN DQ | 1 | 0.033 |
| VLATKOVIC I | 1 | 0.033 | CHEN DF | 1 | 0.033 |
| VIZAN P | 1 | 0.033 | CHEN DC | 1 | 0.033 |
| VITIELLO M | 1 | 0.033 | CHEN DB | 1 | 0.033 |
| VITHANA EN | 1 | 0.033 | CHEN CK | 1 | 0.033 |
| VISWANATHAN AC | 1 | 0.033 | CHEN CC | 1 | 0.033 |
| VIRTUE AT | 1 | 0.033 | CHEN BW | 1 | 0.033 |
| VIRNICCHI G | 1 | 0.033 | CHEN BJ | 1 | 0.033 |
| VINOLAS N | 1 | 0.033 | CHEN BH | 1 | 0.033 |
| VINCI C | 1 | 0.033 | CHEN BE | 1 | 0.033 |
| VINCENT K | 1 | 0.033 | CHEN BD | 1 | 0.033 |
| VILLOTA C | 1 | 0.033 | CHEN BC | 1 | 0.033 |
| VILLENEUVE PJ | 1 | 0.033 | CHEN BB | 1 | 0.033 |
| VILLEGAS-SEPULVEDA N | 1 | 0.033 | CHEKANOVA JA | 1 | 0.033 |
| VILLEGAS VE | 1 | 0.033 | CHEETHAM SW | 1 | 0.033 |
| VILLEGAS J | 1 | 0.033 | CHE Q | 1 | 0.033 |
| VILLANUEVA A | 1 | 0.033 | CHE L | 1 | 0.033 |
| VILLACAMPA F | 1 | 0.033 | CHATTERJEE S | 1 | 0.033 |
| VILIEN M | 1 | 0.033 | CHATTERJEE D | 1 | 0.033 |
| VILELLA-ARIAS SA | 1 | 0.033 | CHARPENTIER M | 1 | 0.033 |
| VIKRAM R | 1 | 0.033 | CHARNECKI T | 1 | 0.033 |
| VIGNERON N | 1 | 0.033 | CHARLEY PA | 1 | 0.033 |
| VIGNEAULT C | 1 | 0.033 | CHARI R | 1 | 0.033 |
| VIGNAL GM | 1 | 0.033 | CHAR K | 1 | 0.033 |
| VIESTER MD | 1 | 0.033 | CHAPMAN SJ | 1 | 0.033 |
| VIERECK J | 1 | 0.033 | CHAPMAN JAW | 1 | 0.033 |
| VIDAUD M | 1 | 0.033 | CHAO TC | 1 | 0.033 |
| VIDARSDOTTIR L | 1 | 0.033 | CHAO J | 1 | 0.033 |
| VIDAN N | 1 | 0.033 | CHAO F | 1 | 0.033 |
| VIDAL DO | 1 | 0.033 | CHANGE HY | 1 | 0.033 |
| VIDAL A | 1 | 0.033 | CHANG ZQ | 1 | 0.033 |
| VESEY AT | 1 | 0.033 | CHANG YZ | 1 | 0.033 |
| VERNOCHET C | 1 | 0.033 | CHANG YY | 1 | 0.033 |
| VERMI W | 1 | 0.033 | CHANG YT | 1 | 0.033 |
| VERMA AK | 1 | 0.033 | CHANG YS | 1 | 0.033 |
| VERHOEVEN RJA | 1 | 0.033 | CHANG YC | 1 | 0.033 |
| VERHOEF EI | 1 | 0.033 | CHANG XL | 1 | 0.033 |
| VERHEGGEN K | 1 | 0.033 | CHANG WJ | 1 | 0.033 |
| VERHAAK RGW | 1 | 0.033 | CHANG WH | 1 | 0.033 |
| VERGULT S | 1 | 0.033 | CHANG SS | 1 | 0.033 |
| VERFAILLIE A | 1 | 0.033 | CHANG S | 1 | 0.033 |
| VERDEL A | 1 | 0.033 | CHANG QS | 1 | 0.033 |
| VENTURIN M | 1 | 0.033 | CHANG PC | 1 | 0.033 |
| VENTURA A | 1 | 0.033 | CHANG KC | 1 | 0.033 |
| VENDRAMIN R | 1 | 0.033 | CHANG JH | 1 | 0.033 |
| VENALAINEN E | 1 | 0.033 | CHANG I | 1 | 0.033 |
| VELLARIKKAL SK | 1 | 0.033 | CHANG HW | 1 | 0.033 |
| VELA-BOZA A | 1 | 0.033 | CHANG HD | 1 | 0.033 |
| VELA LE | 1 | 0.033 | CHANG H | 1 | 0.033 |
| VEERAVALLI L | 1 | 0.033 | CHANG CL | 1 | 0.033 |
| VASTENHOUW NL | 1 | 0.033 | CHANG CJ | 1 | 0.033 |
| VASSALLO I | 1 | 0.033 | CHANG CF | 1 | 0.033 |
| VASQUEZ KM | 1 | 0.033 | CHANG CC | 1 | 0.033 |
| VASCONEZ AE | 1 | 0.033 | CHANG BH | 1 | 0.033 |
| VARMA S | 1 | 0.033 | CHANDLER B | 1 | 0.033 |
| VARGAS M | 1 | 0.033 | CHAN J | 1 | 0.033 |
| VARDY LA | 1 | 0.033 | CHAN GCF | 1 | 0.033 |
| VANNINI I | 1 | 0.033 | CHAMBERLAIN A | 1 | 0.033 |
| VANICHKINA DP | 1 | 0.033 | CHALLA S | 1 | 0.033 |
| VANHAUWAERT S | 1 | 0.033 | CHALLA P | 1 | 0.033 |
| VANDERHEYDEN K | 1 | 0.033 | CHAKRAVARTY D | 1 | 0.033 |
| VANDEPOELE K | 1 | 0.033 | CHAKRAVARTI D | 1 | 0.033 |
| VANDEKERCKHOVE L | 1 | 0.033 | CHAKRABORTY S | 1 | 0.033 |
| VAN ZN | 1 | 0.033 | CHAKRABORTY D | 1 | 0.033 |
| VAN ZANDWIJK N | 1 | 0.033 | CHAKRABORTY A | 1 | 0.033 |
| VAN WERVEN FJ | 1 | 0.033 | CHAKRABARTI S | 1 | 0.033 |
| VAN TYNE D | 1 | 0.033 | CHAIWORAPONGSA T | 1 | 0.033 |
| VAN OORT RJ | 1 | 0.033 | CHAI YQ | 1 | 0.033 |
| VAN NIEUWERBURGH F | 1 | 0.033 | CHAI YH | 1 | 0.033 |
| VAN NIEKERK D | 1 | 0.033 | CHAI XF | 1 | 0.033 |
| VAN LEEUWEN S | 1 | 0.033 | CHAI LL | 1 | 0.033 |
| VAN LEENDERS GJLH | 1 | 0.033 | CHAI JK | 1 | 0.033 |
| VAN L | 1 | 0.033 | CHAI HY | 1 | 0.033 |
| VAN KEYMEULEN A | 1 | 0.033 | CHAI CY | 1 | 0.033 |
| VAN ITERSON M | 1 | 0.033 | CHAEMSAITHONG P | 1 | 0.033 |
| VAN HEESCH S | 1 | 0.033 | CHAE YS | 1 | 0.033 |
| VAN HECKE C | 1 | 0.033 | CHAE YC | 1 | 0.033 |
| VAN DIJK M | 1 | 0.033 | CHADWICK BP | 1 | 0.033 |
| VAN DER MEULEN T | 1 | 0.033 | CHADALAPAKA G | 1 | 0.033 |
| VAN DER MEER RW | 1 | 0.033 | CHACKO N | 1 | 0.033 |
| VAN DEN EYNDEN J | 1 | 0.033 | CETINKAYA M | 1 | 0.033 |
| VAN DE WALLE I | 1 | 0.033 | CETIN G | 1 | 0.033 |
| VAN DE VONDERVOORT IIGM | 1 | 0.033 | CESARONI M | 1 | 0.033 |
| VAN DE VIJVER MJ | 1 | 0.033 | CESANA M | 1 | 0.033 |
| VAN DE RIJN M | 1 | 0.033 | CERK S | 1 | 0.033 |
| VAN AELST S | 1 | 0.033 | CENGIZ M | 1 | 0.033 |
| VALLOT C | 1 | 0.033 | CEN Y | 1 | 0.033 |
| VALLI E | 1 | 0.033 | CEN XB | 1 | 0.033 |
| VALLEDOR L | 1 | 0.033 | CEN SZ | 1 | 0.033 |
| VALERIO F | 1 | 0.033 | CEN BH | 1 | 0.033 |
| VALERI N | 1 | 0.033 | CEN B | 1 | 0.033 |
| VALENTE V | 1 | 0.033 | CECH TR | 1 | 0.033 |
| VALENCIA-HIPOLITO A | 1 | 0.033 | CECCHI D | 1 | 0.033 |
| VALDES-QUEZADA C | 1 | 0.033 | CAZZELLA V | 1 | 0.033 |
| VALAPERTA R | 1 | 0.033 | CAVENEE WK | 1 | 0.033 |
| VAKA D | 1 | 0.033 | CAVANAUGH C | 1 | 0.033 |
| VAID R | 1 | 0.033 | CAUGHEY J | 1 | 0.033 |
| VAGO R | 1 | 0.033 | CATTO JWF | 1 | 0.033 |
| UYTTEBROECK A | 1 | 0.033 | CATHALA G | 1 | 0.033 |
| UYOGA S | 1 | 0.033 | CASTORI-EPPENBERGER S | 1 | 0.033 |
| UYAR A | 1 | 0.033 | CASTLE JC | 1 | 0.033 |
| UVA P | 1 | 0.033 | CASTELVECCHIO S | 1 | 0.033 |
| USZCZYNSKA-RATAJCZAK B | 1 | 0.033 | CASTELLANOS-RUBIO A | 1 | 0.033 |
| USHIJIMA M | 1 | 0.033 | CASTELLANO M | 1 | 0.033 |
| URSVIK A | 1 | 0.033 | CASTELLANO JJ | 1 | 0.033 |
| URIBE-LEWIS S | 1 | 0.033 | CASTELLANO I | 1 | 0.033 |
| URANITSCH S | 1 | 0.033 | CASTELLANO D | 1 | 0.033 |
| UNIACKE J | 1 | 0.033 | CASS AA | 1 | 0.033 |
| UNGEWICKELL A | 1 | 0.033 | CASS A | 1 | 0.033 |
| UNFRIED JP | 1 | 0.033 | CASAS JP | 1 | 0.033 |
| UMLAUF D | 1 | 0.033 | CASA V | 1 | 0.033 |
| UMA S | 1 | 0.033 | CARVALHO S | 1 | 0.033 |
| ULLOA CSD | 1 | 0.033 | CARTER TH | 1 | 0.033 |
| ULLAH U | 1 | 0.033 | CARTER SL | 1 | 0.033 |
| UJINO S | 1 | 0.033 | CARTER G | 1 | 0.033 |
| UHL M | 1 | 0.033 | CARTER DRF | 1 | 0.033 |
| UESAKA M | 1 | 0.033 | CARROLL AJ | 1 | 0.033 |
| UEMURA M | 1 | 0.033 | CARRIERO R | 1 | 0.033 |
| UEBE S | 1 | 0.033 | CARRIERI C | 1 | 0.033 |
| UDAY S | 1 | 0.033 | CARRASCOSA LG | 1 | 0.033 |
| UCKELMANN H | 1 | 0.033 | CARRARO DM | 1 | 0.033 |
| UCHIDA C | 1 | 0.033 | CARMICHAEL TR | 1 | 0.033 |
| UBIETA LD | 1 | 0.033 | CARLSON HL | 1 | 0.033 |
| TZIKA A | 1 | 0.033 | CARLSON EE | 1 | 0.033 |
| TYE CE | 1 | 0.033 | CARLEY A | 1 | 0.033 |
| TYBULEWICZ VLJ | 1 | 0.033 | CARLEVARO-FITA J | 1 | 0.033 |
| TUZOVA AV | 1 | 0.033 | CAREY BW | 1 | 0.033 |
| TURNER AMW | 1 | 0.033 | CARDOSO C | 1 | 0.033 |
| TURK K | 1 | 0.033 | CARDO-VILA M | 1 | 0.033 |
| TURECKI G | 1 | 0.033 | CARBONNELLE D | 1 | 0.033 |
| TUO YL | 1 | 0.033 | CARBONELL F | 1 | 0.033 |
| TUNG YA | 1 | 0.033 | CAPORASO N | 1 | 0.033 |
| TUNG CS | 1 | 0.033 | CAPOGROSSI MC | 1 | 0.033 |
| TUNG CH | 1 | 0.033 | CAPACCIOLI S | 1 | 0.033 |
| TUDORAN O | 1 | 0.033 | CAO ZZ | 1 | 0.033 |
| TUCKER AF | 1 | 0.033 | CAO ZY | 1 | 0.033 |
| TUCK AC | 1 | 0.033 | CAO Z | 1 | 0.033 |
| TUCCOLI A | 1 | 0.033 | CAO YZ | 1 | 0.033 |
| TU ZQ | 1 | 0.033 | CAO YS | 1 | 0.033 |
| TU ZG | 1 | 0.033 | CAO YM | 1 | 0.033 |
| TU ZB | 1 | 0.033 | CAO YL | 1 | 0.033 |
| TU YF | 1 | 0.033 | CAO YJ | 1 | 0.033 |
| TU WJ | 1 | 0.033 | CAO YG | 1 | 0.033 |
| TU LL | 1 | 0.033 | CAO XM | 1 | 0.033 |
| TU L | 1 | 0.033 | CAO XG | 1 | 0.033 |
| TU KS | 1 | 0.033 | CAO WJ | 1 | 0.033 |
| TU JJ | 1 | 0.033 | CAO TZ | 1 | 0.033 |
| TU JB | 1 | 0.033 | CAO SZ | 1 | 0.033 |
| TU HL | 1 | 0.033 | CAO SW | 1 | 0.033 |
| TU GH | 1 | 0.033 | CAO SQ | 1 | 0.033 |
| TSUYADA A | 1 | 0.033 | CAO SJ | 1 | 0.033 |
| TSUKAMOTO T | 1 | 0.033 | CAO SH | 1 | 0.033 |
| TSUDZUKI T | 1 | 0.033 | CAO SC | 1 | 0.033 |
| TSUCHIYA H | 1 | 0.033 | CAO S | 1 | 0.033 |
| TSOI LC | 1 | 0.033 | CAO RM | 1 | 0.033 |
| TSIVIN V | 1 | 0.033 | CAO RB | 1 | 0.033 |
| TSITSIOU E | 1 | 0.033 | CAO QY | 1 | 0.033 |
| TSIRIGOS A | 1 | 0.033 | CAO QL | 1 | 0.033 |
| TSIKRIKA P | 1 | 0.033 | CAO QF | 1 | 0.033 |
| TSENG YW | 1 | 0.033 | CAO NN | 1 | 0.033 |
| TSE HF | 1 | 0.033 | CAO MR | 1 | 0.033 |
| TSANG P | 1 | 0.033 | CAO LY | 1 | 0.033 |
| TSANG FHC | 1 | 0.033 | CAO LX | 1 | 0.033 |
| TSANG ACO | 1 | 0.033 | CAO LM | 1 | 0.033 |
| TSAGRIS M | 1 | 0.033 | CAO LJ | 1 | 0.033 |
| TRYPSTEEN W | 1 | 0.033 | CAO KJ | 1 | 0.033 |
| TRUMPER L | 1 | 0.033 | CAO K | 1 | 0.033 |
| TRUJILLANO D | 1 | 0.033 | CAO JP | 1 | 0.033 |
| TROUPES CD | 1 | 0.033 | CAO JN | 1 | 0.033 |
| TROUP M | 1 | 0.033 | CAO JJ | 1 | 0.033 |
| TRONCOSO P | 1 | 0.033 | CAO HY | 1 | 0.033 |
| TRIPODI M | 1 | 0.033 | CAO HX | 1 | 0.033 |
| TRIPATHI S | 1 | 0.033 | CAO HQ | 1 | 0.033 |
| TRIPATHI P | 1 | 0.033 | CAO HJ | 1 | 0.033 |
| TRINKLE-MULCAHY L | 1 | 0.033 | CAO GW | 1 | 0.033 |
| TRIMARCHI T | 1 | 0.033 | CAO GQ | 1 | 0.033 |
| TRICHE TJ | 1 | 0.033 | CAO FL | 1 | 0.033 |
| TREMBLAY KD | 1 | 0.033 | CAO F | 1 | 0.033 |
| TREMBERGER G | 1 | 0.033 | CAO DY | 1 | 0.033 |
| TREMBATH RC | 1 | 0.033 | CAO D | 1 | 0.033 |
| TRAYES-GIBSON Z | 1 | 0.033 | CAO CY | 1 | 0.033 |
| TRAU M | 1 | 0.033 | CAO CH | 1 | 0.033 |
| TRAN UM | 1 | 0.033 | CAO BR | 1 | 0.033 |
| TRAN TA | 1 | 0.033 | CAO BP | 1 | 0.033 |
| TRAN NT | 1 | 0.033 | CANNON SC | 1 | 0.033 |
| TRAN N | 1 | 0.033 | CANMAN CE | 1 | 0.033 |
| TRAN C | 1 | 0.033 | CANGELOSI A | 1 | 0.033 |
| TRAKHTENBROT L | 1 | 0.033 | CANDUSSIO L | 1 | 0.033 |
| TRAC C | 1 | 0.033 | CANDIDO DD | 1 | 0.033 |
| TOWNES TM | 1 | 0.033 | CAMPOS FS | 1 | 0.033 |
| TOWN T | 1 | 0.033 | CAMPISTOL JM | 1 | 0.033 |
| TOWLER D | 1 | 0.033 | CAMPBELL C | 1 | 0.033 |
| TOWE CT | 1 | 0.033 | CAMP NJ | 1 | 0.033 |
| TOUMA M | 1 | 0.033 | CAMINCI P | 1 | 0.033 |
| TOU E | 1 | 0.033 | CAMERON-SMITH D | 1 | 0.033 |
| TOSSBERG JT | 1 | 0.033 | CAMERO E | 1 | 0.033 |
| TORTORELLI AF | 1 | 0.033 | CAMARGO L | 1 | 0.033 |
| TORRY DS | 1 | 0.033 | CALIN G | 1 | 0.033 |
| TORRING PM | 1 | 0.033 | CALIGIURIA MA | 1 | 0.033 |
| TORREY L | 1 | 0.033 | CALEY DP | 1 | 0.033 |
| TORO R | 1 | 0.033 | CALDAS C | 1 | 0.033 |
| TORNILLO L | 1 | 0.033 | CALCINO AD | 1 | 0.033 |
| TORMO M | 1 | 0.033 | CALABRESE JM | 1 | 0.033 |
| TONG Y | 1 | 0.033 | CAKMAK HA | 1 | 0.033 |
| TONG XW | 1 | 0.033 | CAIZZI L | 1 | 0.033 |
| TONG WM | 1 | 0.033 | CAIRNS MJ | 1 | 0.033 |
| TONG W | 1 | 0.033 | CAILLE D | 1 | 0.033 |
| TONG SL | 1 | 0.033 | CAI ZW | 1 | 0.033 |
| TONG S | 1 | 0.033 | CAI ZL | 1 | 0.033 |
| TONG Q | 1 | 0.033 | CAI ZD | 1 | 0.033 |
| TONG P | 1 | 0.033 | CAI YR | 1 | 0.033 |
| TONG ML | 1 | 0.033 | CAI YP | 1 | 0.033 |
| TONG L | 1 | 0.033 | CAI YL | 1 | 0.033 |
| TONG J | 1 | 0.033 | CAI XS | 1 | 0.033 |
| TONG CX | 1 | 0.033 | CAI XP | 1 | 0.033 |
| TOMSIC J | 1 | 0.033 | CAI WP | 1 | 0.033 |
| TOMMASINI A | 1 | 0.033 | CAI WB | 1 | 0.033 |
| TOMINAGA-YAMANAKA K | 1 | 0.033 | CAI T | 1 | 0.033 |
| TOMASSY GS | 1 | 0.033 | CAI SR | 1 | 0.033 |
| TOLSTORUKOV MY | 1 | 0.033 | CAI R | 1 | 0.033 |
| TOLLERVEY D | 1 | 0.033 | CAI QM | 1 | 0.033 |
| TOKUZUMI A | 1 | 0.033 | CAI LY | 1 | 0.033 |
| TOIYAMA Y | 1 | 0.033 | CAI K | 1 | 0.033 |
| TOES REM | 1 | 0.033 | CAI JY | 1 | 0.033 |
| TODEN S | 1 | 0.033 | CAI JQ | 1 | 0.033 |
| TOCHIGI N | 1 | 0.033 | CAI JP | 1 | 0.033 |
| TIWARI V | 1 | 0.033 | CAI JJ | 1 | 0.033 |
| TISONCIK-GO J | 1 | 0.033 | CAI HR | 1 | 0.033 |
| TIMMERMANS MCP | 1 | 0.033 | CAI HH | 1 | 0.033 |
| TILGNER H | 1 | 0.033 | CAI HB | 1 | 0.033 |
| TIGYI K | 1 | 0.033 | CAI GS | 1 | 0.033 |
| TIESINGA PHE | 1 | 0.033 | CAFFARELLI E | 1 | 0.033 |
| TICHON A | 1 | 0.033 | CADIERGUES MC | 1 | 0.033 |
| TIBSHIRANI R | 1 | 0.033 | CACERES E | 1 | 0.033 |
| TIBERIO GJ | 1 | 0.033 | CACCHIARELLI D | 1 | 0.033 |
| TIBBOEL D | 1 | 0.033 | CABRERA JR | 1 | 0.033 |
| TIANA M | 1 | 0.033 | CABIANCA DS | 1 | 0.033 |
| TIAN ZZ | 1 | 0.033 | CABANTOUS S | 1 | 0.033 |
| TIAN ZJ | 1 | 0.033 | CABANSKI CR | 1 | 0.033 |
| TIAN ZG | 1 | 0.033 | CABALLERO J | 1 | 0.033 |
| TIAN ZF | 1 | 0.033 | BYUN JW | 1 | 0.033 |
| TIAN Z | 1 | 0.033 | BYRON M | 1 | 0.033 |
| TIAN XD | 1 | 0.033 | BYRNE FL | 1 | 0.033 |
| TIAN WG | 1 | 0.033 | BYRD JC | 1 | 0.033 |
| TIAN WD | 1 | 0.033 | BUTTY VL | 1 | 0.033 |
| TIAN SK | 1 | 0.033 | BUTCHART LC | 1 | 0.033 |
| TIAN RR | 1 | 0.033 | BUSKE FA | 1 | 0.033 |
| TIAN QZ | 1 | 0.033 | BURZIO V | 1 | 0.033 |
| TIAN Q | 1 | 0.033 | BURZIO LO | 1 | 0.033 |
| TIAN ML | 1 | 0.033 | BURNS JC | 1 | 0.033 |
| TIAN M | 1 | 0.033 | BURNETT JC | 1 | 0.033 |
| TIAN L | 1 | 0.033 | BURKHARDT B | 1 | 0.033 |
| TIAN K | 1 | 0.033 | BURGHARD C | 1 | 0.033 |
| TIAN JW | 1 | 0.033 | BURGER MC | 1 | 0.033 |
| TIAN JL | 1 | 0.033 | BURGE CB | 1 | 0.033 |
| TIAN HH | 1 | 0.033 | BUREAU JF | 1 | 0.033 |
| TIAN G | 1 | 0.033 | BURDACH J | 1 | 0.033 |
| TIAN FM | 1 | 0.033 | BUQUICCHIO F | 1 | 0.033 |
| TIAN DL | 1 | 0.033 | BUNDSCHUH R | 1 | 0.033 |
| TIAN D | 1 | 0.033 | BUMPSTEAD SJ | 1 | 0.033 |
| TIAN CJ | 1 | 0.033 | BULLOCK MD | 1 | 0.033 |
| TIAN B | 1 | 0.033 | BULLOCK M | 1 | 0.033 |
| TIAG LL | 1 | 0.033 | BUKOWSKI R | 1 | 0.033 |
| TI YF | 1 | 0.033 | BUJANDA L | 1 | 0.033 |
| THURN T | 1 | 0.033 | BUITELAAR JK | 1 | 0.033 |
| THUML T | 1 | 0.033 | BUIGA R | 1 | 0.033 |
| THULLURU HK | 1 | 0.033 | BUI MM | 1 | 0.033 |
| THRUE CA | 1 | 0.033 | BUHIMSCHI IA | 1 | 0.033 |
| THORNBURG CK | 1 | 0.033 | BUHIMSCHI CS | 1 | 0.033 |
| THORENOOR N | 1 | 0.033 | BUENO RDLE | 1 | 0.033 |
| THOMSON JM | 1 | 0.033 | BUENO RDEL | 1 | 0.033 |
| THOMSON DW | 1 | 0.033 | BUCHHOLZ F | 1 | 0.033 |
| THOMPSONG EJ | 1 | 0.033 | BUCHANAN A | 1 | 0.033 |
| THOMPSON JF | 1 | 0.033 | BUCCI G | 1 | 0.033 |
| THOMPSON J | 1 | 0.033 | BUCCI D | 1 | 0.033 |
| THOMPSON EJ | 1 | 0.033 | BUCCHERI S | 1 | 0.033 |
| THOMAS PB | 1 | 0.033 | BU YL | 1 | 0.033 |
| THOMAS ML | 1 | 0.033 | BU XF | 1 | 0.033 |
| THOMAS M | 1 | 0.033 | BU SZ | 1 | 0.033 |
| THOMAS GP | 1 | 0.033 | BU RF | 1 | 0.033 |
| THOMAS D | 1 | 0.033 | BU Q | 1 | 0.033 |
| THOMAS B | 1 | 0.033 | BU PC | 1 | 0.033 |
| THOENISSEN NH | 1 | 0.033 | BRZOSKA P | 1 | 0.033 |
| THERMANN R | 1 | 0.033 | BRYZGHALOV O | 1 | 0.033 |
| THEPSUWAN P | 1 | 0.033 | BRUZZESE D | 1 | 0.033 |
| THEIS M | 1 | 0.033 | BRUSGAARD K | 1 | 0.033 |
| THEIS FJ | 1 | 0.033 | BRUNSCHWEIGER A | 1 | 0.033 |
| THE E | 1 | 0.033 | BRUNS P | 1 | 0.033 |
| THANNICKAL VJ | 1 | 0.033 | BRUNING JC | 1 | 0.033 |
| THANGAPANDI VR | 1 | 0.033 | BRUNET-VEGA A | 1 | 0.033 |
| THALAPPILLY S | 1 | 0.033 | BRUNET S | 1 | 0.033 |
| THAKUR C | 1 | 0.033 | BRUNCK ME | 1 | 0.033 |
| THAI P | 1 | 0.033 | BRUMMER A | 1 | 0.033 |
| TEWARI AK | 1 | 0.033 | BRUHN L | 1 | 0.033 |
| TESCHENDORFF AE | 1 | 0.033 | BRUCE SJ | 1 | 0.033 |
| TERRY T | 1 | 0.033 | BROZ DK | 1 | 0.033 |
| TERRERI S | 1 | 0.033 | BROWN SD | 1 | 0.033 |
| TERRADAS M | 1 | 0.033 | BROWN JWS | 1 | 0.033 |
| TERRACCIANO LM | 1 | 0.033 | BROWN JA | 1 | 0.033 |
| TERRACCIANO D | 1 | 0.033 | BROWN A | 1 | 0.033 |
| TERAI G | 1 | 0.033 | BROUILLET E | 1 | 0.033 |
| TENG ZY | 1 | 0.033 | BROTIN E | 1 | 0.033 |
| TENG LS | 1 | 0.033 | BROSH RM | 1 | 0.033 |
| TENG G | 1 | 0.033 | BROSENS E | 1 | 0.033 |
| TENG F | 1 | 0.033 | BRORSSON CA | 1 | 0.033 |
| TEJASVI T | 1 | 0.033 | BRONISZ A | 1 | 0.033 |
| TEIXEIRA PC | 1 | 0.033 | BROGI E | 1 | 0.033 |
| TEER JK | 1 | 0.033 | BROCQUEVILLE G | 1 | 0.033 |
| TCHOU J | 1 | 0.033 | BROCKS D | 1 | 0.033 |
| TCHITCHEK N | 1 | 0.033 | BROCKDORFF N | 1 | 0.033 |
| TAYLOR J | 1 | 0.033 | BROCK P | 1 | 0.033 |
| TAYLOR DH | 1 | 0.033 | BROADDUS R | 1 | 0.033 |
| TAYARI MM | 1 | 0.033 | BROADBENT JC | 1 | 0.033 |
| TAYARI M | 1 | 0.033 | BRITTAN M | 1 | 0.033 |
| TATEKAWA S | 1 | 0.033 | BRITTAIN E | 1 | 0.033 |
| TATE PH | 1 | 0.033 | BRISTOW RG | 1 | 0.033 |
| TASSONE P | 1 | 0.033 | BRIOSCHI S | 1 | 0.033 |
| TARAZONA S | 1 | 0.033 | BRIATA P | 1 | 0.033 |
| TAPANARI E | 1 | 0.033 | BRHANE Y | 1 | 0.033 |
| TAO ZH | 1 | 0.033 | BREUHAHN K | 1 | 0.033 |
| TAO YX | 1 | 0.033 | BREUER G | 1 | 0.033 |
| TAO YF | 1 | 0.033 | BRENTANI RR | 1 | 0.033 |
| TAO XG | 1 | 0.033 | BRENNER JC | 1 | 0.033 |
| TAO SY | 1 | 0.033 | BRENNAN S | 1 | 0.033 |
| TAO RY | 1 | 0.033 | BRENNAN P | 1 | 0.033 |
| TAO LC | 1 | 0.033 | BRENNAN KJ | 1 | 0.033 |
| TAO L | 1 | 0.033 | BRENKMAN AB | 1 | 0.033 |
| TAO J | 1 | 0.033 | BREGY A | 1 | 0.033 |
| TAO HB | 1 | 0.033 | BREESE MR | 1 | 0.033 |
| TAO GZ | 1 | 0.033 | BRECKWOLDT K | 1 | 0.033 |
| TAO GX | 1 | 0.033 | BRAZAO TF | 1 | 0.033 |
| TAO FB | 1 | 0.033 | BRAUN JE | 1 | 0.033 |
| TAO F | 1 | 0.033 | BRAUN FK | 1 | 0.033 |
| TAO D | 1 | 0.033 | BRATE J | 1 | 0.033 |
| TANURDZIC M | 1 | 0.033 | BRANDES RP | 1 | 0.033 |
| TANTAI JC | 1 | 0.033 | BRANCO MR | 1 | 0.033 |
| TANO K | 1 | 0.033 | BRAMON E | 1 | 0.033 |
| TANIWAKI M | 1 | 0.033 | BRAMHAM CR | 1 | 0.033 |
| TANIUE K | 1 | 0.033 | BRAISSANT O | 1 | 0.033 |
| TANG ZX | 1 | 0.033 | BRAHIC M | 1 | 0.033 |
| TANG ZQ | 1 | 0.033 | BRADNER JE | 1 | 0.033 |
| TANG ZP | 1 | 0.033 | BRADLEY RK | 1 | 0.033 |
| TANG ZL | 1 | 0.033 | BRADFORD JR | 1 | 0.033 |
| TANG ZH | 1 | 0.033 | BRADEL-TRETHEWAY BG | 1 | 0.033 |
| TANG YP | 1 | 0.033 | BRADDOCK M | 1 | 0.033 |
| TANG YJ | 1 | 0.033 | BRACONI C | 1 | 0.033 |
| TANG XS | 1 | 0.033 | BOYNE JR | 1 | 0.033 |
| TANG XR | 1 | 0.033 | BOYMANS S | 1 | 0.033 |
| TANG XF | 1 | 0.033 | BOXER LD | 1 | 0.033 |
| TANG WX | 1 | 0.033 | BOVOLENTA M | 1 | 0.033 |
| TANG WF | 1 | 0.033 | BOUZIT L | 1 | 0.033 |
| TANG WB | 1 | 0.033 | BOUZEK HK | 1 | 0.033 |
| TANG SY | 1 | 0.033 | BOUTROS PC | 1 | 0.033 |
| TANG SF | 1 | 0.033 | BOUSSAHA M | 1 | 0.033 |
| TANG S | 1 | 0.033 | BOURETTE RP | 1 | 0.033 |
| TANG RQ | 1 | 0.033 | BOUMART I | 1 | 0.033 |
| TANG QS | 1 | 0.033 | BOULBERDAA M | 1 | 0.033 |
| TANG QQ | 1 | 0.033 | BOULANGER MC | 1 | 0.033 |
| TANG QL | 1 | 0.033 | BOUHASSIRA E | 1 | 0.033 |
| TANG QF | 1 | 0.033 | BOUCHAREB R | 1 | 0.033 |
| TANG MQ | 1 | 0.033 | BOUCHARD L | 1 | 0.033 |
| TANG LY | 1 | 0.033 | BOTHEREL N | 1 | 0.033 |
| TANG LH | 1 | 0.033 | BOSSIO S | 1 | 0.033 |
| TANG JW | 1 | 0.033 | BOSSE Y | 1 | 0.033 |
| TANG JJ | 1 | 0.033 | BOSQUE A | 1 | 0.033 |
| TANG JH | 1 | 0.033 | BOSCH N | 1 | 0.033 |
| TANG HX | 1 | 0.033 | BOROWSKY ML | 1 | 0.033 |
| TANG HS | 1 | 0.033 | BORNER K | 1 | 0.033 |
| TANG HM | 1 | 0.033 | BORKOWSKA E | 1 | 0.033 |
| TANG HK | 1 | 0.033 | BORKHARDT A | 1 | 0.033 |
| TANG H | 1 | 0.033 | BORGNA V | 1 | 0.033 |
| TANG GN | 1 | 0.033 | BOREK Z | 1 | 0.033 |
| TANG GH | 1 | 0.033 | BORDO D | 1 | 0.033 |
| TANG FC | 1 | 0.033 | BOOY EP | 1 | 0.033 |
| TANG DE | 1 | 0.033 | BOOTON R | 1 | 0.033 |
| TANG CP | 1 | 0.033 | BOOTH CJ | 1 | 0.033 |
| TANG CL | 1 | 0.033 | BOOTEN S | 1 | 0.033 |
| TANASA B | 1 | 0.033 | BOO K | 1 | 0.033 |
| TANAKA K | 1 | 0.033 | BONVENTO G | 1 | 0.033 |
| TANAKA F | 1 | 0.033 | BONNEAU R | 1 | 0.033 |
| TANABE H | 1 | 0.033 | BONNAL RJP | 1 | 0.033 |
| TAN Z | 1 | 0.033 | BONI V | 1 | 0.033 |
| TAN YL | 1 | 0.033 | BONFERT T | 1 | 0.033 |
| TAN WL | 1 | 0.033 | BONEV B | 1 | 0.033 |
| TAN W | 1 | 0.033 | BONETTI A | 1 | 0.033 |
| TAN T | 1 | 0.033 | BONASIO R | 1 | 0.033 |
| TAN SK | 1 | 0.033 | BONAMINO MH | 1 | 0.033 |
| TAN S | 1 | 0.033 | BOLTON EM | 1 | 0.033 |
| TAN MY | 1 | 0.033 | BOLTANA S | 1 | 0.033 |
| TAN MW | 1 | 0.033 | BOLESANI E | 1 | 0.033 |
| TAN M | 1 | 0.033 | BOLDANOVA T | 1 | 0.033 |
| TAN LP | 1 | 0.033 | BOLAND CR | 1 | 0.033 |
| TAN KB | 1 | 0.033 | BOGSTED M | 1 | 0.033 |
| TAN JR | 1 | 0.033 | BOERNER S | 1 | 0.033 |
| TAN JM | 1 | 0.033 | BOECKX B | 1 | 0.033 |
| TAN HW | 1 | 0.033 | BODKER JS | 1 | 0.033 |
| TAN HB | 1 | 0.033 | BODINE DM | 1 | 0.033 |
| TAN G | 1 | 0.033 | BODEGA B | 1 | 0.033 |
| TAN FW | 1 | 0.033 | BOBZEAN SAM | 1 | 0.033 |
| TAN DL | 1 | 0.033 | BO CW | 1 | 0.033 |
| TAN CF | 1 | 0.033 | BLYTHE J | 1 | 0.033 |
| TAN BX | 1 | 0.033 | BLYTHE AJ | 1 | 0.033 |
| TAN BCM | 1 | 0.033 | BLUMENTHAL I | 1 | 0.033 |
| TAMMELA TLJ | 1 | 0.033 | BLUMENTHAL E | 1 | 0.033 |
| TAMMARO R | 1 | 0.033 | BLUM W | 1 | 0.033 |
| TAMBAS M | 1 | 0.033 | BLOTSKY D | 1 | 0.033 |
| TAMAI K | 1 | 0.033 | BLOOMFIELD CD | 1 | 0.033 |
| TAM HW | 1 | 0.033 | BLONDIN P | 1 | 0.033 |
| TALLACK MR | 1 | 0.033 | BLONDEAU JJC | 1 | 0.033 |
| TALKOWSKI ME | 1 | 0.033 | BLOBEL GA | 1 | 0.033 |
| TAKI T | 1 | 0.033 | BLIN J | 1 | 0.033 |
| TAKENAKA K | 1 | 0.033 | BLENCOWE BJ | 1 | 0.033 |
| TAKEDA Y | 1 | 0.033 | BLAU CA | 1 | 0.033 |
| TAKATA R | 1 | 0.033 | BLANCO M | 1 | 0.033 |
| TAKAO M | 1 | 0.033 | BLAIR D | 1 | 0.033 |
| TAKAHASHI Y | 1 | 0.033 | BLACKWELL JM | 1 | 0.033 |
| TAKAHASHI S | 1 | 0.033 | BLACHLY JS | 1 | 0.033 |
| TAKAHASHI N | 1 | 0.033 | BIZOT M | 1 | 0.033 |
| TAKAHASHI H | 1 | 0.033 | BIXBY JL | 1 | 0.033 |
| TAKAHASHI E | 1 | 0.033 | BITZER M | 1 | 0.033 |
| TAKAGI K | 1 | 0.033 | BITSCH N | 1 | 0.033 |
| TAISHI P | 1 | 0.033 | BISCEGLIE L | 1 | 0.033 |
| TAGUCHI A | 1 | 0.033 | BIRRER MJ | 1 | 0.033 |
| TAGHON T | 1 | 0.033 | BIONE S | 1 | 0.033 |
| TACHIKAWA K | 1 | 0.033 | BINIWALE R | 1 | 0.033 |
| TABORDA CC | 1 | 0.033 | BIN-ZHOU | 1 | 0.033 |
| TABEBORDBAR M | 1 | 0.033 | BIN WANG Z | 1 | 0.033 |
| TABATABAI ZL | 1 | 0.033 | BIN MOHAMAD S | 1 | 0.033 |
| TA N | 1 | 0.033 | BILLEREY C | 1 | 0.033 |
| SZELL M | 1 | 0.033 | BILBAO JR | 1 | 0.033 |
| SZELE F | 1 | 0.033 | BILAL E | 1 | 0.033 |
| SZCZESNA K | 1 | 0.033 | BIERY MC | 1 | 0.033 |
| SZCZEPANOWSKI M | 1 | 0.033 | BIELIK L | 1 | 0.033 |
| SZARVAS T | 1 | 0.033 | BIE BB | 1 | 0.033 |
| SYRETT CM | 1 | 0.033 | BIDDLE FG | 1 | 0.033 |
| SYED J | 1 | 0.033 | BIDA O | 1 | 0.033 |
| SWYTER KR | 1 | 0.033 | BICKEL P | 1 | 0.033 |
| SWINDELL WR | 1 | 0.033 | BICKEBOELLER H | 1 | 0.033 |
| SWEREDOSKI MJ | 1 | 0.033 | BICCIATO S | 1 | 0.033 |
| SWERDEL MR | 1 | 0.033 | BIANCHESSI V | 1 | 0.033 |
| SWEET-CORDERO EA | 1 | 0.033 | BIAN ZH | 1 | 0.033 |
| SWEENEY RT | 1 | 0.033 | BIAN YS | 1 | 0.033 |
| SWARUP V | 1 | 0.033 | BIAN XW | 1 | 0.033 |
| SWARR DT | 1 | 0.033 | BIAN XJ | 1 | 0.033 |
| SWARBRICK A | 1 | 0.033 | BIAN W | 1 | 0.033 |
| SVOBODA P | 1 | 0.033 | BIAGIOLI M | 1 | 0.033 |
| SUZUKI N | 1 | 0.033 | BI ZG | 1 | 0.033 |
| SUZUKI M | 1 | 0.033 | BI ZF | 1 | 0.033 |
| SUZUKI A | 1 | 0.033 | BI YY | 1 | 0.033 |
| SUYAMA S | 1 | 0.033 | BI XL | 1 | 0.033 |
| SUTHERLAND RL | 1 | 0.033 | BI LT | 1 | 0.033 |
| SUSZTAK K | 1 | 0.033 | BI LJ | 1 | 0.033 |
| SUSLUER SY | 1 | 0.033 | BI JH | 1 | 0.033 |
| SURKA CF | 1 | 0.033 | BI JB | 1 | 0.033 |
| SURKA C | 1 | 0.033 | BI HS | 1 | 0.033 |
| SURFACE LE | 1 | 0.033 | BI FR | 1 | 0.033 |
| SURENDRANATH V | 1 | 0.033 | BI F | 1 | 0.033 |
| SURACE EM | 1 | 0.033 | BI CW | 1 | 0.033 |
| SUO MM | 1 | 0.033 | BHUIYAN MIH | 1 | 0.033 |
| SUO J | 1 | 0.033 | BHIN J | 1 | 0.033 |
| SUNG BH | 1 | 0.033 | BHATTACHARYYA S | 1 | 0.033 |
| SUNDRAM U | 1 | 0.033 | BHATTACHARYA PK | 1 | 0.033 |
| SUNDARAM GM | 1 | 0.033 | BHATTACHARJEE G | 1 | 0.033 |
| SUNAMURA N | 1 | 0.033 | BHATTA A | 1 | 0.033 |
| SUN ZR | 1 | 0.033 | BHARTI SK | 1 | 0.033 |
| SUN ZQ | 1 | 0.033 | BHANDARI A | 1 | 0.033 |
| SUN ZK | 1 | 0.033 | BHAGAT TD | 1 | 0.033 |
| SUN ZF | 1 | 0.033 | BHAGAT T | 1 | 0.033 |
| SUN YY | 1 | 0.033 | BHAGAT G | 1 | 0.033 |
| SUN YX | 1 | 0.033 | BHADURI A | 1 | 0.033 |
| SUN YP | 1 | 0.033 | BEVILACQUA V | 1 | 0.033 |
| SUN YL | 1 | 0.033 | BEVERIDGE NJ | 1 | 0.033 |
| SUN YK | 1 | 0.033 | BETTS JA | 1 | 0.033 |
| SUN XW | 1 | 0.033 | BETIST MC | 1 | 0.033 |
| SUN XT | 1 | 0.033 | BETANCUR JG | 1 | 0.033 |
| SUN XP | 1 | 0.033 | BERTOLOTTI M | 1 | 0.033 |
| SUN XH | 1 | 0.033 | BERTHELSEN CHB | 1 | 0.033 |
| SUN XD | 1 | 0.033 | BERRONDO C | 1 | 0.033 |
| SUN XB | 1 | 0.033 | BERNSTEIN D | 1 | 0.033 |
| SUN WX | 1 | 0.033 | BERNSTEIN BE | 1 | 0.033 |
| SUN SY | 1 | 0.033 | BERNHART SH | 1 | 0.033 |
| SUN SS | 1 | 0.033 | BERNAY B | 1 | 0.033 |
| SUN SG | 1 | 0.033 | BERNARD P | 1 | 0.033 |
| SUN RX | 1 | 0.033 | BERLETCH JB | 1 | 0.033 |
| SUN RC | 1 | 0.033 | BERKOVA Z | 1 | 0.033 |
| SUN QX | 1 | 0.033 | BERKLEY JA | 1 | 0.033 |
| SUN QW | 1 | 0.033 | BERINGER M | 1 | 0.033 |
| SUN QL | 1 | 0.033 | BERGMANN KA | 1 | 0.033 |
| SUN QF | 1 | 0.033 | BERGMAN H | 1 | 0.033 |
| SUN QC | 1 | 0.033 | BERGER N | 1 | 0.033 |
| SUN PY | 1 | 0.033 | BERESTEN SF | 1 | 0.033 |
| SUN NX | 1 | 0.033 | BERENGUT J | 1 | 0.033 |
| SUN NH | 1 | 0.033 | BERDEL WE | 1 | 0.033 |
| SUN MH | 1 | 0.033 | BERCHUCK A | 1 | 0.033 |
| SUN LY | 1 | 0.033 | BEQQALI A | 1 | 0.033 |
| SUN LX | 1 | 0.033 | BENVENUTI LA | 1 | 0.033 |
| SUN LS | 1 | 0.033 | BENNETT AM | 1 | 0.033 |
| SUN LJ | 1 | 0.033 | BENFEY PN | 1 | 0.033 |
| SUN LC | 1 | 0.033 | BENARD M | 1 | 0.033 |
| SUN KT | 1 | 0.033 | BEN QW | 1 | 0.033 |
| SUN JZ | 1 | 0.033 | BELTRAN H | 1 | 0.033 |
| SUN JW | 1 | 0.033 | BELTRAMI AP | 1 | 0.033 |
| SUN JQ | 1 | 0.033 | BELMONTE JCI | 1 | 0.033 |
| SUN JL | 1 | 0.033 | BELLONE M | 1 | 0.033 |
| SUN JH | 1 | 0.033 | BELLON A | 1 | 0.033 |
| SUN JF | 1 | 0.033 | BELLENGUEZ C | 1 | 0.033 |
| SUN HQ | 1 | 0.033 | BELL RD | 1 | 0.033 |
| SUN HJ | 1 | 0.033 | BELL JL | 1 | 0.033 |
| SUN HG | 1 | 0.033 | BELGARD TG | 1 | 0.033 |
| SUN DX | 1 | 0.033 | BEILSTEIN MA | 1 | 0.033 |
| SUN DQ | 1 | 0.033 | BEI XY | 1 | 0.033 |
| SUN DL | 1 | 0.033 | BEHLKE MA | 1 | 0.033 |
| SUN DF | 1 | 0.033 | BEERMANN J | 1 | 0.033 |
| SUN DD | 1 | 0.033 | BEER DG | 1 | 0.033 |
| SUN CZ | 1 | 0.033 | BEDROSIAN I | 1 | 0.033 |
| SUN CT | 1 | 0.033 | BEDENIS R | 1 | 0.033 |
| SUN CM | 1 | 0.033 | BECKMANN T | 1 | 0.033 |
| SUN AJ | 1 | 0.033 | BECKHAM CJ | 1 | 0.033 |
| SUKUMAR S | 1 | 0.033 | BECK S | 1 | 0.033 |
| SUI SH | 1 | 0.033 | BECK B | 1 | 0.033 |
| SUI MH | 1 | 0.033 | BECK AH | 1 | 0.033 |
| SUGRUE SP | 1 | 0.033 | BECHOR M | 1 | 0.033 |
| SUGIYAMA M | 1 | 0.033 | BEBAWEE R | 1 | 0.033 |
| SUGIYAMA H | 1 | 0.033 | BEAVER LM | 1 | 0.033 |
| SUGIURA M | 1 | 0.033 | BEAULIEU YB | 1 | 0.033 |
| SUGIMASA H | 1 | 0.033 | BEAUDET AL | 1 | 0.033 |
| SUGDEN D | 1 | 0.033 | BEATO M | 1 | 0.033 |
| SUGAWARA N | 1 | 0.033 | BEARD L | 1 | 0.033 |
| SUGANO S | 1 | 0.033 | BAZIN J | 1 | 0.033 |
| SUGAMURA K | 1 | 0.033 | BAYOGLU B | 1 | 0.033 |
| SUGAI T | 1 | 0.033 | BAVENDIEK U | 1 | 0.033 |
| SUDA S | 1 | 0.033 | BAUTERS C | 1 | 0.033 |
| SUBRAMANIYAN S | 1 | 0.033 | BAUMBUSCH LO | 1 | 0.033 |
| SUBRAMANIAN SL | 1 | 0.033 | BAUM R | 1 | 0.033 |
| SUBRAMANIAM R | 1 | 0.033 | BAUERSACHS J | 1 | 0.033 |
| SUBIRANA JA | 1 | 0.033 | BAUDERLIQUE-LE ROY H | 1 | 0.033 |
| SU ZY | 1 | 0.033 | BAUD D | 1 | 0.033 |
| SU ZR | 1 | 0.033 | BATOVA A | 1 | 0.033 |
| SU ZJ | 1 | 0.033 | BATOREU NM | 1 | 0.033 |
| SU YW | 1 | 0.033 | BATKAI S | 1 | 0.033 |
| SU YR | 1 | 0.033 | BATA-CSORGO Z | 1 | 0.033 |
| SU XY | 1 | 0.033 | BAST RC | 1 | 0.033 |
| SU XH | 1 | 0.033 | BASSO G | 1 | 0.033 |
| SU WQ | 1 | 0.033 | BASSI PF | 1 | 0.033 |
| SU WM | 1 | 0.033 | BASSETT AR | 1 | 0.033 |
| SU W | 1 | 0.033 | BASHYAL A | 1 | 0.033 |
| SU SZ | 1 | 0.033 | BARTON MC | 1 | 0.033 |
| SU SF | 1 | 0.033 | BARTOLOMEI MS | 1 | 0.033 |
| SU S | 1 | 0.033 | BARTHOLOMEUSZ G | 1 | 0.033 |
| SU PX | 1 | 0.033 | BARTENSTEIN M | 1 | 0.033 |
| SU N | 1 | 0.033 | BARTELS CF | 1 | 0.033 |
| SU M | 1 | 0.033 | BARTEL CA | 1 | 0.033 |
| SU LP | 1 | 0.033 | BARRETTE TR | 1 | 0.033 |
| SU K | 1 | 0.033 | BARRES BA | 1 | 0.033 |
| SU JZ | 1 | 0.033 | BARRA J | 1 | 0.033 |
| SU HR | 1 | 0.033 | BARON MA | 1 | 0.033 |
| SU HJ | 1 | 0.033 | BARNHILL LM | 1 | 0.033 |
| SU G | 1 | 0.033 | BARNES PJ | 1 | 0.033 |
| SU FX | 1 | 0.033 | BARMAN P | 1 | 0.033 |
| SU DN | 1 | 0.033 | BARGAY J | 1 | 0.033 |
| SU CQ | 1 | 0.033 | BARDOU F | 1 | 0.033 |
| SU CH | 1 | 0.033 | BARBOUTH D | 1 | 0.033 |
| STUNNENBERG HG | 1 | 0.033 | BARBIER O | 1 | 0.033 |
| STUART PE | 1 | 0.033 | BARAJAS BC | 1 | 0.033 |
| STRUHL K | 1 | 0.033 | BAR D | 1 | 0.033 |
| STROZZI F | 1 | 0.033 | BAR C | 1 | 0.033 |
| STRONG CD | 1 | 0.033 | BAO ZS | 1 | 0.033 |
| STRICKER SH | 1 | 0.033 | BAO ZM | 1 | 0.033 |
| STRELTSOV A | 1 | 0.033 | BAO WS | 1 | 0.033 |
| STRAUSS-SOUKUP JK | 1 | 0.033 | BAO W | 1 | 0.033 |
| STRATIGI K | 1 | 0.033 | BAO TP | 1 | 0.033 |
| STRANGE A | 1 | 0.033 | BAO NR | 1 | 0.033 |
| STRAHLE U | 1 | 0.033 | BAO ML | 1 | 0.033 |
| STRAHL BD | 1 | 0.033 | BAO MH | 1 | 0.033 |
| STONE RM | 1 | 0.033 | BAO LL | 1 | 0.033 |
| STONE A | 1 | 0.033 | BAO LD | 1 | 0.033 |
| STOJADINOVIC A | 1 | 0.033 | BAO L | 1 | 0.033 |
| STOECKLIN G | 1 | 0.033 | BAO JT | 1 | 0.033 |
| STOCSITS RR | 1 | 0.033 | BAO JQ | 1 | 0.033 |
| STIRPARO GG | 1 | 0.033 | BAO JM | 1 | 0.033 |
| STILLMAN DJ | 1 | 0.033 | BAO JJ | 1 | 0.033 |
| STILGENBAUER S | 1 | 0.033 | BAO J | 1 | 0.033 |
| STHANDIER O | 1 | 0.033 | BAO HC | 1 | 0.033 |
| STEWART KM | 1 | 0.033 | BAO H | 1 | 0.033 |
| STEWART DJ | 1 | 0.033 | BANO AS | 1 | 0.033 |
| STEWARD CA | 1 | 0.033 | BANKSON JA | 1 | 0.033 |
| STETEFELD J | 1 | 0.033 | BANIOL M | 1 | 0.033 |
| STERNER KN | 1 | 0.033 | BANGMA CH | 1 | 0.033 |
| STERN-GINOSSAR N | 1 | 0.033 | BANG C | 1 | 0.033 |
| STERGIOPOULOS A | 1 | 0.033 | BANFI S | 1 | 0.033 |
| STEPHEN S | 1 | 0.033 | BANFAI B | 1 | 0.033 |
| STEITZ JA | 1 | 0.033 | BANERJEE S | 1 | 0.033 |
| STEINHILBER D | 1 | 0.033 | BANDRES E | 1 | 0.033 |
| STEINHAUSER ML | 1 | 0.033 | BANDER NH | 1 | 0.033 |
| STEIN JL | 1 | 0.033 | BANDARU S | 1 | 0.033 |
| STEIN J | 1 | 0.033 | BAND G | 1 | 0.033 |
| STEIN GS | 1 | 0.033 | BANCEL B | 1 | 0.033 |
| STEGMAIER K | 1 | 0.033 | BAMMANN H | 1 | 0.033 |
| STEFFLOVA K | 1 | 0.033 | BALZERGUE S | 1 | 0.033 |
| STEFANSSON K | 1 | 0.033 | BALLARINO A | 1 | 0.033 |
| STEFANSSON G | 1 | 0.033 | BALLANTYNE RL | 1 | 0.033 |
| STEARNS V | 1 | 0.033 | BALLANTYNE M | 1 | 0.033 |
| STATT S | 1 | 0.033 | BALL M | 1 | 0.033 |
| STAQUICINI FI | 1 | 0.033 | BALDERES DA | 1 | 0.033 |
| STAPLETON K | 1 | 0.033 | BALCI T | 1 | 0.033 |
| STANIK M | 1 | 0.033 | BALBIN OA | 1 | 0.033 |
| STANCHEVA I | 1 | 0.033 | BALASUBRAMANIAN S | 1 | 0.033 |
| STAMPFER MR | 1 | 0.033 | BALAS MM | 1 | 0.033 |
| STAMOVA BS | 1 | 0.033 | BAKKUS M | 1 | 0.033 |
| STAMER WD | 1 | 0.033 | BAKKER AM | 1 | 0.033 |
| STAIBANO S | 1 | 0.033 | BAKKE I | 1 | 0.033 |
| STAHEL RA | 1 | 0.033 | BAKER RT | 1 | 0.033 |
| STADLER PF | 1 | 0.033 | BAKER KE | 1 | 0.033 |
| STADLER HS | 1 | 0.033 | BAKER JC | 1 | 0.033 |
| SRIVASTAVA S | 1 | 0.033 | BAILIS W | 1 | 0.033 |
| SRIVASTAVA D | 1 | 0.033 | BAILEY-SERRES J | 1 | 0.033 |
| SRINIVASAN T | 1 | 0.033 | BAILEY TL | 1 | 0.033 |
| SRINIVAS M | 1 | 0.033 | BAILEY SD | 1 | 0.033 |
| SRIKANTAN S | 1 | 0.033 | BAILEY PJ | 1 | 0.033 |
| SREENIVASAMURTHY S | 1 | 0.033 | BAI ZG | 1 | 0.033 |
| SPRUYT N | 1 | 0.033 | BAI YX | 1 | 0.033 |
| SPRINGER NM | 1 | 0.033 | BAI YS | 1 | 0.033 |
| SPORILD I | 1 | 0.033 | BAI YQ | 1 | 0.033 |
| SPINAZZI M | 1 | 0.033 | BAI YN | 1 | 0.033 |
| SPILIANAKIS CG | 1 | 0.033 | BAI YH | 1 | 0.033 |
| SPIEKER T | 1 | 0.033 | BAI XZ | 1 | 0.033 |
| SPENCER SP | 1 | 0.033 | BAI XW | 1 | 0.033 |
| SPENCER CCA | 1 | 0.033 | BAI XN | 1 | 0.033 |
| SPENCE M | 1 | 0.033 | BAI XD | 1 | 0.033 |
| SPEKTOR R | 1 | 0.033 | BAI X | 1 | 0.033 |
| SPEICHER DW | 1 | 0.033 | BAI WL | 1 | 0.033 |
| SPEERS C | 1 | 0.033 | BAI WB | 1 | 0.033 |
| SPADARO PA | 1 | 0.033 | BAI RB | 1 | 0.033 |
| SOUQUERE S | 1 | 0.033 | BAI CX | 1 | 0.033 |
| SOUMILLON M | 1 | 0.033 | BAI C | 1 | 0.033 |
| SOULIERE MF | 1 | 0.033 | BAI BY | 1 | 0.033 |
| SOULIER J | 1 | 0.033 | BAHRING S | 1 | 0.033 |
| SOUKUP D | 1 | 0.033 | BAHLER J | 1 | 0.033 |
| SOTO-REYES E | 1 | 0.033 | BAGEDATI D | 1 | 0.033 |
| SOSSEY-ALAOUI K | 1 | 0.033 | BAER MR | 1 | 0.033 |
| SOSINSKA P | 1 | 0.033 | BAEK SH | 1 | 0.033 |
| SOSHNEV AA | 1 | 0.033 | BADVE S | 1 | 0.033 |
| SORIN V | 1 | 0.033 | BADI I | 1 | 0.033 |
| SOREQ L | 1 | 0.033 | BADAL SS | 1 | 0.033 |
| SOREQ H | 1 | 0.033 | BACOLLA A | 1 | 0.033 |
| SORENSEN PHB | 1 | 0.033 | BACKIYARANI S | 1 | 0.033 |
| SOPTA M | 1 | 0.033 | BACHAND F | 1 | 0.033 |
| SOON PSH | 1 | 0.033 | BACCI L | 1 | 0.033 |
| SONG ZW | 1 | 0.033 | BACCARELLI AA | 1 | 0.033 |
| SONG ZM | 1 | 0.033 | BACAL F | 1 | 0.033 |
| SONG Z | 1 | 0.033 | BAATSEN P | 1 | 0.033 |
| SONG YS | 1 | 0.033 | AZAZY AEM | 1 | 0.033 |
| SONG YR | 1 | 0.033 | AZARGASHB E | 1 | 0.033 |
| SONG YQ | 1 | 0.033 | AYANGA BA | 1 | 0.033 |
| SONG YL | 1 | 0.033 | AYAD NG | 1 | 0.033 |
| SONG YD | 1 | 0.033 | AY F | 1 | 0.033 |
| SONG XW | 1 | 0.033 | AVRUTIN A | 1 | 0.033 |
| SONG XT | 1 | 0.033 | AVRAMOPOULOS P | 1 | 0.033 |
| SONG XQ | 1 | 0.033 | AVITABILE C | 1 | 0.033 |
| SONG XH | 1 | 0.033 | AVILA R | 1 | 0.033 |
| SONG XF | 1 | 0.033 | AVCI CB | 1 | 0.033 |
| SONG XB | 1 | 0.033 | AURE MR | 1 | 0.033 |
| SONG WH | 1 | 0.033 | AUNG T | 1 | 0.033 |
| SONG WB | 1 | 0.033 | AUMAYR K | 1 | 0.033 |
| SONG TQ | 1 | 0.033 | AUGOFF K | 1 | 0.033 |
| SONG RS | 1 | 0.033 | AUERBACH W | 1 | 0.033 |
| SONG RH | 1 | 0.033 | AU SLK | 1 | 0.033 |
| SONG QX | 1 | 0.033 | AU PCK | 1 | 0.033 |
| SONG QL | 1 | 0.033 | ATTANASIO M | 1 | 0.033 |
| SONG PP | 1 | 0.033 | ATTAL S | 1 | 0.033 |
| SONG NX | 1 | 0.033 | ATLASI Y | 1 | 0.033 |
| SONG M | 1 | 0.033 | ATKINSON SR | 1 | 0.033 |
| SONG LY | 1 | 0.033 | ATKINSON MJ | 1 | 0.033 |
| SONG LL | 1 | 0.033 | ATIANJOH FE | 1 | 0.033 |
| SONG KS | 1 | 0.033 | ATANASIO A | 1 | 0.033 |
| SONG JZ | 1 | 0.033 | ASTROLOGO S | 1 | 0.033 |
| SONG JW | 1 | 0.033 | ASKARIAN-AMIRI M | 1 | 0.033 |
| SONG JS | 1 | 0.033 | ASIF A | 1 | 0.033 |
| SONG J | 1 | 0.033 | ASHOURI A | 1 | 0.033 |
| SONG HZ | 1 | 0.033 | ASHLEY-KOCH AE | 1 | 0.033 |
| SONG GL | 1 | 0.033 | ASHLEY E | 1 | 0.033 |
| SONG GB | 1 | 0.033 | ASHIKAWA K | 1 | 0.033 |
| SONG G | 1 | 0.033 | ASHIKAGA T | 1 | 0.033 |
| SONG FB | 1 | 0.033 | ASHIDA M | 1 | 0.033 |
| SONG F | 1 | 0.033 | ASHER VA | 1 | 0.033 |
| SONG E | 1 | 0.033 | ASGHARZADEH S | 1 | 0.033 |
| SONG CW | 1 | 0.033 | ASCHRAFI A | 1 | 0.033 |
| SONG CJ | 1 | 0.033 | ASAHARA H | 1 | 0.033 |
| SONG BT | 1 | 0.033 | ARZATE-MEJIA R | 1 | 0.033 |
| SOLTYS DT | 1 | 0.033 | ARTHANARI Y | 1 | 0.033 |
| SOLOWAY PD | 1 | 0.033 | ARTANDI SE | 1 | 0.033 |
| SOLOVIEFF N | 1 | 0.033 | ARRON ST | 1 | 0.033 |
| SOLOMON TH | 1 | 0.033 | ARRON S | 1 | 0.033 |
| SOLDA G | 1 | 0.033 | ARRIAGA-CANON C | 1 | 0.033 |
| SOKOLOWSKI N | 1 | 0.033 | AROS CJ | 1 | 0.033 |
| SOHN HA | 1 | 0.033 | ARONOW B | 1 | 0.033 |
| SOH J | 1 | 0.033 | ARMUGAM A | 1 | 0.033 |
| SOARES F | 1 | 0.033 | ARMSTRONG R | 1 | 0.033 |
| SNODDY JR | 1 | 0.033 | ARMOUR CD | 1 | 0.033 |
| SNIJDERS AM | 1 | 0.033 | ARMANT O | 1 | 0.033 |
| SMOLLER JW | 1 | 0.033 | ARMAIZ-PENA GN | 1 | 0.033 |
| SMOLLE MA | 1 | 0.033 | ARIYO EO | 1 | 0.033 |
| SMOLLE M | 1 | 0.033 | ARITA T | 1 | 0.033 |
| SMOLA MJ | 1 | 0.033 | ARIEL I | 1 | 0.033 |
| SMITS G | 1 | 0.033 | ARIEL F | 1 | 0.033 |
| SMITH SR | 1 | 0.033 | ARGANI P | 1 | 0.033 |
| SMITH MA | 1 | 0.033 | ARFAT Y | 1 | 0.033 |
| SMITH DL | 1 | 0.033 | ARD R | 1 | 0.033 |
| SMITH CA | 1 | 0.033 | ARCHER K | 1 | 0.033 |
| SMIT JW | 1 | 0.033 | ARBUTHNOT PB | 1 | 0.033 |
| SMERDOU C | 1 | 0.033 | ARBUCKLE S | 1 | 0.033 |
| SMEKALOVA EM | 1 | 0.033 | ARAUJO JC | 1 | 0.033 |
| SMART RC | 1 | 0.033 | ARASE M | 1 | 0.033 |
| SMART CE | 1 | 0.033 | ARAP W | 1 | 0.033 |
| SMALLING R | 1 | 0.033 | ARAI T | 1 | 0.033 |
| SLYSKOVA J | 1 | 0.033 | ARAGONES J | 1 | 0.033 |
| SLOAN SA | 1 | 0.033 | ARABI L | 1 | 0.033 |
| SLAVOV N | 1 | 0.033 | ARAB K | 1 | 0.033 |
| SLACK FJ | 1 | 0.033 | APARICIO-PRAT E | 1 | 0.033 |
| SKORIC A | 1 | 0.033 | AOI J | 1 | 0.033 |
| SKOGERBOE G | 1 | 0.033 | AO H | 1 | 0.033 |
| SKLAR P | 1 | 0.033 | ANTONIOU D | 1 | 0.033 |
| SKAWRAN B | 1 | 0.033 | ANTONARAKIS SE | 1 | 0.033 |
| SIVE H | 1 | 0.033 | ANTHONY DD | 1 | 0.033 |
| SIVANANTHAN J | 1 | 0.033 | ANSARI A | 1 | 0.033 |
| SIROKMAN K | 1 | 0.033 | ANNARATONE L | 1 | 0.033 |
| SIREY TM | 1 | 0.033 | ANNALA M | 1 | 0.033 |
| SIREY T | 1 | 0.033 | ANGRAND PO | 1 | 0.033 |
| SINGH VK | 1 | 0.033 | ANGIONI C | 1 | 0.033 |
| SINGH SX | 1 | 0.033 | ANGELINI GD | 1 | 0.033 |
| SINGH N | 1 | 0.033 | ANG YS | 1 | 0.033 |
| SINGER ZS | 1 | 0.033 | ANDREWS NC | 1 | 0.033 |
| SINGER HA | 1 | 0.033 | ANDREW M | 1 | 0.033 |
| SINDY K | 1 | 0.033 | ANDREASSEN M | 1 | 0.033 |
| SINCLAIR AH | 1 | 0.033 | ANDRE C | 1 | 0.033 |
| SIMS JS | 1 | 0.033 | ANDERSSON PO | 1 | 0.033 |
| SIMS D | 1 | 0.033 | ANDERSON SM | 1 | 0.033 |
| SIMS AH | 1 | 0.033 | ANDERSON SK | 1 | 0.033 |
| SIMPSON RJ | 1 | 0.033 | ANDERSON L | 1 | 0.033 |
| SIMPSON D | 1 | 0.033 | ANDERSON KM | 1 | 0.033 |
| SIMPSON CG | 1 | 0.033 | ANDERSEN RE | 1 | 0.033 |
| SIMONS C | 1 | 0.033 | ANDERSEN JB | 1 | 0.033 |
| SIMONIS M | 1 | 0.033 | ANDER BP | 1 | 0.033 |
| SIMCHOVITZ A | 1 | 0.033 | ANASTASOV N | 1 | 0.033 |
| SIMA H | 1 | 0.033 | AN YH | 1 | 0.033 |
| SIM H | 1 | 0.033 | AN XL | 1 | 0.033 |
| SILVERSIDES D | 1 | 0.033 | AN XG | 1 | 0.033 |
| SILVERMAN IM | 1 | 0.033 | AN XB | 1 | 0.033 |
| SILVEIRA RA | 1 | 0.033 | AN L | 1 | 0.033 |
| SILVA-FISHER JM | 1 | 0.033 | AN GY | 1 | 0.033 |
| SILVA A | 1 | 0.033 | AN CM | 1 | 0.033 |
| SIGURDSSON A | 1 | 0.033 | AMOS CI | 1 | 0.033 |
| SIGDEL KR | 1 | 0.033 | AMORT T | 1 | 0.033 |
| SIEUWERTS AM | 1 | 0.033 | AMON A | 1 | 0.033 |
| SIERRA-MIRANDA M | 1 | 0.033 | AMIT-AVRAHAM I | 1 | 0.033 |
| SIERRA J | 1 | 0.033 | AMIN V | 1 | 0.033 |
| SIEGEL CT | 1 | 0.033 | AMIES I | 1 | 0.033 |
| SIEGEL AB | 1 | 0.033 | AMICONE L | 1 | 0.033 |
| SIEBERT R | 1 | 0.033 | AMELLAH S | 1 | 0.033 |
| SIEBERT A | 1 | 0.033 | AMBROSIO ALB | 1 | 0.033 |
| SIEBENALER RF | 1 | 0.033 | AMARANTE-MENDES GP | 1 | 0.033 |
| SIE D | 1 | 0.033 | AMANT F | 1 | 0.033 |
| SIDMAN RL | 1 | 0.033 | AMADOZ A | 1 | 0.033 |
| SIDHU SB | 1 | 0.033 | ALVAREZ ML | 1 | 0.033 |
| SIDDIQUI H | 1 | 0.033 | ALVAREZ CPP | 1 | 0.033 |
| SIBBRITT T | 1 | 0.033 | ALVARADO AS | 1 | 0.033 |
| SIATECKA M | 1 | 0.033 | ALTUN M | 1 | 0.033 |
| SI ZZ | 1 | 0.033 | ALTER HJ | 1 | 0.033 |
| SI XM | 1 | 0.033 | ALSIBAI KD | 1 | 0.033 |
| SI S | 1 | 0.033 | ALMEIDA R | 1 | 0.033 |
| SI LH | 1 | 0.033 | ALMEIDA MI | 1 | 0.033 |
| SI J | 1 | 0.033 | ALMADA AE | 1 | 0.033 |
| SHYU WC | 1 | 0.033 | ALLTON K | 1 | 0.033 |
| SHUMAN S | 1 | 0.033 | ALLSHIRE RC | 1 | 0.033 |
| SHUAI CJ | 1 | 0.033 | ALLINGHAM RR | 1 | 0.033 |
| SHU XM | 1 | 0.033 | ALLHOFF W | 1 | 0.033 |
| SHU WS | 1 | 0.033 | ALIPERTI V | 1 | 0.033 |
| SHU M | 1 | 0.033 | ALI MA | 1 | 0.033 |
| SHU KH | 1 | 0.033 | ALFANO G | 1 | 0.033 |
| SHU J | 1 | 0.033 | ALEXOPOULOU D | 1 | 0.033 |
| SHROFF N | 1 | 0.033 | ALEXE G | 1 | 0.033 |
| SHOSHANI O | 1 | 0.033 | ALEXANDER R | 1 | 0.033 |
| SHORE AN | 1 | 0.033 | ALESSANDRO R | 1 | 0.033 |
| SHODA K | 1 | 0.033 | ALDO PB | 1 | 0.033 |
| SHISHKIN A | 1 | 0.033 | ALCHTAR A | 1 | 0.033 |
| SHIROKI T | 1 | 0.033 | ALBRECHT AS | 1 | 0.033 |
| SHIRASAWA S | 1 | 0.033 | ALBALA D | 1 | 0.033 |
| SHIRAISHI A | 1 | 0.033 | ALBA MM | 1 | 0.033 |
| SHIRAHIGE K | 1 | 0.033 | ALAM T | 1 | 0.033 |
| SHIOZAKI A | 1 | 0.033 | ALAM J | 1 | 0.033 |
| SHINOMURA Y | 1 | 0.033 | ALAHARI SV | 1 | 0.033 |
| SHIN YK | 1 | 0.033 | ALAHARI SK | 1 | 0.033 |
| SHIN SY | 1 | 0.033 | AL-QURAISHY S | 1 | 0.033 |
| SHIN JW | 1 | 0.033 | AL-EJEH F | 1 | 0.033 |
| SHIN HJR | 1 | 0.033 | AL HUSINI N | 1 | 0.033 |
| SHIN EJ | 1 | 0.033 | AKYUREK LM | 1 | 0.033 |
| SHIN DY | 1 | 0.033 | AKUTSU H | 1 | 0.033 |
| SHIN C | 1 | 0.033 | AKTAN M | 1 | 0.033 |
| SHIMURA Y | 1 | 0.033 | AKRAMI R | 1 | 0.033 |
| SHIMURA H | 1 | 0.033 | AKIZUKI G | 1 | 0.033 |
| SHIMOTOHNO K | 1 | 0.033 | AKIYOSHI S | 1 | 0.033 |
| SHIMOSEGAWA T | 1 | 0.033 | AKIYAMA Y | 1 | 0.033 |
| SHIMIZU R | 1 | 0.033 | AKHTAR A | 1 | 0.033 |
| SHIMIZU M | 1 | 0.033 | AKHBARI P | 1 | 0.033 |
| SHIMIZU C | 1 | 0.033 | AKERMAN M | 1 | 0.033 |
| SHIMAMURA T | 1 | 0.033 | AKERMAN I | 1 | 0.033 |
| SHIMADA T | 1 | 0.033 | AKDEMIR KC | 1 | 0.033 |
| SHIMADA M | 1 | 0.033 | AKBANY A | 1 | 0.033 |
| SHIM KS | 1 | 0.033 | AKAMATSU S | 1 | 0.033 |
| SHIH PH | 1 | 0.033 | AJI G | 1 | 0.033 |
| SHIH HT | 1 | 0.033 | AIYER S | 1 | 0.033 |
| SHIH D | 1 | 0.033 | AIVALIOTIS M | 1 | 0.033 |
| SHICHINO Y | 1 | 0.033 | AIT-SI-ALI S | 1 | 0.033 |
| SHIBATA Y | 1 | 0.033 | AISSANI B | 1 | 0.033 |
| SHIBATA S | 1 | 0.033 | AINIWAER JL | 1 | 0.033 |
| SHIBATA A | 1 | 0.033 | AIFANTIS I | 1 | 0.033 |
| SHI ZZ | 1 | 0.033 | AIELLO D | 1 | 0.033 |
| SHI ZK | 1 | 0.033 | AIELLO AE | 1 | 0.033 |
| SHI ZF | 1 | 0.033 | AIELLO A | 1 | 0.033 |
| SHI XM | 1 | 0.033 | AI ZL | 1 | 0.033 |
| SHI X | 1 | 0.033 | AI YW | 1 | 0.033 |
| SHI WF | 1 | 0.033 | AI LY | 1 | 0.033 |
| SHI W | 1 | 0.033 | AI G | 1 | 0.033 |
| SHI TM | 1 | 0.033 | AI CZ | 1 | 0.033 |
| SHI T | 1 | 0.033 | AHN YJ | 1 | 0.033 |
| SHI SS | 1 | 0.033 | AHN C | 1 | 0.033 |
| SHI SJ | 1 | 0.033 | AHMED M | 1 | 0.033 |
| SHI S | 1 | 0.033 | AHEARN TU | 1 | 0.033 |
| SHI RJ | 1 | 0.033 | AHADI A | 1 | 0.033 |
| SHI QW | 1 | 0.033 | AGUIRRE A | 1 | 0.033 |
| SHI PK | 1 | 0.033 | AGUILAR A | 1 | 0.033 |
| SHI P | 1 | 0.033 | AGRAWAL-SINGH S | 1 | 0.033 |
| SHI OM | 1 | 0.033 | AGIRRE X | 1 | 0.033 |
| SHI MM | 1 | 0.033 | AGIRRE E | 1 | 0.033 |
| SHI LX | 1 | 0.033 | AGATA K | 1 | 0.033 |
| SHI LM | 1 | 0.033 | AGARWAL SK | 1 | 0.033 |
| SHI LL | 1 | 0.033 | AGARWAL S | 1 | 0.033 |
| SHI KQ | 1 | 0.033 | AGARWAL R | 1 | 0.033 |
| SHI JZ | 1 | 0.033 | AGAMI R | 1 | 0.033 |
| SHI JY | 1 | 0.033 | AFONSO-GRUNZ F | 1 | 0.033 |
| SHI JX | 1 | 0.033 | ADRIAANS A | 1 | 0.033 |
| SHI JJ | 1 | 0.033 | ADIPRASITO JB | 1 | 0.033 |
| SHI JH | 1 | 0.033 | ADICONIS X | 1 | 0.033 |
| SHI HZ | 1 | 0.033 | ADCOCK IM | 1 | 0.033 |
| SHI HJ | 1 | 0.033 | ADAMSKI M | 1 | 0.033 |
| SHI FY | 1 | 0.033 | ADAMSKA M | 1 | 0.033 |
| SHI F | 1 | 0.033 | ADAMOSKI D | 1 | 0.033 |
| SHI CB | 1 | 0.033 | ACUTO O | 1 | 0.033 |
| SHI AM | 1 | 0.033 | ACHAWANANTAKUN R | 1 | 0.033 |
| SHEYHIDING I | 1 | 0.033 | ACH RA | 1 | 0.033 |
| SHEWAN AM | 1 | 0.033 | ACH R | 1 | 0.033 |
| SHEVCHENKO B | 1 | 0.033 | ABUDOUREYIMU A | 1 | 0.033 |
| SHERWOOD CC | 1 | 0.033 | ABUALTEEN A | 1 | 0.033 |
| SHEPHERD LE | 1 | 0.033 | ABU-AMERO S | 1 | 0.033 |
| SHENG QH | 1 | 0.033 | ABSI T | 1 | 0.033 |
| SHENG L | 1 | 0.033 | ABRIGNANI S | 1 | 0.033 |
| SHENG KW | 1 | 0.033 | ABRAIRA VE | 1 | 0.033 |
| SHENG JQ | 1 | 0.033 | ABRAHAMSSON J | 1 | 0.033 |
| SHENG HM | 1 | 0.033 | ABRAHAM E | 1 | 0.033 |
| SHENG HH | 1 | 0.033 | ABOUDEHEN K | 1 | 0.033 |
| SHEN ZS | 1 | 0.033 | ABOOBAKAR IF | 1 | 0.033 |
| SHEN ZL | 1 | 0.033 | ABO R | 1 | 0.033 |
| SHEN ZH | 1 | 0.033 | ABECASIS GR | 1 | 0.033 |
| SHEN ZB | 1 | 0.033 | ABDULAHAD W | 1 | 0.033 |
| SHEN YX | 1 | 0.033 | ABDUL KSM | 1 | 0.033 |
| SHEN YT | 1 | 0.033 | ABDEL-RAHMAN O | 1 | 0.033 |
| SHEN YP | 1 | 0.033 | ABDEL-BAKI AA | 1 | 0.033 |
| SHEN YM | 1 | 0.033 | ABBA MC | 1 | 0.033 |
| A LY | 1 | 0.033 |  |  |  |
| (11743 Authors {0} {1} value(s) outside display options.) | | | | | |
| (0 records (0.000%){0} records{1} do not contain data in the field being analyzed.) | | | | | |
